# Supplementary material for: Barriers to healthcare access among women in sub-Saharan Africa: A pooled analysis of multi-country DHS data (2019–2023)
Source: PLoS One. 2026 Feb 19;21(2):e0331328. doi: 10.1371/journal.pone.0331328 (PMC12919819; doi:10.1371/journal.pone.0331328)
Supplement: S1 Table — (DOCX) [file pone.0331328.s001.docx]

| **Variables** | **Crude Odds Ratio [COR]** | **P. value** | **[95% CI]** |
| --- | --- | --- | --- |
| **Maternal age**  15-19  20-35  36-49 | 0.86  0.86  1 | <0.001  <0.001 | [0.83, 0.89]  [0.84, 0. 88]  1 |
| **Residency**  Urban  Rural | 1  2.09 | <0.001 | 1  [2.04, 2.14] |
| **Maternal education**  No education  Primary education  Secondary & Higher | 2.40  1.52  1 | <0.001  <0.001 | [2.33, 2.46]  [1.48, 1.56]  1 |
| **Husband education**  No education  Primary education  secondary & higher | 2.43  1.55  1 | <0.001  <0.001 | [2.35, 2.51]  [1.49, 1.60]  1 |
| **Maternal occupation**  working  not working | 1  0.94 | <0.001 | 1  [0.92, 0.96] |
| **Husband occupation**  working  not working | 1  1.07 | <0.001 | [1.02, 1.12] |
| **Religion**  Muslim  Christian  Other | 1  1.32  1.08 | <0.001  <0.001 | 1  [1.29, 1.35]  [1.04, 1.11] |
| **Contraceptive utilization**  No  Yes | 1.18  1 | <0.001 | [0.82, 0.86]  1 |
| **Place of delivery**  Home  Institution | 1.84  1 | <0.001 | [1.75, 1.93]  1 |
| **ANC visit**  No visit  1-4 visit  > 4 visits | 1.16  1.05  1 | <0.002  <0.003 | [1.05, 1.27]  [1.01, 1.09]  1 |
| **Marital status**  single  Married  Other | 1  1.23  1.32 | <0.001  <0.001 | [1.20, 1.26]  [1.29, 1.36] |
| **Media exposure**  No  Yes | 1.90  1 | <0.001 | [1.85, 1.95]  1 |
| **Internet utilization**  No  Yes | 2.321822  1 | <0.001 | [2.26, 2.37]  1 |
| **Visiting health facility 12 month**  No  Yes | 1.02  1 | <0.046 | [1.00, 1.04]  1 |
| **Health insurance**  No  Yes | 1.21  1 | <0.001 | [1.18, 1.25]  1 |
| **Wealth index**  poor  middle  Rich | 3.58  2.08  1 | <0.001  <0.001 | [3.49, 3.67]  [2.02, 2.14]  1 |
| **Region SSA**  East Africa  West Africa | 1.68  1 | <0.001 | [1.65, 1.72]  1 |
| **community ANC visit**  Low  High | 0.86  1 | <0.001 | [0.85, 0.88]  1 |
| **community wealth index**  Low  High | 0.69  1 | <0.001 | [0.67, 0.70] |
| **Community media exposure**  Low  High | 0.73  1 | <0.001 | [0.72, 0.75]  1 |
| **Community literacy level**  Low  High | 0.77  1 | <0.001 | [0.75, 0.79]  1 |
