# Supplementary material for: Barriers to healthcare access among women in sub-Saharan Africa: A pooled analysis of multi-country DHS data (2019–2023)
Source: PLoS One. 2026 Feb 19;21(2):e0331328. doi: 10.1371/journal.pone.0331328 (PMC12919819; doi:10.1371/journal.pone.0331328)
Supplement: S2 Table — (DOCX) [file pone.0331328.s002.docx]

**Table 6: Stata output to check variance inflation factor and Model fitness test using AIC & BIC**

|  | |  | |  | |
| --- | --- | --- | --- | --- | --- |
| **predictor variable** | | **VIF** | | **1/VIF** | |
| Residency  urban  Rural | | 1.68 | | 0.593611 | |
| **Religion** | |  | |  | |
| Muslim | |  | |  | |
| Christian | | 1.40 | | 0.715070 | |
|  | | 1.13 | | 0.885212 | |
| **Maternal education** | |  | |  | |
| No education  Primary education | | 1.57 | | 0.636390 | |
|  | | 1.98 | | 0.504047 | |
| Husband education | |  | |  | |
| No education  Primary education | | 1.60 | | 0.623492 | |
|  | | 1.70 | | 0.589270 | |
| **marital occupation**  Working  Not working | | 1.15 | | 0.871555 | |
| **Husband occupation**  Working  Not working | | 1.12 | | 0.895415 | |
| **contraceptive use**  No  Yes | | 1.14 | | 0.880307 | |
| **Place of delivery**  Home  Institution | | 1.10 | | 0.907857 | |
| **Media exposure**  No  Yes | | 1.25 | | 0.797038 | |
| **Internet utilization**  yes  No  **Visiting health facility last 12 months**  No  Yes | | 1.41 | | 0.707831 | |
|  | | 1.03 | | 0.970220 | |
| **Health insurance**  Yes  No | | 1.45 | | 0.691150 | |
| **Wealth index** | |  | |  | |
| Poor  Middle  Rich | | 1.22  1.92 | | 0.820366  0.520101 | |
|  | |  | |  | |
| **Community ANC visit**  Low  High | | 1.06 | | 0.939197 | |
| **community wealth index**  Low  High | | 1.46 | | 0.682633 | |
| **community literacy level**  Low  High | | 1.43 | | 0.700580 | |
| **community media exposure**  Low  High | | 1.39 | | 0.721519 | |
| **Distance to health facility**  Low  High | | 1.17 | | 0.854675 | |
| **Region SSA**  East Africa  West Africa | | 1.21  1.68 | | 0.829495  0.595342 | |
|  | |  | |  | |
| Mean VIF | | **1.38** | |  | |
|  | |  | |  | |
| **AIC & BIC** | |  | |  | |
| Model Observation II (null) II (model) df | | AIC | | BIC | |
| 36,110 -122173.3 -119791.1 26 | | 239634.3 | | 239855.1 | |
