# Supplementary material for: Barriers to healthcare access among women in sub-Saharan Africa: A pooled analysis of multi-country DHS data (2019–2023)
Source: PLoS One. 2026 Feb 19;21(2):e0331328. doi: 10.1371/journal.pone.0331328 (PMC12919819; doi:10.1371/journal.pone.0331328)
Supplement: Attachment — Submitted filename: PONE-D-.pdf [file pone.0331328.s005.pdf]

PLOS ONE

Barriers to accessing health care among women in Sub-Saharan Africa: Multilevel analysis of recent demographic and health surveys from 2019-2023.

--Manuscript Draft--

Manuscript Number:	PONE-D-24-28999R2
Article Type:	Research Article
Full Title:	Barriers to accessing health care among women in Sub-Saharan Africa: Multilevel analysis of recent demographic and health surveys from 2019-2023.
Short Title:	Barriers to accessing health care among women in Sub-Saharan Africa
Corresponding Author:	Gebreeyesus Abera Zeleke, MSc<br>University of Gondar College of Medicine and Health Sciences<br>Gondar, ETHIOPIA
Keywords:	Access to healthcare; Barriers to Healthcare; Sub-Saharan Africa; Reproductive-Age Women
Abstract:	Background:<br>Barriers to accessing healthcare have significantly contributed to the high rates of maternal and child mortality and morbidity in developing regions, particularly in Sub-Saharan Africa. Access to healthcare is shaped by multiple factors, including financial constraints, geographic location, the availability of services, and the quality of care provided. This study aimed to assess the magnitude of these barriers and to identify the factors influencing healthcare access among women of reproductive age in Sub-Saharan Africa.<br>Methods:<br>This study utilized secondary data from the most recent Demographic and Health Surveys conducted between 2019 and 2023 across Sub-Saharan Africa. A weighted sample of 134,470 women of reproductive age was included in the analysis. Data were analyzed using Stata version 14, employing a multilevel mixed-effects logistic regression model. Factors associated with barriers to healthcare access were considered statistically significant at P-values less than 0.05. Adjusted odds ratios (AORs) with 95% confidence intervals (CIs) were used to interpret the results.<br>Result: In this study, the magnitude of barriers to accessing healthcare among reproductive age women were found to be 55.84% (95% CI: 55.57–56.10). Husband's educational status [AOR = 1.23, 95% CI: 1.15–1.30], Media exposure [AOR = 1.17, 95% CI: 1.11–1.24], antenatal care (ANC) visit status [AOR = 1.31, 95% CI: 1.10–1.55], and place of delivery [AOR = 1.33, 95% CI: 1.24–1.43]. Health insurance utilization [AOR = 1.18, 95% CI: 1.11–1.26], wealth index status, and visiting a health facility within the past 12 months [AOR = 0.91, 95% CI: 0.87–0.97] were individual associated variable. In addition, place of residence [AOR = 1.30, 95% CI: 1.22–1.39], community literacy level [AOR = 1.13, 95% CI: 1.03–1.24], community wealth index [AOR = 1.12, 95% CI: 1.02–1.22], and Region were associated with barriers to accessing healthcare.<br>Conclusion and recommendation: This study found that 55.55% of women in Sub-Saharan Africa experienced barriers to accessing healthcare. Educational status, media exposure, place of residence, antenatal care attendance, place of delivery, health insurance coverage, wealth index, recent visits to health facilities, community literacy levels, community wealth status, and regional differences were determinant factors. These findings highlight the need for Sub-Saharan African governments to prioritize these determinants when formulating policies and strategies aimed at reducing preventable barriers to healthcare access in the region.
Order of Authors:	gebreeyesus abera zeleke, MSc
	Enyew Getaneh Mekonen
	Bewuketu Terefe
	Mulugata Wessie
	Agazhe Aemro
	Alebachew Ferede Zegeye

Powered by Editorial Manager® and ProduXion Manager® from Aries Systems Corporation

	Mohammed Seid Ali
	Berhan Tekeba
	Tadesse Tarik Tamir
	Belayneh Shetie Workneh, MSc
**Response to Reviewers:**	very important comments were given
**Additional Information:**	
**Question**	**Response**
**Financial Disclosure**<br><br>Enter a financial disclosure statement that describes the sources of funding for the work included in this submission. Review the submission guidelines for detailed requirements. View published research articles from *PLOS ONE* for specific examples.<br><br>This statement is required for submission and **will appear in the published article** if the submission is accepted. Please make sure it is accurate.<br><br>**Funded studies**<br>Enter a statement with the following details:<br>• Initials of the authors who received each award<br>• Grant numbers awarded to each author<br>• The full name of each funder<br>• URL of each funder website<br>• Did the sponsors or funders play any role in the study design, data collection and analysis, decision to publish, or preparation of the manuscript?<br><br>Did you receive funding for this work?	The author(s) received no specific funding for this work.
**Competing Interests**<br><br>Use the instructions below to enter a competing interest statement for this submission. On behalf of all authors, disclose any competing interests that could be perceived to bias this work—acknowledging all financial support and any other relevant financial or non-financial competing interests.	The authors have declared that no competing interests exist.

Powered by Editorial Manager® and ProduXion Manager® from Aries Systems Corporation

This statement is required for submission and **will appear in the published article** if the submission is accepted. Please make sure it is accurate and that any funding sources listed in your Funding Information later in the submission form are also declared in your Financial Disclosure statement.<br><br>View published research articles from *PLOS ONE* for specific examples.<br><br>**NO authors have competing interests**<br><br>Enter: *The authors have declared that no competing interests exist.*<br><br>**Authors with competing interests**<br><br>Enter competing interest details beginning with this statement:<br><br>*I have read the journal's policy and the authors of this manuscript have the following competing interests: [insert competing interests here]*<br><br>* typeset	
**Ethics Statement**<br><br>Enter an ethics statement for this submission. This statement is required if the study involved:<br><br>• Human participants<br>• Human specimens or tissue<br>• Vertebrate animals or cephalopods<br>• Vertebrate embryos or tissues<br>• Field research<br><br>Write "N/A" if the submission does not require an ethics statement.<br><br>General guidance is provided below. Consult the submission guidelines for detailed instructions. **Make sure that all information entered here is included in the**	N/A

**Methods section of the manuscript.**<br><br>**Format for specific study types**<br><br>**Human Subject Research (involving human participants and/or tissue)**<br>• Give the name of the institutional review board or ethics committee that approved the study<br>• Include the approval number and/or a statement indicating approval of this research<br>• Indicate the form of consent obtained (written/oral) or the reason that consent was not obtained (e.g. the data were analyzed anonymously)<br><br>**Animal Research (involving vertebrate animals, embryos or tissues)**<br>• Provide the name of the Institutional Animal Care and Use Committee (IACUC) or other relevant ethics board that reviewed the study protocol, and indicate whether they approved this research or granted a formal waiver of ethical approval<br>• Include an approval number if one was obtained<br>• If the study involved *non-human primates*, add *additional details* about animal welfare and steps taken to ameliorate suffering<br>• If anesthesia, euthanasia, or any kind of animal sacrifice is part of the study, include briefly which substances and/or methods were applied<br><br>**Field Research**<br><br>Include the following details if this study involves the collection of plant, animal, or other materials from a natural setting:<br>• Field permit number<br>• Name of the institution or relevant body that granted permission	
**Data Availability**<br><br>Authors are required to make all data underlying the findings described fully available, without restriction, and from the time of publication. PLOS allows rare exceptions to address legal and ethical concerns. See the PLOS Data Policy and FAQ for detailed information.	Yes - all data are fully available without restriction

A Data Availability Statement describing where the data can be found is required at submission. Your answers to this question constitute the Data Availability Statement and **will be published in the article**, if accepted.<br><br>**Important:** Stating 'data available on request from the author' is not sufficient. If your data are only available upon request, select 'No' for the first question and explain your exceptional situation in the text box.<br><br>Do the authors confirm that all data underlying the findings described in their manuscript are fully available without restriction?	
**Describe where the data may be found in full sentences. If you are copying our sample text, replace any instances of XXX with the appropriate details.**<br><br>• If the data are **held or will be held in a public repository**, include URLs, accession numbers or DOIs. If this information will only be available after acceptance, indicate this by ticking the box below. For example: *All XXX files are available from the XXX database (accession number(s) XXX, XXX.*).<br>• If the data are all contained **within the manuscript and/or Supporting Information files**, enter the following: *All relevant data are within the manuscript and its Supporting Information files.*<br>• If neither of these applies but you are able to provide **details of access elsewhere**, with or without limitations, please do so. For example:<br><br>*Data cannot be shared publicly because of [XXX]. Data are available from the XXX Institutional Data Access / Ethics Committee (contact via XXX) for researchers who meet the criteria for access to confidential data.*<br><br>*The data underlying the results*	All relevant data are within the manuscript and its supporting information files.

presented in the study are available from (include the name of the third party and contact information or URL).<br>• This text is appropriate if the data are owned by a third party and authors do not have permission to share the data.<br><br>* typeset	
Additional data availability information:	Tick here if your circumstances are not covered by the questions above and you need the journal’s help to make your data available.

Manuscript Click here to access/download;Manuscript;Barrier to women.docx

Title: Barriers to accessing health care among women's in Sub-Saharan Africa: Multilevel Analysis of Recent Demographic and Health Survey from 2019-2023.

Gebreeyesus Abera Zeleke[1]*, Enyew Getaneh Mekonen[1], Bewuketu Terefe[2], Mulugeta Wassie[3], Agazhe Aemro[3], Alebachew Ferede Zegeye[3], Mohammed Seid Ali[4], Berhan Tekeba[4], Tadesse Tarik Tamir[4], Belayneh Shetie Workneh[5]

[1] Department of Surgical Nursing, School of Nursing, College of Medicine and Health Sciences, University of Gondar, Gondar, Ethiopia

[2] Department of Community Health Nursing, School of Nursing, College of Medicine and Health Sciences, University of Gondar, Gondar, Ethiopia.

[3] Department of Medical Nursing, School of Nursing, College of Medicine and Health Sciences, University of Gondar, Gondar, Ethiopia;

[4] Department of Pediatrics and Child Health Nursing, School of Nursing, College of Medicine and Health Sciences, University of Gondar, Gondar, Ethiopia.

5 Department of Emergency and Critical Care Nursing, School of Nursing, College of Medicine and Health Sciences, University of Gondar, Gondar, Ethiopia

Correspondence Author*
Gebreeyesus Abera Zeleke

Department of Surgical Nursing, School of Nursing, College of Medicine and Health Science, University of Gondar, Gondar, Ethiopia.

Email address: gebreeyesusabe143@gmail.com

Co-authors:

Enyew Getaneh Mekonen **Email Address:** enyewgetaneh12@gmail.com

Bewuketu Terefe: **Email address**: woldeabwomariam@gmail.com

Mulugeta Wassie: **Email address:** mulugeta2113@gmail.com

Agazhe Aemro: **Email address:** agazhe1049@gmail.com

Alebachew Ferede Zegeye: **Email address:** alexferede24@gmail.com

Mohammed Seid Ali: **Email address:** muheseid2592@gmail.com

Berhan Tekeba: **Email address:** berishboss7@gmail.com

Tadesse Tarik Tamir: **Email address:** tadestar140@gmail.com

Belayneh Shetie Workneh: **Email address:** belay2319@gmail.com

Abstract

Background:

Barriers to accessing healthcare have significantly contributed to the high rates of maternal and child mortality and morbidity in developing regions, particularly in Sub-Saharan Africa. Access to healthcare is shaped by multiple factors, including financial constraints, geographic location, the availability of services, and the quality of care provided. This study aimed to assess the magnitude of these barriers and to identify the factors influencing healthcare access among women of reproductive age in Sub-Saharan Africa.

Methods:

This study utilized secondary data from the most recent Demographic and Health Surveys conducted between 2019 and 2023 across Sub-Saharan Africa. A weighted sample of 134,470 women of reproductive age was included in the analysis. Data were analyzed using Stata version 14, employing a multilevel mixed-effects logistic regression model. Factors associated with barriers to healthcare access were considered statistically significant at P-values less than 0.05. Adjusted odds ratios (AORs) with 95% confidence intervals (CIs) were used to interpret the results.

Result: In this study, the magnitude of barriers to accessing healthcare among reproductive age women were found to be 55.84% (95% CI: 55.57–56.10). Husband's educational status [AOR = 1.23, 95% CI: 1.15–1.30], Media exposure [AOR = 1.17, 95% CI: 1.11–1.24], antenatal care (ANC) visit status [AOR = 1.31, 95% CI: 1.10–1.55], and place of delivery [AOR = 1.33, 95% CI: 1.24–1.43]. Health insurance utilization [AOR = 1.18, 95% CI: 1.11–1.26], wealth index status, and visiting a health facility within the past 12 months [AOR = 0.91, 95% CI: 0.87–0.97] were individual associated variable. In addition, place of residence [AOR = 1.30, 95% CI: 1.22–1.39], community literacy level [AOR = 1.13, 95% CI: 1.03–1.24], community wealth index [AOR = 1.12, 95% CI: 1.02–1.22], and Region were associated with barriers to accessing healthcare.

Conclusion and recommendation: This study found that 55.55% of women in Sub-Saharan Africa experienced barriers to accessing healthcare. Educational status, media exposure, place of residence, antenatal care attendance, place of delivery, health insurance coverage, wealth index, recent visits to health facilities, community literacy levels, community wealth status, and regional differences were determinant factors. These findings highlight the need for Sub-Saharan African governments to prioritize these determinants when formulating policies and strategies aimed at reducing preventable barriers to healthcare access in the region.

Key words: Access to Healthcare; Barriers to Healthcare; Sub-Saharan Africa; Reproductive Age Women

Introduction

Access to healthcare is broadly defined as the ability to prevent illness, disability, and premature death, to promote and maintain health, and to attain health equity, particularly for women of reproductive age, by offering inclusive, high-quality care and the best possible availability, affordability, and accessibility of medical services[1-3]. Reproductive-age women's health is a critical global public health concern since it influences population health outcomes and fosters socioeconomic growth[4]. The advancement of socioeconomic development is significantly influenced by the health of women of reproductive age, especially in Africa. [5]. The Sustainable Development Goals (SDGs) place a strong emphasis on women's health, with Targets 3.7 and 3.8 of SDG 3 specifically focusing on achieving universal health coverage by 2030 and ensuring access to sexual and reproductive health services, including education and information on family planning[6, 7]. The Sustainable Development Goals (SDGs) explicitly target a substantial reduction in maternal mortality, with Goal 3.1 aiming to lower the global maternal mortality ratio to fewer than 70 per 100,000 live births by 2030. While these global efforts have contributed to some improvements in women's health outcomes, they have yet to fully address the root causes of maternal mortality. Achieving sustained and comprehensive progress remains a critical challenge. In 2016, Sub-Saharan Africa accounted for a disproportionately high share of the 303,000 maternal deaths reported globally, the majority of which were due to preventable causes. Moreover, non-communicable diseases continue to contribute significantly to female morbidity and mortality across nearly all countries, with a particularly severe impact in low- and middle-income settings, where many women are left with long-term disabilities [4-6]. Access to healthcare involves multiple dimensions, including financial affordability, geographic accessibility, availability of medical services, and the quality of care provided in health facilities. [8]. Access to healthcare is a key determinant of whether individuals utilize health services. Globally, approximately 400 million people lack essential healthcare, and around eight million die each year from preventable diseases—resulting in an estimated economic loss of $6 trillion in low- and middle-income countries. [6, 9, 10]. In 2015, world leaders pledged to achieve universal health coverage as part of the Sustainable Development Goals (SDGs), aiming to ensure access to affordable, high-quality medicines and financial protection for all [11, 12]. In 2017, the World Bank and WHO reported that half of the global population lacks access to essential health services. As a result, many countries in Sub-Saharan Africa have made universal healthcare a key part of their national health

strategies. However, there has been slow progress in turning this commitment into tangible results, particularly in securing national funding for equitable, high-quality healthcare, financial security, and overall health [6, 9, 13, 14]. Reproductive age women and child mortality from low family planning uptake, unsafe abortion practices, unintended pregnancies, and an increase in home births are all implications of women in reproductive age having trouble accessing health care[14]. Previous research has offered some evidence regarding the sociodemographic and economic factors that can be barriers to accessing healthcare, such as marital status, place of residence, maternal age, wealth index, educational attainment, and unemployment status. Nevertheless, women of reproductive age continue to face significant disparities in healthcare utilization and access[10, 14-16]. This study makes three unique contributions to the body of existing literature. In order to provide a current and policy-relevant analysis of the barrier to healthcare access, it first makes use of the most recent (2019–2023) Demographic and Health Survey (DHS) data. Second, our analysis takes a harmonized multi-country approach across ten sub-Saharan African nations, improving cross-national comparability and generalizability in contrast to many previous studies that are limited to single-country analyses or rely on older datasets. In order to gain a better understanding of the structural and contextual factors that influence healthcare access, the study employs a comprehensive multilevel analysis of determinants at the individual and community levels. These contributions put our research in a position to guide focused, empirically supported programs provide fair access to healthcare for reproductive age women in sub-Saharan. Therefore, this study aimed to ascertain the magnitude and potential contributing factors associated with barriers to accessing healthcare among women of reproductive age in sub-Saharan Africa.

Methods and materials

Patient and public involvement statement

Secondary data from the Demographic and Health Surveys (DHS) were used in this investigation.

Study design, study area, and period.

Data from ten Sub-Saharan African countries, collected between 2019 and 2023, were used to perform a multilevel mixed-effects analysis, based on the most recent Demographic and Health Surveys (DHS). The DHS collects community-based, cross-sectional data approximately every five years to generate updated health and health-related indicators.

Data source, study population and sampling technique

A secondary data analysis was conducted using Demographic and Health Survey (DHS) data collected between 2019 and 2023 from selected sub-Saharan African countries. The study specifically included DHS datasets from ten countries Burkina Faso, Gabon, Ghana, Kenya, Liberia, Rwanda, Sierra Leone, Tanzania, The Gambia, and Mozambique each of which had recent and publicly available survey data within the specified timeframe. The datasets were appended to assess the extent of, and factors underlying, barriers to healthcare access among women of reproductive age in sub-Saharan Africa. Each country's DHS comprises multiple datasets. The DHS employs a stratified, two-stage cluster sampling design: in the first stage, enumeration areas are selected, followed by systematic sampling of households within each selected area in the second stage.

The variable barrier to accessing health care were generated by computation of (v467b, v467c, v467d, v467f) from maternal recode (IR) data set was recoded. A binary logistic regression model was used to identify determinants a factor barrier to access the health care. Determinants of barrier to accessing the health care were reported in terms of an AOR with a significance level of (95%). In the univariate analysis, at 95% confidence intervals with a p-value of < 0.25 was considered for the candidate multivariable analysis of data. All variables with p values of <0.05 were considered statistically significant. Since DHS data are nationally representative, stratified multistage cluster sampling is required; therefor, sampling weights must be applied to produce unbiased estimates. To correctly weight DHS data, we use the sampling weights provided in the dataset, specifically the individual weight **(**v005**)** for women's individual recode files. This weight must be divided by 1,000,000 (v005/1000000). using "gen wgt=v005/1000000 Stata commands. A total weighted sample of 134,470 reproductive age women were included in the study **(Table 1).**

Table 1: The weighted Sample size for magnitude and determinants of barrier to accessing health care among reproductive age women in sub-Saharan Africa**.**

Country	Year of survey	weighted sample (n)	weighted sample (%)
Brukinefaso	2021	17,659	13.13
Gabon	2019-2021	6,507.15	4.83
Ghana	2022	15,014	11.16
Kenya	2022	16,716.36	12.43
Liberia	2019-2020	8,065	5.99
Rwanda	2019-2020	14,634	10.88
Sera lion	2019	15,574	11.58
Tanzania	2022	15,254	11.34
Gambia	2019-2020	11,865	8.08
Mozambique	2023	13,181.93	9.80
TOTAL		134,470	100%

Study variable

Outcome variables: The outcome of this study was barrier to access the health care. The Data in barrier of accessing the health care were collected among reproductive age women who used one of the health cares. Measurement of the outcome variables was determined by using computation of four variables; such as Distance to health facility (v467b), difficulty in obtaining money (v467c), getting permission go to the doctor (v467d) and not wanting to go alone (v467f). If a woman of reproductive age reported experiencing at least one of the following challenges, such as difficulty in obtaining money, distance to a health facility, lack of permission to seek treatment, or reluctance to go alone were considered to face significant barriers to healthcare access. These cases were coded as '1' and labeled as 'big problem.' Conversely, women who did not report any of these four barriers were considered to have no significant barriers to healthcare access. These were coded as '0' and labeled as 'no big problem'. This approach is consistent with prior literature using DHS data[17]

Independent Variable: Hence DHS data are hierarchical, independent factors come from two sources that was at individual and community levels factors were considered for this analysis. The individual-level independent variables were women's age (15-19, 20-35, 36-49), women educational status (No education, Primary education , Secondary and Higher education), women occupation (working, not working), Religion (Muslim, Christian , Others), Wealth index (Poor, Middle, media exposure (yes, No), internet utilization (yes, No), Residency (urban, rural), Husband educational status (No education, Primary education, secondary and higher education), Husband occupational status (No working, Working), Currently Contraceptive (No, Yes) Visiting health facility in 12 month (No, Yes), wanted pregnancy(yes, no) .

The community-level variables: Community poverty level (Low, High), Community literacy level (Low, High), Community media exposure, (Low, High), Place of residency (Urban, rural), Region (East, west) in sub Saharan Africa.

Data processing and statistical analysis

Stata version 14 statistical software was used to clean, record, and analyze secondary DHS data sets. The variables in the DHS data are grouped into clusters, and the variables within a cluster are more similar to each other than those in other clusters. The presumptions of equal variance

across clusters and independent data were violated in order to run a typical logistic regression model. This suggests that between-cluster factors must be considered using a complex model.

Given this, multilevel mixed-effects logistic regression was used to determine the factors that associated with barrier to accessing the health care. Multilevel mixed effect logistic regression follows four models: the null model (only outcome variable), mode I (individual level variables), model II (community level variables), and model III (both individual and community level variables). The null model without independent variables was used to check the variability of outcome variable across the cluster. The relationship of individual-level variables with outcome variable (Model I) and the association of community-level variables with outcome variable (Model II) were determined. In the final model, the association of both individual and community-level variables was fitted with the outcome variable.

Random effects

The intra-class correlation coefficient (ICC), proportionate change in variance (PCV), and median odds ratio (MOR) were used to quantify random effects or measures of variation of the outcome variables.

To assess the variation between clusters, the proportionate change in variance (PCV) and intra-cluster correlation coefficient (ICC) were calculated. By treating the cluster as a random variable, the ICC shows that the variation in the health care access barrier between clusters may be computed as follows; $ICC = \frac{VC}{VC+3.29} \times 100\%$. The MOR, which is determined by using clusters as a random variable and calculating the median value of the odds ratio between the highest-risk and lowest-risk areas for health care access barriers, is calculated; $MOR= e^{0.95\sqrt{VC}}$. Furthermore, the PCV calculates a variable that explains the variation in the degree of barriers to accessing healthcare ; $PCV = \frac{Vnull-VC}{Vnull} \times 100\%$;. where VC is the cluster level variance and Vnull is the variance of the null model.

The fixed effects were employed to calculate the correlation between the probability of barriers to obtaining health care access and independent factors at the individual and community levels. It was assessed and presented using adjusted odds ratio (AOR) and 95% confidence intervals with a p-value of < 0.05. Because of the nested nature of the model, deviation = -2(log likelihood ratio) was used to compare models, and the model with the lowest deviance was selected as the best-

fitted model. By evaluating the variance inflation factors, the model assumption for multi-collinearity was confirmed, and the results fell within an acceptable range of 1.03-1.98. In addition. The model including random intercepts and slopes (Model III) had the lowest AIC= (AIC=239608.6), indicating the best balance between model fit and parsimony.

Patient and public involvement

Since this study data was obtain from demographic and health survey (DHS) secondary source, no patient and public were involved.

Result

In this study a total of 134,470 reproductive age women were included in Sub-Saharan African. The Mean age of participants were 28.99 (Std. Dev. 9.68) years, and the Variance of 93.86557. More than two thirds (69.8) of women did not use contraception, and over 72.22% reproductive women did not use health insurance, and over half of (54.22%) women were living rural areas in sub-Saharan Africa. **(Table 2)**

Table 2: Sociodemographic and economic characteristics of reproductive women who had barriers to accessing health care in sub-Saharan Africa.

**Variable**	**frequency(n)**	**percentage (%)**
**Individual and community level variables**		
**Maternal age**		
15-19	27,890.4	20.7
20-35	69,441.4	51.64
36-49	37,138.6	27.64
**Residency**		
Urban	61,558.03	45.78
Rural	72,912.41	54.22
**Religious status**		
Muslim	48834.95	41.96
Christian	46856.90	39.30
Other	23,524.59	19.73
**Educational Status**		
No formal education	35,051.70	26.06
Primary education	39,651.17	29.48
Secondary and high	59,767.57	44.44
**Husband educational**		
No formal education	29,414.44	36.72

Primary education	21,245.78	26.52
secondary and above	29,428.78	36.74
**Marital Occupational**		
Not working	50,732.90	37.81
working	83,417	62.18
**Husband Occupation**		
Not working	10,390.43	13.0%
Working	69,698.11	87.%
**Marital status**		
single	42,213.40	31.4
Married	58,262.00	43.3
Other	33,995.4	25.3
**Contraceptive Utilization**		
No	93,855.00	69.8
Yes	40,615.5	30.2
**place of delivery**		
Home	8,279.46	15.5
Institution	44,979.28	84.5
ANC visit		
No visiting	2,012.84	3.77
1-4 visiting	24,459.34	45.92
>4 visiting	26,786.55	50.29
**Media exposure**		
No	34,628.45	25.75
Yes	99,831.57	74.24
**Intermate utilization**		
No	93,020.34	69.17
Yes	41,450.10	30.82
**Visiting health facility 12 month**		
No	57,137.73	42.49
Yes	77,332.71	57.50
**Health insurance**		
No	85,044.69	72.22
Yes	32,709.39	27.77
**Wealth index**		
poor	47,227.18	35.12
middle	25,771.45	19.16
High	61,471.81	45.74

**Region in sub-Saharan Africa**		
East Africa	59,786.29	44.46
West Africa	74,684.15	55.53
**Community poverty level**		
Low	70,776.71	52.63
High	63,693.73	47.47
**Community media exposure**		
Low	61,642.30	45.84
High	72,828.14	54.15
**Community distance health facility**		
Low	65,962	49
High	68,508	51
**Community literacy level**		
Low	57,615.01	42.84
High	76,855.43	57.15
**Community ANC visit**		
Low	77,912.32	57.96
High	56,491.92	42.03

In this study, the magnitude of barriers to accessing health care among reproductive age women was found to be 55.84 (95% CI: (55.57, 56.10) owing to one or more of the four factors, the most commonly cited being the difficulty in getting money and the distance from medical facilities (**Figure 1)**

Random effect (Measures of variation) and model fitness

To determine if the data supported the choice to evaluate randomness at the community level, a null model was employed. With a variance of.3784967 and a p value of 0.000, the null model's results showed that there were notable variations in the barriers to accessing healthcare among clusters. A significant amount of the variance in the outcome between clusters was jointly explained by both individual- and community-level variables, as evidenced by the intra-cluster correlation (ICC), which dropped from 10.31% in the null model to 3.96% in the fully adjusted model (model III). According to the proportionate change in variance (PCV), Model III explains the greatest percentage (64.09%) of the group-level variation in comparison to the null model, group comparison showed that, Model I (50.19%) and Model II (24.56%). This suggests that the model fits better as more predictors are included. Akaike information criteria **(AIC)** Among the

models evaluated, Model III demonstrated the best fit with the lowest AIC (239,608.6), indicating a substantial improvement over the null model (AIC = 1,128,437) and Model II (AIC = 1,127,704). Model I also showed good fit (AIC = 239,764.8), though slightly less optimal than Model III **(Table 3).**

Table 3: Model comparison and Fitness random effect analysis for barriers of accessing health care of among reproductive age women in sub-Saharan Africa**.**

**Parameter**	**Null model**	**model I**	**model II**	**model III**
Variance	.3784967	.1885269	0.28553	0.1358817
ICC	10.31%	5.41%	7.98%	3.96%
MOR	1.79	1.51	1.66	1.41
PCV	Reference	50.19%	24.56%	64.09%
**Model fines**				
LLR	-90175.365	-21798.27	-86841.704	-21433.284
Deviance	1803596.73	43596.54	173683.408	42866.568
AIC	1128437	239764.8	1127704	239608.6

ICC: intra-cluster correlation**, LLR:** loglikelihood ratio**, MOR:** median odds ratio**, PCV:** proportional change in variance. **AIC:** Akaike information criteria.

Determinants of individual and community level factors with barrios of accessing health care among reproductive age women in sub-Saharan Africa.

In the final fitted model of multivariable multilevel logistic regression, Husband educational status, media exposure, internet utilization, place of residency, ANC visit status, place of delivery, health insurance utilization status, wealth index status, visiting health facility within 12 months, and country category (western, eastern Africa) were significantly associated with barriers of accessing health care among reproductive age women in sub-Saharan Africa. The odds of barrier of accessing health care were 1.30 times higher among reproductive women living in rural residency compared to women living in urban area **(AOR=** 1.30, 95 CI: (1.22, 1.39). and the odds of barrier of accessing health care were 1.23 and 1.21 times more likely to among reproductive age women whose husband had no education and primary education compared to women whose husband had secondary and above educational level respectively **AOR**=1.23, 95 CI: (1.15, 1.30,) and (**AOR**=1.21, 95% CI: (1.31, 1.41). In addition, odds of barrier of accessing health care were 1.33 times more likely to occur women delivered at home compared to women who had institutional

delivery. (**AOR**=1.33, 95% CI: (1.24, 1.43) and the odds of barrier of accessing health care were 1.31- and 1.13-times higher women who had no history of antenatal visit and women who had < 4 ANC visit compared to women who had more than four ANC visit Respectively. (**AOR**= 1.31, 95% CI: (1.10,1.55) and (**AOR=** 1.13, 95% CI: (1.07,1.19) and the odds of the barrier of accessing health care among reproductive age woman who had no media exposure and intranet access were 1.17 and 1.68 times more likely to compared with a woman who had media exposure and internet access respectively (**AOR**=1.17, 95% CI: (1.11, 1.24) and (**AOR**= 1.68, 95% CI: (1.57, 1.79). and the barrier of accessing health care among reproductive age woman who were not visiting health facility within 12 months 9 % times less likely compared to women who had visiting health care facility within 12 months of study period (**AOR=**0.91, 95% (0.87, 0.97). The odds of perceived barrier of accessing health care among reproductive age woman who had no health insurance utilization were 1.18 times more likely to compared with a woman who had used health insurance (**AOR=**1.18 95% CI: (1.11, 1.26) and The odds of perceived barrier of accessing health care service among reproductive age woman who were classified under poor and middle wealth status were 2.29 and 1.57 times more likely accessing health care challenge compared to reproductive women who were classified under rich wealth status category(**AOR**=2.29 95% CI: (2.14, 2.45) and (**AOR**=1.57.95% CI: (1.46, 1.68). The odds of perceived barrier of accessing health care service among reproductive age woman who in west Africa region were 2.38 times more likely barriers accessing health care compared to east Africa region category (**AOR**=2.38, 95% CI: (2.21, 2.55) **(Table 4)**

Table 4: Multivariable multilevel logistic regression analysis of individual-level and community level variable associated with accessing health care among reproductive women in sub Saharan Africa.

Individual and community level factors	Model I AOR (95% CI)	Model II AOR (95% CI)	Model III AOR (95% CI)
**Maternal age**			
15-19	0.96(0.86, 1.07)		0.96 (0.86,1.08)
20-35	1.06(1.01,1.13)		1.04 (0.99, 1.11)
36-45	1		1
**Residency**			
Urban		1	1
Rural		2.29 (2.24, 2.35)	**1.30 (1.22, 1.39)**

**Maternal education**		
No education	1.10 (1.02, 1.18)	1.04 (0.97, 1.11)
Primary education	0.93 (0.87, 0.99)	1.06 (0.99, 1.14)
Secondary and above	1	1
**Husband education**		
No education	1.26 (1.19,1.35)	**1.23 (1.15, 1.30)**
Primary education	1.04(0.97,1.12)	1.23 (0.67, 1.22)
secondary education	1	1
**Marital occupation**		
working	1	1
not working	0.99 (0.94, 1.04)	**1.02 (1.01, 1.11)**
**Husband occupation**		
working	1	1
not working	0.90 (0.83, 0.97)	1.00 (0.92, 1.08)
**Religion**		
Muslim	1	1
Christian	1.62 (1.53, 1.71)	0.46 (0.57, 1.94)
Other	1.22 (1.13, 1.31)	1.36 (0.75, 1.46)
**Contraceptive utilization**		
No	1.11 (1.05,1.17	1.03 (0.98, 1.08)
Yes	1	1
**Place of delivery**		
home	1.30 (1.21, 1.40)	**1.33 (1.24, 1.43)**
Institution	1	**1**
**ANC visit**		
No	1.01 (0.85, 1.19)	**1.31 (1.10, 1.55)**
1-4 visit	0.94 (0.89, 0.99)	1.13 (0.37, 1.19)
> 4 visits	1	1
**Media exposure**		
No	1.14 (1.08, 1.21)	**1.17 (1.11, 1.24)**
Yes	1	1
**Internet utilization**		
No	1.69 (1.58, 1.80)	1.68 (0.67, 1.79)
Yes	1	1
**Visiting health facility 12 month**		
No	0.92 (0.87, 0.98)	**0.91 (0.87, 0.97)**
Yes	1	1
**Health insurance**		
No	1.26 (1.19, 1.34)	**1.18 (1.11, 1.26)**
Yes	1	1
**Wealth index**		
poor	2.80 (2.63, 2.97)	**2.29 (2.14, 2.45)**
middle	1.79 (1.68, 1.92)	**1.57 (1.46, 1.68)**
Rich	1	1

**Region SSA**			
East Africa		0.50 (0.49, 0.52)	**0.42 (0.39, 0.45)**
West Africa		1	1
**community ANC visit**			
Low		1.00 (0.93, 1.09)	0.93 (0.86, 1.00)
High		1	1
**community wealth index**			
Low		0.75 (0.70, 0.81)	**1.12 (1.02, 1.22)**
High		1	1
**Community media exposure**			
Low		1.01 (0.92, 1.10)	0.94 (0.86, 1.03)
High		1	1
**Community literacy level**			
Low		1.10(1.00, 1.20)	**1.13 (1.03, 1.24)**
High		1	**1**

Table 5: Key Individual and Community-Level Determinants of Barriers to Healthcare Access Among Women of Reproductive Age in Sub-Saharan Africa.

**Individual and community level factors**	**Model I AOR (95% CI)**	**Model II AOR (95% CI)**	**Model III AOR (95% CI)**
**Residency**			
Urban		1	1
Rural		2.29 (2.24, 2.35)	**1.30 (1.22, 1.39)**
**Husband education**			
No education	1.26 (1.19,1.35)		**1.23 (1.15, 1.30)**
Primary education	1.04(0.97,1.12)		1.23 (0.67, 1.22)
secondary education	1		1
**Marital occupation**			
working	1		1
not working	0.99 (0.94 1.04)		**1.02 (1.01, 1.11)**
**Place of delivery**			
home	1.30 (1.21, 1.40)		**1.33 (1.24,1.43)**
Institution	1		**1**
**ANC visit**			
No	1.01 (0.85, 1.19)		**1.31(1.10,1.55)**
1-4 visit	0.94 (0.89, 0.99)		1.13(0.37,1.19)
> 4 visits	1		1
**Media exposure**			
No	1.14 (1.08, 1.21)		**1.17 (1.11, 1.24)**
Yes	1		1

**Visiting health facility 12 month**			
No	0.92 (0.87, 0.98)		**0.91(0.87, 0.97)**
Yes	1		1
**Health insurance**			
No	1.26 (1.19, 1.34)		**1.18 (1.11,1.26)**
Yes	1		1
**Wealth index**			
poor	2.80 (2.63, 2.97)		**2.29 (2.14, 2.45)**
middle	1.79 (1.68, 1.92)		**1.57 (1.46, 1.68)**
Rich	1		1
**Region SSA**			
East Africa		0.50 (0.49, 0.52)	**0.42 (0.39, 0.45)**
West Africa		1	1
**community wealth index**			
Low		0.75 (0.70, 0.81)	**1.12 (1.02,1.22)**
High		1	1
**Community literacy level**			
Low		1.10(1.00, 1.20)	**1.13(1.03,1.24)**
High		1	**1**

Discussion

Barriers to accessing health care have contributed to maternal and child mortality and morbidity in developing countries such as those in Sub-Saharan Africa. This study aimed to assess the magnitude and determinants of barriers to health care access among women of reproductive age in Sub-Saharan Africa.

In this study, the magnitude of barriers to accessing health care among reproductive age women was found to be 55.84 (95% CI: (55.57, 56.10) owing to one or more of the four factors, the most commonly cited being the difficulty in getting money and the distance from medical facilities.

This finding is lower than the previous study conducted in South Africa 65%[18, 19], and Tanzania 65% [15], Ethiopia 71.8%,70.2, 69.9%[1, 20, 21], (SSA) 60.4%, 61.5% [17]. this variation could be due to the fact that socio-economic and variability in health infrastructure and health system policy, women's attitudes towards health care seeking behavior and cultural differences across countries in sub-Saharan Africa, moreover, this discrepancy might be due to study period, this study includes, the most recent DHS data set from 2019-2023. On the other hand, the finding of this study is similar to but slightly higher than in previous study in Gambia 45.5%[22], Ghana (51%)[23]. this variation could be due to the fact that socio-economic and variability in health infrastructure and health system policy, women's attitudes towards health care seeking behavior and cultural differences across countries in sub-Saharan Africa.

The odds of barrier of accessing health care were 1.30 times higher among reproductive women living in rural residency compared to women living in urban area. This finding is supported by previous studies conducted in Ethiopia [1, 20], guinea[24], Gambia, Tanzania and Ghana [15, 22, 25] respectively. Sub-Saharan[23], East Africa[26]. This association may be, due to the fact that rural areas have low health infrastructure i.e. inaccessibility of health facilities, less privileged and the influences of socio-cultural practices that need women to seek permission from their husband to visit the facility or women's decisions for seeking healthcare services is under their husband/partners control. Moreover, male involvement and support for women health care access are limited[1, 14, 23, 26]. This implies that the government should expand infrastructure services in rural area and encourage male involvement and support for improving health care access for reproductive age women[14].

The odds of perceived barrier of accessing health care were 1.23 and 1.21 times more likely to among reproductive age women whose husband had no education and primary education

compared to women whose husband had secondary and above educational level respectively. This find was consistent with previous study conducted in east Africa, Ethiopia, Tanzania, Benin, Bangladesh respectively [1, 15, 20, 26, 27]. The possible explanation could be in better access to the healthcare among women with educated husbands might be due to better participation and involvement of husbands in their families' health[23]. Women whose partners are educated are also likely to be informed regarding their fundamental human rights and may have higher health literacy. As a result, they are more likely to deal with any form of barrier to healthcare, compared to their counterparts who are less educated and may have lower health literacy [14, 15, 20, 28].

The odds of barrier to accessing health care were 1.33 times more likely to occur women delivered at home compared to women who had institutional delivery. This evidence was supported by study done in Senegal[29]. The plausible justification might be women who delivered at home may not get health information/counselling from healthcare providers that empower them to use other maternal healthcare services and women who deliver at home may face varous barriers to accessing health care such as financial constraint, lack of transportation, limited autonomy in healthcare decision and sociocultural norms that discourage institutional delivery. Moreover, importance of enhancing health system outreach, strengthen community-based education, and addressing sociocultural and socioeconomic disparities that affect health seeking behavior. On the other hand, the odds of barrier of accessing health care among woman who had no media exposure were 1.17 times more likely to compared with a woman who had media exposure. This evidence was supported by study done in Ethiopia[30], Nepal[31]. This could be the power of mass media in disseminating information concerning maternal health that may enhance women's knowledge and attitude towards health care access and utilization[31]. Barrier of accessing health care among woman who were not visiting health facility within 12 months were 9 % times less likely compared to women who had visiting health care facility within 12 months of study period. The odds of barrier to accessing health care among woman who had no health insurance utilization were 1.18 times more likely to compared with a woman who had used health insurance. This finding was supported with study done in Ethiopia[1], sub-Saharan Africa[23], Tanzania[15], Ghana[25]. The possible reason might be due to the fact that, individuals who were not had health insurance faced for unexpected catastrophic expenditure and difficulties in obtaining money for consultation of the doctor[1]. The odds of barrier to accessing healthcare among woman who were classified under poor and middle wealth status were 2.29 and 1.57 times more likely accessing health care difficulty

compared to women who were classified under rich wealth index category**.** This evidenced was supported with study done in Ethiopia, [1, 30, 32, 33] Tanzania[15] Uganda[34], sub Saharan Africa[14]. This finding might be explained by the fact that high out-of-pocket expenses and a lack of accessibility to healthcare facilities pose significant obstacles for middle-class and impoverished women in sub-Saharan Africa. Many people in the region cannot afford healthcare services because over 40% of health costs are paid for directly by individuals[35-38]

The odds of barrier of accessing health care among woman who live west Africa region were 2.38 times more likely barriers to accessing health care compared to east Africa region. According a recent joint report from the World Health Organization and the United Nations states that high out-of-pocket costs, a lack of adequate healthcare infrastructure, and other sociocultural barriers make it more difficult for women of reproductive age in West African nations such as Nigeria, Mali, and Sierra Leone to access maternal healthcare. On the other hand, East African countries like Rwanda, Kenya, and Ethiopia have strengthened their community health initiatives and gender-inclusive policies, which have improved women's autonomy in accessing healthcare services[39]. Furthermore, our current findings are supported by recent Demographic and Health Survey (DHS) program data (2022–2023), which indicate that women in West African countries are 2-3 times more likely to report delays in accessing healthcare due to cost, shortages of healthcare personnel, and long distances to health facilities. In contrast, East African countries, particularly Uganda and Tanzania, have strengthened their health insurance systems and rural outreach initiatives. Additionally, the World Bank's 2023 Health Service Coverage Index reveals that women in West Africa have significantly lower access to family planning, skilled birth attendance, and other essential reproductive health services[22, 40, 41]

Strength and limitation

This strength of study nationally representative data to evaluate contextual and individual characteristics related to barriers to healthcare access among reproductive age women in the SSA. Thus, findings can therefore, generalized to all women in their reproductive age in SSA, in order to account for the clusters within the sample, the study also employed advanced statistical models. This study has its own limitation, that is, the findings do not establish a cause and effect relationship due to the cross-sectional nature of the data/surveys and health-worker related factors could not be accounted for in this study.

Conclusion and Recommendation

This study found that 55.55% of reproductive age women had barriers to accessing healthcare in SSA. Husband educational status, media exposure, internet utilization, place of residency, ANC visit status, place of delivery, health insurance utilization status, wealth index status, visiting health facility within 12 months, community literacy level and community wealth index and country category (western, eastern Africa) were significantly associated with barriers of accessing health care. Therefore, the Government and ministry of health in Sub-Saharan Africa countries should taking in to consideration those factors while designing policies and strategies targeting to health care access and tackling the preventable barriers to accessing health care in sub-Saharan Africa.

Declaration

Ethical approval and consent to participate

This study was based on analysis of existing survey datasets in the public domain (SSA) that are freely available online with all the identifier information anonymized, no ethical approval was required

Consent for publication

Not applicable

Data and material availability

Demographic and Health Survey were used, and it is publicly available online at (https://www.dhsprogram.com).

Competing interest

Authors Declared that, there is no competing interest.

Funding

Not applicable

Author's contributions

GAZ: involved in designing, data extraction, data analysis, interpretation, report and manuscript writing.

BT: involved in data analysis, interpretation, and, and manuscript writing.

BT: involved in analysis,

TTT: involved in designing the study, interpretation, report and

BSW: involved in analysis, and result interpretation

AFZ: involved in designing and interpretation study

EGM: analysis and manuscript writing

MW: Result interpretation

AA: manuscript writing: involved in data extraction

MSA: involved manuscript writing

Acknowledgements

We would like to say the DHS programs for allowing us to use the relevant DHS dataset in this study.

Reference

1. Tamirat, K.S., Z.T. Tessema, and F.B.J.B.H.S.R. Kebede, Factors associated with the perceived barriers of health care access among reproductive-age women in Ethiopia: a secondary data analysis of 2016 Ethiopian demographic and health survey. 2020. **20**: p. 1-8.
2. Erasmus, M.O., The barriers to access for maternal health care amongst pregnant adolescents in the Mitchells plain sub-district. 2017.
3. Washington, D.L., et al., Access to care for women veterans: delayed healthcare and unmet need. 2011. **26**: p. 655-661.
4. Regan, L.J.B.M.A., Addressing unmet needs in global women's health. 2018.
5. Izugbara, C., E. Covan, and E. Fugate-Whitlock, Women's health in Africa: Issues, challenges and opportunities. 2017: Routledge.
6. Organization, W.H., Primary health care on the road to universal health coverage: 2019 global monitoring report: Executive summary. 2019, World Health Organization.
7. Badiuzzaman, M., S.M. Murshed, and M.J.T.J.o.D.S. Rieger, Improving maternal health care in a post conflict setting: evidence from Chittagong Hill tracts of Bangladesh. 2020. **56**(2): p. 384-400.

8. Peters, D.H., et al., Poverty and access to health care in developing countries. 2008. **1136**(1): p. 161-171.
9. Alkire, B.C., et al., The economic consequences of mortality amenable to high-quality health care in low-and middle-income countries. 2018. **37**(6): p. 988-996.
10. Nyakang'o, S.B. and A.J.M. Booth, Women's perceived barriers to giving birth in health facilities in rural Kenya: A qualitative evidence synthesis. 2018. **67**: p. 1-11.
11. Colglazier, W.J.S., Sustainable development agenda: 2030. 2015. **349**(6252): p. 1048-1050.
12. Cahill, C.M., et al., Preoperative anemia management program reduces blood transfusion in elective cardiac surgical patients, improving outcomes and decreasing hospital length of stay. 2021. **61**(9): p. 2629-2636.
13. Peeri, N.C., et al., The SARS, MERS and novel coronavirus (COVID-19) epidemics, the newest and biggest global health threats: what lessons have we learned? 2020. **49**(3): p. 717-726.
14. Tessema, Z.T., et al., Determinants of accessing healthcare in Sub-Saharan Africa: a mixed-effect analysis of recent Demographic and Health Surveys from 36 countries. 2022. **12**(1): p. e054397.
15. Bintabara, D., K. Nakamura, and K.J.B.o. Seino, Improving access to healthcare for women in Tanzania by addressing socioeconomic determinants and health insurance: a population-based cross-sectional survey. 2018. **8**(9): p. e023013.
16. Kea, A.Z., et al., Exploring barriers to the use of formal maternal health services and priority areas for action in Sidama zone, southern Ethiopia. 2018. **18**: p. 1-12.
17. Zegeye, B., et al., Breaking barriers to healthcare access: a multilevel analysis of individual-and community-level factors affecting women's access to healthcare services in Benin. 2021. **18**(2): p. 750.
18. Silal, S.P., et al., Exploring inequalities in access to and use of maternal health services in South Africa. 2012. **12**: p. 1-12.
19. Tsawe, M. and A.S.J.B.r.n. Susuman, Determinants of access to and use of maternal health care services in the Eastern Cape, South Africa: a quantitative and qualitative investigation. 2014. **7**: p. 1-10.

20. Zegeye, B., et al., Barriers and facilitators to accessing health care services among married women in Ethiopia: a multi-level analysis of the Ethiopia demographic and health survey. 2021.
21. Demsash, A.W., B. Chakilu, and A. Mazengia, Knowledge Sharing Practice and Its Associated Factors Among Healthcare Providers at University of Gondar Comprehensive Specialized Hospital, North West Ethiopian: Cross-sectional Study. 2021.
22. Tsegaw, M., B. Mulat, and K.J.B.o. Shitu, Problems with accessing healthcare and associated factors among reproductive-aged women in the Gambia using Gambia demographic and Health Survey 2019/2020: a cross-sectional study. 2023. **13**(8): p. e073491.
23. Seidu, A.-A.J.P.O., Mixed effects analysis of factors associated with barriers to accessing healthcare among women in sub-Saharan Africa: Insights from demographic and health surveys. 2020. **15**(11): p. e0241409.
24. Seidu, A.-A., et al., Individual and contextual factors associated with barriers to accessing healthcare among women in Papua New Guinea: insights from a nationwide demographic and health survey. 2021. **13**(6): p. 573-585.
25. Seidu, A.-A., et al., Barriers to accessing healthcare among women in Ghana: a multilevel modelling. 2020. **20**: p. 1-12.
26. Minyihun, A., Z.T.J.R.M. Tessema, and H. Policy, Determinants of access to health care among women in East African countries: a multilevel analysis of recent demographic and health surveys from 2008 to 2017. 2020: p. 1803-1813.
27. Sen, K.K., et al., Do education and living standard matter in breaking barriers to healthcare access among women in Bangladesh? 2023. **23**(1): p. 1431.
28. Dimbuene, Z.T., et al., Women's education and utilization of maternal health services in Africa: a multi-country and socioeconomic status analysis. 2018. **50**(6): p. 725-748.
29. Zegeye, B., et al., Predictors of institutional delivery service utilization among women of reproductive age in Senegal: a population-based study. 2021. **79**: p. 1-11.
30. Demsash, A.W., A.D.J.B.H. Walle, and C. Informatics, Women's health service access and associated factors in Ethiopia: application of geographical information system and multilevel analysis. 2023. **30**(1).

31. Tamang, T.M. Factors associated with completion of continuum of Care for Maternal Health in Nepal. in IUSSP XXVIII International Population Conference, Cape Town, South Africa. 2017.
32. Fentie, E.A., et al., Spatial distribution and determinants of barriers of health care access among female youths in Ethiopia, a mixed effect and spatial analysis. 2023. **13**(1): p. 21517.
33. Alamneh, T.S., et al., Barriers for health care access affects maternal continuum of care utilization in Ethiopia; spatial analysis and generalized estimating equation. 2022. **17**(4): p. e0266490.
34. Kalule-Sabiti, I., A.Y. Amoateng, and M.J.A.P.S. Ngake, The effect of socio-demographic factors on the utilization of maternal health care services in Uganda. 2014: p. 515-525.
35. Shiferaw, S., et al., Why do women prefer home births in Ethiopia? 2013. **13**: p. 1-10.
36. Yaya, S., G. Bishwajit, and V.J.B.g.h. Shah, Wealth, education and urban–rural inequality and maternal healthcare service usage in Malawi. 2016. **1**(2): p. e000085.
37. Voto, T.P., B.E. Voto, and N.J.E. Ngepah, The Impact of Out-of-Pocket Health Expenditure and Public Health Expenditure on Poverty in Sub-Saharan Africa. 2025. **13**(5): p. 134.
38. Alamneh, T.S., et al., Socioeconomic inequality in barriers for accessing health care among married reproductive aged women in sub-Saharan African countries: a decomposition analysis. 2022. **22**(1): p. 130.
39. AKINWALE, A.A.J.I.J.o.I.R. and A. Studies, Gender equity and social progress: empowering women and girls to drive sustainable development in Sub–Saharan Africa. 2023.
40. Arhin, K., E.F. Oteng-Abayie, and J.J.H.e.r. Novignon, Assessing the efficiency of health systems in achieving the universal health coverage goal: evidence from Sub-Saharan Africa. 2023. **13**(1): p. 25.
41. Muhajarine, N., et al., Inequalities and factors associated with maternal healthcare services utilisation in Mozambique: evidence from the Demographic and Health Survey 2022−2023. 2025. **10**(5).

Figure legend

Fig 1: Barriers to accessing health care and determinant factor among reproductive age women in sub-Saharan Africa

Manuscript Click here to access/download;Manuscript;Barrier to HCA LAST Rivised.docx

Title: Barriers to accessing health care among women's in Sub-Saharan Africa: Multilevel Analysis of Recent Demographic and Health Survey from 2019-2023.

Gebreeyesus Abera Zeleke[1]*, Enyew Getaneh Mekonen[1], Bewuketu Terefe[2], Mulugeta Wassie[3], Agazhe Aemro[3], Alebachew Ferede Zegeye[3], Mohammed Seid Ali[4], Berhan Tekeba[4], Tadesse Tarik Tamir[4], Belayneh Shetie Workneh[5]

[1] Department of Surgical Nursing, School of Nursing, College of Medicine and Health Sciences, University of Gondar, Gondar, Ethiopia

[2] Department of Community Health Nursing, School of Nursing, College of Medicine and Health Sciences, University of Gondar, Gondar, Ethiopia.

[3] Department of Medical Nursing, School of Nursing, College of Medicine and Health Sciences, University of Gondar, Gondar, Ethiopia;

[4] Department of Pediatrics and Child Health Nursing, School of Nursing, College of Medicine and Health Sciences, University of Gondar, Gondar, Ethiopia.

5 Department of Emergency and Critical Care Nursing, School of Nursing, College of Medicine and Health Sciences, University of Gondar, Gondar, Ethiopia

Correspondence Author*
Gebreeyesus Abera Zeleke

Department of Surgical Nursing, School of Nursing, College of Medicine and Health Science, University of Gondar, Gondar, Ethiopia.

Email address: gebreeyesusabe143@gmail.com

Co-authors:

Enyew Getaneh Mekonen **Email Address:** enyewgetaneh12@gmail.com

Bewuketu Terefe: **Email address**: woldeabwomariam@gmail.com

Mulugeta Wassie: **Email address:** mulugeta2113@gmail.com

Agazhe Aemro: **Email address:** agazhe1049@gmail.com

Alebachew Ferede Zegeye: **Email address:** alexferede24@gmail.com

Mohammed Seid Ali: **Email address:** muheseid2592@gmail.com

Berhan Tekeba: **Email address:** berishboss7@gmail.com

Tadesse Tarik Tamir: **Email address:** tadestar140@gmail.com
Belayneh Shetie Workneh: **Email address:** belay2319@gmail.com

Abstract

Background:

Barriers to accessing healthcare have significantly contributed to the high rates of maternal and child mortality and morbidity in developing regions, particularly in Sub-Saharan Africa. Access to healthcare is shaped by multiple factors, including financial constraints, geographic location, the availability of services, and the quality of care provided. This study aimed to assess the magnitude of these barriers and to identify the factors influencing healthcare access among women of reproductive age in Sub-Saharan Africa.

Methods:

This study utilized secondary data from the most recent Demographic and Health Surveys conducted between 2019 and 2023 across Sub-Saharan Africa. A weighted sample of 134,470 women of reproductive age was included in the analysis. Data were analyzed using Stata version 14, employing a multilevel mixed-effects logistic regression model. Factors associated with barriers to healthcare access were considered statistically significant at P-values less than 0.05. Adjusted odds ratios (AORs) with 95% confidence intervals (CIs) were used to interpret the results.

Result: In this study, the magnitude of barriers to accessing healthcare among reproductive age women were found to be 55.84% (95% CI: 55.57–56.10). Husband's educational status [AOR = 1.23, 95% CI: 1.15–1.30], Media exposure [AOR = 1.17, 95% CI: 1.11–1.24], Antenatal care visit [AOR = 1.31, 95% CI: 1.10–1.55], and place of delivery [AOR = 1.33, 95% CI: 1.24–1.43]. Health insurance utilization [AOR = 1.18, 95% CI: 1.11–1.26], Wealth index, 2.29 [2.14, 2.45], 1.57 [1.46, 1.68] and visiting a health facility within the past 12 months [AOR = 0.91, 95% CI: 0.87–0.97] were individual associated variable. In addition, place of residence [AOR = 1.30, 95% CI: 1.22–1.39], community literacy level [AOR = 1.13, 95% CI: 1.03–1.24], community wealth index [AOR = 1.12, 95% CI: 1.02–1.22], and Region were associated with barriers to accessing healthcare.

Conclusion and recommendation: This study found that 55.55% of women in Sub-Saharan Africa experienced barriers to accessing healthcare. Educational status, media exposure, place of residence, antenatal care attendance, place of delivery, health insurance coverage, wealth index, recent visits to health facilities, community literacy levels, community wealth status, and regional differences were determinant factors. These findings highlight the need for Sub-Saharan African governments to prioritize these determinants when formulating policies and strategies aimed at reducing preventable barriers to healthcare access in the region.

Key words: Access to Healthcare; Barriers to Healthcare; Sub-Saharan Africa; Reproductive Age Women

Introduction

Access to healthcare is broadly defined as the ability to prevent illness, disability, and premature death, to promote and maintain health, and to attain health equity, particularly for women of reproductive age, by offering inclusive, high-quality care and the best possible availability, affordability, and accessibility of medical services[1-3]. Reproductive-age women's health is a critical global public health concern since it influences population health outcomes and fosters socioeconomic growth[4]. The advancement of socioeconomic development is significantly influenced by the health of women of reproductive age, especially in Africa. [5]. The Sustainable Development Goals (SDGs) place a strong emphasis on women's health, with Targets 3.7 and 3.8 of SDG 3 specifically focusing on achieving universal health coverage by 2030 and ensuring access to sexual and reproductive health services, including education and information on family planning[6, 7]. The Sustainable Development Goals (SDGs) explicitly target a substantial reduction in maternal mortality, with Goal 3.1 aiming to lower the global maternal mortality ratio to fewer than 70 per 100,000 live births by 2030. While these global efforts have contributed to some improvements in women's health outcomes, they have yet to fully address the root causes of maternal mortality. Achieving sustained and comprehensive progress remains a critical challenge. In 2016, Sub-Saharan Africa accounted for a disproportionately high share of the 303,000 maternal deaths reported globally, the majority of which were due to preventable causes. Moreover, non-communicable diseases continue to contribute significantly to female morbidity and mortality across nearly all countries, with a particularly severe impact in low- and middle-income settings, where many women are left with long-term disabilities [4-6]. Access to healthcare involves multiple dimensions, including financial affordability, geographic accessibility, availability of medical services, and the quality of care provided in health facilities. [8]. Access to healthcare is a key determinant of whether individuals utilize health services. Globally, approximately 400 million people lack essential healthcare, and around eight million die each year from preventable diseases—resulting in an estimated economic loss of $6 trillion in low- and middle-income countries. [6, 9, 10]. In 2015, world leaders pledged to achieve universal health coverage as part of the Sustainable Development Goals (SDGs), aiming to ensure access to affordable, high-quality medicines and financial protection for all [11, 12]. In 2017, the World Bank and WHO reported that half of the global population lacks access to essential health services. As a result, many countries in Sub-Saharan Africa have made universal healthcare a key part of their national health

strategies. However, there has been slow progress in turning this commitment into tangible results, particularly in securing national funding for equitable, high-quality healthcare, financial security, and overall health [6, 9, 13, 14]. Reproductive age women and child mortality from low family planning uptake, unsafe abortion practices, unintended pregnancies, and an increase in home births are all implications of women in reproductive age having trouble accessing health care[14]. Previous research has offered some evidence regarding the sociodemographic and economic factors that can be barriers to accessing healthcare, such as marital status, place of residence, maternal age, wealth index, educational attainment, and unemployment status. Nevertheless, women of reproductive age continue to face significant disparities in healthcare utilization and access[10, 14-16]. This study makes three unique contributions to the body of existing literature. In order to provide a current and policy-relevant analysis of the barrier to healthcare access, it first makes use of the most recent (2019–2023) Demographic and Health Survey (DHS) data. Second, our analysis takes a harmonized multi-country approach across ten sub-Saharan African nations, improving cross-national comparability and generalizability in contrast to many previous studies that are limited to single-country analyses or rely on older datasets. In order to gain a better understanding of the structural and contextual factors that influence healthcare access, the study employs a comprehensive multilevel analysis of determinants at the individual and community levels. These contributions put our research in a position to guide focused, empirically supported programs provide fair access to healthcare for reproductive age women in sub-Saharan. Therefore, this study aimed to ascertain the magnitude and potential contributing factors associated with barriers to accessing healthcare among women of reproductive age in sub-Saharan Africa.

Methods and materials

Patient and public involvement statement

Secondary data from the Demographic and Health Surveys (DHS) were used in this investigation.

Study design, study area, and period.

Data from ten Sub-Saharan African countries, collected between 2019 and 2023, were used to perform a multilevel mixed-effects analysis, based on the most recent Demographic and Health Surveys (DHS). The DHS collects community-based, cross-sectional data approximately every five years to generate updated health and health-related indicators.

Data source, study population and sampling technique

A secondary data analysis was conducted using Demographic and Health Survey (DHS) data collected between 2019 and 2023 from selected sub-Saharan African countries. The study specifically included DHS datasets from ten countries Burkina Faso, Gabon, Ghana, Kenya, Liberia, Rwanda, Sierra Leone, Tanzania, The Gambia, and Mozambique each of which had recent and publicly available survey data within the specified timeframe. The datasets were appended to assess the extent of, and factors underlying, barriers to healthcare access among women of reproductive age in sub-Saharan Africa. Each country's DHS comprises multiple datasets. The DHS employs a stratified, two-stage cluster sampling design: in the first stage, enumeration areas are selected, followed by systematic sampling of households within each selected area in the second stage.

The variable barrier to accessing health care were generated by computation of (v467b, v467c, v467d, v467f) from maternal recode (IR) data set was recoded. A binary logistic regression model was used to identify determinants a factor barrier to access the health care. Determinants of barrier to accessing the health care were reported in terms of an AOR with a significance level of (95%). In the univariate analysis, at 95% confidence intervals with a p-value of < 0.25 was considered for the candidate multivariable analysis of data. All variables with p values of <0.05 were considered statistically significant. Since DHS data are nationally representative, stratified multistage cluster sampling is required; therefor, sampling weights must be applied to produce unbiased estimates. To correctly weight DHS data, we use the sampling weights provided in the dataset, specifically the individual weight **(**v005**)** for women's individual recode files. This weight must be divided by 1,000,000 (v005/1000000). using "gen wgt=v005/1000000 Stata commands. A total weighted sample of 134,470 reproductive age women were included in the study **(Table 1).**

Table 1: The weighted Sample size for magnitude and determinants of barrier to accessing health care among reproductive age women in sub-Saharan Africa**.**

Country	Year of survey	weighted sample (n)	weighted sample (%)
Brukinefaso	2021	17659	13.13
Gabon	2019-2021	6507.15	4.83
Ghana	2022	15014	11.16
Kenya	2022	16716.36	12.43
Liberia	2019-2020	8065	5.99
Rwanda	2019-2020	14634	10.88
Sera lion	2019	15574	11.58
Tanzania	2022	15254	11.34
Gambia	2019-2020	11865	8.08
Mozambique	2023	13181.93	9.80
TOTAL		134470	100%

Study variable

Outcome variables: The outcome of this study was barrier to access the health care. The Data in barrier of accessing the health care were collected among reproductive age women who used one of the health cares. Measurement of the outcome variables was determined by using computation of four variables; such as Distance to health facility (v467b), difficulty in obtaining money (v467c), getting permission go to the doctor (v467d) and not wanting to go alone (v467f). If a woman of reproductive age reported experiencing at least one of the following challenges, such as difficulty in obtaining money, distance to a health facility, lack of permission to seek treatment, or reluctance to go alone were considered to face significant barriers to healthcare access. These cases were coded as '1' and labeled as 'big problem.' Conversely, women who did not report any of these four barriers were considered to have no significant barriers to healthcare access. These were coded as '0' and labeled as 'no big problem'. This approach is consistent with prior literature using DHS data[17]

Independent Variable: Hence DHS data are hierarchical, independent factors come from two sources that was at individual and community levels factors were considered for this analysis. The individual-level independent variables were women's age (15-19, 20-35, 36-49), Women educational status (No education, Primary education , Secondary & Higher education), women occupation (working, not working), Religion (Muslim, Christian , Others), Wealth index (Poor, Middle, Rich) media exposure (yes, No), internet utilization (yes, No), Residency (urban, rural), Husband educational status (No education, Primary education, secondary& higher education), Husband occupational status (No working, Working), Currently Contraceptive (No, Yes) Visiting health facility in 12 month (No, Yes), place of delivery (Home , Institutional delivery), ANC visit (No antenatal visit, 1-4 visit, > 4 visit)

The community-level variables: Poverty level (Low, High), Literacy level (Low, High), Media exposure, (Low, High), Residency (Urban, rural), Region (East, west), Community ANC visit

Data processing and statistical analysis

Stata version 14 statistical software was used to clean, record, and analyze secondary DHS data sets. The variables in the DHS data are grouped into clusters, and the variables within a cluster are more similar to each other than those in other clusters. The presumptions of equal variance

across clusters and independent data were violated in order to run a typical logistic regression model. This suggests that between-cluster factors must be considered using a complex model.

Given this, multilevel mixed-effects logistic regression was used to determine the factors that associated with barrier to accessing the health care. Multilevel mixed effect logistic regression follows four models. The null model (only outcome variable), mode I (individual level variable such as age, Religious status**,** educational status, Husband educational, marital occupation**,** Husband occupation**,** marital status**,** Contraceptive Utilization, place of delivery, ANC visit, media exposure, Intermate utilization**,** visiting health facility within 12 months, health insurance , wealth index) model II community level variables, Region in sub-Saharan Africa**,** community poverty level, community media exposure, Community distance health facility, Community literacy level, Community ANC visit), and model III (both individual and community level variables such as age, Religious status**,** educational status, Husband educational, marital occupation**,** Husband Occupation**,** marital status**,** Contraceptive Utilization, place pf delivery, ANC visit, media exposure, Intermate utilization**,** visiting health facility within 12 months, health insurance , wealth index), model II (community level variables, Region in sub-Saharan Africa**,** community poverty level, community media exposure, Community distance health facility, Community literacy level, Community ANC visit). The null model without independent variables was used to check the variability of outcome variable across the cluster. The relationship of individual-level variables with outcome variable (Model I) and the association of community-level variables with outcome variable (Model II) were determined. In the final model, the association of both individual and community-level variables was fitted with the outcome variable.

Random effects

The intra-class correlation coefficient (ICC), proportionate change in variance (PCV), and median odds ratio (MOR) were used to quantify random effects or measures of variation of the outcome variables.

To assess the variation between clusters, the proportionate change in variance (PCV) and intra-cluster correlation coefficient (ICC) were calculated. By treating the cluster as a random variable, the ICC shows that the variation in the health care access barrier between clusters may be computed as follow $\boldsymbol{ICC} = \frac{\sigma^2 uo}{\sigma^2 uo + \pi^2/3} \times \mathbf{100\%}$**.** The MOR, which is determined by using clusters as a

random variable and calculating the median value of the odds ratio between the highest-risk and lowest-risk areas for health care access barriers, is calculated. $MOR = \exp(0.6745\ \sqrt{(2 * \sigma^2 uo)})$ Furthermore, the PCV calculates a variable that explains the variation in the degree of barriers to accessing healthcare ; $PCV = \frac{\sigma^2 uo - \sigma^2}{\sigma^2 uo} \times 100\%$;. where VC is the cluster level variance and Vnull is the variance of the null model.

The associations between independent factors at the individual and community levels and barriers to accessing health care were estimated using the fixed effects. It was assessed and presented using adjusted odds ratio (AOR) and 95% confidence intervals with a p-value of < 0.05. Because of the nested nature of the model, deviation = -2(log likelihood ratio) was used to compare models, and the model with the lowest deviance was selected as the best-fitted model. By evaluating the variance inflation factors, the model assumption for multi-collinearity was confirmed. In addition, Model comparison was performed using Akaike's Information Criterion (AIC). The model includes both random intercepts and random slopes (Model III) was evaluated alongside alternative models, with AIC values calculated to assess relative model fit and parsimony.

Ethical approval and consent to participate

This study was based on analysis of existing survey datasets in the public domain (SSA) that are freely available online with all the identifier information anonymized, no ethical approval was required

Result

In this study a total of 134,470 reproductive age women were included in Sub-Saharan African. The Mean age of participants were 28.99 (Std. Dev. 9.68) years. More than two thirds (69.8%) of women did not use contraception, and over 72.22% reproductive women did not use health insurance, and over half of (54.22%) women were living rural areas in sub-Saharan Africa **(Table 2)**

Table 2: Sociodemographic and economic characteristics of reproductive women who had barriers to accessing health care in sub-Saharan Africa.

**Variable**	**frequency(n)**	**percentage (%)**
**Individual and community level variables**		

**Maternal age**		
15-19	27890.4	20.7
20-35	69441.4	51.64
36-49	37138.6	27.64
**Residency**		
Urban	61558.03	45.78
Rural	72912.41	54.22
**Religious status**		
Muslim	48834.95	41.96
Christian	46856.90	39.30
Other	23524.59	19.73
**Educational Status**		
No formal education	35051.70	26.06
Primary education	39651.17	29.48
Secondary and higher education	59767.57	44.44
**Husband educational**		
No formal education	29414.44	36.72
Primary education	21245.78	26.52
secondary and higher education	29428.78	36.74
**Maternal Occupational**		
Not working	50732.90	37.81
working	83417.01	62.19
**Husband Occupation**		
Not working	10390.43	13.00
Working	69698.11	87.00
**Maternal status**		
single	42213.40	31.4
Married	58262.00	43.3
Other	33995.4	25.3
**Contraceptive Utilization**		
No	93855.00	69.8
Yes	40615.5	30.2
**place of delivery**		
Home	8279.46	15.5
Institution	44979.28	84.5
ANC visit		
No visiting	2012.84	3.77
1-4 visiting	24459.34	45.92
>4 visiting	26786.55	50.29

Media exposure

No	34628.45	25.75
Yes	99831.57	74.24
**Intermate utilization**		
No	93020.34	69.17
Yes	41450.10	30.82
**Visiting health facility 12 month**		
No	57137.73	42.49
Yes	77332.71	57.50
**Health insurance**		
No	85044.69	72.22
Yes	32709.39	27.77
**Wealth index**		
poor	47227.18	35.12
middle	25771.45	19.16
Rich	61471.81	45.74
**Region in sub-Saharan Africa**		
East Africa	59786.29	44.46
West Africa	74684.16	55.54
**Community poverty level**		
Low	70776.71	52.63
High	63693.73	47.47
**Community media exposure**		
Low	61642.30	45.84
High	72828.14	54.15
**Community distance health facility**		
Low	65962	49.00
High	68508	51.00
**Community literacy level**		
Low	57615.01	42.84
High	76855.43	57.15
**Community ANC visit**		
Low	77912.32	57.96
High	56491.92	42.03

In this study, the magnitude of barriers to accessing health care among reproductive age women was found to be 55.84 (95% CI: (55.57, 56.10) owing to one or more of the four factors, the most commonly cited being the difficulty in getting money and the distance from medical facilities (**Figure 1)**

Random effect (Measures of variation) and model fitness

To determine if the data supported the choice to evaluate randomness at the community level, a null model was employed. With the variance of 0.3784967 and a p-value of < 0.001, the null model's results showed that there were notable variations in the barriers to accessing healthcare among clusters. A significant amount of the variance in the outcome between clusters was jointly explained by both individual- and community-level variables, as evidenced by the intra-cluster correlation (ICC), which dropped from 10.31% in the null model to 3.96% in the fully adjusted model (model III). According to the proportionate change in variance (PCV), Model III explains the greatest percentage (64.09%) of the group-level variation in comparison to the null model, group comparison showed that, Model I (50.19%) and Model II (24.56%). This suggests that the model fits better as more predictors are included. Model comparison using Akaike's Information Criterion (AIC) indicated that Model III achieved the best fit, as evidenced by the lowest AIC (239,608.6), markedly lower than both the null model (AIC = 1,128,437) and Model II (AIC = 1,127,704). Model I also showed good fit (AIC = 239,764.8), though slightly less optimal than Model III. Multicollinearity using variance inflation factors (VIF) indicated that all VIF values ranged from 1.03-1.98, which is within accepted range **(Table 3)**

Table 3: Model comparison and Fitness random effect analysis for barriers of accessing health care of among reproductive age women in sub-Saharan Africa**.**

**Parameter**	**Null model**	**model I**	**model II**	**model III**
Variance	0.3784967	0.1885269	0.28553	0.1358817
ICC	10.31%	5.41%	7.98%	3.96%
MOR	1.79	1.51	1.66	1.41
PCV	Reference	50.19%	24.56%	64.09%
**Model fines**				
LLR	-90175.365	-21798.27	-86841.704	-21433.284
Deviance	1803596.73	43596.54	173683.408	42866.568
AIC	1128437	239764.8	1127704	239608.6

ICC: intra-cluster correlation**, LLR:** loglikelihood ratio**, MOR:** median odds ratio**, PCV:** proportional change in variance. **AIC:** Akaike information criteria.

Determinants of barriers to accessing health care among reproductive age women in Sub-Saharan Africa

Bivariable logistic regression analysis of individual and community level variables associated with barriers to accessing health care among reproductive age women in Sub-Saharan Africa

Bivariable multilevel logistic regression analysis indicated that husband's educational status, media exposure, internet utilization, place of residence, antenatal care attendance, place of delivery, health insurance coverage, community wealth index, recent health facility visits, and country category (Western vs. Eastern Africa) were associated with barriers to accessing health care among reproductive-age women at a p-value < 0.25 (**Table 4**)

Table 4: Bivariable logistic regression of individual and community level factors associated with barriers to accessing health care among reproductive age women in Sub-Saharan Africa.

Variables	Crude Odds Ratio [COR]	P. value	[95% CI]
**Maternal age**			
15-19	0.86	<0.001	[0.83, 0.89]
20-35	0.86	<0.001	[0.84, 0. 88]
36-49	1		1
**Residency**			
Urban	1		1
Rural	2.09	<0.001	[2.04, 2.14]
**Maternal education**			
No education	2.40	<0.001	[2.33, 2.46]
Primary education	1.52	<0.001	[1.48, 1.56]
Secondary & Higher	1		1
**Husband education**			
No education	2.43	<0.001	[2.35, 2.51]
Primary education	1.55	<0.001	[1.49, 1.60]
secondary & higher	1		1
**Maternal occupation**			
working	1		1
not working	0.94	<0.001	[0.92, 0.96]
**Husband occupation**			
working	1		
not working	1.07	<0.001	[1.02, 1.12]
**Religion**			
Muslim	1		1
Christian	1.32	<0.001	[1.29, 1.35]
Other	1.08	<0.001	[1.04, 1.11]

**Contraceptive utilization**			
No	1.18	<0.001	[0.82, 0.86]
Yes	1		1
**Place of delivery**			
Home	1.84	<0.001	[1.75, 1.93]
Institution	1		1
**ANC visit**			
No visit	1.16	<0.002	[1.05, 1.27]
1-4 visit	1.05	<0.003	[1.01, 1.09]
> 4 visits	1		1
**Marital status**			
single	1		
Married	1.23	<0.001	[1.20, 1.26]
Other	1.32	<0.001	[1.29, 1.36]
**Media exposure**			
No	1.90	<0.001	[1.85, 1.95]
Yes	1		1
**Internet utilization**			
No	2.321822	<0.001	[2.26, 2.37]
Yes	1		1
**Visiting health facility 12 month**			
No	1.02	<0.046	[1.00, 1.04]
Yes	1		1
**Health insurance**			
No	1.21	<0.001	[1.18, 1.25]
Yes	1		1
**Wealth index**			
poor	3.58	<0.001	[3.49, 3.67]
middle	2.08	<0.001	[2.02, 2.14]
Rich	1		1
**Region SSA**			
East Africa	1.68	<0.001	[1.65, 1.72]
West Africa	1		1
**community ANC visit**			
Low	0.86	<0.001	[0.85, 0.88]
High	1		1
**community wealth index**			
Low	0.69	<0.001	[0.67, 0.70]
High	1		
**Community media exposure**			
Low	0.73	<0.001	[0.72, 0.75]
High	1		1
**Community literacy level**			
Low	0.77	<0.001	[0.75, 0.79]
High	1		1

Multivariable multilevel logistic regression analysis of individual and community level factors associated with Barriers to health care access among reproductive Age Women in Sub-Saharan Africa.

In the final fitted model of multivariable multilevel logistic regression, Husband educational status, media exposure, place of residency, Antenatal care visit, place of delivery, health insurance utilization, wealth index status, visiting health facility within 12 months, and country category (western, eastern Africa) were significantly associated with barriers of accessing health care among reproductive age women in sub-Saharan Africa. The odds of barrier of accessing health care were 1.30 times higher among reproductive women living in rural residency compared to women living in urban area **[AOR=**1.30, 95 CI: (1.22, 1.39)]. and the odds of barrier of accessing health care were 1.23 times more likely to among reproductive age women whose husband had no education compared to women whose husband had secondary and higher education [**AOR**=1.23, 95 CI: (1.15, 1.30,)]. In addition, odds of barrier of accessing health care were 1.33 times more likely to occur women delivered at home compared to women who had institutional delivery. **(AOR**=1.33, 95% CI: (1.24, 1.43) and the odds of barrier of accessing health care were 1.31 times higher among women who had no history of antenatal visit compared to women who had more than four ANC visit. [**AOR**= 1.31, 95% CI: (1.10, 1.55)]. The odds of the barrier of accessing health care among reproductive age woman who had no media exposure were 1.17 times more likely to compared with a woman who had media exposure **[AOR**=1.17, 95% CI: (1.11, 1.24)] and the barrier of accessing health care among reproductive age woman who were not visiting health facility within 12 months 9% times less likely compared to women who had visiting health care facility within 12 months of study period **[AOR=**0.91, 95% CI: (0.87, 0.97)]. The odds of perceived barrier of accessing health care among reproductive age woman who had no health insurance utilization were 1.18 times more likely to compared with a woman who had used health insurance [**AOR=**1.18 95% CI: (1.11, 1.26)] and The odds of perceived barrier of accessing health care service among reproductive age woman who were classified under poor and middle wealth status were 2.29 and 1.57 times more likely barries accessing health care compared to reproductive women who were classified under rich wealth status category **[AOR**=2.29 95% CI: (2.14, 2.45)] and [**AOR**=1.57, 95% CI: (1.46, 1.68)]. The odds of perceived barrier of accessing health care service among reproductive age woman who reside in west Africa region were 2.38 times more

likely barriers accessing health care compared to east Africa region category. [**AOR**=2.38, 95% CI: (2.21, 2.55)] **(Table 5).**

Table 5: Multivariable multilevel logistic regression of individual and community level factors associated with barriers to accessing health care among reproductive age women in Sub-Saharan Africa.

Individual and community level factors	Model I AOR [95% CI]	Model II AOR [95% CI]	Model III AOR [95% CI]
**Maternal age**			
15-19	0.96 [0.86, 1.07]		0.96 [0.86,1.08]
20-35	1.06 [1.01,1.13]		1.04 [0.99, 1.11]
36-45	1		1
**Residency**			
Urban		1	1
Rural		2.29 [2.24, 2.35]	**1.30 [1.22, 1.39]**
**Maternal education**			
No education	1.10 [1.02, 1.18]		1.04 [0.97, 1.11]
Primary education	0.93 [0.87, 0.99]		1.06 [0.99, 1.14]
Secondary and above	1		1
**Husband education**			
No education	1.26 [1.19,1.35]		**1.23 [1.15, 1.30]**
Primary education	1.04 [0.97,1.12]		1.23 [0.67, 1.22]
secondary & higher education	1		1
**Maternal occupation**			
working	1		1
not working	0.99 [0.94, 1.04]		**1.02 [1.01, 1.11]**
**Husband occupation**			
working	1		1
not working	0.90 [0.83, 0.97]		1.00 [0.92, 1.08]
**Religion**			
Muslim	1		1
Christian	1.62 [1.53, 1.71]		0.46 [0.57, 1.94]
Other	1.22 [1.13, 1.31]		1.36 [0.75, 1.46]
**Contraceptive utilization**			
No	1.11 [1.05,1.17]		1.03 [0.98, 1.08]
Yes	1		1
**Place of delivery**			
home	1.30 [1.21, 1.40]		**1.33 [1.24, 1.43]**
Institution	1		**1**

**Antenatal Care Visit**			
No	1.01 [0.85, 1.19]		**1.31 [1.10, 1.55]**
1-4 visit	0.94 [0.89, 0.99]		1.13 [0.37, 1.19]
> 4 visits	1		1
**Media exposure**			
No	1.14 [1.08, 1.21]		**1.17 [1.11, 1.24]**
Yes	1		1
**Internet utilization**			
No	1.69 [1.58, 1.80]		1.68 [0.67, 1.79]
Yes	1		1
**Visiting health facility 12 month**			
No	0.92 [0.87, 0.98]		**0.91 [0.87, 0.97]**
Yes	1		1
**Health insurance**			
No	1.26 [1.19, 1.34]		**1.18 [1.11, 1.26]**
Yes	1		1
**Wealth index**			
poor	2.80 [2.63, 2.97]		**2.29 [2.14, 2.45]**
middle	1.79 [1.68, 1.92]		**1.57 [1.46, 1.68]**
Rich	1		1
**Region SSA**			
East Africa		1	**1**
West Africa		2.0 [1.92-2.04]	**2.38 [2.21, 2.55]**
**community ANC visit**			
Low		1.00 [0.93, 1.09]	0.93 [0.86, 1.00]
High		1	1
**community wealth index**			
Low		0.75 [0.70, 0.81]	**1.12 [1.02, 1.22]**
High		1	1
**Community media exposure**			
Low		1.01 [0.92, 1.10]	0.94 [0.86, 1.03]
High		1	1
**Community literacy level**			
Low		1.10 [1.00, 1.20]	**1.13 [1.03, 1.24]**
High		1	**1**

Model I: *individual level variable,* **Model II:** *Community level variable,* **Model III**: *Individual and community level variable*

Discussion

Barriers to accessing health care have contributed to maternal and child mortality and morbidity in developing countries such as those in Sub-Saharan Africa. This study aimed to assess the

magnitude and determinants of barriers to health care access among women of reproductive age in Sub-Saharan Africa.

In this study, the magnitude of barriers to accessing health care among reproductive age women was found to be 55.84 (95% CI: (55.57, 56.10) owing to one or more of the four factors, the most commonly cited being the difficulty in getting money and the distance from medical facilities. This finding is lower than the previous study conducted in South Africa 65% [18, 19], and Tanzania 65% [15], Ethiopia 71.8%,70.2, 69.9%[1, 20, 21], (SSA) 60.4%, 61.5% [17]. this variation could be due to the fact that socio-economic and variability in health infrastructure and health system policy, women's attitudes towards health care seeking behavior and cultural differences across countries in sub-Saharan Africa, moreover, this discrepancy might be due to study period, this study includes, the most recent DHS data set from 2019-2023. On the other hand, the finding of this study is similar to but slightly higher than in previous study in Gambia 45.5%[22], Ghana (51%)[23]. this variation could be due to the fact that socio-economic and variability in health infrastructure and health system policy, women's attitudes towards health care seeking behavior and cultural differences across countries in sub-Saharan Africa.

The odds of barrier of accessing health care were 1.30 times higher among reproductive women living in rural residency compared to women living in urban area. This finding is supported by previous studies conducted in Ethiopia [1, 20], guinea[24], Gambia, Tanzania and Ghana [15, 22, 25] respectively. Sub-Saharan[23], East Africa[26]. This association may be, due to the fact that rural areas have low health infrastructure i.e. inaccessibility of health facilities, less privileged and the influences of socio-cultural practices that need women to seek permission from their husband to visit the facility or women's decisions for seeking healthcare services is under their husband/partners control. Moreover, male involvement and support for women health care access are limited[1, 14, 23, 26]. This implies that the government should expand infrastructure services in rural area and encourage male involvement and support for improving health care access for reproductive age women[14].

The odds of perceived barrier of accessing health care were 1.23 and 1.21 times more likely to among reproductive age women whose husband had no education and primary education compared to women whose husband had secondary and above educational level respectively. This find was consistent with previous study conducted in east Africa, Ethiopia, Tanzania, Benin,

Bangladesh respectively [1, 15, 20, 26, 27]. The possible explanation could be in better access to the healthcare among women with educated husbands might be due to better participation and involvement of husbands in their families' health[23]. Women whose partners are educated are also likely to be informed regarding their fundamental human rights and may have higher health literacy. As a result, they are more likely to deal with any form of barrier to healthcare, compared to their counterparts who are less educated and may have lower health literacy [14, 15, 20, 28].

The odds of barrier to accessing health care were 1.33 times more likely to occur women delivered at home compared to women who had institutional delivery. This evidence was supported by study done in Senegal[29]. The plausible justification might be women who delivered at home may not get health information/counselling from healthcare providers that empower them to use other maternal healthcare services and women who deliver at home may face varous barriers to accessing health care such as financial constraint, lack of transportation, limited autonomy in healthcare decision and sociocultural norms that discourage institutional delivery. Moreover, importance of enhancing health system outreach, strengthen community-based education, and addressing sociocultural and socioeconomic disparities that affect health seeking behavior. On the other hand, the odds of barrier of accessing health care among woman who had no media exposure were 1.17 times more likely to compared with a woman who had media exposure. This evidence was supported by study done in Ethiopia[30], Nepal[31]. This could be the power of mass media in disseminating information concerning maternal health that may enhance women's knowledge and attitude towards health care access and utilization[31]. Barrier of accessing health care among woman who were not visiting health facility within 12 months were 9 % times less likely compared to women who had visiting health care facility within 12 months of study period. The odds of barrier to accessing health care among woman who had no health insurance utilization were 1.18 times more likely to compared with a woman who had used health insurance. This finding was supported with study done in Ethiopia[1], sub-Saharan Africa[23], Tanzania[15], Ghana[25]. The possible reason might be due to the fact that, individuals who were not had health insurance faced for unexpected catastrophic expenditure and difficulties in obtaining money for consultation of the doctor[1]. The odds of barrier to accessing healthcare among woman who were classified under poor and middle wealth status were 2.29 and 1.57 times more likely accessing health care difficulty compared to women who were classified under rich wealth index category**.** This evidenced was supported with study done in Ethiopia, [1, 30, 32, 33] Tanzania[15] Uganda[34], sub Saharan

Africa[14]. This finding might be explained by the fact that high out-of-pocket expenses and a lack of accessibility to healthcare facilities pose significant obstacles for middle-class and impoverished women in sub-Saharan Africa. Many people in the region cannot afford healthcare services because over 40% of health costs are paid for directly by individuals[35-38]

The odds of barrier of accessing health care among woman who live west Africa region were 2.38 times more likely barriers to accessing health care compared to east Africa region. According a recent joint report from the World Health Organization and the United Nations states that high out-of-pocket costs, a lack of adequate healthcare infrastructure, and other sociocultural barriers make it more difficult for women of reproductive age in West African nations such as Nigeria, Mali, and Sierra Leone to access maternal healthcare. On the other hand, East African countries like Rwanda, Kenya, and Ethiopia have strengthened their community health initiatives and gender-inclusive policies, which have improved women's autonomy in accessing healthcare services[39]. Furthermore, our current findings are supported by recent Demographic and Health Survey (DHS) program data (2022–2023), which indicate that women in West African countries are 2-3 times more likely to report delays in accessing healthcare due to cost, shortages of healthcare personnel, and long distances to health facilities. In contrast, East African countries, particularly Uganda and Tanzania, have strengthened their health insurance systems and rural outreach initiatives. Additionally, the World Bank's 2023 Health Service Coverage Index reveals that women in West Africa have significantly lower access to family planning, skilled birth attendance, and other essential reproductive health services[22, 40, 41]

Strength and limitation

This strength of study nationally representative data to evaluate contextual and individual characteristics related to barriers to healthcare access among reproductive age women in the SSA. Thus, findings can therefore, generalized to all women in their reproductive age in SSA, in order to account for the clusters within the sample, the study also employed advanced statistical models. This study has several limitations. The cross-sectional design restricts the ability to draw causal inferences, and health worker–related factors were not incorporated into the analysis. Furthermore, reliance on self-reported data may introduce recall or social desirability bias; future research should consider integrating objective measures, such as wearable devices, as demonstrated by Zhao et al. (2025)[42]

Conclusion and Recommendation

This study found that 55.55% of reproductive age women had barriers to accessing healthcare in SSA. Husband educational status, media exposure, internet utilization, place of residency, ANC visit status, place of delivery, health insurance utilization status, wealth index status, visiting health facility within 12 months, community literacy level and community wealth index and country category (western, eastern Africa) were significantly associated with barriers of accessing health care. Therefore, the Government and ministry of health in Sub-Saharan Africa countries should taking in to consideration those factors while designing policies and strategies targeting to health care access and tackling the preventable barriers to accessing health care in sub-Saharan Africa.

Declaration

Ethical approval and consent to participate

This study was based on analysis of existing survey datasets in the public domain (SSA) that are freely available online with all the identifier information anonymized, no ethical approval was required

Consent for publication

Not applicable

Data and material availability

Demographic and Health Survey were used, and it is publicly available online at (https://www.dhsprogram.com).

Competing interest

Authors Declared that, there is no competing interest.

Funding

Not applicable

Author's contributions

GAZ: involved in designing, data extraction, data analysis, interpretation, report and manuscript writing.

BT: involved in data analysis, interpretation, and, and manuscript writing.

BT: involved in analysis,

TTT: involved in designing the study, interpretation, report and

BSW: involved in analysis, and result interpretation

AFZ: involved in designing and interpretation study

EGM: analysis and manuscript writing

MW: Result interpretation

AA: manuscript writing: involved in data extraction

MSA: involved manuscript writing

Acknowledgements

We would like to say the DHS programs for allowing us to use the relevant DHS dataset in this study.

Reference

1. Tamirat, K.S., Z.T. Tessema, and F.B.J.B.H.S.R. Kebede, Factors associated with the perceived barriers of health care access among reproductive-age women in Ethiopia: a secondary data analysis of 2016 Ethiopian demographic and health survey. 2020. **20**: p. 1-8.
2. Erasmus, M.O., The barriers to access for maternal health care amongst pregnant adolescents in the Mitchells plain sub-district. 2017.
3. Washington, D.L., et al., Access to care for women veterans: delayed healthcare and unmet need. 2011. **26**: p. 655-661.
4. Regan, L.J.B.M.A., Addressing unmet needs in global women's health. 2018.
5. Izugbara, C., E. Covan, and E. Fugate-Whitlock, Women's health in Africa: Issues, challenges and opportunities. 2017: Routledge.
6. Organization, W.H., Primary health care on the road to universal health coverage: 2019 global monitoring report: Executive summary. 2019, World Health Organization.
7. Badiuzzaman, M., S.M. Murshed, and M.J.T.J.o.D.S. Rieger, Improving maternal health care in a post conflict setting: evidence from Chittagong Hill tracts of Bangladesh. 2020. **56**(2): p. 384-400.
8. Peters, D.H., et al., Poverty and access to health care in developing countries. 2008. **1136**(1): p. 161-171.
9. Alkire, B.C., et al., The economic consequences of mortality amenable to high-quality health care in low-and middle-income countries. 2018. **37**(6): p. 988-996.
10. Nyakang'o, S.B. and A.J.M. Booth, Women's perceived barriers to giving birth in health facilities in rural Kenya: A qualitative evidence synthesis. 2018. **67**: p. 1-11.
11. Colglazier, W.J.S., Sustainable development agenda: 2030. 2015. **349**(6252): p. 1048-1050.

12. Cahill, C.M., et al., Preoperative anemia management program reduces blood transfusion in elective cardiac surgical patients, improving outcomes and decreasing hospital length of stay. 2021. **61**(9): p. 2629-2636.
13. Peeri, N.C., et al., The SARS, MERS and novel coronavirus (COVID-19) epidemics, the newest and biggest global health threats: what lessons have we learned? 2020. **49**(3): p. 717-726.
14. Tessema, Z.T., et al., Determinants of accessing healthcare in Sub-Saharan Africa: a mixed-effect analysis of recent Demographic and Health Surveys from 36 countries. 2022. **12**(1): p. e054397.
15. Bintabara, D., K. Nakamura, and K.J.B.o. Seino, Improving access to healthcare for women in Tanzania by addressing socioeconomic determinants and health insurance: a population-based cross-sectional survey. 2018. **8**(9): p. e023013.
16. Kea, A.Z., et al., Exploring barriers to the use of formal maternal health services and priority areas for action in Sidama zone, southern Ethiopia. 2018. **18**: p. 1-12.
17. Zegeye, B., et al., Breaking barriers to healthcare access: a multilevel analysis of individual-and community-level factors affecting women's access to healthcare services in Benin. 2021. **18**(2): p. 750.
18. Silal, S.P., et al., Exploring inequalities in access to and use of maternal health services in South Africa. 2012. **12**: p. 1-12.
19. Tsawe, M. and A.S.J.B.r.n. Susuman, Determinants of access to and use of maternal health care services in the Eastern Cape, South Africa: a quantitative and qualitative investigation. 2014. **7**: p. 1-10.
20. Zegeye, B., et al., Barriers and facilitators to accessing health care services among married women in Ethiopia: a multi-level analysis of the Ethiopia demographic and health survey. 2021.
21. Demsash, A.W., B. Chakilu, and A. Mazengia, Knowledge Sharing Practice and Its Associated Factors Among Healthcare Providers at University of Gondar Comprehensive Specialized Hospital, North West Ethiopian: Cross-sectional Study. 2021.
22. Tsegaw, M., B. Mulat, and K.J.B.o. Shitu, Problems with accessing healthcare and associated factors among reproductive-aged women in the Gambia using Gambia demographic and Health Survey 2019/2020: a cross-sectional study. 2023. **13**(8): p. e073491.
23. Seidu, A.-A.J.P.O., Mixed effects analysis of factors associated with barriers to accessing healthcare among women in sub-Saharan Africa: Insights from demographic and health surveys. 2020. **15**(11): p. e0241409.
24. Seidu, A.-A., et al., Individual and contextual factors associated with barriers to accessing healthcare among women in Papua New Guinea: insights from a nationwide demographic and health survey. 2021. **13**(6): p. 573-585.
25. Seidu, A.-A., et al., Barriers to accessing healthcare among women in Ghana: a multilevel modelling. 2020. **20**: p. 1-12.
26. Minyihun, A., Z.T.J.R.M. Tessema, and H. Policy, Determinants of access to health care among women in East African countries: a multilevel analysis of recent demographic and health surveys from 2008 to 2017. 2020: p. 1803-1813.
27. Sen, K.K., et al., Do education and living standard matter in breaking barriers to healthcare access among women in Bangladesh? 2023. **23**(1): p. 1431.

28. Dimbuene, Z.T., et al., Women's education and utilization of maternal health services in Africa: a multi-country and socioeconomic status analysis. 2018. **50**(6): p. 725-748.
29. Zegeye, B., et al., Predictors of institutional delivery service utilization among women of reproductive age in Senegal: a population-based study. 2021. **79**: p. 1-11.
30. Demsash, A.W., A.D.J.B.H. Walle, and C. Informatics, Women's health service access and associated factors in Ethiopia: application of geographical information system and multilevel analysis. 2023. **30**(1).
31. Tamang, T.M. Factors associated with completion of continuum of Care for Maternal Health in Nepal. in IUSSP XXVIII International Population Conference, Cape Town, South Africa. 2017.
32. Fentie, E.A., et al., Spatial distribution and determinants of barriers of health care access among female youths in Ethiopia, a mixed effect and spatial analysis. 2023. **13**(1): p. 21517.
33. Alamneh, T.S., et al., Barriers for health care access affects maternal continuum of care utilization in Ethiopia; spatial analysis and generalized estimating equation. 2022. **17**(4): p. e0266490.
34. Kalule-Sabiti, I., A.Y. Amoateng, and M.J.A.P.S. Ngake, The effect of socio-demographic factors on the utilization of maternal health care services in Uganda. 2014: p. 515-525.
35. Shiferaw, S., et al., Why do women prefer home births in Ethiopia? 2013. **13**: p. 1-10.
36. Yaya, S., G. Bishwajit, and V.J.B.g.h. Shah, Wealth, education and urban–rural inequality and maternal healthcare service usage in Malawi. 2016. **1**(2): p. e000085.
37. Voto, T.P., B.E. Voto, and N.J.E. Ngepah, The Impact of Out-of-Pocket Health Expenditure and Public Health Expenditure on Poverty in Sub-Saharan Africa. 2025. **13**(5): p. 134.
38. Alamneh, T.S., et al., Socioeconomic inequality in barriers for accessing health care among married reproductive aged women in sub-Saharan African countries: a decomposition analysis. 2022. **22**(1): p. 130.
39. AKINWALE, A.A.J.I.J.o.I.R. and A. Studies, Gender equity and social progress: empowering women and girls to drive sustainable development in Sub–Saharan Africa. 2023.
40. Arhin, K., E.F. Oteng-Abayie, and J.J.H.e.r. Novignon, Assessing the efficiency of health systems in achieving the universal health coverage goal: evidence from Sub-Saharan Africa. 2023. **13**(1): p. 25.
41. Muhajarine, N., et al., Inequalities and factors associated with maternal healthcare services utilisation in Mozambique: evidence from the Demographic and Health Survey 2022−2023. 2025. **10**(5).
42. Zhao, Y., et al., Highly sensitive, wearable piezoresistive methylcellulose/chitosan@ MXene aerogel sensor array for real-time monitoring of physiological signals of pilots. 2025. **68**(2): p. 542-551.

Figure legend

Fig 1: Barriers to accessing health care and determinant factor among reproductive age women in sub-Saharan Africa

Manuscript Click here to access/download;Manuscript;Barrier to women1.docx

Barriers to accessing health care among women's in Sub-Saharan Africa: Multilevel analysis of recent demographic and health survey from 2019-2023.

Gebreeyesus Abera Zeleke[1]*, Enyew Getaneh Mekonen[1], Bewuketu Terefe[2], Mulugeta Wassie[3], Agazhe Aemro[3], Alebachew Ferede Zegeye[3], Mohammed Seid Ali[4], Berhan Tekeba[4], Tadesse Tarik Tamir[4], Belayneh Shetie Workneh[5]

[1] Department of Surgical Nursing, School of Nursing, College of Medicine and Health Sciences, University of Gondar, Gondar, Ethiopia

[2] Department of Community Health Nursing, School of Nursing, College of Medicine and Health Sciences, University of Gondar, Gondar, Ethiopia.

[3] Department of Medical Nursing, School of Nursing, College of Medicine and Health Sciences, University of Gondar, Gondar, Ethiopia;

[4] Department of Pediatrics and Child Health Nursing, School of Nursing, College of Medicine and Health Sciences, University of Gondar, Gondar, Ethiopia.

5 Department of Emergency and Critical Care Nursing, School of Nursing, College of Medicine and Health Sciences, University of Gondar, Gondar, Ethiopia

Correspondence Author*
Gebreeyesus Abera Zeleke

Department of Surgical Nursing, School of Nursing, College of Medicine and Health Science, University of Gondar, Gondar, Ethiopia.

Email address: gebreeyesusabe143@gmail.com

Co-authors:

Enyew Getaneh Mekonen **Email Address:** enyewgetaneh12@gmail.com

Bewuketu Terefe: **Email address**: woldeabwomariam@gmail.com

Mulugeta Wassie: **Email address:** mulugeta2113@gmail.com

Agazhe Aemro: **Email address:** agazhe1049@gmail.com

Alebachew Ferede Zegeye: **Email address:** alexferede24@gmail.com

Mohammed Seid Ali: **Email address:** muheseid2592@gmail.com

Berhan Tekeba: **Email address:** berishboss7@gmail.com

Tadesse Tarik Tamir: **Email address:** tadestar140@gmail.com
Belayneh Shetie Workneh: **Email address:** belay2319@gmail.com

// Abstract

Background:

Barriers to accessing healthcare have significantly contributed to the high rates of maternal and child mortality and morbidity in developing regions, particularly in Sub-Saharan Africa. Access to healthcare is shaped by multiple factors, including financial constraints, geographic location, the availability of services, and the quality of care provided. This study aimed to assess the magnitude of these barriers and to identify the factors influencing healthcare access among women of reproductive age in Sub-Saharan Africa.

Methods:

This study utilized secondary data from the most recent Demographic and Health Surveys conducted between 2019 and 2023 across Sub-Saharan Africa. A weighted sample of 134,470 women of reproductive age was included in the analysis. Data were analyzed using Stata version 14, employing a multilevel mixed-effects logistic regression model. Factors associated with barriers to healthcare access were considered statistically significant at P-values less than 0.05. Adjusted odds ratios (AORs) with 95% confidence intervals (CIs) were used to interpret the results.

Result: In this study, the magnitude of barriers to accessing healthcare among reproductive age women were found to be 55.84% (95% CI: 55.57–56.10). Husband's educational status [AOR = 1.23, 95% CI: 1.15–1.30], Media exposure [AOR = 1.17, 95% CI: 1.11–1.24], Antenatal care visit [AOR = 1.31, 95% CI: 1.10–1.55], and place of delivery [AOR = 1.33, 95% CI: 1.24–1.43]. Health insurance utilization [AOR = 1.18, 95% CI: 1.11–1.26], Wealth index, 2.29 [2.14, 2.45], 1.57 [1.46, 1.68] and visiting a health facility within the past 12 months [AOR = 0.91, 95% CI: 0.87–0.97] were individual associated variable. In addition, place of residence [AOR = 1.30, 95% CI: 1.22–1.39], community literacy level [AOR = 1.13, 95% CI: 1.03–1.24], community wealth index [AOR = 1.12, 95% CI: 1.02–1.22], and Region were associated with barriers to accessing healthcare.

Conclusion and recommendation: This study found that 55.55% of women in Sub-Saharan Africa experienced barriers to accessing healthcare. Educational status, media exposure, place of residence, antenatal care attendance, place of delivery, health insurance coverage, wealth index, recent visits to health facilities, community literacy levels, community wealth status, and regional differences were determinant factors. These findings highlight the need for Sub-Saharan African governments to prioritize these determinants when formulating policies and strategies aimed at reducing preventable barriers to healthcare access in the region.

Key words: Access to Healthcare; Barriers to Healthcare; Sub-Saharan Africa; Reproductive Age Women

Introduction

Access to healthcare is broadly defined as the ability to prevent illness, disability, and premature death, to promote and maintain health, and to attain health equity, particularly for women of reproductive age, by offering inclusive, high-quality care and the best possible availability, affordability, and accessibility of medical services[1-3]. Reproductive-age women's health is a critical global public health concern since it influences population health outcomes and fosters socioeconomic growth[4]. The advancement of socioeconomic development is significantly influenced by the health of women of reproductive age, especially in Africa. [5]. The Sustainable Development Goals (SDGs) place a strong emphasis on women's health, with Targets 3.7 and 3.8 of SDG 3 specifically focusing on achieving universal health coverage by 2030 and ensuring access to sexual and reproductive health services, including education and information on family planning[6, 7]. The Sustainable Development Goals (SDGs) explicitly target a substantial reduction in maternal mortality, with Goal 3.1 aiming to lower the global maternal mortality ratio to fewer than 70 per 100,000 live births by 2030. While these global efforts have contributed to some improvements in women's health outcomes, they have yet to fully address the root causes of maternal mortality. Achieving sustained and comprehensive progress remains a critical challenge. In 2016, Sub-Saharan Africa accounted for a disproportionately high share of the 303,000 maternal deaths reported globally, the majority of which were due to preventable causes. Moreover, non-communicable diseases continue to contribute significantly to female morbidity and mortality across nearly all countries, with a particularly severe impact in low- and middle-income settings, where many women are left with long-term disabilities [4-6]. Access to healthcare involves multiple dimensions, including financial affordability, geographic accessibility, availability of medical services, and the quality of care provided in health facilities. [8]. Access to healthcare is a key determinant of whether individuals utilize health services. Globally, approximately 400 million people lack essential healthcare, and around eight million die each year from preventable diseases—resulting in an estimated economic loss of $6 trillion in low- and middle-income countries. [6, 9, 10]. In 2015, world leaders pledged to achieve universal health coverage as part of the Sustainable Development Goals (SDGs), aiming to ensure access to affordable, high-quality medicines and financial protection for all [11, 12]. In 2017, the World Bank and WHO reported that half of the global population lacks access to essential health services. As a result, many countries in Sub-Saharan Africa have made universal healthcare a key part of their national health

strategies. However, there has been slow progress in turning this commitment into tangible results, particularly in securing national funding for equitable, high-quality healthcare, financial security, and overall health [6, 9, 13, 14]. Reproductive age women and child mortality from low family planning uptake, unsafe abortion practices, unintended pregnancies, and an increase in home births are all implications of women in reproductive age having trouble accessing health care[14]. Previous research has offered some evidence regarding the sociodemographic and economic factors that can be barriers to accessing healthcare, such as marital status, place of residence, maternal age, wealth index, educational attainment, and unemployment status. Nevertheless, women of reproductive age continue to face significant disparities in healthcare utilization and access[10, 14-16]. This study makes three unique contributions to the body of existing literature. In order to provide a current and policy-relevant analysis of the barrier to healthcare access, it first makes use of the most recent (2019–2023) Demographic and Health Survey (DHS) data. Second, our analysis takes a harmonized multi-country approach across ten sub-Saharan African nations, improving cross-national comparability and generalizability in contrast to many previous studies that are limited to single-country analyses or rely on older datasets. In order to gain a better understanding of the structural and contextual factors that influence healthcare access, the study employs a comprehensive multilevel analysis of determinants at the individual and community levels. These contributions put our research in a position to guide focused, empirically supported programs provide fair access to healthcare for reproductive age women in sub-Saharan. Therefore, this study aimed to ascertain the magnitude and potential contributing factors associated with barriers to accessing healthcare among women of reproductive age in sub-Saharan Africa.

Methods and materials

Patient and public involvement statement

Secondary data from the Demographic and Health Surveys (DHS) were used in this investigation.

Study design, study area, and period.

Data from ten Sub-Saharan African countries, collected between 2019 and 2023, were used to perform a multilevel mixed-effects analysis, based on the most recent Demographic and Health Surveys (DHS). The DHS collects community-based, cross-sectional data approximately every five years to generate updated health and health-related indicators.

Data source, study population and sampling technique

A secondary data analysis was conducted using Demographic and Health Survey (DHS) data collected between 2019 and 2023 from selected sub-Saharan African countries. The study specifically included DHS datasets from ten countries Burkina Faso, Gabon, Ghana, Kenya, Liberia, Rwanda, Sierra Leone, Tanzania, The Gambia, and Mozambique each of which had recent and publicly available survey data within the specified timeframe. The datasets were appended to assess the extent of, and factors underlying, barriers to healthcare access among women of reproductive age in sub-Saharan Africa. Each country's DHS comprises multiple datasets. The DHS employs a stratified, two-stage cluster sampling design: in the first stage, enumeration areas are selected, followed by systematic sampling of households within each selected area in the second stage.

The variable barrier to accessing health care were generated by computation of (v467b, v467c, v467d, v467f) from maternal recode (IR) data set was recoded. A binary logistic regression model was used to identify determinants a factor barrier to access the health care. Determinants of barrier to accessing the health care were reported in terms of an AOR with a significance level of (95%). In the univariate analysis, at 95% confidence intervals with a p-value of < 0.25 was considered for the candidate multivariable analysis of data. All variables with p values of <0.05 were considered statistically significant. Since DHS data are nationally representative, stratified multistage cluster sampling is required; therefor, sampling weights must be applied to produce unbiased estimates. To correctly weight DHS data, we use the sampling weights provided in the dataset, specifically the individual weight **(**v005**)** for women's individual recode files. This weight must be divided by 1,000,000 (v005/1000000). using "gen wgt=v005/1000000 Stata commands. A total weighted sample of 134,470 reproductive age women were included in the study **(Table 1).**

Table 1: The weighted Sample size for magnitude and determinants of barrier to accessing health care among reproductive age women in sub-Saharan Africa**.**

Country	Year of survey	weighted sample (n)	weighted sample (%)
Brukinefaso	2021	17659	13.13
Gabon	2019-2021	6507.15	4.83
Ghana	2022	15014	11.16
Kenya	2022	16716.36	12.43
Liberia	2019-2020	8065	5.99
Rwanda	2019-2020	14634	10.88
Sera lion	2019	15574	11.58
Tanzania	2022	15254	11.34
Gambia	2019-2020	11865	8.08
Mozambique	2023	13181.93	9.80
TOTAL		134470	100%

Study variable

Outcome variables: The outcome of this study was barrier to access the health care. The Data in barrier of accessing the health care were collected among reproductive age women who used one of the health cares. Measurement of the outcome variables was determined by using computation of four variables; such as Distance to health facility (v467b), difficulty in obtaining money (v467c), getting permission go to the doctor (v467d) and not wanting to go alone (v467f). If a woman of reproductive age reported experiencing at least one of the following challenges, such as difficulty in obtaining money, distance to a health facility, lack of permission to seek treatment, or reluctance to go alone were considered to face significant barriers to healthcare access. These cases were coded as '1' and labeled as 'big problem.' Conversely, women who did not report any of these four barriers were considered to have no significant barriers to healthcare access. These were coded as '0' and labeled as 'no big problem'. This approach is consistent with prior literature using DHS data[17]

Independent Variable: Hence DHS data are hierarchical, independent factors come from two sources that was at individual and community levels factors were considered for this analysis. The individual-level independent variables were women's age (15-19, 20-35, 36-49), Women educational status (No education, Primary education , Secondary & Higher education), women occupation (working, not working), Religion (Muslim, Christian , Others), Wealth index (Poor, Middle, Rich) media exposure (yes, No), internet utilization (yes, No), Residency (urban, rural), Husband educational status (No education, Primary education, secondary& higher education), Husband occupational status (No working, Working), Currently Contraceptive (No, Yes) Visiting health facility in 12 month (No, Yes), place of delivery (Home , Institutional delivery), ANC visit (No antenatal visit, 1-4 visit, > 4 visit)

The community-level variables: Poverty level (Low, High), Literacy level (Low, High), Media exposure, (Low, High), Residency (Urban, rural), Region (East, west), Community ANC visit

Data processing and statistical analysis

Stata version 14 statistical software was used to clean, record, and analyze secondary DHS data sets. The variables in the DHS data are grouped into clusters, and the variables within a cluster are more similar to each other than those in other clusters. The presumptions of equal variance

across clusters and independent data were violated in order to run a typical logistic regression model. This suggests that between-cluster factors must be considered using a complex model.

Given this, multilevel mixed-effects logistic regression was used to determine the factors that associated with barrier to accessing the health care. Multilevel mixed effect logistic regression follows four models. The null model (only outcome variable), mode I (individual level variable such as age, Religious status**,** educational status, Husband educational, marital occupation**,** Husband occupation**,** marital status**,** Contraceptive Utilization, place of delivery, ANC visit, media exposure, Intermate utilization**,** visiting health facility within 12 months, health insurance , wealth index) model II community level variables, Region in sub-Saharan Africa**,** community poverty level, community media exposure, Community distance health facility, Community literacy level, Community ANC visit), and model III (both individual and community level variables such as age, Religious status**,** educational status, Husband educational, marital occupation**,** Husband Occupation**,** marital status**,** Contraceptive Utilization, place pf delivery, ANC visit, media exposure, Intermate utilization**,** visiting health facility within 12 months, health insurance , wealth index), model II (community level variables, Region in sub-Saharan Africa**,** community poverty level, community media exposure, Community distance health facility, Community literacy level, Community ANC visit). The null model without independent variables was used to check the variability of outcome variable across the cluster. The relationship of individual-level variables with outcome variable (Model I) and the association of community-level variables with outcome variable (Model II) were determined. In the final model, the association of both individual and community-level variables was fitted with the outcome variable.

Random effects

The intra-class correlation coefficient (ICC), proportionate change in variance (PCV), and median odds ratio (MOR) were used to quantify random effects or measures of variation of the outcome variables.

To assess the variation between clusters, the proportionate change in variance (PCV) and intra-cluster correlation coefficient (ICC) were calculated. By treating the cluster as a random variable, the ICC shows that the variation in the health care access barrier between clusters may be computed as follow $\boldsymbol{ICC} = \frac{\sigma^2 uo}{\sigma^2 uo + \pi^2/3} \times \mathbf{100\%}$. The MOR, which is determined by using clusters as a

random variable and calculating the median value of the odds ratio between the highest-risk and lowest-risk areas for health care access barriers, is calculated. $MOR = \exp(0.6745\ \sqrt{(2 * \sigma^2 uo)})$ Furthermore, the PCV calculates a variable that explains the variation in the degree of barriers to accessing healthcare ; $PCV = \frac{\sigma^2 uo - \sigma^2}{\sigma^2 uo} \times 100\%$;. where VC is the cluster level variance and Vnull is the variance of the null model.

The associations between independent factors at the individual and community levels and barriers to accessing health care were estimated using the fixed effects. It was assessed and presented using adjusted odds ratio (AOR) and 95% confidence intervals with a p-value of < 0.05. Because of the nested nature of the model, deviation = -2(log likelihood ratio) was used to compare models, and the model with the lowest deviance was selected as the best-fitted model. By evaluating the variance inflation factors, the model assumption for multi-collinearity was confirmed. In addition, Model comparison was performed using Akaike's Information Criterion (AIC). The model includes both random intercepts and random slopes (Model III) was evaluated alongside alternative models, with AIC values calculated to assess relative model fit and parsimony.

Ethical approval and consent to participate

This study was based on analysis of existing survey datasets in the public domain (SSA) that are freely available online with all the identifier information anonymized, no ethical approval was required

Result

In this study a total of 134,470 reproductive age women were included in Sub-Saharan African. The Mean age of participants were 28.99 (Std. Dev. 9.68) years. More than two thirds (69.8%) of women did not use contraception, and over 72.22% reproductive women did not use health insurance, and over half of (54.22%) women were living rural areas in sub-Saharan Africa **(Table 2)**

Table 2: Sociodemographic and economic characteristics of reproductive women who had barriers to accessing health care in sub-Saharan Africa.

Variable	frequency(n)	percentage (%)
**Individual and community level variables**		

**Maternal age**		
15-19	27890.4	20.7
20-35	69441.4	51.64
36-49	37138.6	27.64
**Residency**		
Urban	61558.03	45.78
Rural	72912.41	54.22
**Religious status**		
Muslim	48834.95	41.96
Christian	46856.90	39.30
Other	23524.59	19.73
**Educational Status**		
No formal education	35051.70	26.06
Primary education	39651.17	29.48
Secondary and higher education	59767.57	44.44
**Husband educational**		
No formal education	29414.44	36.72
Primary education	21245.78	26.52
secondary and higher education	29428.78	36.74
**Maternal Occupational**		
Not working	50732.90	37.81
working	83417.01	62.19
**Husband Occupation**		
Not working	10390.43	13.00
Working	69698.11	87.00
**Maternal status**		
single	42213.40	31.4
Married	58262.00	43.3
Other	33995.4	25.3
**Contraceptive Utilization**		
No	93855.00	69.8
Yes	40615.5	30.2
**place of delivery**		
Home	8279.46	15.5
Institution	44979.28	84.5
ANC visit		
No visiting	2012.84	3.77
1-4 visiting	24459.34	45.92
>4 visiting	26786.55	50.29

Media exposure

No	34628.45	25.75
Yes	99831.57	74.24
**Intermate utilization**		
No	93020.34	69.17
Yes	41450.10	30.82
**Visiting health facility 12 month**		
No	57137.73	42.49
Yes	77332.71	57.50
**Health insurance**		
No	85044.69	72.22
Yes	32709.39	27.77
**Wealth index**		
poor	47227.18	35.12
middle	25771.45	19.16
Rich	61471.81	45.74
**Region in sub-Saharan Africa**		
East Africa	59786.29	44.46
West Africa	74684.16	55.54
**Community poverty level**		
Low	70776.71	52.63
High	63693.73	47.47
**Community media exposure**		
Low	61642.30	45.84
High	72828.14	54.15
**Community distance health facility**		
Low	65962	49.00
High	68508	51.00
**Community literacy level**		
Low	57615.01	42.84
High	76855.43	57.15
**Community ANC visit**		
Low	77912.32	57.96
High	56491.92	42.03

In this study, the magnitude of barriers to accessing health care among reproductive age women was found to be 55.84 (95% CI: (55.57, 56.10) owing to one or more of the four factors, the most commonly cited being the difficulty in getting money and the distance from medical facilities (**Figure 1)**

Random effect (Measures of variation) and model fitness

To determine if the data supported the choice to evaluate randomness at the community level, a null model was employed. With the variance of 0.3784967 and a p-value of < 0.001, the null model's results showed that there were notable variations in the barriers to accessing healthcare among clusters. A significant amount of the variance in the outcome between clusters was jointly explained by both individual- and community-level variables, as evidenced by the intra-cluster correlation (ICC), which dropped from 10.31% in the null model to 3.96% in the fully adjusted model (model III). According to the proportionate change in variance (PCV), Model III explains the greatest percentage (64.09%) of the group-level variation in comparison to the null model, group comparison showed that, Model I (50.19%) and Model II (24.56%). This suggests that the model fits better as more predictors are included. Model comparison using Akaike's Information Criterion (AIC) indicated that Model III achieved the best fit, as evidenced by the lowest AIC (239,608.6), markedly lower than both the null model (AIC = 1,128,437) and Model II (AIC = 1,127,704). Model I also showed good fit (AIC = 239,764.8), though slightly less optimal than Model III. Multicollinearity using variance inflation factors (VIF) indicated that all VIF values ranged from 1.03-1.98, which is within accepted range **(Table 3)**

Table 3: Model comparison and Fitness random effect analysis for barriers of accessing health care of among reproductive age women in sub-Saharan Africa**.**

**Parameter**	**Null model**	**model I**	**model II**	**model III**
Variance	0.3784967	0.1885269	0.28553	0.1358817
ICC	10.31%	5.41%	7.98%	3.96%
MOR	1.79	1.51	1.66	1.41
PCV	Reference	50.19%	24.56%	64.09%
**Model fines**				
LLR	-90175.365	-21798.27	-86841.704	-21433.284
Deviance	1803596.73	43596.54	173683.408	42866.568
AIC	1128437	239764.8	1127704	239608.6

ICC: intra-cluster correlation**, LLR:** loglikelihood ratio**, MOR:** median odds ratio**, PCV:** proportional change in variance. **AIC:** Akaike information criteria.

Determinants of barriers to accessing health care among reproductive age women in Sub-Saharan Africa

Bivariable logistic regression analysis of individual and community level variables associated with barriers to accessing health care among reproductive age women in Sub-Saharan Africa

Bivariable multilevel logistic regression analysis indicated that husband's educational status, media exposure, internet utilization, place of residence, antenatal care attendance, place of delivery, health insurance coverage, community wealth index, recent health facility visits, and country category (Western vs. Eastern Africa) were associated with barriers to accessing health care among reproductive-age women at a p-value < 0.25 (**Table 4**)

Table 4: Bivariable logistic regression of individual and community level factors associated with barriers to accessing health care among reproductive age women in Sub-Saharan Africa.

Variables	Crude Odds Ratio [COR]	P. value	[95% CI]
**Maternal age**			
15-19	0.86	<0.001	[0.83, 0.89]
20-35	0.86	<0.001	[0.84, 0. 88]
36-49	1		1
**Residency**			
Urban	1		1
Rural	2.09	<0.001	[2.04, 2.14]
**Maternal education**			
No education	2.40	<0.001	[2.33, 2.46]
Primary education	1.52	<0.001	[1.48, 1.56]
Secondary & Higher	1		1
**Husband education**			
No education	2.43	<0.001	[2.35, 2.51]
Primary education	1.55	<0.001	[1.49, 1.60]
secondary & higher	1		1
**Maternal occupation**			
working	1		1
not working	0.94	<0.001	[0.92, 0.96]
**Husband occupation**			
working	1		
not working	1.07	<0.001	[1.02, 1.12]
**Religion**			
Muslim	1		1
Christian	1.32	<0.001	[1.29, 1.35]
Other	1.08	<0.001	[1.04, 1.11]

**Contraceptive utilization**			
No	1.18	<0.001	[0.82, 0.86]
Yes	1		1
**Place of delivery**			
Home	1.84	<0.001	[1.75, 1.93]
Institution	1		1
**ANC visit**			
No visit	1.16	<0.002	[1.05, 1.27]
1-4 visit	1.05	<0.003	[1.01, 1.09]
> 4 visits	1		1
**Marital status**			
single	1		
Married	1.23	<0.001	[1.20, 1.26]
Other	1.32	<0.001	[1.29, 1.36]
**Media exposure**			
No	1.90	<0.001	[1.85, 1.95]
Yes	1		1
**Internet utilization**			
No	2.321822	<0.001	[2.26, 2.37]
Yes	1		1
**Visiting health facility 12 month**			
No	1.02	<0.046	[1.00, 1.04]
Yes	1		1
**Health insurance**			
No	1.21	<0.001	[1.18, 1.25]
Yes	1		1
**Wealth index**			
poor	3.58	<0.001	[3.49, 3.67]
middle	2.08	<0.001	[2.02, 2.14]
Rich	1		1
**Region SSA**			
East Africa	1.68	<0.001	[1.65, 1.72]
West Africa	1		1
**community ANC visit**			
Low	0.86	<0.001	[0.85, 0.88]
High	1		1
**community wealth index**			
Low	0.69	<0.001	[0.67, 0.70]
High	1		
**Community media exposure**			
Low	0.73	<0.001	[0.72, 0.75]
High	1		1
**Community literacy level**			
Low	0.77	<0.001	[0.75, 0.79]
High	1		1

Multivariable multilevel logistic regression analysis of individual and community level factors associated with Barriers to health care access among reproductive Age Women in Sub-Saharan Africa.

In the final fitted model of multivariable multilevel logistic regression, Husband educational status, media exposure, place of residency, Antenatal care visit, place of delivery, health insurance utilization, wealth index status, visiting health facility within 12 months, and country category (western, eastern Africa) were significantly associated with barriers of accessing health care among reproductive age women in sub-Saharan Africa. The odds of barrier of accessing health care were 1.30 times higher among reproductive women living in rural residency compared to women living in urban area **[AOR=**1.30, 95 CI: (1.22, 1.39)]. and the odds of barrier of accessing health care were 1.23 times more likely to among reproductive age women whose husband had no education compared to women whose husband had secondary and higher education [**AOR**=1.23, 95 CI: (1.15, 1.30,)]. In addition, odds of barrier of accessing health care were 1.33 times more likely to occur women delivered at home compared to women who had institutional delivery. **(AOR**=1.33, 95% CI: (1.24, 1.43) and the odds of barrier of accessing health care were 1.31 times higher among women who had no history of antenatal visit compared to women who had more than four ANC visit. [**AOR**= 1.31, 95% CI: (1.10, 1.55)]. The odds of the barrier of accessing health care among reproductive age woman who had no media exposure were 1.17 times more likely to compared with a woman who had media exposure **[AOR**=1.17, 95% CI: (1.11, 1.24)] and the barrier of accessing health care among reproductive age woman who were not visiting health facility within 12 months 9% times less likely compared to women who had visiting health care facility within 12 months of study period **[AOR=**0.91, 95% CI: (0.87, 0.97)]. The odds of perceived barrier of accessing health care among reproductive age woman who had no health insurance utilization were 1.18 times more likely to compared with a woman who had used health insurance [**AOR=**1.18 95% CI: (1.11, 1.26)] and The odds of perceived barrier of accessing health care service among reproductive age woman who were classified under poor and middle wealth status were 2.29 and 1.57 times more likely barries accessing health care compared to reproductive women who were classified under rich wealth status category **[AOR**=2.29 95% CI: (2.14, 2.45)] and [**AOR**=1.57, 95% CI: (1.46, 1.68)]. The odds of perceived barrier of accessing health care service among reproductive age woman who reside in west Africa region were 2.38 times more

likely barriers accessing health care compared to east Africa region category. [**AOR**=2.38, 95% CI: (2.21, 2.55)] **(Table 5).**

Table 5: Multivariable multilevel logistic regression of individual and community level factors associated with barriers to accessing health care among reproductive age women in Sub-Saharan Africa.

Individual and community level factors	Model I AOR [95% CI]	Model II AOR [95% CI]	Model III AOR [95% CI]
**Maternal age**			
15-19	0.96 [0.86, 1.07]		0.96 [0.86,1.08]
20-35	1.06 [1.01,1.13]		1.04 [0.99, 1.11]
36-45	1		1
**Residency**			
Urban		1	1
Rural		2.29 [2.24, 2.35]	**1.30 [1.22, 1.39]**
**Maternal education**			
No education	1.10 [1.02, 1.18]		1.04 [0.97, 1.11]
Primary education	0.93 [0.87, 0.99]		1.06 [0.99, 1.14]
Secondary and above	1		1
**Husband education**			
No education	1.26 [1.19,1.35]		**1.23 [1.15, 1.30]**
Primary education	1.04 [0.97,1.12]		1.23 [0.67, 1.22]
secondary & higher education	1		1
**Maternal occupation**			
working	1		1
not working	0.99 [0.94, 1.04]		**1.02 [1.01, 1.11]**
**Husband occupation**			
working	1		1
not working	0.90 [0.83, 0.97]		1.00 [0.92, 1.08]
**Religion**			
Muslim	1		1
Christian	1.62 [1.53, 1.71]		0.46 [0.57, 1.94]
Other	1.22 [1.13, 1.31]		1.36 [0.75, 1.46]
**Contraceptive utilization**			
No	1.11 [1.05,1.17]		1.03 [0.98, 1.08]
Yes	1		1
**Place of delivery**			
home	1.30 [1.21, 1.40]		**1.33 [1.24, 1.43]**
Institution	1		**1**

**Antenatal Care Visit**			
No	1.01 [0.85, 1.19]		**1.31 [1.10, 1.55]**
1-4 visit	0.94 [0.89, 0.99]		1.13 [0.37, 1.19]
> 4 visits	1		1
**Media exposure**			
No	1.14 [1.08, 1.21]		**1.17 [1.11, 1.24]**
Yes	1		1
**Internet utilization**			
No	1.69 [1.58, 1.80]		1.68 [0.67, 1.79]
Yes	1		1
**Visiting health facility 12 month**			
No	0.92 [0.87, 0.98]		**0.91 [0.87, 0.97]**
Yes	1		1
**Health insurance**			
No	1.26 [1.19, 1.34]		**1.18 [1.11, 1.26]**
Yes	1		1
**Wealth index**			
poor	2.80 [2.63, 2.97]		**2.29 [2.14, 2.45]**
middle	1.79 [1.68, 1.92]		**1.57 [1.46, 1.68]**
Rich	1		1
**Region SSA**			
East Africa		1	**1**
West Africa		2.0 [1.92-2.04]	**2.38 [2.21, 2.55]**
**community ANC visit**			
Low		1.00 [0.93, 1.09]	0.93 [0.86, 1.00]
High		1	1
**community wealth index**			
Low		0.75 [0.70, 0.81]	**1.12 [1.02, 1.22]**
High		1	1
**Community media exposure**			
Low		1.01 [0.92, 1.10]	0.94 [0.86, 1.03]
High		1	1
**Community literacy level**			
Low		1.10 [1.00, 1.20]	**1.13 [1.03, 1.24]**
High		1	**1**

Model I: *individual level variable,* **Model II:** *Community level variable,* **Model III**: *Individual and community level variable*

Discussion

Barriers to accessing health care have contributed to maternal and child mortality and morbidity in developing countries such as those in Sub-Saharan Africa. This study aimed to assess the

magnitude and determinants of barriers to health care access among women of reproductive age in Sub-Saharan Africa.

In this study, the magnitude of barriers to accessing health care among reproductive age women was found to be 55.84 (95% CI: (55.57, 56.10) owing to one or more of the four factors, the most commonly cited being the difficulty in getting money and the distance from medical facilities.

This finding is lower than the previous study conducted in South Africa 65% [18, 19], and Tanzania 65% [15], Ethiopia 71.8%,70.2, 69.9%[1, 20, 21], (SSA) 60.4%, 61.5% [17]. this variation could be due to the fact that socio-economic and variability in health infrastructure and health system policy, women's attitudes towards health care seeking behavior and cultural differences across countries in sub-Saharan Africa, moreover, this discrepancy might be due to study period, this study includes, the most recent DHS data set from 2019-2023. On the other hand, the finding of this study is similar to but slightly higher than in previous study in Gambia 45.5%[22], Ghana (51%)[23]. this variation could be due to the fact that socio-economic and variability in health infrastructure and health system policy, women's attitudes towards health care seeking behavior and cultural differences across countries in sub-Saharan Africa.

The odds of barrier of accessing health care were 1.30 times higher among reproductive women living in rural residency compared to women living in urban area. This finding is supported by previous studies conducted in Ethiopia [1, 20], guinea[24], Gambia, Tanzania and Ghana [15, 22, 25] respectively. Sub-Saharan[23], East Africa[26]. This association may be, due to the fact that rural areas have low health infrastructure i.e. inaccessibility of health facilities, less privileged and the influences of socio-cultural practices that need women to seek permission from their husband to visit the facility or women's decisions for seeking healthcare services is under their husband/partners control. Moreover, male involvement and support for women health care access are limited[1, 14, 23, 26]. This implies that the government should expand infrastructure services in rural area and encourage male involvement and support for improving health care access for reproductive age women[14].

The odds of perceived barrier of accessing health care were 1.23 and 1.21 times more likely to among reproductive age women whose husband had no education and primary education compared to women whose husband had secondary and above educational level respectively. This find was consistent with previous study conducted in east Africa, Ethiopia, Tanzania, Benin,

Bangladesh respectively [1, 15, 20, 26, 27]. The possible explanation could be in better access to the healthcare among women with educated husbands might be due to better participation and involvement of husbands in their families' health[23]. Women whose partners are educated are also likely to be informed regarding their fundamental human rights and may have higher health literacy. As a result, they are more likely to deal with any form of barrier to healthcare, compared to their counterparts who are less educated and may have lower health literacy [14, 15, 20, 28].

The odds of barrier to accessing health care were 1.33 times more likely to occur women delivered at home compared to women who had institutional delivery. This evidence was supported by study done in Senegal[29]. The plausible justification might be women who delivered at home may not get health information/counselling from healthcare providers that empower them to use other maternal healthcare services and women who deliver at home may face varous barriers to accessing health care such as financial constraint, lack of transportation, limited autonomy in healthcare decision and sociocultural norms that discourage institutional delivery. Moreover, importance of enhancing health system outreach, strengthen community-based education, and addressing sociocultural and socioeconomic disparities that affect health seeking behavior. On the other hand, the odds of barrier of accessing health care among woman who had no media exposure were 1.17 times more likely to compared with a woman who had media exposure. This evidence was supported by study done in Ethiopia[30], Nepal[31]. This could be the power of mass media in disseminating information concerning maternal health that may enhance women's knowledge and attitude towards health care access and utilization[31]. Barrier of accessing health care among woman who were not visiting health facility within 12 months were 9 % times less likely compared to women who had visiting health care facility within 12 months of study period. The odds of barrier to accessing health care among woman who had no health insurance utilization were 1.18 times more likely to compared with a woman who had used health insurance. This finding was supported with study done in Ethiopia[1], sub-Saharan Africa[23], Tanzania[15], Ghana[25]. The possible reason might be due to the fact that, individuals who were not had health insurance faced for unexpected catastrophic expenditure and difficulties in obtaining money for consultation of the doctor[1]. The odds of barrier to accessing healthcare among woman who were classified under poor and middle wealth status were 2.29 and 1.57 times more likely accessing health care difficulty compared to women who were classified under rich wealth index category**.** This evidenced was supported with study done in Ethiopia, [1, 30, 32, 33] Tanzania[15] Uganda[34], sub Saharan

Africa[14]. This finding might be explained by the fact that high out-of-pocket expenses and a lack of accessibility to healthcare facilities pose significant obstacles for middle-class and impoverished women in sub-Saharan Africa. Many people in the region cannot afford healthcare services because over 40% of health costs are paid for directly by individuals[35-38]

The odds of barrier of accessing health care among woman who live west Africa region were 2.38 times more likely barriers to accessing health care compared to east Africa region. According a recent joint report from the World Health Organization and the United Nations states that high out-of-pocket costs, a lack of adequate healthcare infrastructure, and other sociocultural barriers make it more difficult for women of reproductive age in West African nations such as Nigeria, Mali, and Sierra Leone to access maternal healthcare. On the other hand, East African countries like Rwanda, Kenya, and Ethiopia have strengthened their community health initiatives and gender-inclusive policies, which have improved women's autonomy in accessing healthcare services[39]. Furthermore, our current findings are supported by recent Demographic and Health Survey (DHS) program data (2022–2023), which indicate that women in West African countries are 2-3 times more likely to report delays in accessing healthcare due to cost, shortages of healthcare personnel, and long distances to health facilities. In contrast, East African countries, particularly Uganda and Tanzania, have strengthened their health insurance systems and rural outreach initiatives. Additionally, the World Bank's 2023 Health Service Coverage Index reveals that women in West Africa have significantly lower access to family planning, skilled birth attendance, and other essential reproductive health services[22, 40, 41]

Strength and limitation

This strength of study nationally representative data to evaluate contextual and individual characteristics related to barriers to healthcare access among reproductive age women in the SSA. Thus, findings can therefore, generalized to all women in their reproductive age in SSA, in order to account for the clusters within the sample, the study also employed advanced statistical models. This study has several limitations. The cross-sectional design restricts the ability to draw causal inferences, and health worker–related factors were not incorporated into the analysis. Furthermore, reliance on self-reported data may introduce recall or social desirability bias; future research should consider integrating objective measures, such as wearable devices, as demonstrated by Zhao et al. (2025)[42]

Conclusion and Recommendation

This study found that 55.55% of reproductive age women had barriers to accessing healthcare in SSA. Husband educational status, media exposure, internet utilization, place of residency, ANC visit status, place of delivery, health insurance utilization status, wealth index status, visiting health facility within 12 months, community literacy level and community wealth index and country category (western, eastern Africa) were significantly associated with barriers of accessing health care. Therefore, the Government and ministry of health in Sub-Saharan Africa countries should taking in to consideration those factors while designing policies and strategies targeting to health care access and tackling the preventable barriers to accessing health care in sub-Saharan Africa.

Declaration

Ethical approval and consent to participate

This study was based on analysis of existing survey datasets in the public domain (SSA) that are freely available online with all the identifier information anonymized, no ethical approval was required

Consent for publication

Not applicable

Data and material availability

Demographic and Health Survey were used, and it is publicly available online at (https://www.dhsprogram.com).

Competing interest

Authors Declared that, there is no competing interest.

Funding

Not applicable

Author's contributions

GAZ: involved in designing, data extraction, data analysis, interpretation, report and manuscript writing.

BT: involved in data analysis, interpretation, and, and manuscript writing.

BT: involved in analysis,

TTT: involved in designing the study, interpretation, report and

BSW: involved in analysis, and result interpretation

AFZ: involved in designing and interpretation study

EGM: analysis and manuscript writing

MW: Result interpretation

AA: manuscript writing: involved in data extraction

MSA: involved manuscript writing

Acknowledgements

We would like to say the DHS programs for allowing us to use the relevant DHS dataset in this study.

Reference

1. Tamirat, K.S., Z.T. Tessema, and F.B.J.B.H.S.R. Kebede, Factors associated with the perceived barriers of health care access among reproductive-age women in Ethiopia: a secondary data analysis of 2016 Ethiopian demographic and health survey. 2020. **20**: p. 1-8.
2. Erasmus, M.O., The barriers to access for maternal health care amongst pregnant adolescents in the Mitchells plain sub-district. 2017.
3. Washington, D.L., et al., Access to care for women veterans: delayed healthcare and unmet need. 2011. **26**: p. 655-661.
4. Regan, L.J.B.M.A., Addressing unmet needs in global women's health. 2018.
5. Izugbara, C., E. Covan, and E. Fugate-Whitlock, Women's health in Africa: Issues, challenges and opportunities. 2017: Routledge.
6. Organization, W.H., Primary health care on the road to universal health coverage: 2019 global monitoring report: Executive summary. 2019, World Health Organization.
7. Badiuzzaman, M., S.M. Murshed, and M.J.T.J.o.D.S. Rieger, Improving maternal health care in a post conflict setting: evidence from Chittagong Hill tracts of Bangladesh. 2020. **56**(2): p. 384-400.
8. Peters, D.H., et al., Poverty and access to health care in developing countries. 2008. **1136**(1): p. 161-171.
9. Alkire, B.C., et al., The economic consequences of mortality amenable to high-quality health care in low-and middle-income countries. 2018. **37**(6): p. 988-996.
10. Nyakang'o, S.B. and A.J.M. Booth, Women's perceived barriers to giving birth in health facilities in rural Kenya: A qualitative evidence synthesis. 2018. **67**: p. 1-11.
11. Colglazier, W.J.S., Sustainable development agenda: 2030. 2015. **349**(6252): p. 1048-1050.

12. Cahill, C.M., et al., Preoperative anemia management program reduces blood transfusion in elective cardiac surgical patients, improving outcomes and decreasing hospital length of stay. 2021. **61**(9): p. 2629-2636.
13. Peeri, N.C., et al., The SARS, MERS and novel coronavirus (COVID-19) epidemics, the newest and biggest global health threats: what lessons have we learned? 2020. **49**(3): p. 717-726.
14. Tessema, Z.T., et al., Determinants of accessing healthcare in Sub-Saharan Africa: a mixed-effect analysis of recent Demographic and Health Surveys from 36 countries. 2022. **12**(1): p. e054397.
15. Bintabara, D., K. Nakamura, and K.J.B.o. Seino, Improving access to healthcare for women in Tanzania by addressing socioeconomic determinants and health insurance: a population-based cross-sectional survey. 2018. **8**(9): p. e023013.
16. Kea, A.Z., et al., Exploring barriers to the use of formal maternal health services and priority areas for action in Sidama zone, southern Ethiopia. 2018. **18**: p. 1-12.
17. Zegeye, B., et al., Breaking barriers to healthcare access: a multilevel analysis of individual-and community-level factors affecting women's access to healthcare services in Benin. 2021. **18**(2): p. 750.
18. Silal, S.P., et al., Exploring inequalities in access to and use of maternal health services in South Africa. 2012. **12**: p. 1-12.
19. Tsawe, M. and A.S.J.B.r.n. Susuman, Determinants of access to and use of maternal health care services in the Eastern Cape, South Africa: a quantitative and qualitative investigation. 2014. **7**: p. 1-10.
20. Zegeye, B., et al., Barriers and facilitators to accessing health care services among married women in Ethiopia: a multi-level analysis of the Ethiopia demographic and health survey. 2021.
21. Demsash, A.W., B. Chakilu, and A. Mazengia, Knowledge Sharing Practice and Its Associated Factors Among Healthcare Providers at University of Gondar Comprehensive Specialized Hospital, North West Ethiopian: Cross-sectional Study. 2021.
22. Tsegaw, M., B. Mulat, and K.J.B.o. Shitu, Problems with accessing healthcare and associated factors among reproductive-aged women in the Gambia using Gambia demographic and Health Survey 2019/2020: a cross-sectional study. 2023. **13**(8): p. e073491.
23. Seidu, A.-A.J.P.O., Mixed effects analysis of factors associated with barriers to accessing healthcare among women in sub-Saharan Africa: Insights from demographic and health surveys. 2020. **15**(11): p. e0241409.
24. Seidu, A.-A., et al., Individual and contextual factors associated with barriers to accessing healthcare among women in Papua New Guinea: insights from a nationwide demographic and health survey. 2021. **13**(6): p. 573-585.
25. Seidu, A.-A., et al., Barriers to accessing healthcare among women in Ghana: a multilevel modelling. 2020. **20**: p. 1-12.
26. Minyihun, A., Z.T.J.R.M. Tessema, and H. Policy, Determinants of access to health care among women in East African countries: a multilevel analysis of recent demographic and health surveys from 2008 to 2017. 2020: p. 1803-1813.
27. Sen, K.K., et al., Do education and living standard matter in breaking barriers to healthcare access among women in Bangladesh? 2023. **23**(1): p. 1431.

28. Dimbuene, Z.T., et al., Women's education and utilization of maternal health services in Africa: a multi-country and socioeconomic status analysis. 2018. **50**(6): p. 725-748.
29. Zegeye, B., et al., Predictors of institutional delivery service utilization among women of reproductive age in Senegal: a population-based study. 2021. **79**: p. 1-11.
30. Demsash, A.W., A.D.J.B.H. Walle, and C. Informatics, Women's health service access and associated factors in Ethiopia: application of geographical information system and multilevel analysis. 2023. **30**(1).
31. Tamang, T.M. Factors associated with completion of continuum of Care for Maternal Health in Nepal. in IUSSP XXVIII International Population Conference, Cape Town, South Africa. 2017.
32. Fentie, E.A., et al., Spatial distribution and determinants of barriers of health care access among female youths in Ethiopia, a mixed effect and spatial analysis. 2023. **13**(1): p. 21517.
33. Alamneh, T.S., et al., Barriers for health care access affects maternal continuum of care utilization in Ethiopia; spatial analysis and generalized estimating equation. 2022. **17**(4): p. e0266490.
34. Kalule-Sabiti, I., A.Y. Amoateng, and M.J.A.P.S. Ngake, The effect of socio-demographic factors on the utilization of maternal health care services in Uganda. 2014: p. 515-525.
35. Shiferaw, S., et al., Why do women prefer home births in Ethiopia? 2013. **13**: p. 1-10.
36. Yaya, S., G. Bishwajit, and V.J.B.g.h. Shah, Wealth, education and urban–rural inequality and maternal healthcare service usage in Malawi. 2016. **1**(2): p. e000085.
37. Voto, T.P., B.E. Voto, and N.J.E. Ngepah, The Impact of Out-of-Pocket Health Expenditure and Public Health Expenditure on Poverty in Sub-Saharan Africa. 2025. **13**(5): p. 134.
38. Alamneh, T.S., et al., Socioeconomic inequality in barriers for accessing health care among married reproductive aged women in sub-Saharan African countries: a decomposition analysis. 2022. **22**(1): p. 130.
39. AKINWALE, A.A.J.I.J.o.I.R. and A. Studies, Gender equity and social progress: empowering women and girls to drive sustainable development in Sub–Saharan Africa. 2023.
40. Arhin, K., E.F. Oteng-Abayie, and J.J.H.e.r. Novignon, Assessing the efficiency of health systems in achieving the universal health coverage goal: evidence from Sub-Saharan Africa. 2023. **13**(1): p. 25.
41. Muhajarine, N., et al., Inequalities and factors associated with maternal healthcare services utilisation in Mozambique: evidence from the Demographic and Health Survey 2022−2023. 2025. **10**(5).
42. Zhao, Y., et al., Highly sensitive, wearable piezoresistive methylcellulose/chitosan@ MXene aerogel sensor array for real-time monitoring of physiological signals of pilots. 2025. **68**(2): p. 542-551.

Figure legend

Fig 1: Barriers to accessing health care and determinant factor among reproductive age women in sub-Saharan Africa

Manuscript

Click here to access/download;Manuscript;Barrier to accessing health careN.docx

Barriers to accessing health care among women's in Sub-Saharan Africa: Multilevel analysis of recent demographic and health survey from 2019-2023.

Gebreeyesus Abera Zeleke[1]*, Enyew Getaneh Mekonen[1], Bewuketu Terefe[2], Mulugeta Wassie[3], Agazhe Aemro[3], Alebachew Ferede Zegeye[3], Mohammed Seid Ali[4], Berhan Tekeba[4], Tadesse Tarik Tamir[4], Belayneh Shetie Workneh[5]

[1] Department of Surgical Nursing, School of Nursing, College of Medicine and Health Sciences, University of Gondar, Gondar, Ethiopia

[2] Department of Community Health Nursing, School of Nursing, College of Medicine and Health Sciences, University of Gondar, Gondar, Ethiopia.

[3] Department of Medical Nursing, School of Nursing, College of Medicine and Health Sciences, University of Gondar, Gondar, Ethiopia;

[4] Department of Pediatrics and Child Health Nursing, School of Nursing, College of Medicine and Health Sciences, University of Gondar, Gondar, Ethiopia.

5 Department of Emergency and Critical Care Nursing, School of Nursing, College of Medicine and Health Sciences, University of Gondar, Gondar, Ethiopia

Correspondence Author*

Gebreeyesus Abera Zeleke

Department of Surgical Nursing, School of Nursing, College of Medicine and Health Science, University of Gondar, Gondar, Ethiopia.

Email address: gebreeyesusabe143@gmail.com

Co-authors:

Enyew Getaneh Mekonen **Email Address:** enyewgetaneh12@gmail.com

Bewuketu Terefe: **Email address**: woldeabwomariam@gmail.com

Mulugeta Wassie: **Email address:** mulugeta2113@gmail.com

Agazhe Aemro: **Email address:** agazhe1049@gmail.com

Alebachew Ferede Zegeye: **Email address:** alexferede24@gmail.com

Mohammed Seid Ali: **Email address:** muheseid2592@gmail.com

Berhan Tekeba: **Email address:** berishboss7@gmail.com

Tadesse Tarik Tamir: **Email address:** tadestar140@gmail.com

Belayneh Shetie Workneh: **Email address:** belay2319@gmail.com

Abstract

Background:

Barriers to accessing healthcare have significantly contributed to the high rates of maternal and child mortality and morbidity in developing regions, particularly in Sub-Saharan Africa. Access to healthcare is shaped by multiple factors, including financial constraints, geographic location, the availability of services, and the quality of care provided. This study aimed to assess the magnitude of these barriers and to identify the factors influencing healthcare access among women of reproductive age in Sub-Saharan Africa.

Methods:

This study utilized secondary data from the most recent Demographic and Health Surveys conducted between 2019 and 2023 across Sub-Saharan Africa. A weighted sample of 134,470 women of reproductive age was included in the analysis. Data were analyzed using Stata version 14, employing a multilevel mixed-effects logistic regression model. Factors associated with barriers to healthcare access were considered statistically significant at P-values less than 0.05. Adjusted odds ratios (AORs) with 95% confidence intervals (CIs) were used to interpret the results.

Result: In this study, the magnitude of barriers to accessing healthcare among reproductive age women were found to be 55.84% (95% CI: 55.57–56.10). Husband's educational status [AOR = 1.23, 95% CI: 1.15–1.30], Media exposure [AOR = 1.17, 95% CI: 1.11–1.24], Antenatal care visit [AOR = 1.31, 95% CI: 1.10–1.55], and place of delivery [AOR = 1.33, 95% CI: 1.24–1.43]. Health insurance utilization [AOR = 1.18, 95% CI: 1.11–1.26], Wealth index, 2.29 [2.14, 2.45], 1.57 [1.46, 1.68] and visiting a health facility within the past 12 months [AOR = 0.91, 95% CI: 0.87–0.97] were individual associated variable. In addition, place of residence [AOR = 1.30, 95% CI: 1.22–1.39], community literacy level [AOR = 1.13, 95% CI: 1.03–1.24], community wealth index [AOR = 1.12, 95% CI: 1.02–1.22], and Region were associated with barriers to accessing healthcare.

Conclusion and recommendation: This study found that 55.55% of women in Sub-Saharan Africa experienced barriers to accessing healthcare. Educational status, media exposure, place of residence, antenatal care attendance, place of delivery, health insurance coverage, wealth index, recent visits to health facilities, community literacy levels, community wealth status, and regional differences were determinant factors. These findings highlight the need for Sub-Saharan African governments to prioritize these determinants when formulating policies and strategies aimed at reducing preventable barriers to healthcare access in the region.

Key words: Access to Healthcare; Barriers to Healthcare; Sub-Saharan Africa; Reproductive Age Women

Introduction

Access to healthcare is broadly defined as the ability to prevent illness, disability, and premature death, to promote and maintain health, and to attain health equity, particularly for women of reproductive age, by offering inclusive, high-quality care and the best possible availability, affordability, and accessibility of medical services[1-3]. Reproductive-age women's health is a critical global public health concern since it influences population health outcomes and fosters socioeconomic growth[4]. The advancement of socioeconomic development is significantly influenced by the health of women of reproductive age, especially in Africa. [5]. The Sustainable Development Goals (SDGs) place a strong emphasis on women's health, with Targets 3.7 and 3.8 of SDG 3 specifically focusing on achieving universal health coverage by 2030 and ensuring access to sexual and reproductive health services, including education and information on family planning[6, 7]. The Sustainable Development Goals (SDGs) explicitly target a substantial reduction in maternal mortality, with Goal 3.1 aiming to lower the global maternal mortality ratio to fewer than 70 per 100,000 live births by 2030. While these global efforts have contributed to some improvements in women's health outcomes, they have yet to fully address the root causes of maternal mortality. Achieving sustained and comprehensive progress remains a critical challenge. In 2016, Sub-Saharan Africa accounted for a disproportionately high share of the 303,000 maternal deaths reported globally, the majority of which were due to preventable causes. Moreover, non-communicable diseases continue to contribute significantly to female morbidity and mortality across nearly all countries, with a particularly severe impact in low- and middle-income settings, where many women are left with long-term disabilities [4-6]. Access to healthcare involves multiple dimensions, including financial affordability, geographic accessibility, availability of medical services, and the quality of care provided in health facilities. [8]. Access to healthcare is a key determinant of whether individuals utilize health services. Globally, approximately 400 million people lack essential healthcare, and around eight million die each year from preventable diseases—resulting in an estimated economic loss of $6 trillion in low- and middle-income countries. [6, 9, 10]. In 2015, world leaders pledged to achieve universal health coverage as part of the Sustainable Development Goals (SDGs), aiming to ensure access to affordable, high-quality medicines and financial protection for all [11, 12]. In 2017, the World Bank and WHO reported that half of the global population lacks access to essential health services. As a result, many countries in Sub-Saharan Africa have made universal healthcare a key part of their national health

strategies. However, there has been slow progress in turning this commitment into tangible results, particularly in securing national funding for equitable, high-quality healthcare, financial security, and overall health [6, 9, 13, 14]. Reproductive age women and child mortality from low family planning uptake, unsafe abortion practices, unintended pregnancies, and an increase in home births are all implications of women in reproductive age having trouble accessing health care[14]. Previous research has offered some evidence regarding the sociodemographic and economic factors that can be barriers to accessing healthcare, such as marital status, place of residence, maternal age, wealth index, educational attainment, and unemployment status. Nevertheless, women of reproductive age continue to face significant disparities in healthcare utilization and access[10, 14-16]. This study makes three unique contributions to the body of existing literature. In order to provide a current and policy-relevant analysis of the barrier to healthcare access, it first makes use of the most recent (2019–2023) Demographic and Health Survey (DHS) data. Second, our analysis takes a harmonized multi-country approach across ten sub-Saharan African nations, improving cross-national comparability and generalizability in contrast to many previous studies that are limited to single-country analyses or rely on older datasets. In order to gain a better understanding of the structural and contextual factors that influence healthcare access, the study employs a comprehensive multilevel analysis of determinants at the individual and community levels. These contributions put our research in a position to guide focused, empirically supported programs provide fair access to healthcare for reproductive age women in sub-Saharan. Therefore, this study aimed to ascertain the magnitude and potential contributing factors associated with barriers to accessing healthcare among women of reproductive age in sub-Saharan Africa.

Methods and materials

Patient and public involvement statement

Secondary data from the Demographic and Health Surveys (DHS) were used in this investigation.

Study design, study area, and period.

Data from ten Sub-Saharan African countries, collected between 2019 and 2023, were used to perform a multilevel mixed-effects analysis, based on the most recent Demographic and Health Surveys (DHS). The DHS collects community-based, cross-sectional data approximately every five years to generate updated health and health-related indicators.

Data source, study population and sampling technique

A secondary data analysis was conducted using Demographic and Health Survey (DHS) data collected between 2019 and 2023 from selected sub-Saharan African countries. The study specifically included DHS datasets from ten countries Burkina Faso, Gabon, Ghana, Kenya, Liberia, Rwanda, Sierra Leone, Tanzania, The Gambia, and Mozambique each of which had recent and publicly available survey data within the specified timeframe. The datasets were appended to assess the extent of, and factors underlying, barriers to healthcare access among women of reproductive age in sub-Saharan Africa. Each country's DHS comprises multiple datasets. The DHS employs a stratified, two-stage cluster sampling design: in the first stage, enumeration areas are selected, followed by systematic sampling of households within each selected area in the second stage.

The variable barrier to accessing health care were generated by computation of (v467b, v467c, v467d, v467f) from maternal recode (IR) data set was recoded. A binary logistic regression model was used to identify determinants a factor barrier to access the health care. Determinants of barrier to accessing the health care were reported in terms of an AOR with a significance level of (95%). In the univariate analysis, at 95% confidence intervals with a p-value of < 0.25 was considered for the candidate multivariable analysis of data. All variables with p values of <0.05 were considered statistically significant. Since DHS data are nationally representative, stratified multistage cluster sampling is required; therefor, sampling weights must be applied to produce unbiased estimates. To correctly weight DHS data, we use the sampling weights provided in the dataset, specifically the individual weight **(**v005**)** for women's individual recode files. This weight must be divided by 1,000,000 (v005/1000000). using "gen wgt=v005/1000000 Stata commands. A total weighted sample of 134,470 reproductive age women were included in the study **(Table 1).**

Table 1: The weighted Sample size for magnitude and determinants of barrier to accessing health care among reproductive age women in sub-Saharan Africa**.**

**Country**	**Year of survey**	**weighted sample (n)**	**weighted sample (%)**
Brukinefaso	2021	17659	13.13
Gabon	2019-2021	6507.15	4.83
Ghana	2022	15014	11.16
Kenya	2022	16716.36	12.43
Liberia	2019-2020	8065	5.99
Rwanda	2019-2020	14634	10.88
Sera lion	2019	15574	11.58
Tanzania	2022	15254	11.34
Gambia	2019-2020	11865	8.08
Mozambique	2023	13181.93	9.80
TOTAL		134470	100%

Study variable

Outcome variables: The outcome of this study was barrier to access the health care. The Data in barrier of accessing the health care were collected among reproductive age women who used one of the health cares. Measurement of the outcome variables was determined by using computation of four variables; such as Distance to health facility (v467b), difficulty in obtaining money (v467c), getting permission go to the doctor (v467d) and not wanting to go alone (v467f). If a woman of reproductive age reported experiencing at least one of the following challenges, such as difficulty in obtaining money, distance to a health facility, lack of permission to seek treatment, or reluctance to go alone were considered to face significant barriers to healthcare access. These cases were coded as '1' and labeled as 'big problem.' Conversely, women who did not report any of these four barriers were considered to have no significant barriers to healthcare access. These were coded as '0' and labeled as 'no big problem'. This approach is consistent with prior literature using DHS data[17]

Independent Variable: Hence DHS data are hierarchical, independent factors come from two sources that was at individual and community levels factors were considered for this analysis. The individual-level independent variables were women's age (15-19, 20-35, 36-49), Women educational status (No education, Primary education , Secondary & Higher education), women occupation (working, not working), Religion (Muslim, Christian , Others), Wealth index (Poor, Middle, Rich) media exposure (yes, No), internet utilization (yes, No), Residency (urban, rural), Husband educational status (No education, Primary education, secondary& higher education), Husband occupational status (No working, Working), Currently Contraceptive (No, Yes) Visiting health facility in 12 month (No, Yes), place of delivery (Home , Institutional delivery), ANC visit (No antenatal visit, 1-4 visit, > 4 visit)

The community-level variables: Poverty level (Low, High), Literacy level (Low, High), Media exposure, (Low, High), Residency (Urban, rural), Region (East, west), Community ANC visit

Data processing and statistical analysis

Stata version 14 statistical software was used to clean, record, and analyze secondary DHS data sets. The variables in the DHS data are grouped into clusters, and the variables within a cluster are more similar to each other than those in other clusters. The presumptions of equal variance

across clusters and independent data were violated in order to run a typical logistic regression model. This suggests that between-cluster factors must be considered using a complex model.

Given this, multilevel mixed-effects logistic regression was used to determine the factors that associated with barrier to accessing the health care. Multilevel mixed effect logistic regression follows four models. The null model (only outcome variable), mode I (individual level variable such as age, Religious status, educational status, Husband educational, marital occupation, Husband occupation, marital status, Contraceptive Utilization, place of delivery, ANC visit, media exposure, Intermate utilization, visiting health facility within 12 months, health insurance , wealth index) model II community level variables, Region in sub-Saharan Africa, community poverty level, community media exposure, Community distance health facility, Community literacy level, Community ANC visit), and model III (both individual and community level variables such as age, Religious status, educational status, Husband educational, marital occupation, Husband Occupation, marital status, Contraceptive Utilization, place pf delivery, ANC visit, media exposure, Intermate utilization, visiting health facility within 12 months, health insurance , wealth index), model II (community level variables, Region in sub-Saharan Africa, community poverty level, community media exposure, Community distance health facility, Community literacy level, Community ANC visit). The null model without independent variables was used to check the variability of outcome variable across the cluster. The relationship of individual-level variables with outcome variable (Model I) and the association of community-level variables with outcome variable (Model II) were determined. In the final model, the association of both individual and community-level variables was fitted with the outcome variable.

Random effects

The intra-class correlation coefficient (ICC), proportionate change in variance (PCV), and median odds ratio (MOR) were used to quantify random effects or measures of variation of the outcome variables.

To assess the variation between clusters, the proportionate change in variance (PCV) and intra-cluster correlation coefficient (ICC) were calculated. By treating the cluster as a random variable, the ICC shows that the variation in the health care access barrier between clusters may be computed as follow $\boldsymbol{ICC} = \frac{\sigma^2 uo}{\sigma^2 uo + \pi^2/3} \times \mathbf{100\%}$. The MOR, which is determined by using clusters as a

random variable and calculating the median value of the odds ratio between the highest-risk and lowest-risk areas for health care access barriers, is calculated. $MOR = \exp(0.6745\ \sqrt{(2 * \sigma^2 uo)})$ Furthermore, the PCV calculates a variable that explains the variation in the degree of barriers to accessing healthcare ; $PCV = \frac{\sigma^2 uo - \sigma^2}{\sigma^2 uo} \times 100\%$;**.** where VC is the cluster level variance and Vnull is the variance of the null model.

The associations between independent factors at the individual and community levels and barriers to accessing health care were estimated using the fixed effects. It was assessed and presented using adjusted odds ratio (AOR) and 95% confidence intervals with a p-value of < 0.05. Because of the nested nature of the model, deviation = -2(log likelihood ratio) was used to compare models, and the model with the lowest deviance was selected as the best-fitted model. By evaluating the variance inflation factors, the model assumption for multi-collinearity was confirmed. In addition, Model comparison was performed using Akaike's Information Criterion (AIC). The model includes both random intercepts and random slopes (Model III) was evaluated alongside alternative models, with AIC values calculated to assess relative model fit and parsimony.

Ethical approval and consent to participate

This study was based on analysis of existing survey datasets in the public domain (SSA) that are freely available online with all the identifier information anonymized, no ethical approval was required

Result

In this study a total of 134,470 reproductive age women were included in Sub-Saharan African. The Mean age of participants were 28.99 (Std. Dev. 9.68) years. More than two thirds (69.8%) of women did not use contraception, and over 72.22% reproductive women did not use health insurance, and over half of (54.22%) women were living rural areas in sub-Saharan Africa **(Table 2)**

Table 2: Sociodemographic and economic characteristics of reproductive women who had barriers to accessing health care in sub-Saharan Africa.

**Variable**	**frequency(n)**	**percentage (%)**
**Individual and community level variables**		

**Maternal age**		
15-19	27890.4	20.7
20-35	69441.4	51.64
36-49	37138.6	27.64
**Residency**		
Urban	61558.03	45.78
Rural	72912.41	54.22
**Religious status**		
Muslim	48834.95	41.96
Christian	46856.90	39.30
Other	23524.59	19.73
**Educational Status**		
No formal education	35051.70	26.06
Primary education	39651.17	29.48
Secondary and higher education	59767.57	44.44
**Husband educational**		
No formal education	29414.44	36.72
Primary education	21245.78	26.52
secondary and higher education	29428.78	36.74
**Maternal Occupational**		
Not working	50732.90	37.81
working	83417.01	62.19
**Husband Occupation**		
Not working	10390.43	13.00
Working	69698.11	87.00
**Maternal status**		
single	42213.40	31.4
Married	58262.00	43.3
Other	33995.4	25.3
**Contraceptive Utilization**		
No	93855.00	69.8
Yes	40615.5	30.2
**place of delivery**		
Home	8279.46	15.5
Institution	44979.28	84.5
ANC visit		
No visiting	2012.84	3.77
1-4 visiting	24459.34	45.92
>4 visiting	26786.55	50.29

Media exposure

No	34628.45	25.75
Yes	99831.57	74.24
**Intermate utilization**		
No	93020.34	69.17
Yes	41450.10	30.82
**Visiting health facility 12 month**		
No	57137.73	42.49
Yes	77332.71	57.50
**Health insurance**		
No	85044.69	72.22
Yes	32709.39	27.77
**Wealth index**		
poor	47227.18	35.12
middle	25771.45	19.16
Rich	61471.81	45.74
**Region in sub-Saharan Africa**		
East Africa	59786.29	44.46
West Africa	74684.16	55.54
**Community poverty level**		
Low	70776.71	52.63
High	63693.73	47.47
**Community media exposure**		
Low	61642.30	45.84
High	72828.14	54.15
**Community distance health facility**		
Low	65962	49.00
High	68508	51.00
**Community literacy level**		
Low	57615.01	42.84
High	76855.43	57.15
**Community ANC visit**		
Low	77912.32	57.96
High	56491.92	42.03

In this study, the magnitude of barriers to accessing health care among reproductive age women was found to be 55.84 (95% CI: (55.57, 56.10) owing to one or more of the four factors, the most commonly cited being the difficulty in getting money and the distance from medical facilities (**Figure 1)**

Random effect (Measures of variation) and model fitness

To determine if the data supported the choice to evaluate randomness at the community level, a null model was employed. With the variance of 0.3784967 and a p-value of < 0.001, the null model's results showed that there were notable variations in the barriers to accessing healthcare among clusters. A significant amount of the variance in the outcome between clusters was jointly explained by both individual- and community-level variables, as evidenced by the intra-cluster correlation (ICC), which dropped from 10.31% in the null model to 3.96% in the fully adjusted model (model III). According to the proportionate change in variance (PCV), Model III explains the greatest percentage (64.09%) of the group-level variation in comparison to the null model, group comparison showed that, Model I (50.19%) and Model II (24.56%). This suggests that the model fits better as more predictors are included. Model comparison using Akaike's Information Criterion (AIC) indicated that Model III achieved the best fit, as evidenced by the lowest AIC (239,608.6), markedly lower than both the null model (AIC = 1,128,437) and Model II (AIC = 1,127,704). Model I also showed good fit (AIC = 239,764.8), though slightly less optimal than Model III. Multicollinearity using variance inflation factors (VIF) indicated that all VIF values ranged from 1.03-1.98, which is within accepted range **(Table 3)**

Table 3: Model comparison and Fitness random effect analysis for barriers of accessing health care of among reproductive age women in sub-Saharan Africa**.**

**Parameter**	**Null model**	**model I**	**model II**	**model III**
Variance	0.3784967	0.1885269	0.28553	0.1358817
ICC	10.31%	5.41%	7.98%	3.96%
MOR	1.79	1.51	1.66	1.41
PCV	Reference	50.19%	24.56%	64.09%
**Model fines**				
LLR	-90175.365	-21798.27	-86841.704	-21433.284
Deviance	1803596.73	43596.54	173683.408	42866.568
AIC	1128437	239764.8	1127704	239608.6

ICC: intra-cluster correlation**, LLR:** loglikelihood ratio**, MOR:** median odds ratio**, PCV:** proportional change in variance. **AIC:** Akaike information criteria.

Determinants of barriers to accessing health care among reproductive age women in Sub-Saharan Africa

Bivariable logistic regression analysis of individual and community level variables associated with barriers to accessing health care among reproductive age women in Sub-Saharan Africa

Bivariable multilevel logistic regression analysis indicated that husband's educational status, media exposure, internet utilization, place of residence, antenatal care attendance, place of delivery, health insurance coverage, community wealth index, recent health facility visits, and country category (Western vs. Eastern Africa) were associated with barriers to accessing health care among reproductive-age women at a p-value < 0.25 (**Table 4**)

Table 4: Bivariable logistic regression of individual and community level factors associated with barriers to accessing health care among reproductive age women in Sub-Saharan Africa.

Variables	Crude Odds Ratio [COR]	P. value	[95% CI]
**Maternal age**			
15-19	0.86	<0.001	[0.83, 0.89]
20-35	0.86	<0.001	[0.84, 0. 88]
36-49	1		1
**Residency**			
Urban	1		1
Rural	2.09	<0.001	[2.04, 2.14]
**Maternal education**			
No education	2.40	<0.001	[2.33, 2.46]
Primary education	1.52	<0.001	[1.48, 1.56]
Secondary & Higher	1		1
**Husband education**			
No education	2.43	<0.001	[2.35, 2.51]
Primary education	1.55	<0.001	[1.49, 1.60]
secondary & higher	1		1
**Maternal occupation**			
working	1		1
not working	0.94	<0.001	[0.92, 0.96]
**Husband occupation**			
working	1		
not working	1.07	<0.001	[1.02, 1.12]
**Religion**			
Muslim	1		1
Christian	1.32	<0.001	[1.29, 1.35]
Other	1.08	<0.001	[1.04, 1.11]

**Contraceptive utilization**			
No	1.18	<0.001	[0.82, 0.86]
Yes	1		1
**Place of delivery**			
Home	1.84	<0.001	[1.75, 1.93]
Institution	1		1
**ANC visit**			
No visit	1.16	<0.002	[1.05, 1.27]
1-4 visit	1.05	<0.003	[1.01, 1.09]
> 4 visits	1		1
**Marital status**			
single	1		
Married	1.23	<0.001	[1.20, 1.26]
Other	1.32	<0.001	[1.29, 1.36]
**Media exposure**			
No	1.90	<0.001	[1.85, 1.95]
Yes	1		1
**Internet utilization**			
No	2.321822	<0.001	[2.26, 2.37]
Yes	1		1
**Visiting health facility 12 month**			
No	1.02	<0.046	[1.00, 1.04]
Yes	1		1
**Health insurance**			
No	1.21	<0.001	[1.18, 1.25]
Yes	1		1
**Wealth index**			
poor	3.58	<0.001	[3.49, 3.67]
middle	2.08	<0.001	[2.02, 2.14]
Rich	1		1
**Region SSA**			
East Africa	1.68	<0.001	[1.65, 1.72]
West Africa	1		1
**community ANC visit**			
Low	0.86	<0.001	[0.85, 0.88]
High	1		1
**community wealth index**			
Low	0.69	<0.001	[0.67, 0.70]
High	1		
**Community media exposure**			
Low	0.73	<0.001	[0.72, 0.75]
High	1		1
**Community literacy level**			
Low	0.77	<0.001	[0.75, 0.79]
High	1		1

Multivariable multilevel logistic regression analysis of individual and community level factors associated with Barriers to health care access among reproductive Age Women in Sub-Saharan Africa.

In the final fitted model of multivariable multilevel logistic regression, Husband educational status, media exposure, place of residency, Antenatal care visit, place of delivery, health insurance utilization, wealth index status, visiting health facility within 12 months, and country category (western, eastern Africa) were significantly associated with barriers of accessing health care among reproductive age women in sub-Saharan Africa. The odds of barrier of accessing health care were 1.30 times higher among reproductive women living in rural residency compared to women living in urban area **[AOR=**1.30, 95 CI: (1.22, 1.39)]. and the odds of barrier of accessing health care were 1.23 times more likely to among reproductive age women whose husband had no education compared to women whose husband had secondary and higher education [**AOR**=1.23, 95 CI: (1.15, 1.30,)]. In addition, odds of barrier of accessing health care were 1.33 times more likely to occur women delivered at home compared to women who had institutional delivery. **(AOR**=1.33, 95% CI: (1.24, 1.43) and the odds of barrier of accessing health care were 1.31 times higher among women who had no history of antenatal visit compared to women who had more than four ANC visit. [**AOR**= 1.31, 95% CI: (1.10, 1.55)]. The odds of the barrier of accessing health care among reproductive age woman who had no media exposure were 1.17 times more likely to compared with a woman who had media exposure **[AOR**=1.17, 95% CI: (1.11, 1.24)] and the barrier of accessing health care among reproductive age woman who were not visiting health facility within 12 months 9% times less likely compared to women who had visiting health care facility within 12 months of study period **[AOR=**0.91, 95% CI: (0.87, 0.97)]. The odds of perceived barrier of accessing health care among reproductive age woman who had no health insurance utilization were 1.18 times more likely to compared with a woman who had used health insurance [**AOR=**1.18 95% CI: (1.11, 1.26)] and The odds of perceived barrier of accessing health care service among reproductive age woman who were classified under poor and middle wealth status were 2.29 and 1.57 times more likely barries accessing health care compared to reproductive women who were classified under rich wealth status category **[AOR**=2.29 95% CI: (2.14, 2.45)] and [**AOR**=1.57, 95% CI: (1.46, 1.68)]. The odds of perceived barrier of accessing health care service among reproductive age woman who reside in west Africa region were 2.38 times more

likely barriers accessing health care compared to east Africa region category. [**AOR**=2.38, 95% CI: (2.21, 2.55)] **(Table 5).**

Table 5: Multivariable multilevel logistic regression of individual and community level factors associated with barriers to accessing health care among reproductive age women in Sub-Saharan Africa.

**Individual and community level factors**	**Model I AOR [95% CI]**	**Model II AOR [95% CI]**	**Model III AOR [95% CI]**
**Maternal age**			
15-19	0.96 [0.86, 1.07]		0.96 [0.86,1.08]
20-35	1.06 [1.01,1.13]		1.04 [0.99, 1.11]
36-45	1		1
**Residency**			
Urban		1	1
Rural		2.29 [2.24, 2.35]	**1.30 [1.22, 1.39]**
**Maternal education**			
No education	1.10 [1.02, 1.18]		1.04 [0.97, 1.11]
Primary education	0.93 [0.87, 0.99]		1.06 [0.99, 1.14]
Secondary and above	1		1
**Husband education**			
No education	1.26 [1.19,1.35]		**1.23 [1.15, 1.30]**
Primary education	1.04 [0.97,1.12]		1.23 [0.67, 1.22]
secondary & higher education	1		1
**Maternal occupation**			
working	1		1
not working	0.99 [0.94, 1.04]		**1.02 [1.01, 1.11]**
**Husband occupation**			
working	1		1
not working	0.90 [0.83, 0.97]		1.00 [0.92, 1.08]
**Religion**			
Muslim	1		1
Christian	1.62 [1.53, 1.71]		0.46 [0.57, 1.94]
Other	1.22 [1.13, 1.31]		1.36 [0.75, 1.46]
**Contraceptive utilization**			
No	1.11 [1.05,1.17]		1.03 [0.98, 1.08]
Yes	1		1
**Place of delivery**			
home	1.30 [1.21, 1.40]		**1.33 [1.24, 1.43]**
Institution	1		**1**

**Antenatal Care Visit**			
No	1.01 [0.85, 1.19]		**1.31 [1.10, 1.55]**
1-4 visit	0.94 [0.89, 0.99]		1.13 [0.37, 1.19]
> 4 visits	1		1
**Media exposure**			
No	1.14 [1.08, 1.21]		**1.17 [1.11, 1.24]**
Yes	1		1
**Internet utilization**			
No	1.69 [1.58, 1.80]		1.68 [0.67, 1.79]
Yes	1		1
**Visiting health facility 12 month**			
No	0.92 [0.87, 0.98]		**0.91 [0.87, 0.97]**
Yes	1		1
**Health insurance**			
No	1.26 [1.19, 1.34]		**1.18 [1.11, 1.26]**
Yes	1		1
**Wealth index**			
poor	2.80 [2.63, 2.97]		**2.29 [2.14, 2.45]**
middle	1.79 [1.68, 1.92]		**1.57 [1.46, 1.68]**
Rich	1		1
**Region SSA**			
East Africa		1	**1**
West Africa		2.0 [1.92-2.04]	**2.38 [2.21, 2.55]**
**community ANC visit**			
Low		1.00 [0.93, 1.09]	0.93 [0.86, 1.00]
High		1	1
**community wealth index**			
Low		0.75 [0.70, 0.81]	**1.12 [1.02, 1.22]**
High		1	1
**Community media exposure**			
Low		1.01 [0.92, 1.10]	0.94 [0.86, 1.03]
High		1	1
**Community literacy level**			
Low		1.10 [1.00, 1.20]	**1.13 [1.03, 1.24]**
High		1	**1**

Model I: *individual level variable,* **Model II:** *Community level variable,* **Model III**: *Individual and community level variable*

Discussion

Barriers to accessing health care have contributed to maternal and child mortality and morbidity in developing countries such as those in Sub-Saharan Africa. This study aimed to assess the

magnitude and determinants of barriers to health care access among women of reproductive age in Sub-Saharan Africa.

In this study, the magnitude of barriers to accessing health care among reproductive age women was found to be 55.84 (95% CI: (55.57, 56.10) owing to one or more of the four factors, the most commonly cited being the difficulty in getting money and the distance from medical facilities. This finding is lower than the previous study conducted in South Africa 65% [18, 19], and Tanzania 65% [15], Ethiopia 71.8%,70.2, 69.9%[1, 20, 21], (SSA) 60.4%, 61.5% [17]. this variation could be due to the fact that socio-economic and variability in health infrastructure and health system policy, women's attitudes towards health care seeking behavior and cultural differences across countries in sub-Saharan Africa, moreover, this discrepancy might be due to study period, this study includes, the most recent DHS data set from 2019-2023. On the other hand, the finding of this study is similar to but slightly higher than in previous study in Gambia 45.5%[22], Ghana (51%)[23]. this variation could be due to the fact that socio-economic and variability in health infrastructure and health system policy, women's attitudes towards health care seeking behavior and cultural differences across countries in sub-Saharan Africa.

The odds of barrier of accessing health care were 1.30 times higher among reproductive women living in rural residency compared to women living in urban area. This finding is supported by previous studies conducted in Ethiopia [1, 20], guinea[24], Gambia, Tanzania and Ghana [15, 22, 25] respectively. Sub-Saharan[23], East Africa[26]. This association may be, due to the fact that rural areas have low health infrastructure i.e. inaccessibility of health facilities, less privileged and the influences of socio-cultural practices that need women to seek permission from their husband to visit the facility or women's decisions for seeking healthcare services is under their husband/partners control. Moreover, male involvement and support for women health care access are limited[1, 14, 23, 26]. This implies that the government should expand infrastructure services in rural area and encourage male involvement and support for improving health care access for reproductive age women[14].

The odds of perceived barrier of accessing health care were 1.23 and 1.21 times more likely to among reproductive age women whose husband had no education and primary education compared to women whose husband had secondary and above educational level respectively. This find was consistent with previous study conducted in east Africa, Ethiopia, Tanzania, Benin,

Bangladesh respectively [1, 15, 20, 26, 27]. The possible explanation could be in better access to the healthcare among women with educated husbands might be due to better participation and involvement of husbands in their families' health[23]. Women whose partners are educated are also likely to be informed regarding their fundamental human rights and may have higher health literacy. As a result, they are more likely to deal with any form of barrier to healthcare, compared to their counterparts who are less educated and may have lower health literacy [14, 15, 20, 28].

The odds of barrier to accessing health care were 1.33 times more likely to occur women delivered at home compared to women who had institutional delivery. This evidence was supported by study done in Senegal[29]. The plausible justification might be women who delivered at home may not get health information/counselling from healthcare providers that empower them to use other maternal healthcare services and women who deliver at home may face varous barriers to accessing health care such as financial constraint, lack of transportation, limited autonomy in healthcare decision and sociocultural norms that discourage institutional delivery. Moreover, importance of enhancing health system outreach, strengthen community-based education, and addressing sociocultural and socioeconomic disparities that affect health seeking behavior. On the other hand, the odds of barrier of accessing health care among woman who had no media exposure were 1.17 times more likely to compared with a woman who had media exposure. This evidence was supported by study done in Ethiopia[30], Nepal[31]. This could be the power of mass media in disseminating information concerning maternal health that may enhance women's knowledge and attitude towards health care access and utilization[31]. Barrier of accessing health care among woman who were not visiting health facility within 12 months were 9 % times less likely compared to women who had visiting health care facility within 12 months of study period. The odds of barrier to accessing health care among woman who had no health insurance utilization were 1.18 times more likely to compared with a woman who had used health insurance. This finding was supported with study done in Ethiopia[1], sub-Saharan Africa[23], Tanzania[15], Ghana[25]. The possible reason might be due to the fact that, individuals who were not had health insurance faced for unexpected catastrophic expenditure and difficulties in obtaining money for consultation of the doctor[1]. The odds of barrier to accessing healthcare among woman who were classified under poor and middle wealth status were 2.29 and 1.57 times more likely accessing health care difficulty compared to women who were classified under rich wealth index category**.** This evidenced was supported with study done in Ethiopia, [1, 30, 32, 33] Tanzania[15] Uganda[34], sub Saharan

Africa[14]. This finding might be explained by the fact that high out-of-pocket expenses and a lack of accessibility to healthcare facilities pose significant obstacles for middle-class and impoverished women in sub-Saharan Africa. Many people in the region cannot afford healthcare services because over 40% of health costs are paid for directly by individuals[35-38]

The odds of barrier of accessing health care among woman who live west Africa region were 2.38 times more likely barriers to accessing health care compared to east Africa region. According a recent joint report from the World Health Organization and the United Nations states that high out-of-pocket costs, a lack of adequate healthcare infrastructure, and other sociocultural barriers make it more difficult for women of reproductive age in West African nations such as Nigeria, Mali, and Sierra Leone to access maternal healthcare. On the other hand, East African countries like Rwanda, Kenya, and Ethiopia have strengthened their community health initiatives and gender-inclusive policies, which have improved women's autonomy in accessing healthcare services[39]. Furthermore, our current findings are supported by recent Demographic and Health Survey (DHS) program data (2022–2023), which indicate that women in West African countries are 2-3 times more likely to report delays in accessing healthcare due to cost, shortages of healthcare personnel, and long distances to health facilities. In contrast, East African countries, particularly Uganda and Tanzania, have strengthened their health insurance systems and rural outreach initiatives. Additionally, the World Bank's 2023 Health Service Coverage Index reveals that women in West Africa have significantly lower access to family planning, skilled birth attendance, and other essential reproductive health services[22, 40, 41]

Strength and limitation

This strength of study nationally representative data to evaluate contextual and individual characteristics related to barriers to healthcare access among reproductive age women in the SSA. Thus, findings can therefore, generalized to all women in their reproductive age in SSA, in order to account for the clusters within the sample, the study also employed advanced statistical models. This study has several limitations. The cross-sectional design restricts the ability to draw causal inferences, and health worker–related factors were not incorporated into the analysis. Furthermore, reliance on self-reported data may introduce recall or social desirability bias; future research should consider integrating objective measures, such as wearable devices, as demonstrated by Zhao et al. (2025)[42]

Conclusion and Recommendation

This study found that 55.55% of reproductive age women had barriers to accessing healthcare in SSA. Husband educational status, media exposure, internet utilization, place of residency, ANC visit status, place of delivery, health insurance utilization status, wealth index status, visiting health facility within 12 months, community literacy level and community wealth index and country category (western, eastern Africa) were significantly associated with barriers of accessing health care. Therefore, the Government and ministry of health in Sub-Saharan Africa countries should taking in to consideration those factors while designing policies and strategies targeting to health care access and tackling the preventable barriers to accessing health care in sub-Saharan Africa.

Declaration

Ethical approval and consent to participate

This study was based on analysis of existing survey datasets in the public domain (SSA) that are freely available online with all the identifier information anonymized, no ethical approval was required

Consent for publication

Not applicable

Data and material availability

Demographic and Health Survey were used, and it is publicly available online at (https://www.dhsprogram.com).

Competing interest

Authors Declared that, there is no competing interest.

Funding

Not applicable

Author's contributions

GAZ: involved in designing, data extraction, data analysis, interpretation, report and manuscript writing.

BT: involved in data analysis, interpretation, and, and manuscript writing.

BT: involved in analysis,

TTT: involved in designing the study, interpretation, report and

BSW: involved in analysis, and result interpretation

AFZ: involved in designing and interpretation study

EGM: analysis and manuscript writing

MW: Result interpretation

AA: manuscript writing: involved in data extraction

MSA: involved manuscript writing

Acknowledgements

We would like to say the DHS programs for allowing us to use the relevant DHS dataset in this study.

Reference

1. Tamirat, K.S., Z.T. Tessema, and F.B.J.B.H.S.R. Kebede, Factors associated with the perceived barriers of health care access among reproductive-age women in Ethiopia: a secondary data analysis of 2016 Ethiopian demographic and health survey. 2020. **20**: p. 1-8.
2. Erasmus, M.O., The barriers to access for maternal health care amongst pregnant adolescents in the Mitchells plain sub-district. 2017.
3. Washington, D.L., et al., Access to care for women veterans: delayed healthcare and unmet need. 2011. **26**: p. 655-661.
4. Regan, L.J.B.M.A., Addressing unmet needs in global women's health. 2018.
5. Izugbara, C., E. Covan, and E. Fugate-Whitlock, Women's health in Africa: Issues, challenges and opportunities. 2017: Routledge.
6. Organization, W.H., Primary health care on the road to universal health coverage: 2019 global monitoring report: Executive summary. 2019, World Health Organization.
7. Badiuzzaman, M., S.M. Murshed, and M.J.T.J.o.D.S. Rieger, Improving maternal health care in a post conflict setting: evidence from Chittagong Hill tracts of Bangladesh. 2020. **56**(2): p. 384-400.
8. Peters, D.H., et al., Poverty and access to health care in developing countries. 2008. **1136**(1): p. 161-171.
9. Alkire, B.C., et al., The economic consequences of mortality amenable to high-quality health care in low-and middle-income countries. 2018. **37**(6): p. 988-996.
10. Nyakang'o, S.B. and A.J.M. Booth, Women's perceived barriers to giving birth in health facilities in rural Kenya: A qualitative evidence synthesis. 2018. **67**: p. 1-11.
11. Colglazier, W.J.S., Sustainable development agenda: 2030. 2015. **349**(6252): p. 1048-1050.

12. Cahill, C.M., et al., Preoperative anemia management program reduces blood transfusion in elective cardiac surgical patients, improving outcomes and decreasing hospital length of stay. 2021. **61**(9): p. 2629-2636.
13. Peeri, N.C., et al., The SARS, MERS and novel coronavirus (COVID-19) epidemics, the newest and biggest global health threats: what lessons have we learned? 2020. **49**(3): p. 717-726.
14. Tessema, Z.T., et al., Determinants of accessing healthcare in Sub-Saharan Africa: a mixed-effect analysis of recent Demographic and Health Surveys from 36 countries. 2022. **12**(1): p. e054397.
15. Bintabara, D., K. Nakamura, and K.J.B.o. Seino, Improving access to healthcare for women in Tanzania by addressing socioeconomic determinants and health insurance: a population-based cross-sectional survey. 2018. **8**(9): p. e023013.
16. Kea, A.Z., et al., Exploring barriers to the use of formal maternal health services and priority areas for action in Sidama zone, southern Ethiopia. 2018. **18**: p. 1-12.
17. Zegeye, B., et al., Breaking barriers to healthcare access: a multilevel analysis of individual-and community-level factors affecting women's access to healthcare services in Benin. 2021. **18**(2): p. 750.
18. Silal, S.P., et al., Exploring inequalities in access to and use of maternal health services in South Africa. 2012. **12**: p. 1-12.
19. Tsawe, M. and A.S.J.B.r.n. Susuman, Determinants of access to and use of maternal health care services in the Eastern Cape, South Africa: a quantitative and qualitative investigation. 2014. **7**: p. 1-10.
20. Zegeye, B., et al., Barriers and facilitators to accessing health care services among married women in Ethiopia: a multi-level analysis of the Ethiopia demographic and health survey. 2021.
21. Demsash, A.W., B. Chakilu, and A. Mazengia, Knowledge Sharing Practice and Its Associated Factors Among Healthcare Providers at University of Gondar Comprehensive Specialized Hospital, North West Ethiopian: Cross-sectional Study. 2021.
22. Tsegaw, M., B. Mulat, and K.J.B.o. Shitu, Problems with accessing healthcare and associated factors among reproductive-aged women in the Gambia using Gambia demographic and Health Survey 2019/2020: a cross-sectional study. 2023. **13**(8): p. e073491.
23. Seidu, A.-A.J.P.O., Mixed effects analysis of factors associated with barriers to accessing healthcare among women in sub-Saharan Africa: Insights from demographic and health surveys. 2020. **15**(11): p. e0241409.
24. Seidu, A.-A., et al., Individual and contextual factors associated with barriers to accessing healthcare among women in Papua New Guinea: insights from a nationwide demographic and health survey. 2021. **13**(6): p. 573-585.
25. Seidu, A.-A., et al., Barriers to accessing healthcare among women in Ghana: a multilevel modelling. 2020. **20**: p. 1-12.
26. Minyihun, A., Z.T.J.R.M. Tessema, and H. Policy, Determinants of access to health care among women in East African countries: a multilevel analysis of recent demographic and health surveys from 2008 to 2017. 2020: p. 1803-1813.
27. Sen, K.K., et al., Do education and living standard matter in breaking barriers to healthcare access among women in Bangladesh? 2023. **23**(1): p. 1431.

28. Dimbuene, Z.T., et al., Women's education and utilization of maternal health services in Africa: a multi-country and socioeconomic status analysis. 2018. **50**(6): p. 725-748.
29. Zegeye, B., et al., Predictors of institutional delivery service utilization among women of reproductive age in Senegal: a population-based study. 2021. **79**: p. 1-11.
30. Demsash, A.W., A.D.J.B.H. Walle, and C. Informatics, Women's health service access and associated factors in Ethiopia: application of geographical information system and multilevel analysis. 2023. **30**(1).
31. Tamang, T.M. Factors associated with completion of continuum of Care for Maternal Health in Nepal. in IUSSP XXVIII International Population Conference, Cape Town, South Africa. 2017.
32. Fentie, E.A., et al., Spatial distribution and determinants of barriers of health care access among female youths in Ethiopia, a mixed effect and spatial analysis. 2023. **13**(1): p. 21517.
33. Alamneh, T.S., et al., Barriers for health care access affects maternal continuum of care utilization in Ethiopia; spatial analysis and generalized estimating equation. 2022. **17**(4): p. e0266490.
34. Kalule-Sabiti, I., A.Y. Amoateng, and M.J.A.P.S. Ngake, The effect of socio-demographic factors on the utilization of maternal health care services in Uganda. 2014: p. 515-525.
35. Shiferaw, S., et al., Why do women prefer home births in Ethiopia? 2013. **13**: p. 1-10.
36. Yaya, S., G. Bishwajit, and V.J.B.g.h. Shah, Wealth, education and urban–rural inequality and maternal healthcare service usage in Malawi. 2016. **1**(2): p. e000085.
37. Voto, T.P., B.E. Voto, and N.J.E. Ngepah, The Impact of Out-of-Pocket Health Expenditure and Public Health Expenditure on Poverty in Sub-Saharan Africa. 2025. **13**(5): p. 134.
38. Alamneh, T.S., et al., Socioeconomic inequality in barriers for accessing health care among married reproductive aged women in sub-Saharan African countries: a decomposition analysis. 2022. **22**(1): p. 130.
39. AKINWALE, A.A.J.I.J.o.I.R. and A. Studies, Gender equity and social progress: empowering women and girls to drive sustainable development in Sub–Saharan Africa. 2023.
40. Arhin, K., E.F. Oteng-Abayie, and J.J.H.e.r. Novignon, Assessing the efficiency of health systems in achieving the universal health coverage goal: evidence from Sub-Saharan Africa. 2023. **13**(1): p. 25.
41. Muhajarine, N., et al., Inequalities and factors associated with maternal healthcare services utilisation in Mozambique: evidence from the Demographic and Health Survey 2022−2023. 2025. **10**(5).
42. Zhao, Y., et al., Highly sensitive, wearable piezoresistive methylcellulose/chitosan@ MXene aerogel sensor array for real-time monitoring of physiological signals of pilots. 2025. **68**(2): p. 542-551.

Figure legend

Fig 1: Barriers to accessing health care and determinant factor among reproductive age women in sub-Saharan Africa

Manuscript Click here to access/download;Manuscript;BAHC NEW.docx

Barriers to accessing health care among women in Sub-Saharan Africa: Multilevel analysis of recent demographic and health survey from 2019-2023.

Gebreeyesus Abera Zeleke[1]*, Enyew Getaneh Mekonen[1], Bewuketu Terefe[2], Mulugeta Wassie[3], Agazhe Aemro[3], Alebachew Ferede Zegeye[3], Mohammed Seid Ali[4], Berhan Tekeba[4], Tadesse Tarik Tamir[4], Belayneh Shetie Workneh[5]

[1] Department of Surgical Nursing, School of Nursing, College of Medicine and Health Sciences, University of Gondar, Gondar, Ethiopia

[2] Department of Community Health Nursing, School of Nursing, College of Medicine and Health Sciences, University of Gondar, Gondar, Ethiopia.

[3] Department of Medical Nursing, School of Nursing, College of Medicine and Health Sciences, University of Gondar, Gondar, Ethiopia;

[4] Department of Pediatrics and Child Health Nursing, School of Nursing, College of Medicine and Health Sciences, University of Gondar, Gondar, Ethiopia.

5 Department of Emergency and Critical Care Nursing, School of Nursing, College of Medicine and Health Sciences, University of Gondar, Gondar, Ethiopia

Correspondence Author*
Gebreeyesus Abera Zeleke

Department of Surgical Nursing, School of Nursing, College of Medicine and Health Science, University of Gondar, Gondar, Ethiopia.

Email address: gebreeyesusabe143@gmail.com

Co-authors:

Enyew Getaneh Mekonen **Email Address:** enyewgetaneh12@gmail.com

Bewuketu Terefe: **Email address**: woldeabwomariam@gmail.com

Mulugeta Wassie: **Email address:** mulugeta2113@gmail.com

Agazhe Aemro: **Email address:** agazhe1049@gmail.com

Alebachew Ferede Zegeye: **Email address:** alexferede24@gmail.com

Mohammed Seid Ali: **Email address:** muheseid2592@gmail.com

Berhan Tekeba: **Email address:** berishboss7@gmail.com

Tadesse Tarik Tamir: **Email address:** tadestar140@gmail.com
Belayneh Shetie Workneh: **Email address:** belay2319@gmail.com

Abstract

Background:

Barriers to accessing healthcare have significantly contributed to the high rates of maternal and child mortality and morbidity in developing regions, particularly in Sub-Saharan Africa. Access to healthcare is shaped by multiple factors, including financial constraints, geographic location, the availability of services, and the quality of care provided. This study aimed to assess the magnitude of these barriers and to identify the factors influencing healthcare access among women of reproductive age in Sub-Saharan Africa.

Methods:

This study utilized secondary data from the most recent Demographic and Health Surveys conducted between 2019 and 2023 across Sub-Saharan Africa. A weighted sample of 134,470 women of reproductive age was included in the analysis. Data were analyzed using Stata version 14, employing a multilevel mixed-effects logistic regression model. Factors associated with barriers to healthcare access were considered statistically significant at P-values less than 0.05. Adjusted odds ratios (AORs) with 95% confidence intervals (CIs) were used to interpret the results.

Result: In this study, the magnitude of barriers to accessing healthcare among reproductive age women were found to be 55.84% (95% CI: 55.57–56.10). Husband's educational status [AOR = 1.23, 95% CI: 1.15–1.30], Media exposure [AOR = 1.17, 95% CI: 1.11–1.24], Antenatal care visit [AOR = 1.31, 95% CI: 1.10–1.55], and place of delivery [AOR = 1.33, 95% CI: 1.24–1.43]. Health insurance utilization [AOR = 1.18, 95% CI: 1.11–1.26], Wealth index, 2.29 [2.14, 2.45], 1.57 [1.46, 1.68] and visiting a health facility within the past 12 months [AOR = 0.91, 95% CI: 0.87–0.97] were individual associated variable. In addition, place of residence [AOR = 1.30, 95% CI: 1.22–1.39], community literacy level [AOR = 1.13, 95% CI: 1.03–1.24], community wealth index [AOR = 1.12, 95% CI: 1.02–1.22], and Region were associated with barriers to accessing healthcare.

Conclusion and recommendation: This study found that 55.55% of women in Sub-Saharan Africa experienced barriers to accessing healthcare. Educational status, media exposure, place of residence, antenatal care attendance, place of delivery, health insurance coverage, wealth index, recent visits to health facilities, community literacy levels, community wealth status, and regional differences were determinant factors. These findings highlight the need for Sub-Saharan African governments to prioritize these determinants when formulating policies and strategies aimed at reducing preventable barriers to healthcare access in the region.

Key words: Access to Healthcare; Barriers to Healthcare; Sub-Saharan Africa; Reproductive Age Women

Introduction

Access to healthcare is broadly defined as the ability to prevent illness, disability, and premature death, to promote and maintain health, and to attain health equity, particularly for women of reproductive age, by offering inclusive, high-quality care and the best possible availability, affordability, and accessibility of medical services[1-3]. Reproductive-age women's health is a critical global public health concern since it influences population health outcomes and fosters socioeconomic growth[4]. The advancement of socioeconomic development is significantly influenced by the health of women of reproductive age, especially in Africa. [5]. The Sustainable Development Goals (SDGs) place a strong emphasis on women's health, with Targets 3.7 and 3.8 of SDG 3 specifically focusing on achieving universal health coverage by 2030 and ensuring access to sexual and reproductive health services, including education and information on family planning[6, 7]. The Sustainable Development Goals (SDGs) explicitly target a substantial reduction in maternal mortality, with Goal 3.1 aiming to lower the global maternal mortality ratio to fewer than 70 per 100,000 live births by 2030. While these global efforts have contributed to some improvements in women's health outcomes, they have yet to fully address the root causes of maternal mortality. Achieving sustained and comprehensive progress remains a critical challenge. In 2016, Sub-Saharan Africa accounted for a disproportionately high share of the 303,000 maternal deaths reported globally, the majority of which were due to preventable causes. Moreover, non-communicable diseases continue to contribute significantly to female morbidity and mortality across nearly all countries, with a particularly severe impact in low- and middle-income settings, where many women are left with long-term disabilities [4-6]. Access to healthcare involves multiple dimensions, including financial affordability, geographic accessibility, availability of medical services, and the quality of care provided in health facilities. [8]. Access to healthcare is a key determinant of whether individuals utilize health services. Globally, approximately 400 million people lack essential healthcare, and around eight million die each year from preventable diseases—resulting in an estimated economic loss of $6 trillion in low- and middle-income countries. [6, 9, 10]. In 2015, world leaders pledged to achieve universal health coverage as part of the Sustainable Development Goals (SDGs), aiming to ensure access to affordable, high-quality medicines and financial protection for all [11, 12]. In 2017, the World Bank and WHO reported that half of the global population lacks access to essential health services. As a result, many countries in Sub-Saharan Africa have made universal healthcare a key part of their national health

strategies. However, there has been slow progress in turning this commitment into tangible results, particularly in securing national funding for equitable, high-quality healthcare, financial security, and overall health [6, 9, 13, 14]. Reproductive age women and child mortality from low family planning uptake, unsafe abortion practices, unintended pregnancies, and an increase in home births are all implications of women in reproductive age having trouble accessing health care[14]. Previous research has offered some evidence regarding the sociodemographic and economic factors that can be barriers to accessing healthcare, such as marital status, place of residence, maternal age, wealth index, educational attainment, and unemployment status. Nevertheless, women of reproductive age continue to face significant disparities in healthcare utilization and access[10, 14-16]. This study makes three unique contributions to the body of existing literature. In order to provide a current and policy-relevant analysis of the barrier to healthcare access, it first makes use of the most recent (2019–2023) Demographic and Health Survey (DHS) data. Second, our analysis takes a harmonized multi-country approach across ten sub-Saharan African nations, improving cross-national comparability and generalizability in contrast to many previous studies that are limited to single-country analyses or rely on older datasets. In order to gain a better understanding of the structural and contextual factors that influence healthcare access, the study employs a comprehensive multilevel analysis of determinants at the individual and community levels. These contributions put our research in a position to guide focused, empirically supported programs provide fair access to healthcare for reproductive age women in sub-Saharan. Therefore, this study aimed to ascertain the magnitude and potential contributing factors associated with barriers to accessing healthcare among women of reproductive age in sub-Saharan Africa.

Methods and materials

Patient and public involvement statement

Secondary data from the Demographic and Health Surveys (DHS) were used in this investigation.

Study design, study area, and period.

Data from ten Sub-Saharan African countries, collected between 2019 and 2023, were used to perform a multilevel mixed-effects analysis, based on the most recent Demographic and Health Surveys (DHS). The DHS collects community-based, cross-sectional data approximately every five years to generate updated health and health-related indicators.

Data source, study population and sampling technique

A secondary data analysis was conducted using Demographic and Health Survey (DHS) data collected between 2019 and 2023 from selected sub-Saharan African countries. The study specifically included DHS datasets from ten countries Burkina Faso, Gabon, Ghana, Kenya, Liberia, Rwanda, Sierra Leone, Tanzania, The Gambia, and Mozambique each of which had recent and publicly available survey data within the specified timeframe. The datasets were appended to assess the extent of, and factors underlying, barriers to healthcare access among women of reproductive age in sub-Saharan Africa. Each country's DHS comprises multiple datasets. The DHS employs a stratified, two-stage cluster sampling design: in the first stage, enumeration areas are selected, followed by systematic sampling of households within each selected area in the second stage.

The variable barrier to accessing health care were generated by computation of (v467b, v467c, v467d, v467f) from maternal recode (IR) data set was recoded. A binary logistic regression model was used to identify determinants a factor barrier to access the health care. Determinants of barrier to accessing the health care were reported in terms of an AOR with a significance level of (95%). In the univariate analysis, at 95% confidence intervals with a p-value of < 0.25 was considered for the candidate multivariable analysis of data. All variables with p values of <0.05 were considered statistically significant. Since DHS data are nationally representative, stratified multistage cluster sampling is required; therefor, sampling weights must be applied to produce unbiased estimates. To correctly weight DHS data, we use the sampling weights provided in the dataset, specifically the individual weight **(**v005**)** for women's individual recode files. This weight must be divided by 1,000,000 (v005/1000000). using "gen wgt=v005/1000000 Stata commands. A total weighted sample of 134,470 reproductive age women were included in the study **(Table 1).**

Table 1: The weighted Sample size for magnitude and determinants of barrier to accessing health care among reproductive age women in sub-Saharan Africa**.**

Country	Year of survey	weighted sample (n)	weighted sample (%)
Brukinefaso	2021	17659	13.13
Gabon	2019-2021	6507.15	4.83
Ghana	2022	15014	11.16
Kenya	2022	16716.36	12.43
Liberia	2019-2020	8065	5.99
Rwanda	2019-2020	14634	10.88
Sera lion	2019	15574	11.58
Tanzania	2022	15254	11.34
Gambia	2019-2020	11865	8.08
Mozambique	2023	13181.93	9.80
TOTAL		134470	100%

Study variable

Outcome variables: The outcome of this study was barrier to access the health care. The Data in barrier of accessing the health care were collected among reproductive age women who used one of the health cares. Measurement of the outcome variables was determined by using computation of four variables; such as Distance to health facility (v467b), difficulty in obtaining money (v467c), getting permission go to the doctor (v467d) and not wanting to go alone (v467f). If a woman of reproductive age reported experiencing at least one of the following challenges, such as difficulty in obtaining money, distance to a health facility, lack of permission to seek treatment, or reluctance to go alone were considered to face significant barriers to healthcare access. These cases were coded as '1' and labeled as 'big problem.' Conversely, women who did not report any of these four barriers were considered to have no significant barriers to healthcare access. These were coded as '0' and labeled as 'no big problem'. This approach is consistent with prior literature using DHS data[17]

Independent Variable: Hence DHS data are hierarchical, independent factors come from two sources that was at individual and community levels factors were considered for this analysis. The individual-level independent variables were women's age (15-19, 20-35, 36-49), Women educational status (No education, Primary education , Secondary & Higher education), women occupation (working, not working), Religion (Muslim, Christian , Others), Wealth index (Poor, Middle, Rich) media exposure (yes, No), internet utilization (yes, No), Residency (urban, rural), Husband educational status (No education, Primary education, secondary& higher education), Husband occupational status (No working, Working), Currently Contraceptive (No, Yes) Visiting health facility in 12 month (No, Yes), place of delivery (Home , Institutional delivery), ANC visit (No antenatal visit, 1-4 visit, > 4 visit)

The community-level variables: Poverty level (Low, High), Literacy level (Low, High), Media exposure, (Low, High), Residency (Urban, rural), Region (East, west), Community ANC visit

Data processing and statistical analysis

Stata version 14 statistical software was used to clean, record, and analyze secondary DHS data sets. The variables in the DHS data are grouped into clusters, and the variables within a cluster are more similar to each other than those in other clusters. The presumptions of equal variance

across clusters and independent data were violated in order to run a typical logistic regression model. This suggests that between-cluster factors must be considered using a complex model.

Given this, multilevel mixed-effects logistic regression was used to determine the factors that associated with barrier to accessing the health care. Multilevel mixed effect logistic regression follows four models. The null model (only outcome variable), mode I (individual level variable such as age, Religious status**,** educational status, Husband educational, marital occupation**,** Husband occupation**,** marital status**,** Contraceptive Utilization, place of delivery, ANC visit, media exposure, Intermate utilization**,** visiting health facility within 12 months, health insurance , wealth index) model II community level variables, Region in sub-Saharan Africa**,** community poverty level, community media exposure, Community distance health facility, Community literacy level, Community ANC visit), and model III (both individual and community level variables such as age, Religious status**,** educational status, Husband educational, marital occupation**,** Husband Occupation**,** marital status**,** Contraceptive Utilization, place pf delivery, ANC visit, media exposure, Intermate utilization**,** visiting health facility within 12 months, health insurance , wealth index), model II (community level variables, Region in sub-Saharan Africa**,** community poverty level, community media exposure, Community distance health facility, Community literacy level, Community ANC visit). The null model without independent variables was used to check the variability of outcome variable across the cluster. The relationship of individual-level variables with outcome variable (Model I) and the association of community-level variables with outcome variable (Model II) were determined. In the final model, the association of both individual and community-level variables was fitted with the outcome variable.

Random effects

The intra-class correlation coefficient (ICC), proportionate change in variance (PCV), and median odds ratio (MOR) were used to quantify random effects or measures of variation of the outcome variables.

To assess the variation between clusters, the proportionate change in variance (PCV) and intra-cluster correlation coefficient (ICC) were calculated. By treating the cluster as a random variable, the ICC shows that the variation in the health care access barrier between clusters may be computed as follow $\boldsymbol{ICC} = \frac{\sigma^2 uo}{\sigma^2 uo + \pi^2/3} \times \mathbf{100\%}$. The MOR, which is determined by using clusters as a

random variable and calculating the median value of the odds ratio between the highest-risk and lowest-risk areas for health care access barriers, is calculated. $MOR = \exp(0.6745\ \sqrt{(2 * \sigma^2 uo}))$ Furthermore, the PCV calculates a variable that explains the variation in the degree of barriers to accessing healthcare ; $PCV = \frac{\sigma^2 uo - \sigma^2}{\sigma^2 uo} \times 100\%$;. where VC is the cluster level variance and Vnull is the variance of the null model.

The associations between independent factors at the individual and community levels and barriers to accessing health care were estimated using the fixed effects. It was assessed and presented using adjusted odds ratio (AOR) and 95% confidence intervals with a p-value of < 0.05. Because of the nested nature of the model, deviation = -2(log likelihood ratio) was used to compare models, and the model with the lowest deviance was selected as the best-fitted model. By evaluating the variance inflation factors, the model assumption for multi-collinearity was confirmed. In addition, Model comparison was performed using Akaike's Information Criterion (AIC). The model includes both random intercepts and random slopes (Model III) was evaluated alongside alternative models, with AIC values calculated to assess relative model fit and parsimony.

Ethical approval and consent to participate

This study was based on analysis of existing survey datasets in the public domain (SSA) that are freely available online with all the identifier information anonymized, no ethical approval was required

Result

In this study a total of 134,470 reproductive age women were included in Sub-Saharan African. The Mean age of participants were 28.99 (Std. Dev. 9.68) years. More than two thirds (69.8%) of women did not use contraception, and over 72.22% reproductive women did not use health insurance, and over half of (54.22%) women were living rural areas in sub-Saharan Africa **(Table 2)**

Table 2: Sociodemographic and economic characteristics of reproductive women who had barriers to accessing health care in sub-Saharan Africa.

Variable	frequency(n)	percentage (%)
**Individual and community level variables**		

**Maternal age**		
15-19	27890.4	20.7
20-35	69441.4	51.64
36-49	37138.6	27.64
**Residency**		
Urban	61558.03	45.78
Rural	72912.41	54.22
**Religious status**		
Muslim	48834.95	41.96
Christian	46856.90	39.30
Other	23524.59	19.73
**Educational Status**		
No formal education	35051.70	26.06
Primary education	39651.17	29.48
Secondary and higher education	59767.57	44.44
**Husband educational**		
No formal education	29414.44	36.72
Primary education	21245.78	26.52
secondary and higher education	29428.78	36.74
**Maternal Occupational**		
Not working	50732.90	37.81
working	83417.01	62.19
**Husband Occupation**		
Not working	10390.43	13.00
Working	69698.11	87.00
**Maternal status**		
single	42213.40	31.4
Married	58262.00	43.3
Other	33995.4	25.3
**Contraceptive Utilization**		
No	93855.00	69.8
Yes	40615.5	30.2
**place of delivery**		
Home	8279.46	15.5
Institution	44979.28	84.5
ANC visit		
No visiting	2012.84	3.77
1-4 visiting	24459.34	45.92
>4 visiting	26786.55	50.29

Media exposure

No	34628.45	25.75
Yes	99831.57	74.24
**Intermate utilization**		
No	93020.34	69.17
Yes	41450.10	30.82
**Visiting health facility 12 month**		
No	57137.73	42.49
Yes	77332.71	57.50
**Health insurance**		
No	85044.69	72.22
Yes	32709.39	27.77
**Wealth index**		
poor	47227.18	35.12
middle	25771.45	19.16
Rich	61471.81	45.74
**Region in sub-Saharan Africa**		
East Africa	59786.29	44.46
West Africa	74684.16	55.54
**Community poverty level**		
Low	70776.71	52.63
High	63693.73	47.47
**Community media exposure**		
Low	61642.30	45.84
High	72828.14	54.15
**Community distance health facility**		
Low	65962	49.00
High	68508	51.00
**Community literacy level**		
Low	57615.01	42.84
High	76855.43	57.15
**Community ANC visit**		
Low	77912.32	57.96
High	56491.92	42.03

In this study, the magnitude of barriers to accessing health care among reproductive age women was found to be 55.84 (95% CI: (55.57, 56.10) owing to one or more of the four factors, the most commonly cited being the difficulty in getting money and the distance from medical facilities (**Figure 1)**

Random effect (Measures of variation) and model fitness

To determine if the data supported the choice to evaluate randomness at the community level, a null model was employed. With the variance of 0.3784967 and a p-value of < 0.001, the null model's results showed that there were notable variations in the barriers to accessing healthcare among clusters. A significant amount of the variance in the outcome between clusters was jointly explained by both individual- and community-level variables, as evidenced by the intra-cluster correlation (ICC), which dropped from 10.31% in the null model to 3.96% in the fully adjusted model (model III). According to the proportionate change in variance (PCV), Model III explains the greatest percentage (64.09%) of the group-level variation in comparison to the null model, group comparison showed that, Model I (50.19%) and Model II (24.56%). This suggests that the model fits better as more predictors are included. Model comparison using Akaike's Information Criterion (AIC) indicated that Model III achieved the best fit, as evidenced by the lowest AIC (239,608.6), markedly lower than both the null model (AIC = 1,128,437) and Model II (AIC = 1,127,704). Model I also showed good fit (AIC = 239,764.8), though slightly less optimal than Model III. Multicollinearity using variance inflation factors (VIF) indicated that all VIF values ranged from 1.03-1.98, which is within accepted range **(Table 3)**

Table 3: Model comparison and Fitness random effect analysis for barriers of accessing health care of among reproductive age women in sub-Saharan Africa**.**

Parameter	Null model	model I	model II	model III
Variance	0.3784967	0.1885269	0.28553	0.1358817
ICC	10.31%	5.41%	7.98%	3.96%
MOR	1.79	1.51	1.66	1.41
PCV	Reference	50.19%	24.56%	64.09%
**Model fines**				
LLR	-90175.365	-21798.27	-86841.704	-21433.284
Deviance	1803596.73	43596.54	173683.408	42866.568
AIC	1128437	239764.8	1127704	239608.6

ICC: intra-cluster correlation**, LLR:** loglikelihood ratio**, MOR:** median odds ratio**, PCV:** proportional change in variance. **AIC:** Akaike information criteria.

Determinants of barriers to accessing health care among reproductive age women in Sub-Saharan Africa

Bivariable logistic regression analysis of individual and community level variables associated with barriers to accessing health care among reproductive age women in Sub-Saharan Africa

Bivariable multilevel logistic regression analysis indicated that husband's educational status, media exposure, internet utilization, place of residence, antenatal care attendance, place of delivery, health insurance coverage, community wealth index, recent health facility visits, and country category (Western vs. Eastern Africa) were associated with barriers to accessing health care among reproductive-age women at a p-value < 0.25 (**Table 4**)

Table 4: Bivariable logistic regression of individual and community level factors associated with barriers to accessing health care among reproductive age women in Sub-Saharan Africa.

Variables	Crude Odds Ratio [COR]	P. value	[95% CI]
**Maternal age**			
15-19	0.86	<0.001	[0.83, 0.89]
20-35	0.86	<0.001	[0.84, 0. 88]
36-49	1		1
**Residency**			
Urban	1		1
Rural	2.09	<0.001	[2.04, 2.14]
**Maternal education**			
No education	2.40	<0.001	[2.33, 2.46]
Primary education	1.52	<0.001	[1.48, 1.56]
Secondary & Higher	1		1
**Husband education**			
No education	2.43	<0.001	[2.35, 2.51]
Primary education	1.55	<0.001	[1.49, 1.60]
secondary & higher	1		1
**Maternal occupation**			
working	1		1
not working	0.94	<0.001	[0.92, 0.96]
**Husband occupation**			
working	1		
not working	1.07	<0.001	[1.02, 1.12]
**Religion**			
Muslim	1		1
Christian	1.32	<0.001	[1.29, 1.35]
Other	1.08	<0.001	[1.04, 1.11]

**Contraceptive utilization**			
No	1.18	<0.001	[0.82, 0.86]
Yes	1		1
**Place of delivery**			
Home	1.84	<0.001	[1.75, 1.93]
Institution	1		1
**ANC visit**			
No visit	1.16	<0.002	[1.05, 1.27]
1-4 visit	1.05	<0.003	[1.01, 1.09]
> 4 visits	1		1
**Marital status**			
single	1		
Married	1.23	<0.001	[1.20, 1.26]
Other	1.32	<0.001	[1.29, 1.36]
**Media exposure**			
No	1.90	<0.001	[1.85, 1.95]
Yes	1		1
**Internet utilization**			
No	2.321822	<0.001	[2.26, 2.37]
Yes	1		1
**Visiting health facility 12 month**			
No	1.02	<0.046	[1.00, 1.04]
Yes	1		1
**Health insurance**			
No	1.21	<0.001	[1.18, 1.25]
Yes	1		1
**Wealth index**			
poor	3.58	<0.001	[3.49, 3.67]
middle	2.08	<0.001	[2.02, 2.14]
Rich	1		1
**Region SSA**			
East Africa	1.68	<0.001	[1.65, 1.72]
West Africa	1		1
**community ANC visit**			
Low	0.86	<0.001	[0.85, 0.88]
High	1		1
**community wealth index**			
Low	0.69	<0.001	[0.67, 0.70]
High	1		
**Community media exposure**			
Low	0.73	<0.001	[0.72, 0.75]
High	1		1
**Community literacy level**			
Low	0.77	<0.001	[0.75, 0.79]
High	1		1

Multivariable multilevel logistic regression analysis of individual and community level factors associated with Barriers to health care access among reproductive Age Women in Sub-Saharan Africa.

In the final fitted model of multivariable multilevel logistic regression, Husband educational status, media exposure, place of residency, Antenatal care visit, place of delivery, health insurance utilization, wealth index status, visiting health facility within 12 months, and country category (western, eastern Africa) were significantly associated with barriers of accessing health care among reproductive age women in sub-Saharan Africa. The odds of barrier of accessing health care were 1.30 times higher among reproductive women living in rural residency compared to women living in urban area **[AOR=**1.30, 95 CI: (1.22, 1.39)]. and the odds of barrier of accessing health care were 1.23 times more likely to among reproductive age women whose husband had no education compared to women whose husband had secondary and higher education [**AOR**=1.23, 95 CI: (1.15, 1.30,)]. In addition, odds of barrier of accessing health care were 1.33 times more likely to occur women delivered at home compared to women who had institutional delivery. **(AOR**=1.33, 95% CI: (1.24, 1.43) and the odds of barrier of accessing health care were 1.31 times higher among women who had no history of antenatal visit compared to women who had more than four ANC visit. [**AOR**= 1.31, 95% CI: (1.10, 1.55)]. The odds of the barrier of accessing health care among reproductive age woman who had no media exposure were 1.17 times more likely to compared with a woman who had media exposure **[AOR**=1.17, 95% CI: (1.11, 1.24)] and the barrier of accessing health care among reproductive age woman who were not visiting health facility within 12 months 9% times less likely compared to women who had visiting health care facility within 12 months of study period **[AOR=**0.91, 95% CI: (0.87, 0.97)]. The odds of perceived barrier of accessing health care among reproductive age woman who had no health insurance utilization were 1.18 times more likely to compared with a woman who had used health insurance [**AOR=**1.18 95% CI: (1.11, 1.26)] and The odds of perceived barrier of accessing health care service among reproductive age woman who were classified under poor and middle wealth status were 2.29 and 1.57 times more likely barries accessing health care compared to reproductive women who were classified under rich wealth status category **[AOR**=2.29 95% CI: (2.14, 2.45)] and [**AOR**=1.57, 95% CI: (1.46, 1.68)]. The odds of perceived barrier of accessing health care service among reproductive age woman who reside in west Africa region were 2.38 times more

likely barriers accessing health care compared to east Africa region category. [**AOR**=2.38, 95% CI: (2.21, 2.55)] **(Table 5).**

Table 5: Multivariable multilevel logistic regression of individual and community level factors associated with barriers to accessing health care among reproductive age women in Sub-Saharan Africa.

Individual and community level factors	Model I AOR [95% CI]	Model II AOR [95% CI]	Model III AOR [95% CI]
**Maternal age**			
15-19	0.96 [0.86, 1.07]		0.96 [0.86,1.08]
20-35	1.06 [1.01,1.13]		1.04 [0.99, 1.11]
36-45	1		1
**Residency**			
Urban		1	1
Rural		2.29 [2.24, 2.35]	**1.30 [1.22, 1.39]**
**Maternal education**			
No education	1.10 [1.02, 1.18]		1.04 [0.97, 1.11]
Primary education	0.93 [0.87, 0.99]		1.06 [0.99, 1.14]
Secondary and above	1		1
**Husband education**			
No education	1.26 [1.19,1.35]		**1.23 [1.15, 1.30]**
Primary education	1.04 [0.97,1.12]		1.23 [0.67, 1.22]
secondary & higher education	1		1
**Maternal occupation**			
working	1		1
not working	0.99 [0.94, 1.04]		**1.02 [1.01, 1.11]**
**Husband occupation**			
working	1		1
not working	0.90 [0.83, 0.97]		1.00 [0.92, 1.08]
**Religion**			
Muslim	1		1
Christian	1.62 [1.53, 1.71]		0.46 [0.57, 1.94]
Other	1.22 [1.13, 1.31]		1.36 [0.75, 1.46]
**Contraceptive utilization**			
No	1.11 [1.05,1.17]		1.03 [0.98, 1.08]
Yes	1		1
**Place of delivery**			
home	1.30 [1.21, 1.40]		**1.33 [1.24, 1.43]**
Institution	1		**1**

**Antenatal Care Visit**			
No	1.01 [0.85, 1.19]		**1.31 [1.10, 1.55]**
1-4 visit	0.94 [0.89, 0.99]		1.13 [0.37, 1.19]
> 4 visits	1		1
**Media exposure**			
No	1.14 [1.08, 1.21]		**1.17 [1.11, 1.24]**
Yes	1		1
**Internet utilization**			
No	1.69 [1.58, 1.80]		1.68 [0.67, 1.79]
Yes	1		1
**Visiting health facility 12 month**			
No	0.92 [0.87, 0.98]		**0.91 [0.87, 0.97]**
Yes	1		1
**Health insurance**			
No	1.26 [1.19, 1.34]		**1.18 [1.11, 1.26]**
Yes	1		1
**Wealth index**			
poor	2.80 [2.63, 2.97]		**2.29 [2.14, 2.45]**
middle	1.79 [1.68, 1.92]		**1.57 [1.46, 1.68]**
Rich	1		1
**Region SSA**			
East Africa		1	**1**
West Africa		2.0 [1.92-2.04]	**2.38 [2.21, 2.55]**
**community ANC visit**			
Low		1.00 [0.93, 1.09]	0.93 [0.86, 1.00]
High		1	1
**community wealth index**			
Low		0.75 [0.70, 0.81]	**1.12 [1.02, 1.22]**
High		1	1
**Community media exposure**			
Low		1.01 [0.92, 1.10]	0.94 [0.86, 1.03]
High		1	1
**Community literacy level**			
Low		1.10 [1.00, 1.20]	**1.13 [1.03, 1.24]**
High		1	**1**

Model I: *individual level variable,* **Model II:** *Community level variable,* **Model III**: *Individual and community level variable*

Discussion

Barriers to accessing health care have contributed to maternal and child mortality and morbidity in developing countries such as those in Sub-Saharan Africa. This study aimed to assess the

magnitude and determinants of barriers to health care access among women of reproductive age in Sub-Saharan Africa.

In this study, the magnitude of barriers to accessing health care among reproductive age women was found to be 55.84 (95% CI: (55.57, 56.10) owing to one or more of the four factors, the most commonly cited being the difficulty in getting money and the distance from medical facilities. This finding is lower than the previous study conducted in South Africa 65% [18, 19], and Tanzania 65% [15], Ethiopia 71.8%,70.2, 69.9%[1, 20, 21], (SSA) 60.4%, 61.5% [17]. this variation could be due to the fact that socio-economic and variability in health infrastructure and health system policy, women's attitudes towards health care seeking behavior and cultural differences across countries in sub-Saharan Africa, moreover, this discrepancy might be due to study period, this study includes, the most recent DHS data set from 2019-2023. On the other hand, the finding of this study is similar to but slightly higher than in previous study in Gambia 45.5%[22], Ghana (51%)[23]. this variation could be due to the fact that socio-economic and variability in health infrastructure and health system policy, women's attitudes towards health care seeking behavior and cultural differences across countries in sub-Saharan Africa.

The odds of barrier of accessing health care were 1.30 times higher among reproductive women living in rural residency compared to women living in urban area. This finding is supported by previous studies conducted in Ethiopia [1, 20], guinea[24], Gambia, Tanzania and Ghana [15, 22, 25] respectively. Sub-Saharan[23], East Africa[26]. This association may be, due to the fact that rural areas have low health infrastructure i.e. inaccessibility of health facilities, less privileged and the influences of socio-cultural practices that need women to seek permission from their husband to visit the facility or women's decisions for seeking healthcare services is under their husband/partners control. Moreover, male involvement and support for women health care access are limited[1, 14, 23, 26]. This implies that the government should expand infrastructure services in rural area and encourage male involvement and support for improving health care access for reproductive age women[14].

The odds of perceived barrier of accessing health care were 1.23 and 1.21 times more likely to among reproductive age women whose husband had no education and primary education compared to women whose husband had secondary and above educational level respectively. This find was consistent with previous study conducted in east Africa, Ethiopia, Tanzania, Benin,

Bangladesh respectively [1, 15, 20, 26, 27]. The possible explanation could be in better access to the healthcare among women with educated husbands might be due to better participation and involvement of husbands in their families' health[23]. Women whose partners are educated are also likely to be informed regarding their fundamental human rights and may have higher health literacy. As a result, they are more likely to deal with any form of barrier to healthcare, compared to their counterparts who are less educated and may have lower health literacy [14, 15, 20, 28].

The odds of barrier to accessing health care were 1.33 times more likely to occur women delivered at home compared to women who had institutional delivery. This evidence was supported by study done in Senegal[29]. The plausible justification might be women who delivered at home may not get health information/counselling from healthcare providers that empower them to use other maternal healthcare services and women who deliver at home may face varous barriers to accessing health care such as financial constraint, lack of transportation, limited autonomy in healthcare decision and sociocultural norms that discourage institutional delivery. Moreover, importance of enhancing health system outreach, strengthen community-based education, and addressing sociocultural and socioeconomic disparities that affect health seeking behavior. On the other hand, the odds of barrier of accessing health care among woman who had no media exposure were 1.17 times more likely to compared with a woman who had media exposure. This evidence was supported by study done in Ethiopia[30], Nepal[31]. This could be the power of mass media in disseminating information concerning maternal health that may enhance women's knowledge and attitude towards health care access and utilization[31]. Barrier of accessing health care among woman who were not visiting health facility within 12 months were 9 % times less likely compared to women who had visiting health care facility within 12 months of study period. The odds of barrier to accessing health care among woman who had no health insurance utilization were 1.18 times more likely to compared with a woman who had used health insurance. This finding was supported with study done in Ethiopia[1], sub-Saharan Africa[23], Tanzania[15], Ghana[25]. The possible reason might be due to the fact that, individuals who were not had health insurance faced for unexpected catastrophic expenditure and difficulties in obtaining money for consultation of the doctor[1]. The odds of barrier to accessing healthcare among woman who were classified under poor and middle wealth status were 2.29 and 1.57 times more likely accessing health care difficulty compared to women who were classified under rich wealth index category**.** This evidenced was supported with study done in Ethiopia, [1, 30, 32, 33] Tanzania[15] Uganda[34], sub Saharan

Africa[14]. This finding might be explained by the fact that high out-of-pocket expenses and a lack of accessibility to healthcare facilities pose significant obstacles for middle-class and impoverished women in sub-Saharan Africa. Many people in the region cannot afford healthcare services because over 40% of health costs are paid for directly by individuals[35-38]

The odds of barrier of accessing health care among woman who live west Africa region were 2.38 times more likely barriers to accessing health care compared to east Africa region. According a recent joint report from the World Health Organization and the United Nations states that high out-of-pocket costs, a lack of adequate healthcare infrastructure, and other sociocultural barriers make it more difficult for women of reproductive age in West African nations such as Nigeria, Mali, and Sierra Leone to access maternal healthcare. On the other hand, East African countries like Rwanda, Kenya, and Ethiopia have strengthened their community health initiatives and gender-inclusive policies, which have improved women's autonomy in accessing healthcare services[39]. Furthermore, our current findings are supported by recent Demographic and Health Survey (DHS) program data (2022–2023), which indicate that women in West African countries are 2-3 times more likely to report delays in accessing healthcare due to cost, shortages of healthcare personnel, and long distances to health facilities. In contrast, East African countries, particularly Uganda and Tanzania, have strengthened their health insurance systems and rural outreach initiatives. Additionally, the World Bank's 2023 Health Service Coverage Index reveals that women in West Africa have significantly lower access to family planning, skilled birth attendance, and other essential reproductive health services[22, 40, 41]

Strength and limitation

This strength of study nationally representative data to evaluate contextual and individual characteristics related to barriers to healthcare access among reproductive age women in the SSA. Thus, findings can therefore, generalized to all women in their reproductive age in SSA, in order to account for the clusters within the sample, the study also employed advanced statistical models. This study has several limitations. The cross-sectional design restricts the ability to draw causal inferences, and health worker–related factors were not incorporated into the analysis. Furthermore, reliance on self-reported data may introduce recall or social desirability bias; future research should consider integrating objective measures, such as wearable devices, as demonstrated by Zhao et al. (2025)[42]

Conclusion and Recommendation

This study found that 55.55% of reproductive age women had barriers to accessing healthcare in SSA. Husband educational status, media exposure, internet utilization, place of residency, ANC visit status, place of delivery, health insurance utilization status, wealth index status, visiting health facility within 12 months, community literacy level and community wealth index and country category (western, eastern Africa) were significantly associated with barriers of accessing health care. Therefore, the Government and ministry of health in Sub-Saharan Africa countries should taking in to consideration those factors while designing policies and strategies targeting to health care access and tackling the preventable barriers to accessing health care in sub-Saharan Africa.

Declaration

Ethical approval and consent to participate

This study was based on analysis of existing survey datasets in the public domain (SSA) that are freely available online with all the identifier information anonymized, no ethical approval was required

Consent for publication

Not applicable

Data and material availability

Demographic and Health Survey were used, and it is publicly available online at (https://www.dhsprogram.com).

Competing interest

Authors Declared that, there is no competing interest.

Funding

Not applicable

Author's contributions

GAZ: involved in designing, data extraction, data analysis, interpretation, report and manuscript writing.

BT: involved in data analysis, interpretation, and, and manuscript writing.

BT: involved in analysis,

TTT: involved in designing the study, interpretation, report and

BSW: involved in analysis, and result interpretation

AFZ: involved in designing and interpretation study

EGM: analysis and manuscript writing

MW: Result interpretation

AA: manuscript writing: involved in data extraction

MSA: involved manuscript writing

Acknowledgements

We would like to say the DHS programs for allowing us to use the relevant DHS dataset in this study.

Reference

1. Tamirat, K.S., Z.T. Tessema, and F.B.J.B.H.S.R. Kebede, Factors associated with the perceived barriers of health care access among reproductive-age women in Ethiopia: a secondary data analysis of 2016 Ethiopian demographic and health survey. 2020. **20**: p. 1-8.
2. Erasmus, M.O., The barriers to access for maternal health care amongst pregnant adolescents in the Mitchells plain sub-district. 2017.
3. Washington, D.L., et al., Access to care for women veterans: delayed healthcare and unmet need. 2011. **26**: p. 655-661.
4. Regan, L.J.B.M.A., Addressing unmet needs in global women's health. 2018.
5. Izugbara, C., E. Covan, and E. Fugate-Whitlock, Women's health in Africa: Issues, challenges and opportunities. 2017: Routledge.
6. Organization, W.H., Primary health care on the road to universal health coverage: 2019 global monitoring report: Executive summary. 2019, World Health Organization.
7. Badiuzzaman, M., S.M. Murshed, and M.J.T.J.o.D.S. Rieger, Improving maternal health care in a post conflict setting: evidence from Chittagong Hill tracts of Bangladesh. 2020. **56**(2): p. 384-400.
8. Peters, D.H., et al., Poverty and access to health care in developing countries. 2008. **1136**(1): p. 161-171.
9. Alkire, B.C., et al., The economic consequences of mortality amenable to high-quality health care in low-and middle-income countries. 2018. **37**(6): p. 988-996.
10. Nyakang'o, S.B. and A.J.M. Booth, Women's perceived barriers to giving birth in health facilities in rural Kenya: A qualitative evidence synthesis. 2018. **67**: p. 1-11.
11. Colglazier, W.J.S., Sustainable development agenda: 2030. 2015. **349**(6252): p. 1048-1050.

12. Cahill, C.M., et al., Preoperative anemia management program reduces blood transfusion in elective cardiac surgical patients, improving outcomes and decreasing hospital length of stay. 2021. **61**(9): p. 2629-2636.
13. Peeri, N.C., et al., The SARS, MERS and novel coronavirus (COVID-19) epidemics, the newest and biggest global health threats: what lessons have we learned? 2020. **49**(3): p. 717-726.
14. Tessema, Z.T., et al., Determinants of accessing healthcare in Sub-Saharan Africa: a mixed-effect analysis of recent Demographic and Health Surveys from 36 countries. 2022. **12**(1): p. e054397.
15. Bintabara, D., K. Nakamura, and K.J.B.o. Seino, Improving access to healthcare for women in Tanzania by addressing socioeconomic determinants and health insurance: a population-based cross-sectional survey. 2018. **8**(9): p. e023013.
16. Kea, A.Z., et al., Exploring barriers to the use of formal maternal health services and priority areas for action in Sidama zone, southern Ethiopia. 2018. **18**: p. 1-12.
17. Zegeye, B., et al., Breaking barriers to healthcare access: a multilevel analysis of individual-and community-level factors affecting women's access to healthcare services in Benin. 2021. **18**(2): p. 750.
18. Silal, S.P., et al., Exploring inequalities in access to and use of maternal health services in South Africa. 2012. **12**: p. 1-12.
19. Tsawe, M. and A.S.J.B.r.n. Susuman, Determinants of access to and use of maternal health care services in the Eastern Cape, South Africa: a quantitative and qualitative investigation. 2014. **7**: p. 1-10.
20. Zegeye, B., et al., Barriers and facilitators to accessing health care services among married women in Ethiopia: a multi-level analysis of the Ethiopia demographic and health survey. 2021.
21. Demsash, A.W., B. Chakilu, and A. Mazengia, Knowledge Sharing Practice and Its Associated Factors Among Healthcare Providers at University of Gondar Comprehensive Specialized Hospital, North West Ethiopian: Cross-sectional Study. 2021.
22. Tsegaw, M., B. Mulat, and K.J.B.o. Shitu, Problems with accessing healthcare and associated factors among reproductive-aged women in the Gambia using Gambia demographic and Health Survey 2019/2020: a cross-sectional study. 2023. **13**(8): p. e073491.
23. Seidu, A.-A.J.P.O., Mixed effects analysis of factors associated with barriers to accessing healthcare among women in sub-Saharan Africa: Insights from demographic and health surveys. 2020. **15**(11): p. e0241409.
24. Seidu, A.-A., et al., Individual and contextual factors associated with barriers to accessing healthcare among women in Papua New Guinea: insights from a nationwide demographic and health survey. 2021. **13**(6): p. 573-585.
25. Seidu, A.-A., et al., Barriers to accessing healthcare among women in Ghana: a multilevel modelling. 2020. **20**: p. 1-12.
26. Minyihun, A., Z.T.J.R.M. Tessema, and H. Policy, Determinants of access to health care among women in East African countries: a multilevel analysis of recent demographic and health surveys from 2008 to 2017. 2020: p. 1803-1813.
27. Sen, K.K., et al., Do education and living standard matter in breaking barriers to healthcare access among women in Bangladesh? 2023. **23**(1): p. 1431.

28. Dimbuene, Z.T., et al., Women's education and utilization of maternal health services in Africa: a multi-country and socioeconomic status analysis. 2018. **50**(6): p. 725-748.
29. Zegeye, B., et al., Predictors of institutional delivery service utilization among women of reproductive age in Senegal: a population-based study. 2021. **79**: p. 1-11.
30. Demsash, A.W., A.D.J.B.H. Walle, and C. Informatics, Women's health service access and associated factors in Ethiopia: application of geographical information system and multilevel analysis. 2023. **30**(1).
31. Tamang, T.M. Factors associated with completion of continuum of Care for Maternal Health in Nepal. in IUSSP XXVIII International Population Conference, Cape Town, South Africa. 2017.
32. Fentie, E.A., et al., Spatial distribution and determinants of barriers of health care access among female youths in Ethiopia, a mixed effect and spatial analysis. 2023. **13**(1): p. 21517.
33. Alamneh, T.S., et al., Barriers for health care access affects maternal continuum of care utilization in Ethiopia; spatial analysis and generalized estimating equation. 2022. **17**(4): p. e0266490.
34. Kalule-Sabiti, I., A.Y. Amoateng, and M.J.A.P.S. Ngake, The effect of socio-demographic factors on the utilization of maternal health care services in Uganda. 2014: p. 515-525.
35. Shiferaw, S., et al., Why do women prefer home births in Ethiopia? 2013. **13**: p. 1-10.
36. Yaya, S., G. Bishwajit, and V.J.B.g.h. Shah, Wealth, education and urban–rural inequality and maternal healthcare service usage in Malawi. 2016. **1**(2): p. e000085.
37. Voto, T.P., B.E. Voto, and N.J.E. Ngepah, The Impact of Out-of-Pocket Health Expenditure and Public Health Expenditure on Poverty in Sub-Saharan Africa. 2025. **13**(5): p. 134.
38. Alamneh, T.S., et al., Socioeconomic inequality in barriers for accessing health care among married reproductive aged women in sub-Saharan African countries: a decomposition analysis. 2022. **22**(1): p. 130.
39. AKINWALE, A.A.J.I.J.o.I.R. and A. Studies, Gender equity and social progress: empowering women and girls to drive sustainable development in Sub–Saharan Africa. 2023.
40. Arhin, K., E.F. Oteng-Abayie, and J.J.H.e.r. Novignon, Assessing the efficiency of health systems in achieving the universal health coverage goal: evidence from Sub-Saharan Africa. 2023. **13**(1): p. 25.
41. Muhajarine, N., et al., Inequalities and factors associated with maternal healthcare services utilisation in Mozambique: evidence from the Demographic and Health Survey 2022−2023. 2025. **10**(5).
42. Zhao, Y., et al., Highly sensitive, wearable piezoresistive methylcellulose/chitosan@ MXene aerogel sensor array for real-time monitoring of physiological signals of pilots. 2025. **68**(2): p. 542-551.

Figure legend

Fig 1: Barriers to accessing health care and determinant factor among reproductive age women in sub-Saharan Africa

Manuscript

Click here to access/download;Manuscript;addetinal revised manuscrpt.docx

Barriers to accessing health care among women in Sub-Saharan Africa: Multilevel analysis of recent demographic and health survey from 2019-2023.

Gebreeyesus Abera Zeleke[1]*, Enyew Getaneh Mekonen[1], Bewuketu Terefe[2], Mulugeta Wassie[3], Agazhe Aemro[3], Alebachew Ferede Zegeye[3], Mohammed Seid Ali[4], Berhan Tekeba[4], Tadesse Tarik Tamir[4], Belayneh Shetie Workneh[5]

[1] Department of Surgical Nursing, School of Nursing, College of Medicine and Health Sciences, University of Gondar, Gondar, Ethiopia

[2] Department of Community Health Nursing, School of Nursing, College of Medicine and Health Sciences, University of Gondar, Gondar, Ethiopia.

[3] Department of Medical Nursing, School of Nursing, College of Medicine and Health Sciences, University of Gondar, Gondar, Ethiopia;

[4] Department of Pediatrics and Child Health Nursing, School of Nursing, College of Medicine and Health Sciences, University of Gondar, Gondar, Ethiopia.

5 Department of Emergency and Critical Care Nursing, School of Nursing, College of Medicine and Health Sciences, University of Gondar, Gondar, Ethiopia

Correspondence Author*
Gebreeyesus Abera Zeleke

Department of Surgical Nursing, School of Nursing, College of Medicine and Health Science, University of Gondar, Gondar, Ethiopia.

Email address: gebreeyesusabe143@gmail.com

Co-authors:

Enyew Getaneh Mekonen **Email Address:** enyewgetaneh12@gmail.com

Bewuketu Terefe: **Email address**: woldeabwomariam@gmail.com

Mulugeta Wassie: **Email address:** mulugeta2113@gmail.com

Agazhe Aemro: **Email address:** agazhe1049@gmail.com

Alebachew Ferede Zegeye: **Email address:** alexferede24@gmail.com

Mohammed Seid Ali: **Email address:** muheseid2592@gmail.com

Berhan Tekeba: **Email address:** berishboss7@gmail.com

Tadesse Tarik Tamir: **Email address:** tadestar140@gmail.com

Belayneh Shetie Workneh: **Email address:** belay2319@gmail.com

Abstract

Background:

Barriers to accessing healthcare have significantly contributed to the high rates of maternal and child mortality and morbidity in developing regions, particularly in Sub-Saharan Africa. Access to healthcare is shaped by multiple factors, including financial constraints, geographic location, the availability of services, and the quality of care provided. This study aimed to assess the magnitude of these barriers and to identify the factors influencing healthcare access among women of reproductive age in Sub-Saharan Africa.

Methods:

This study utilized secondary data from the most recent Demographic and Health Surveys conducted between 2019 and 2023 across Sub-Saharan Africa. A weighted sample of 134,470 women of reproductive age was included in the analysis. Data were analyzed using Stata version 14, employing a multilevel mixed-effects logistic regression model. Factors associated with barriers to healthcare access were considered statistically significant at P-values less than 0.05. Adjusted odds ratios (AORs) with 95% confidence intervals (CIs) were used to interpret the results.

Result: In this study, the magnitude of barriers to accessing healthcare among reproductive age women were found to be 55.84% (95% CI: 55.57–56.10). Husband's educational status [AOR = 1.23, 95% CI: 1.15–1.30], Media exposure [AOR = 1.17, 95% CI: 1.11–1.24], Antenatal care visit [AOR = 1.31, 95% CI: 1.10–1.55], and place of delivery [AOR = 1.33, 95% CI: 1.24–1.43]. Health insurance utilization [AOR = 1.18, 95% CI: 1.11–1.26], Wealth index, 2.29 [2.14, 2.45], 1.57 [1.46, 1.68] and visiting a health facility within the past 12 months [AOR = 0.91, 95% CI: 0.87–0.97] were individual associated variable. In addition, place of residence [AOR = 1.30, 95% CI: 1.22–1.39], community literacy level [AOR = 1.13, 95% CI: 1.03–1.24], community wealth index [AOR = 1.12, 95% CI: 1.02–1.22], and Region were associated with barriers to accessing healthcare.

Conclusion and recommendation: This study found that 55.55% of women in Sub-Saharan Africa experienced barriers to accessing healthcare. Educational status, media exposure, place of residence, antenatal care attendance, place of delivery, health insurance coverage, wealth index, recent visits to health facilities, community literacy levels, community wealth status, and regional differences were determinant factors. These findings highlight the need for Sub-Saharan African governments to prioritize these determinants when formulating policies and strategies aimed at reducing preventable barriers to healthcare access in the region.

Key words: Access to Healthcare; Barriers to Healthcare; Sub-Saharan Africa; Reproductive Age Women

Introduction

Access to healthcare is broadly defined as the ability to prevent illness, disability, and premature death, to promote and maintain health, and to attain health equity, particularly for women of reproductive age, by offering inclusive, high-quality care and the best possible availability, affordability, and accessibility of medical services[1-3]. Reproductive-age women's health is a critical global public health concern since it influences population health outcomes and fosters socioeconomic growth[4]. The advancement of socioeconomic development is significantly influenced by the health of women of reproductive age, especially in Africa. [5]. The Sustainable Development Goals (SDGs) place a strong emphasis on women's health, with Targets 3.7 and 3.8 of SDG 3 specifically focusing on achieving universal health coverage by 2030 and ensuring access to sexual and reproductive health services, including education and information on family planning[6, 7]. The Sustainable Development Goals (SDGs) explicitly target a substantial reduction in maternal mortality, with Goal 3.1 aiming to lower the global maternal mortality ratio to fewer than 70 per 100,000 live births by 2030. While these global efforts have contributed to some improvements in women's health outcomes, they have yet to fully address the root causes of maternal mortality. Achieving sustained and comprehensive progress remains a critical challenge. In 2016, Sub-Saharan Africa accounted for a disproportionately high share of the 303,000 maternal deaths reported globally, the majority of which were due to preventable causes. Moreover, non-communicable diseases continue to contribute significantly to female morbidity and mortality across nearly all countries, with a particularly severe impact in low- and middle-income settings, where many women are left with long-term disabilities [4-6]. Access to healthcare involves multiple dimensions, including financial affordability, geographic accessibility, availability of medical services, and the quality of care provided in health facilities. [8]. Access to healthcare is a key determinant of whether individuals utilize health services. Globally, approximately 400 million people lack essential healthcare, and around eight million die each year from preventable diseases—resulting in an estimated economic loss of $6 trillion in low- and middle-income countries. [6, 9, 10]. In 2015, world leaders pledged to achieve universal health coverage as part of the Sustainable Development Goals (SDGs), aiming to ensure access to affordable, high-quality medicines and financial protection for all [11, 12]. In 2017, the World Bank and WHO reported that half of the global population lacks access to essential health services. As a result, many countries in Sub-Saharan Africa have made universal healthcare a key part of their national health

strategies. However, there has been slow progress in turning this commitment into tangible results, particularly in securing national funding for equitable, high-quality healthcare, financial security, and overall health [6, 9, 13, 14]. Reproductive age women and child mortality from low family planning uptake, unsafe abortion practices, unintended pregnancies, and an increase in home births are all implications of women in reproductive age having trouble accessing health care[14]. Previous research has offered some evidence regarding the sociodemographic and economic factors that can be barriers to accessing healthcare, such as marital status, place of residence, maternal age, wealth index, educational attainment, and unemployment status. Nevertheless, women of reproductive age continue to face significant disparities in healthcare utilization and access[10, 14-16]. This study makes three unique contributions to the body of existing literature. In order to provide a current and policy-relevant analysis of the barrier to healthcare access, it first makes use of the most recent (2019–2023) Demographic and Health Survey (DHS) data. Second, our analysis takes a harmonized multi-country approach across ten sub-Saharan African nations, improving cross-national comparability and generalizability in contrast to many previous studies that are limited to single-country analyses or rely on older datasets. In order to gain a better understanding of the structural and contextual factors that influence healthcare access, the study employs a comprehensive multilevel analysis of determinants at the individual and community levels. These contributions put our research in a position to guide focused, empirically supported programs provide fair access to healthcare for reproductive age women in sub-Saharan. Therefore, this study aimed to ascertain the magnitude and potential contributing factors associated with barriers to accessing healthcare among women of reproductive age in sub-Saharan Africa.

Methods and materials

Patient and public involvement statement

Secondary data from the Demographic and Health Surveys (DHS) were used in this investigation.

Study design, study area, and period.

Data from ten Sub-Saharan African countries, collected between 2019 and 2023, were used to perform a multilevel mixed-effects analysis, based on the most recent Demographic and Health Surveys (DHS). The DHS collects community-based, cross-sectional data approximately every five years to generate updated health and health-related indicators.

Data source, study population and sampling technique

A secondary data analysis was conducted using Demographic and Health Survey (DHS) data collected between 2019 and 2023 from selected sub-Saharan African countries. The study specifically included DHS datasets from ten countries Burkina Faso, Gabon, Ghana, Kenya, Liberia, Rwanda, Sierra Leone, Tanzania, The Gambia, and Mozambique each of which had recent and publicly available survey data within the specified timeframe. The datasets were appended to assess the extent of, and factors underlying, barriers to healthcare access among women of reproductive age in sub-Saharan Africa. Each country's DHS comprises multiple datasets. The DHS employs a stratified, two-stage cluster sampling design: in the first stage, enumeration areas are selected, followed by systematic sampling of households within each selected area in the second stage.

The variable barrier to accessing health care were generated by computation of (v467b, v467c, v467d, v467f) from maternal recode (IR) data set was recoded. A binary logistic regression model was used to identify determinants a factor barrier to access the health care. Determinants of barrier to accessing the health care were reported in terms of an AOR with a significance level of (95%). In the univariate analysis, at 95% confidence intervals with a p-value of < 0.25 was considered for the candidate multivariable analysis of data. All variables with p values of <0.05 were considered statistically significant. Since DHS data are nationally representative, stratified multistage cluster sampling is required; therefor, sampling weights must be applied to produce unbiased estimates. To correctly weight DHS data, we use the sampling weights provided in the dataset, specifically the individual weight **(**v005**)** for women's individual recode files. This weight must be divided by 1,000,000 (v005/1000000). using "gen wgt=v005/1000000 Stata commands. A total weighted sample of 134,470 reproductive age women were included in the study **(Table 1).**

Table 1: The weighted Sample size for magnitude and determinants of barrier to accessing health care among reproductive age women in sub-Saharan Africa**.**

Country	Year of survey	weighted sample (n)	weighted sample (%)
Brukinefaso	2021	17659	13.13
Gabon	2019-2021	6507.15	4.83
Ghana	2022	15014	11.16
Kenya	2022	16716.36	12.43
Liberia	2019-2020	8065	5.99
Rwanda	2019-2020	14634	10.88
Sera lion	2019	15574	11.58
Tanzania	2022	15254	11.34
Gambia	2019-2020	11865	8.08
Mozambique	2023	13181.93	9.80
TOTAL		134470	100%

Study variable

Outcome variables: The outcome of this study was barrier to access the health care. The Data in barrier of accessing the health care were collected among reproductive age women who used one of the health cares. Measurement of the outcome variables was determined by using computation of four variables; such as Distance to health facility (v467b), difficulty in obtaining money (v467c), getting permission go to the doctor (v467d) and not wanting to go alone (v467f). If a woman of reproductive age reported experiencing at least one of the following challenges, such as difficulty in obtaining money, distance to a health facility, lack of permission to seek treatment, or reluctance to go alone were considered to face significant barriers to healthcare access. These cases were coded as '1' and labeled as 'big problem.' Conversely, women who did not report any of these four barriers were considered to have no significant barriers to healthcare access. These were coded as '0' and labeled as 'no big problem'. This approach is consistent with prior literature using DHS data[17]

Independent Variable: Hence DHS data are hierarchical, independent factors come from two sources that was at individual and community levels factors were considered for this analysis. The individual-level independent variables were women's age (15-19, 20-35, 36-49), Women educational status (No education, Primary education , Secondary & Higher education), women occupation (working, not working), Religion (Muslim, Christian , Others), Wealth index (Poor, Middle, Rich) media exposure (yes, No), internet utilization (yes, No), Residency (urban, rural), Husband educational status (No education, Primary education, secondary& higher education), Husband occupational status (No working, Working), Currently Contraceptive (No, Yes) Visiting health facility in 12 month (No, Yes), place of delivery (Home , Institutional delivery), ANC visit (No antenatal visit, 1-4 visit, > 4 visit)

The community-level variables: Poverty level (Low, High), Literacy level (Low, High), Media exposure, (Low, High), Residency (Urban, rural), Region (East, west), Community ANC visit

Data processing and statistical analysis

Stata version 14 statistical software was used to clean, record, and analyze secondary DHS data sets. The variables in the DHS data are grouped into clusters, and the variables within a cluster are more similar to each other than those in other clusters. The presumptions of equal variance

across clusters and independent data were violated in order to run a typical logistic regression model. This suggests that between-cluster factors must be considered using a complex model.

Given this, multilevel mixed-effects logistic regression was used to determine the factors that associated with barrier to accessing the health care. Multilevel mixed effect logistic regression follows four models. The null model (only outcome variable), mode I (individual level variable such as age, Religious status**,** educational status, Husband educational, marital occupation**,** Husband occupation**,** marital status**,** Contraceptive Utilization, place of delivery, ANC visit, media exposure, Intermate utilization**,** visiting health facility within 12 months, health insurance , wealth index) model II community level variables, Region in sub-Saharan Africa**,** community poverty level, community media exposure, Community distance health facility, Community literacy level, Community ANC visit), and model III (both individual and community level variables such as age, Religious status**,** educational status, Husband educational, marital occupation**,** Husband Occupation**,** marital status**,** Contraceptive Utilization, place pf delivery, ANC visit, media exposure, Intermate utilization**,** visiting health facility within 12 months, health insurance , wealth index), model II (community level variables, Region in sub-Saharan Africa**,** community poverty level, community media exposure, Community distance health facility, Community literacy level, Community ANC visit). The null model without independent variables was used to check the variability of outcome variable across the cluster. The relationship of individual-level variables with outcome variable (Model I) and the association of community-level variables with outcome variable (Model II) were determined. In the final model, the association of both individual and community-level variables was fitted with the outcome variable.

Random effects

The intra-class correlation coefficient (ICC), proportionate change in variance (PCV), and median odds ratio (MOR) were used to quantify random effects or measures of variation of the outcome variables.

To assess the variation between clusters, the proportionate change in variance (PCV) and intra-cluster correlation coefficient (ICC) were calculated. By treating the cluster as a random variable, the ICC shows that the variation in the health care access barrier between clusters may be computed as follow $ICC = \frac{\sigma^2 uo}{\sigma^2 uo + \pi^2/3} \times 100\%$. The MOR, which is determined by using clusters as a

random variable and calculating the median value of the odds ratio between the highest-risk and lowest-risk areas for health care access barriers, is calculated. $\boldsymbol{MOR = \exp(0.6745\ \sqrt{(2 * \sigma^2 uo}})$) Furthermore, the PCV calculates a variable that explains the variation in the degree of barriers to accessing healthcare ; $\boldsymbol{PCV = \frac{\sigma^2 uo - \sigma^2}{\sigma^2 uo} \times 100\%};$. where VC is the cluster level variance and Vnull is the variance of the null model.

The associations between independent factors at the individual and community levels and barriers to accessing health care were estimated using the fixed effects. It was assessed and presented using adjusted odds ratio (AOR) and 95% confidence intervals with a p-value of < 0.05. Because of the nested nature of the model, deviation = -2(log likelihood ratio) was used to compare models, and the model with the lowest deviance was selected as the best-fitted model. By evaluating the variance inflation factors, the model assumption for multi-collinearity was confirmed. In addition, Model comparison was performed using Akaike's Information Criterion (AIC). The model includes both random intercepts and random slopes (Model III) was evaluated alongside alternative models, with AIC values calculated to assess relative model fit and parsimony.

Ethical approval and consent to participate

This study was based on analysis of existing survey datasets in the public domain (SSA) that are freely available online with all the identifier information anonymized, no ethical approval was required

Result

In this study a total of 134,470 reproductive age women were included in Sub-Saharan African. The Mean age of participants were 28.99 (Std. Dev. 9.68) years. More than two thirds (69.8%) of women did not use contraception, and over 72.22% reproductive women did not use health insurance, and over half of (54.22%) women were living rural areas in sub-Saharan Africa **(Table 2)**

Table 2: Sociodemographic and economic characteristics of reproductive women who had barriers to accessing health care in sub-Saharan Africa.

**Variable**	**frequency(n)**	**percentage (%)**
**Individual and community level variables**		

**Maternal age**		
15-19	27890.4	20.7
20-35	69441.4	51.64
36-49	37138.6	27.64
**Residency**		
Urban	61558.03	45.78
Rural	72912.41	54.22
**Religious status**		
Muslim	48834.95	41.96
Christian	46856.90	39.30
Other	23524.59	19.73
**Educational Status**		
No formal education	35051.70	26.06
Primary education	39651.17	29.48
Secondary and higher education	59767.57	44.44
**Husband educational**		
No formal education	29414.44	36.72
Primary education	21245.78	26.52
secondary and higher education	29428.78	36.74
**Maternal Occupational**		
Not working	50732.90	37.81
working	83417.01	62.19
**Husband Occupation**		
Not working	10390.43	13.00
Working	69698.11	87.00
**Maternal status**		
single	42213.40	31.4
Married	58262.00	43.3
Other	33995.4	25.3
**Contraceptive Utilization**		
No	93855.00	69.8
Yes	40615.5	30.2
**place of delivery**		
Home	8279.46	15.5
Institution	44979.28	84.5
ANC visit		
No visiting	2012.84	3.77
1-4 visiting	24459.34	45.92
>4 visiting	26786.55	50.29

Media exposure

No	34628.45	25.75
Yes	99831.57	74.24
**Intermate utilization**		
No	93020.34	69.17
Yes	41450.10	30.82
**Visiting health facility 12 month**		
No	57137.73	42.49
Yes	77332.71	57.50
**Health insurance**		
No	85044.69	72.22
Yes	32709.39	27.77
**Wealth index**		
poor	47227.18	35.12
middle	25771.45	19.16
Rich	61471.81	45.74
**Region in sub-Saharan Africa**		
East Africa	59786.29	44.46
West Africa	74684.16	55.54
**Community poverty level**		
Low	70776.71	52.63
High	63693.73	47.47
**Community media exposure**		
Low	61642.30	45.84
High	72828.14	54.15
**Community distance health facility**		
Low	65962	49.00
High	68508	51.00
**Community literacy level**		
Low	57615.01	42.84
High	76855.43	57.15
**Community ANC visit**		
Low	77912.32	57.96
High	56491.92	42.03

In this study, the magnitude of barriers to accessing health care among reproductive age women was found to be 55.84 (95% CI: (55.57, 56.10) owing to one or more of the four factors, the most commonly cited being the difficulty in getting money and the distance from medical facilities (**Figure 1)**

Random effect (Measures of variation) and model fitness

To determine if the data supported the choice to evaluate randomness at the community level, a null model was employed. With the variance of 0.3784967 and a p-value of < 0.001, the null model's results showed that there were notable variations in the barriers to accessing healthcare among clusters. A significant amount of the variance in the outcome between clusters was jointly explained by both individual- and community-level variables, as evidenced by the intra-cluster correlation (ICC), which dropped from 10.31% in the null model to 3.96% in the fully adjusted model (model III). According to the proportionate change in variance (PCV), Model III explains the greatest percentage (64.09%) of the group-level variation in comparison to the null model, group comparison showed that, Model I (50.19%) and Model II (24.56%). This suggests that the model fits better as more predictors are included. Model comparison using Akaike's Information Criterion (AIC) indicated that Model III achieved the best fit, as evidenced by the lowest AIC (239,608.6), markedly lower than both the null model (AIC = 1,128,437) and Model II (AIC = 1,127,704). Model I also showed good fit (AIC = 239,764.8), though slightly less optimal than Model III. Multicollinearity using variance inflation factors (VIF) indicated that all VIF values ranged from 1.03-1.98, which is within accepted range **(Table 3)**

Table 3: Model comparison and Fitness random effect analysis for barriers of accessing health care of among reproductive age women in sub-Saharan Africa**.**

Parameter	Null model	model I	model II	model III
Variance	0.3784967	0.1885269	0.28553	0.1358817
ICC	10.31%	5.41%	7.98%	3.96%
MOR	1.79	1.51	1.66	1.41
PCV	Reference	50.19%	24.56%	64.09%
**Model fines**				
LLR	-90175.365	-21798.27	-86841.704	-21433.284
Deviance	1803596.73	43596.54	173683.408	42866.568
AIC	1128437	239764.8	1127704	239608.6

ICC: intra-cluster correlation**, LLR:** loglikelihood ratio**, MOR:** median odds ratio**, PCV:** proportional change in variance. **AIC:** Akaike information criteria.

Determinants of barriers to accessing health care among reproductive age women in Sub-Saharan Africa

Bivariable logistic regression analysis of individual and community level variables associated with barriers to accessing health care among reproductive age women in Sub-Saharan Africa

Bivariable multilevel logistic regression analysis indicated that husband's educational status, media exposure, internet utilization, place of residence, antenatal care attendance, place of delivery, health insurance coverage, community wealth index, recent health facility visits, and country category (Western vs. Eastern Africa) were associated with barriers to accessing health care among reproductive-age women at a p-value < 0.25 (**Table 4**)

Table 4: Bivariable logistic regression of individual and community level factors associated with barriers to accessing health care among reproductive age women in Sub-Saharan Africa.

Variables	Crude Odds Ratio [COR]	P. value	[95% CI]
**Maternal age**			
15-19	0.86	<0.001	[0.83, 0.89]
20-35	0.86	<0.001	[0.84, 0. 88]
36-49	1		1
**Residency**			
Urban	1		1
Rural	2.09	<0.001	[2.04, 2.14]
**Maternal education**			
No education	2.40	<0.001	[2.33, 2.46]
Primary education	1.52	<0.001	[1.48, 1.56]
Secondary & Higher	1		1
**Husband education**			
No education	2.43	<0.001	[2.35, 2.51]
Primary education	1.55	<0.001	[1.49, 1.60]
secondary & higher	1		1
**Maternal occupation**			
working	1		1
not working	0.94	<0.001	[0.92, 0.96]
**Husband occupation**			
working	1		
not working	1.07	<0.001	[1.02, 1.12]
**Religion**			
Muslim	1		1
Christian	1.32	<0.001	[1.29, 1.35]
Other	1.08	<0.001	[1.04, 1.11]

**Contraceptive utilization**			
No	1.18	<0.001	[0.82, 0.86]
Yes	1		1
**Place of delivery**			
Home	1.84	<0.001	[1.75, 1.93]
Institution	1		1
**ANC visit**			
No visit	1.16	<0.002	[1.05, 1.27]
1-4 visit	1.05	<0.003	[1.01, 1.09]
> 4 visits	1		1
**Marital status**			
single	1		
Married	1.23	<0.001	[1.20, 1.26]
Other	1.32	<0.001	[1.29, 1.36]
**Media exposure**			
No	1.90	<0.001	[1.85, 1.95]
Yes	1		1
**Internet utilization**			
No	2.321822	<0.001	[2.26, 2.37]
Yes	1		1
**Visiting health facility 12 month**			
No	1.02	<0.046	[1.00, 1.04]
Yes	1		1
**Health insurance**			
No	1.21	<0.001	[1.18, 1.25]
Yes	1		1
**Wealth index**			
poor	3.58	<0.001	[3.49, 3.67]
middle	2.08	<0.001	[2.02, 2.14]
Rich	1		1
**Region SSA**			
East Africa	1.68	<0.001	[1.65, 1.72]
West Africa	1		1
**community ANC visit**			
Low	0.86	<0.001	[0.85, 0.88]
High	1		1
**community wealth index**			
Low	0.69	<0.001	[0.67, 0.70]
High	1		
**Community media exposure**			
Low	0.73	<0.001	[0.72, 0.75]
High	1		1
**Community literacy level**			
Low	0.77	<0.001	[0.75, 0.79]
High	1		1

Multivariable multilevel logistic regression analysis of individual and community level factors associated with Barriers to health care access among reproductive Age Women in Sub-Saharan Africa.

In the final fitted model of multivariable multilevel logistic regression, Husband educational status, media exposure, place of residency, Antenatal care visit, place of delivery, health insurance utilization, wealth index status, visiting health facility within 12 months, and country category (western, eastern Africa) were significantly associated with barriers of accessing health care among reproductive age women in sub-Saharan Africa. The odds of barrier of accessing health care were 1.30 times higher among reproductive women living in rural residency compared to women living in urban area **[AOR=**1.30, 95 CI: (1.22, 1.39)]. and the odds of barrier of accessing health care were 1.23 times more likely to among reproductive age women whose husband had no education compared to women whose husband had secondary and higher education [**AOR**=1.23, 95 CI: (1.15, 1.30,)]. In addition, odds of barrier of accessing health care were 1.33 times more likely to occur women delivered at home compared to women who had institutional delivery. **(AOR**=1.33, 95% CI: (1.24, 1.43) and the odds of barrier of accessing health care were 1.31 times higher among women who had no history of antenatal visit compared to women who had more than four ANC visit. [**AOR**= 1.31, 95% CI: (1.10, 1.55)]. The odds of the barrier of accessing health care among reproductive age woman who had no media exposure were 1.17 times more likely to compared with a woman who had media exposure **[AOR**=1.17, 95% CI: (1.11, 1.24)] and the barrier of accessing health care among reproductive age woman who were not visiting health facility within 12 months 9% times less likely compared to women who had visiting health care facility within 12 months of study period **[AOR=**0.91, 95% CI: (0.87, 0.97)]. The odds of perceived barrier of accessing health care among reproductive age woman who had no health insurance utilization were 1.18 times more likely to compared with a woman who had used health insurance [**AOR=**1.18 95% CI: (1.11, 1.26)] and The odds of perceived barrier of accessing health care service among reproductive age woman who were classified under poor and middle wealth status were 2.29 and 1.57 times more likely barries accessing health care compared to reproductive women who were classified under rich wealth status category **[AOR**=2.29 95% CI: (2.14, 2.45)] and [**AOR**=1.57, 95% CI: (1.46, 1.68)]. The odds of perceived barrier of accessing health care service among reproductive age woman who reside in west Africa region were 2.38 times more

likely barriers accessing health care compared to east Africa region category. [**AOR**=2.38, 95% CI: (2.21, 2.55)] **(Table 5).**

Table 5: Multivariable multilevel logistic regression of individual and community level factors associated with barriers to accessing health care among reproductive age women in Sub-Saharan Africa.

Individual and community level factors	Model I AOR [95% CI]	Model II AOR [95% CI]	Model III AOR [95% CI]
**Maternal age**			
15-19	0.96 [0.86, 1.07]		0.96 [0.86,1.08]
20-35	1.06 [1.01,1.13]		1.04 [0.99, 1.11]
36-45	1		1
**Residency**			
Urban		1	1
Rural		2.29 [2.24, 2.35]	**1.30 [1.22, 1.39]**
**Maternal education**			
No education	1.10 [1.02, 1.18]		1.04 [0.97, 1.11]
Primary education	0.93 [0.87, 0.99]		1.06 [0.99, 1.14]
Secondary and above	1		1
**Husband education**			
No education	1.26 [1.19,1.35]		**1.23 [1.15, 1.30]**
Primary education	1.04 [0.97,1.12]		1.23 [0.67, 1.22]
secondary & higher education	1		1
**Maternal occupation**			
working	1		1
not working	0.99 [0.94, 1.04]		**1.02 [1.01, 1.11]**
**Husband occupation**			
working	1		1
not working	0.90 [0.83, 0.97]		1.00 [0.92, 1.08]
**Religion**			
Muslim	1		1
Christian	1.62 [1.53, 1.71]		0.46 [0.57, 1.94]
Other	1.22 [1.13, 1.31]		1.36 [0.75, 1.46]
**Contraceptive utilization**			
No	1.11 [1.05,1.17]		1.03 [0.98, 1.08]
Yes	1		1
**Place of delivery**			
home	1.30 [1.21, 1.40]		**1.33 [1.24, 1.43]**
Institution	1		**1**

**Antenatal Care Visit**			
No	1.01 [0.85, 1.19]		**1.31 [1.10, 1.55]**
1-4 visit	0.94 [0.89, 0.99]		1.13 [0.37, 1.19]
> 4 visits	1		1
**Media exposure**			
No	1.14 [1.08, 1.21]		**1.17 [1.11, 1.24]**
Yes	1		1
**Internet utilization**			
No	1.69 [1.58, 1.80]		1.68 [0.67, 1.79]
Yes	1		1
**Visiting health facility 12 month**			
No	0.92 [0.87, 0.98]		**0.91 [0.87, 0.97]**
Yes	1		1
**Health insurance**			
No	1.26 [1.19, 1.34]		**1.18 [1.11, 1.26]**
Yes	1		1
**Wealth index**			
poor	2.80 [2.63, 2.97]		**2.29 [2.14, 2.45]**
middle	1.79 [1.68, 1.92]		**1.57 [1.46, 1.68]**
Rich	1		1
**Region SSA**			
East Africa		1	**1**
West Africa		2.0 [1.92-2.04]	**2.38 [2.21, 2.55]**
**community ANC visit**			
Low		1.00 [0.93, 1.09]	0.93 [0.86, 1.00]
High		1	1
**community wealth index**			
Low		0.75 [0.70, 0.81]	**1.12 [1.02, 1.22]**
High		1	1
**Community media exposure**			
Low		1.01 [0.92, 1.10]	0.94 [0.86, 1.03]
High		1	1
**Community literacy level**			
Low		1.10 [1.00, 1.20]	**1.13 [1.03, 1.24]**
High		1	**1**

Model I: *individual level variable,* **Model II:** *Community level variable,* **Model III**: *Individual and community level variable*

Discussion

Barriers to accessing health care have contributed to maternal and child mortality and morbidity in developing countries such as those in Sub-Saharan Africa. This study aimed to assess the

magnitude and determinants of barriers to health care access among women of reproductive age in Sub-Saharan Africa.

In this study, the magnitude of barriers to accessing health care among reproductive age women was found to be 55.84 (95% CI: (55.57, 56.10) owing to one or more of the four factors, the most commonly cited being the difficulty in getting money and the distance from medical facilities. This finding is lower than the previous study conducted in South Africa 65% [18, 19], and Tanzania 65% [15], Ethiopia 71.8%,70.2, 69.9%[1, 20, 21], (SSA) 60.4%, 61.5% [17]. this variation could be due to the fact that socio-economic and variability in health infrastructure and health system policy, women's attitudes towards health care seeking behavior and cultural differences across countries in sub-Saharan Africa, moreover, this discrepancy might be due to study period, this study includes, the most recent DHS data set from 2019-2023. On the other hand, the finding of this study is similar to but slightly higher than in previous study in Gambia 45.5%[22], Ghana (51%)[23]. this variation could be due to the fact that socio-economic and variability in health infrastructure and health system policy, women's attitudes towards health care seeking behavior and cultural differences across countries in sub-Saharan Africa.

The odds of barrier of accessing health care were 1.30 times higher among reproductive women living in rural residency compared to women living in urban area. This finding is supported by previous studies conducted in Ethiopia [1, 20], guinea[24], Gambia, Tanzania and Ghana [15, 22, 25] respectively. Sub-Saharan[23], East Africa[26]. This association may be, due to the fact that rural areas have low health infrastructure i.e. inaccessibility of health facilities, less privileged and the influences of socio-cultural practices that need women to seek permission from their husband to visit the facility or women's decisions for seeking healthcare services is under their husband/partners control. Moreover, male involvement and support for women health care access are limited[1, 14, 23, 26]. This implies that the government should expand infrastructure services in rural area and encourage male involvement and support for improving health care access for reproductive age women[14].

The odds of perceived barrier of accessing health care were 1.23 and 1.21 times more likely to among reproductive age women whose husband had no education and primary education compared to women whose husband had secondary and above educational level respectively. This find was consistent with previous study conducted in east Africa, Ethiopia, Tanzania, Benin,

Bangladesh respectively [1, 15, 20, 26, 27]. The possible explanation could be in better access to the healthcare among women with educated husbands might be due to better participation and involvement of husbands in their families' health[23]. Women whose partners are educated are also likely to be informed regarding their fundamental human rights and may have higher health literacy. As a result, they are more likely to deal with any form of barrier to healthcare, compared to their counterparts who are less educated and may have lower health literacy [14, 15, 20, 28].

The odds of barrier to accessing health care were 1.33 times more likely to occur women delivered at home compared to women who had institutional delivery. This evidence was supported by study done in Senegal[29]. The plausible justification might be women who delivered at home may not get health information/counselling from healthcare providers that empower them to use other maternal healthcare services and women who deliver at home may face varous barriers to accessing health care such as financial constraint, lack of transportation, limited autonomy in healthcare decision and sociocultural norms that discourage institutional delivery. Moreover, importance of enhancing health system outreach, strengthen community-based education, and addressing sociocultural and socioeconomic disparities that affect health seeking behavior. On the other hand, the odds of barrier of accessing health care among woman who had no media exposure were 1.17 times more likely to compared with a woman who had media exposure. This evidence was supported by study done in Ethiopia[30], Nepal[31]. This could be the power of mass media in disseminating information concerning maternal health that may enhance women's knowledge and attitude towards health care access and utilization[31]. Barrier of accessing health care among woman who were not visiting health facility within 12 months were 9 % times less likely compared to women who had visiting health care facility within 12 months of study period. The odds of barrier to accessing health care among woman who had no health insurance utilization were 1.18 times more likely to compared with a woman who had used health insurance. This finding was supported with study done in Ethiopia[1], sub-Saharan Africa[23], Tanzania[15], Ghana[25]. The possible reason might be due to the fact that, individuals who were not had health insurance faced for unexpected catastrophic expenditure and difficulties in obtaining money for consultation of the doctor[1]. The odds of barrier to accessing healthcare among woman who were classified under poor and middle wealth status were 2.29 and 1.57 times more likely accessing health care difficulty compared to women who were classified under rich wealth index category**.** This evidenced was supported with study done in Ethiopia, [1, 30, 32, 33] Tanzania[15] Uganda[34], sub Saharan

Africa[14]. This finding might be explained by the fact that high out-of-pocket expenses and a lack of accessibility to healthcare facilities pose significant obstacles for middle-class and impoverished women in sub-Saharan Africa. Many people in the region cannot afford healthcare services because over 40% of health costs are paid for directly by individuals[35-38]

The odds of barrier of accessing health care among woman who live west Africa region were 2.38 times more likely barriers to accessing health care compared to east Africa region. According a recent joint report from the World Health Organization and the United Nations states that high out-of-pocket costs, a lack of adequate healthcare infrastructure, and other sociocultural barriers make it more difficult for women of reproductive age in West African nations such as Nigeria, Mali, and Sierra Leone to access maternal healthcare. On the other hand, East African countries like Rwanda, Kenya, and Ethiopia have strengthened their community health initiatives and gender-inclusive policies, which have improved women's autonomy in accessing healthcare services[39]. Furthermore, our current findings are supported by recent Demographic and Health Survey (DHS) program data (2022–2023), which indicate that women in West African countries are 2-3 times more likely to report delays in accessing healthcare due to cost, shortages of healthcare personnel, and long distances to health facilities. In contrast, East African countries, particularly Uganda and Tanzania, have strengthened their health insurance systems and rural outreach initiatives. Additionally, the World Bank's 2023 Health Service Coverage Index reveals that women in West Africa have significantly lower access to family planning, skilled birth attendance, and other essential reproductive health services[22, 40, 41]

Strength and limitation

This strength of study nationally representative data to evaluate contextual and individual characteristics related to barriers to healthcare access among reproductive age women in the SSA. Thus, findings can therefore, generalized to all women in their reproductive age in SSA, in order to account for the clusters within the sample, the study also employed advanced statistical models. This study has several limitations. The cross-sectional design restricts the ability to draw causal inferences, and health worker–related factors were not incorporated into the analysis. Furthermore, reliance on self-reported data may introduce recall or social desirability bias; future research should consider integrating objective measures, such as wearable devices, as demonstrated by Zhao et al. (2025)[42]

Conclusion and Recommendation

This study found that 55.55% of reproductive age women had barriers to accessing healthcare in SSA. Husband educational status, media exposure, internet utilization, place of residency, ANC visit status, place of delivery, health insurance utilization status, wealth index status, visiting health facility within 12 months, community literacy level and community wealth index and country category (western, eastern Africa) were significantly associated with barriers of accessing health care. Therefore, the Government and ministry of health in Sub-Saharan Africa countries should taking in to consideration those factors while designing policies and strategies targeting to health care access and tackling the preventable barriers to accessing health care in sub-Saharan Africa.

Declaration

Ethical approval and consent to participate

This study was based on analysis of existing survey datasets in the public domain (SSA) that are freely available online with all the identifier information anonymized, no ethical approval was required

Consent for publication

Not applicable

Data and material availability

Demographic and Health Survey were used, and it is publicly available online at (https://www.dhsprogram.com).

Competing interest

Authors Declared that, there is no competing interest.

Funding

Not applicable

Author's contributions

GAZ: involved in designing, data extraction, data analysis, interpretation, report and manuscript writing.

BT: involved in data analysis, interpretation, and, and manuscript writing.

BT: involved in analysis,

TTT: involved in designing the study, interpretation, report and

BSW: involved in analysis, and result interpretation

AFZ: involved in designing and interpretation study

EGM: analysis and manuscript writing

MW: Result interpretation

AA: manuscript writing: involved in data extraction

MSA: involved manuscript writing

Acknowledgements

We would like to say the DHS programs for allowing us to use the relevant DHS dataset in this study.

Reference

1. Tamirat, K.S., Z.T. Tessema, and F.B.J.B.H.S.R. Kebede, Factors associated with the perceived barriers of health care access among reproductive-age women in Ethiopia: a secondary data analysis of 2016 Ethiopian demographic and health survey. 2020. **20**: p. 1-8.
2. Erasmus, M.O., The barriers to access for maternal health care amongst pregnant adolescents in the Mitchells plain sub-district. 2017.
3. Washington, D.L., et al., Access to care for women veterans: delayed healthcare and unmet need. 2011. **26**: p. 655-661.
4. Regan, L.J.B.M.A., Addressing unmet needs in global women's health. 2018.
5. Izugbara, C., E. Covan, and E. Fugate-Whitlock, Women's health in Africa: Issues, challenges and opportunities. 2017: Routledge.
6. Organization, W.H., Primary health care on the road to universal health coverage: 2019 global monitoring report: Executive summary. 2019, World Health Organization.
7. Badiuzzaman, M., S.M. Murshed, and M.J.T.J.o.D.S. Rieger, Improving maternal health care in a post conflict setting: evidence from Chittagong Hill tracts of Bangladesh. 2020. **56**(2): p. 384-400.
8. Peters, D.H., et al., Poverty and access to health care in developing countries. 2008. **1136**(1): p. 161-171.
9. Alkire, B.C., et al., The economic consequences of mortality amenable to high-quality health care in low-and middle-income countries. 2018. **37**(6): p. 988-996.
10. Nyakang'o, S.B. and A.J.M. Booth, Women's perceived barriers to giving birth in health facilities in rural Kenya: A qualitative evidence synthesis. 2018. **67**: p. 1-11.
11. Colglazier, W.J.S., Sustainable development agenda: 2030. 2015. **349**(6252): p. 1048-1050.

12. Cahill, C.M., et al., Preoperative anemia management program reduces blood transfusion in elective cardiac surgical patients, improving outcomes and decreasing hospital length of stay. 2021. **61**(9): p. 2629-2636.
13. Peeri, N.C., et al., The SARS, MERS and novel coronavirus (COVID-19) epidemics, the newest and biggest global health threats: what lessons have we learned? 2020. **49**(3): p. 717-726.
14. Tessema, Z.T., et al., Determinants of accessing healthcare in Sub-Saharan Africa: a mixed-effect analysis of recent Demographic and Health Surveys from 36 countries. 2022. **12**(1): p. e054397.
15. Bintabara, D., K. Nakamura, and K.J.B.o. Seino, Improving access to healthcare for women in Tanzania by addressing socioeconomic determinants and health insurance: a population-based cross-sectional survey. 2018. **8**(9): p. e023013.
16. Kea, A.Z., et al., Exploring barriers to the use of formal maternal health services and priority areas for action in Sidama zone, southern Ethiopia. 2018. **18**: p. 1-12.
17. Zegeye, B., et al., Breaking barriers to healthcare access: a multilevel analysis of individual-and community-level factors affecting women's access to healthcare services in Benin. 2021. **18**(2): p. 750.
18. Silal, S.P., et al., Exploring inequalities in access to and use of maternal health services in South Africa. 2012. **12**: p. 1-12.
19. Tsawe, M. and A.S.J.B.r.n. Susuman, Determinants of access to and use of maternal health care services in the Eastern Cape, South Africa: a quantitative and qualitative investigation. 2014. **7**: p. 1-10.
20. Zegeye, B., et al., Barriers and facilitators to accessing health care services among married women in Ethiopia: a multi-level analysis of the Ethiopia demographic and health survey. 2021.
21. Demsash, A.W., B. Chakilu, and A. Mazengia, Knowledge Sharing Practice and Its Associated Factors Among Healthcare Providers at University of Gondar Comprehensive Specialized Hospital, North West Ethiopian: Cross-sectional Study. 2021.
22. Tsegaw, M., B. Mulat, and K.J.B.o. Shitu, Problems with accessing healthcare and associated factors among reproductive-aged women in the Gambia using Gambia demographic and Health Survey 2019/2020: a cross-sectional study. 2023. **13**(8): p. e073491.
23. Seidu, A.-A.J.P.O., Mixed effects analysis of factors associated with barriers to accessing healthcare among women in sub-Saharan Africa: Insights from demographic and health surveys. 2020. **15**(11): p. e0241409.
24. Seidu, A.-A., et al., Individual and contextual factors associated with barriers to accessing healthcare among women in Papua New Guinea: insights from a nationwide demographic and health survey. 2021. **13**(6): p. 573-585.
25. Seidu, A.-A., et al., Barriers to accessing healthcare among women in Ghana: a multilevel modelling. 2020. **20**: p. 1-12.
26. Minyihun, A., Z.T.J.R.M. Tessema, and H. Policy, Determinants of access to health care among women in East African countries: a multilevel analysis of recent demographic and health surveys from 2008 to 2017. 2020: p. 1803-1813.
27. Sen, K.K., et al., Do education and living standard matter in breaking barriers to healthcare access among women in Bangladesh? 2023. **23**(1): p. 1431.

28. Dimbuene, Z.T., et al., Women's education and utilization of maternal health services in Africa: a multi-country and socioeconomic status analysis. 2018. **50**(6): p. 725-748.
29. Zegeye, B., et al., Predictors of institutional delivery service utilization among women of reproductive age in Senegal: a population-based study. 2021. **79**: p. 1-11.
30. Demsash, A.W., A.D.J.B.H. Walle, and C. Informatics, Women's health service access and associated factors in Ethiopia: application of geographical information system and multilevel analysis. 2023. **30**(1).
31. Tamang, T.M. Factors associated with completion of continuum of Care for Maternal Health in Nepal. in IUSSP XXVIII International Population Conference, Cape Town, South Africa. 2017.
32. Fentie, E.A., et al., Spatial distribution and determinants of barriers of health care access among female youths in Ethiopia, a mixed effect and spatial analysis. 2023. **13**(1): p. 21517.
33. Alamneh, T.S., et al., Barriers for health care access affects maternal continuum of care utilization in Ethiopia; spatial analysis and generalized estimating equation. 2022. **17**(4): p. e0266490.
34. Kalule-Sabiti, I., A.Y. Amoateng, and M.J.A.P.S. Ngake, The effect of socio-demographic factors on the utilization of maternal health care services in Uganda. 2014: p. 515-525.
35. Shiferaw, S., et al., Why do women prefer home births in Ethiopia? 2013. **13**: p. 1-10.
36. Yaya, S., G. Bishwajit, and V.J.B.g.h. Shah, Wealth, education and urban–rural inequality and maternal healthcare service usage in Malawi. 2016. **1**(2): p. e000085.
37. Voto, T.P., B.E. Voto, and N.J.E. Ngepah, The Impact of Out-of-Pocket Health Expenditure and Public Health Expenditure on Poverty in Sub-Saharan Africa. 2025. **13**(5): p. 134.
38. Alamneh, T.S., et al., Socioeconomic inequality in barriers for accessing health care among married reproductive aged women in sub-Saharan African countries: a decomposition analysis. 2022. **22**(1): p. 130.
39. AKINWALE, A.A.J.I.J.o.I.R. and A. Studies, Gender equity and social progress: empowering women and girls to drive sustainable development in Sub–Saharan Africa. 2023.
40. Arhin, K., E.F. Oteng-Abayie, and J.J.H.e.r. Novignon, Assessing the efficiency of health systems in achieving the universal health coverage goal: evidence from Sub-Saharan Africa. 2023. **13**(1): p. 25.
41. Muhajarine, N., et al., Inequalities and factors associated with maternal healthcare services utilisation in Mozambique: evidence from the Demographic and Health Survey 2022−2023. 2025. **10**(5).
42. Zhao, Y., et al., Highly sensitive, wearable piezoresistive methylcellulose/chitosan@ MXene aerogel sensor array for real-time monitoring of physiological signals of pilots. 2025. **68**(2): p. 542-551.

Figure legend

Fig 1: Barriers to accessing health care and determinant factor among reproductive age women in sub-Saharan Africa

 Click here to access/download;Figure;Figure 1 hc.docx

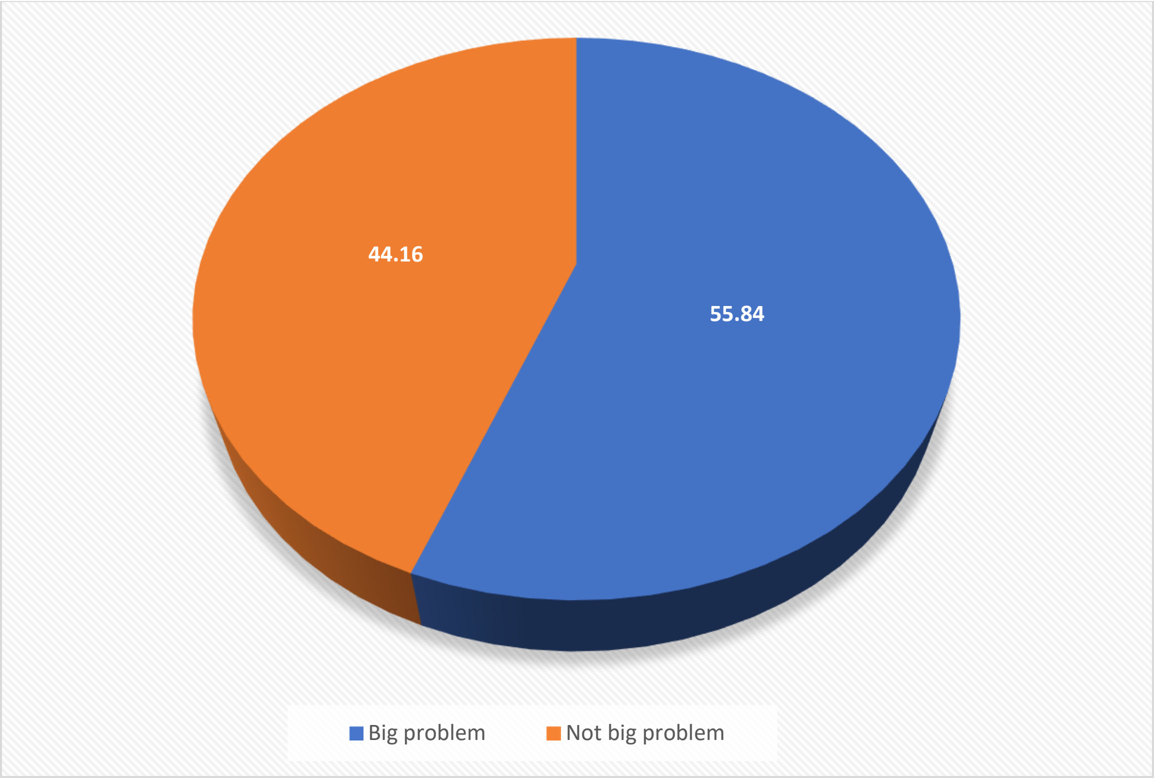

Figure 1: Barriers to accessing health care and determinant factor among reproductive age women in sub-Saharan Africa**.**

Click here to access/download
Supporting Information
VIF AND AIC AND BIC table.docx

Title: Barriers to accessing health care among women's in Sub-Saharan Africa: Multilevel Analysis of Recent Demographic and Health Survey from 2019-2023.

Formatted: Justified

Gebreeyesus Abera Zeleke[1]*, Enyew Getaneh Mekonen[1], Bewuketu Terefe[2], Mulugeta Wassie[3], Agazhe Aemro[3], Alebachew Ferede Zegeye[3], Mohammed Seid Ali[4], Berhan Tekeba[4], Tadesse Tarik Tamir[4], Belayneh Shetie Workneh[5]

[1] Department of Surgical Nursing, School of Nursing, College of Medicine and Health Sciences, University of Gondar, Gondar, Ethiopia

[2] Department of Community Health Nursing, School of Nursing, College of Medicine and Health Sciences, University of Gondar, Gondar, Ethiopia.

[3] Department of Medical Nursing, School of Nursing, College of Medicine and Health Sciences, University of Gondar, Gondar, Ethiopia;

[4] Department of Pediatrics and Child Health Nursing, School of Nursing, College of Medicine and Health Sciences, University of Gondar, Gondar, Ethiopia.

5 Department of Emergency and Critical Care Nursing, School of Nursing, College of Medicine and Health Sciences, University of Gondar, Gondar, Ethiopia

Correspondence Author*
Gebreeyesus Abera Zeleke

Department of Surgical Nursing, School of Nursing, College of Medicine and Health Science, University of Gondar, Gondar, Ethiopia.

Email address: gebreeyesusabe143@gmail.com

Co-authors:

Enyew Getaneh Mekonen **Email Address:** enyewgetaneh12@gmail.com

Bewuketu Terefe: **Email address**: woldeabwomariam@gmail.com

Mulugeta Wassie: **Email address:** mulugeta2113@gmail.com

Agazhe Aemro: **Email address:** agazhe1049@gmail.com

Alebachew Ferede Zegeye: **Email address:** alexferede24@gmail.com

Mohammed Seid Ali: **Email address:** muheseid2592@gmail.com

Berhan Tekeba: **Email address:** berishboss7@gmail.com

Tadesse Tarik Tamir: **Email address:** tadestar140@gmail.com

Belayneh Shetie Workneh: **Email address:** belay2319@gmail.com

Abstract

Background:

~~Introduction:~~

Barriers to accessing healthcare have ~~played a significant role in~~significantly contributed to the high rates of maternal and child mortality and morbidity in developing regions, particularly in Sub-Saharan Africa. Access to healthcare is shaped by multiple factors, including financial constraints, geographic location, the availability of services, and the quality of care provided. This study aimed to assess the ~~extent~~magnitude of these barriers and to identify the factors influencing healthcare access among women of reproductive age in Sub-Saharan Africa.

Methods: ~~Secondary~~

This study utilized secondary data ~~analysis was conducted using~~ from the most recent Demographic and Health Surveys~~, study from~~ conducted between 2019~~-~~ and 2023 ~~in~~across Sub-Saharan Africa. ~~In this study, the total~~A weighted sample of ~~134470~~134,470 women of reproductive age ~~women weighted sample~~ was included in the analysis. Data were ~~employed.~~ analyzed using Stata version 14~~ was used to analyze the data and~~, employing a multilevel mixed-effects logistic regression model~~ was and Significant factors~~. Factors associated with ~~barrier~~barriers to ~~health care~~healthcare access ~~in the multilevel mixed-effect logistic regression model~~ were ~~declared~~considered statistically significant at ~~p~~P-values~~ <~~ less than 0.05. ~~The adjusted~~Adjusted odds ~~ratio (AOR) and~~ratios (AORs) with 95% confidence ~~interval (CI~~intervals (CIs) were used to interpret the results.

Result: In this study, the magnitude of barriers to accessing ~~health care~~healthcare among reproductive age women ~~was~~were found to be ~~[~~55.84% (95% CI: ~~(~~55.57~~,~~ 56.10~~)]. Individual and community level factors such as women's husband~~). Husband's educational status [AOR~~=[~~ = 1.23, 95% CI: ~~(~~1.15 1.30~~, 1.15)]~~,]. Media exposure [AOR~~=[~~ = 1.17, 95% CI: ~~(~~1.11~~,~~ 1.24~~)],~~], antenatal care (ANC) visit status [AOR = 1.31, 95% CI: ~~(~~1.10~~,~~ 1.55~~)]~~], and place of delivery [AOR~~=[~~ = 1.33, 95% CI: ~~(~~1.24~~,~~ 1.43~~)], health~~]. Health insurance utilization ~~status~~ [AOR~~=~~ = 1.18, 95% CI: ~~(~~1.11~~,~~ 1.26~~)]~~,], wealth index status, and visiting a health facility within the past 12 months [AOR~~=~~ = 0.91, 95% CI~~ (~~: 0.87~~,~~ 0.97~~)],~~] were individual associated variable. In addition, place of

Formatted: Font: 18 pt, Font color: Text 1

Formatted: Normal

Formatted: Font: Bold

Formatted: Font color: Accent 1

Formatted: Normal

Formatted: Font: Times New Roman, 12 pt

residency, residence [AOR = 1.30, 95% CI: (1.22, –1.39)],], community -literacy level [AOR= = 1.13 955, 95% CI: (1.03,–1.24)] and], community wealth index [AOR= = 1.12, 95% CI: (1.02,–1.22)]], and country category (western, eastern Africa)Region were significantly associated with barriers ofto accessing health care among reproductive age women in sub-Saharan Africa.healthcare.

Formatted: Font: Times New Roman, 12 pt, Font color: Auto

Conclusion and recommendation: This study found that 55.55% of women ~~had~~in Sub-Saharan Africa experienced barriers to accessing healthcare~~ in SSA.~~. Educational status, media exposure, place of ~~residency, ANC visit status~~residence, antenatal care attendance, place of delivery, health insurance coverage, wealth index, ~~visiting~~recent visits to health ~~facility within 12 months~~facilities, community literacy ~~level and~~levels, community wealth ~~index~~status, and ~~country region~~regional differences were ~~significant~~determinant factors~~ with barriers of accessing health care. Therefore;~~. These findings highlight the ~~Governments in~~need for Sub-Saharan African ~~countries should focus on addressing~~governments to prioritize these ~~factors~~determinants when ~~developing~~formulating policies and strategies aimed at ~~overcoming~~reducing preventable barriers to healthcare access in the region.

Key words: ~~Accessing Health care,~~Access to Healthcare; Barriers~~,~~ to Healthcare; Sub-Saharan Africa~~,~~; Reproductive ~~women.~~Age Women

Formatted: Font color: Accent 1

Formatted: Font color: Auto

Formatted: Font: Times New Roman, Font color: Auto

Introduction~~Background~~

Access to ~~health care~~healthcare is broadly defined ~~promoting~~as the ability to prevent illness, disability, and ~~sustaining~~premature death, to promote and maintain health, ~~controlling illnesses, preventing disabilities and early deaths,~~ and ~~attaining~~to attain health equity, particularly for women of reproductive age ~~achieving based on quality and~~, by offering inclusive ~~healthcare access and optimal health care~~, high-quality care and the best possible availability, affordability, and accessibility of ~~the~~ medical ~~care~~services~~(~~[1-3~~)~~]. ~~The~~Reproductive-age women's health ~~of women of reproductive age continues to be~~is a ~~significant~~critical global public health ~~issue, as their well-being is closely linked to the overall health and prosperity of society(4). The health of women of reproductive age, particularly in Africa, plays a vital role in driving~~concern since it influences population health outcomes and fosters socioeconomic growth[4]. The advancement of socioeconomic development~~. (5). The Sustainable Development Goals (SDGs) prioritize~~ is significantly influenced by the health of women of reproductive age, especially in Africa. [5]. The Sustainable Development Goals (SDGs) place a strong emphasis on women's health, with Targets 3.7 and 3.8 of SDG~~-~~ 3 specifically ~~emphasizing the thing of~~focusing on achieving universal health ~~content~~coverage by 2030 and ~~icing~~ensuring access to sexual and reproductive health services, including education and information on family planning ~~(6, 7). The SDGs specifically state that the goal is to significantly reduced rate of maternal death,~~ [6, 7]. The Sustainable Development Goals (SDGs) explicitly target a substantial reduction in maternal mortality, with Goal 3.1 ~~calling for~~aiming to lower the global maternal mortality ~~rate~~ratio to ~~be reduced to less~~fewer than 70 per 100,000 live births by 2030. ~~Although~~While these global efforts have ~~brought~~contributed to some improvements ~~to~~in women's health outcomes, they have ~~not~~yet to fully ~~resolved the underlying issues. More work is needed to achieve~~address the root causes of maternal mortality. Achieving sustained and comprehensive ~~and lasting~~ progress ~~in this area.~~remains a critical challenge. In 2016, Sub-Saharan Africa accounted for a disproportionately high ~~number~~share of the 303,000 maternal deaths ~~worldwide, with~~ reported globally, the majority ~~resulting from~~of which were due to

Formatted: Font: +Body (Calibri), 11 pt, Not Bold

Formatted: Normal

Formatted: Font: 18 pt, Font color: Text 1

Formatted: Font: Arial, Font color: Custom Color(RGB(59,59,59))

Formatted: Font: +Body (Calibri), 11 pt

Formatted: Font color: Custom Color(RGB(59,59,59))

Formatted: Font color: Custom Color(RGB(59,59,59))

Formatted: Font color: Custom Color(RGB(59,59,59))

Formatted: Font color: Custom Color(RGB(59,59,59))

Formatted: Font color: Auto, Pattern: Clear

preventable ~~disease. Additionally~~causes. Moreover, non-communicable diseases ~~have caused the deaths of numerous women~~continue to contribute significantly to female morbidity and mortality across nearly ~~every country~~all countries, with ~~many more left disabled,~~a particularly severe impact in low- and middle-income ~~countries. (4-6).~~settings, where many women are left with long-term disabilities [4-6]. Access to healthcare involves multiple dimensions, including financial affordability, geographic ~~location, the~~accessibility, availability of medical services, and the quality of care ~~delivered in health facilities (8).~~provided in health facilities. [8]. Access to healthcare is ~~the~~a key determinant of whether individuals utilize health services. Globally, approximately 400 million people ~~are without~~lack essential healthcare, and around eight million die each year from preventable diseases—resulting in an estimated economic loss of $6 trillion ~~for~~in low- and middle-income countries. ~~(~~[6, 9, 10~~)~~]. In 2015, world leaders pledged to achieve universal health coverage as part of the Sustainable Development Goals (SDGs), ~~which aim~~aiming to ensure access to affordable, high-quality medicines and ~~provide~~ financial protection for all ~~(11, 12).~~[11, 12]. In 2017, the World Bank and WHO reported that half of the global population lacks access to essential health services. As a result, many countries in Sub-Saharan Africa have made universal healthcare a key part of their national health strategies. However, there has been slow progress in turning this commitment into tangible results, particularly in securing national funding for equitable, high-quality healthcare, financial security, and overall health ~~(~~[6, 9, 13, 14~~)~~].

Reproductive age women and child mortality from low family planning uptake, unsafe abortion practices, unintended pregnancies, and an increase in home births are all implications of women in reproductive age having trouble accessing health care~~(14).~~ [14]. Previous research has offered some evidence regarding the sociodemographic and economic factors that can be barriers to accessing healthcare, such as marital status, place of residence, ~~mother~~maternal age, wealth index, educational attainment, and unemployment status. Nevertheless, women of reproductive age continue to face significant disparities in healthcare utilization and access~~(~~[10, 14-16~~)~~]~~. Different empirical evidence related to~~. This study makes three unique contributions to the body of existing literature. In order to provide a current and policy-relevant analysis of the barrier to healthcare access, it first makes use of the most recent (2019–2023) Demographic and Health Survey (DHS) data. Second, our analysis takes a harmonized multi-country approach across ten sub-Saharan African nations, improving cross-national

Formatted: Font: Times New Roman, 12 pt

Formatted: Font: Times New Roman, 12 pt

Formatted: Font: Times New Roman, 12 pt

Formatted: Space After: 10 pt

comparability and generalizability in contrast to many previous studies that are limited to single-country analyses or rely on older datasets. In order to gain a better understanding of the structural and contextual factors that influence healthcare access~~ among reproductive age women has been explored~~, the study employs a comprehensive multilevel analysis of determinants at the ~~country level~~individual and ~~multicounty~~community levels. These contributions put our research in ~~Africa. However, there are limited studies that have incorporated the recent demographic health survey data sets~~a position to guide focused, empirically supported programs provide fair access to healthcare for reproductive age women in sub-Saharan~~ Africa. Therefor~~. Therefore, this study aimed to ~~generate~~ascertain the magnitude and potential contributing factors associated with ~~barrier~~barriers to accessing ~~ health care~~healthcare among ~~the~~women of reproductive age ~~women~~ in sub-Saharan Africa.

Methods and materials

Formatted: Font: 18 pt

Patient and public involvement statement

Secondary data from the Demographic and Health Surveys (DHS) were used in this investigation.

Study design, study area, and period.

Formatted: Justified

Data from ~~the~~ ten ~~sub~~Sub-Saharan African countries, collected between 2019 and 2023, were used to ~~conduct~~perform a multilevel mixed~~ effect~~ effects analysis, ~~following~~based on the most recent ~~demographic health study. Data from~~Demographic and Health Surveys (DHS). The DHS collects community-based, cross-sectional ~~research is gathered by the DHS~~data approximately every five years to ~~create new~~generate updated health and health-related indicators.

Formatted: Font: +Headings (Calibri Light), 13 pt, Font color: Accent 1

Data source, study population and sampling technique

A secondary data analysis was ~~carried out~~conducted using Demographic and Health Survey (DHS) data collected between 2019 and 2023 from selected sub-Saharan African countries. The study specifically included DHS datasets from ten countries~~:~~ Burkina Faso, Gabon, Ghana, Kenya, Liberia, Rwanda, Sierra Leone, Tanzania, The Gambia, and Mozambique~~,~~ each of which had recent and publicly available ~~and recent~~ survey data within the specified timeframe. The datasets were appended to ~~examine~~assess the extent of, and factors underlying~~ factors associated with,~~ barriers to healthcare access among women of reproductive age in sub-Saharan Africa. Each ~~country's survey includes~~country's DHS comprises multiple datasets. The DHS employs a

stratified, two-stage cluster sampling design~~, where~~: in the first stage~~ involves selecting,~~ enumeration areas are selected, followed by ~~a second stage in which~~systematic sampling of households ~~are systematically sampled ~~within each selected area in the second stage.

The variable barrier to accessing health care were generated by computation of (v467b, v467c, v467d, v467f) from maternal recode (IR) data set was recoded. A binary logistic regression model was used to identify determinants a factor barrier to access the health care. Determinants of barrier to accessing the health care were reported in terms of an AOR with a significance level of (95%). In the univariate analysis, at 95% confidence intervals with a p-value of < 0.25 was considered for the candidate multivariable analysis of data. All variables with p values of <0.05 were considered statistically significant. Since DHS data are nationally representative, stratified multistage cluster sampling is required; therefor, sampling weights must be applied to produce unbiased estimates. To correctly weight DHS data, we use the sampling weights provided in the dataset, specifically the individual weight **(**v005**)** for women's individual recode files. This weight must be divided by 1,000,000 (v005/1000000). using "gen wgt=v005/1000000 Stata commands. A total weighted sample of 134,470~~.~~ reproductive age women were included in the study **(Table 1).**

Formatted: Font: Bold

Table 1: The weighted Sample size for magnitude and determinants of barrier to accessing health care among reproductive age women in sub-Saharan Africa**.**

**Country**	**Year of survey**	**weighted sample (n)**	**weighted sample (%)**
Brukinefaso	2021	17,659	13.13
Gabon	2019-2021	6,507.15	4.83
Ghana	2022	15,014	11.16
Kenya	2022	16,716.36	12.43
Liberia	2019-2020	8,065	5.99
Rwanda	2019-2020	14,634	10.88
Sera lion	2019	15,574	11.58
Tanzania	2022	15,254	11.34
Gambia	2019-2020	11,865	8.08
Mozambique	2023	13,181.93	9.80
TOTAL		134,470	100%

Study variable

Outcome variables: The outcome of this study was barrier to access the health care. The Data in barrier of accessing the health care were collected among reproductive age women who used one of the health cares. Measurement of the outcome variables was determined by using computation of four variables~~.~~; such as Distance to health facility~~,~~ (v467b), difficulty in obtaining money~~,~~ (v467c), getting permission, ~~for treatment~~go to the doctor (v467d), and not wanting to go alone ~~were computed.~~(v467f). If a woman of reproductive age ~~women faced~~reported experiencing at least one ~~or more of the problems, women were considered to have serious barriers to healthcare access and coded as “1” and written~~of the following challenges, such as ~~big problem, whereas, if women did not face any problem~~difficulty in ~~the four factors such as (getting~~obtaining money, distance to ~~the~~a health facility, ~~getting~~lack of permission ~~for~~to seek treatment, ~~not wanting~~or reluctance to go alone~~),~~ were considered ~~not~~to face significant barriers to healthcare access. These cases were coded as ‘1’ and labeled as ‘big problem.’ Conversely, women who did not report any of these four barriers were considered to have no significant barriers to healthcare access~~ and~~. These were coded as ~~“0” and not beg problem.~~ ‘0’ and labeled as ‘no big problem’. This approach is consistent with prior literature using DHS data[17].

Formatted: Font: Bold, Font color: Accent 1

Formatted: Strong, Font: +Headings (Calibri Light), Font color: Accent 1

Formatted: Font: Times New Roman, Font color: Auto

Independent Variable: Hence DHS data are hierarchical, independent factors come from two sources that was at individual and community levels factors were considered for this analysis. The individual-level independent variables were women's age (15-19, 20-35, 36-49), women educational status (No education, Primary education , Secondary and Higher education), women occupation (working, not working), Religion (Muslim, Christian , Others), Wealth index (Poor, Middle, media exposure (yes, No), internet utilization (yes, No), Residency (urban, rural), Husband educational status (No education, Primary education, secondary and higher education), Husband occupational status (No working, Working), Currently Contraceptive (No, Yes) Visiting health facility in 12 month (No, Yes), wanted pregnancy(yes, no) .

The community-level variables: Community poverty level (Low, High), Community literacy level (Low, High), Community media exposure, (Low, High), ~~(Low, High),~~ Place of residency (Urban, rural), Region (East, west) in sub Saharan Africa.

Data processing and statistical analysis

Stata version 14 statistical software was used to clean, record, and analyze secondary DHS data sets. The variables in the DHS data are grouped into clusters, and the variables within a cluster are more similar to each other than those in other clusters. The presumptions of equal variance across clusters and independent data were violated in order to run a typical logistic regression model. This suggests that between-cluster factors must be considered using a complex model.

Given this, multilevel mixed-effects logistic regression was used to determine the factors that associated with barrier to accessing the health care. Multilevel mixed effect logistic regression follows four models: the null model (only outcome variable), mode I (individual level variables), model II (community level variables), and model III (both individual and community level variables). The null model without independent variables was used to check the variability of barrier accessing the health careoutcome variable across the cluster. The relationship of individual-level variables with outcome variable (Model I) and the association of community-level variables with outcome variable (Model II) were determined. In the final model, the association of both individual and community-level variables was fitted with the outcome variable.

Random effects

The intra-class correlation coefficient (ICC), proportionate change in variance (PCV), and median odds ratio (MOR) were used to quantify random effects or measures of variation of the outcome variables.

To assess the variation between clusters, the proportionate change in variance (PCV) and intra-cluster correlation coefficient (ICC) were calculated. By treating the cluster as a random variable, the ICC shows that the variation in the health care access barrier between clusters may be computed as follows; $ICC = \frac{VC}{VC+3.29} \times 100\%$. The MOR, which is determined by using clusters as a random variable and calculating the median value of the odds ratio between the highest-risk and lowest-risk areas for health care access barriers, is calculated;

MOR= $e^{0.95\sqrt{VC}}$. Furthermore, the PCV calculates a variable that explains the variation in the degree of barriers to accessing healthcare ; $PCV = \frac{Vnull-VC}{Vnull} \times 100\%$;. where VC is the cluster level variance and Vnull is the variance of the null model.

The fixed effects were employed to calculate the correlation between the probability of barriers to obtaining health care access and independent factors at the individual and community levels. It

was assessed and presented using adjusted odds ratio (AOR) and 95% confidence intervals with a p-value of < 0.05. Because of the nested nature of the model, deviation = -2(log likelihood ratio) was used to compare models, and the model with the lowest deviance was selected as the best-fitted model. ~~The variables used in the models were verified for multi-collinearity by measuring~~By evaluating the variance inflation factors ~~(VIF), with~~, the model assumption for multi-collinearity was confirmed, and the ~~findings falling~~results fell within an acceptable ~~limits~~range of 1~~-10.~~.03-1.98. In addition. The model including random intercepts and slopes (Model III) had the lowest AIC= (AIC=239608.6), indicating the best balance between model fit and parsimony.

Patient and public involvement

Since this study data was obtain from demographic and health survey (DHS) secondary source, no patient and public were involved.

Result

In this study a total of 134,470 reproductive age women were included in Sub-Saharan African. The Mean age of participants were 28.99 (Std. Dev. 9.68) years, and the Variance of 93.86557. More than two thirds (69.8) of women ~~in reproductive age~~ did not use contraception~~; additionally, 69.17% of women in reproductive age and~~, and over 72.22% reproductive women did not use ~~the internet or~~ health insurance ~~respectively. of the women in reproductive age, 51.64% were in the 20–35 age range, and~~, and over half of (54.22~~%~~%) women were ~~from~~living rural areas ~~of~~in sub-Saharan Africa. **(Table 2)**

Table 2: Sociodemographic and economic characteristics of reproductive women who had barriers to accessing health care in sub-Saharan Africa.

Variable	frequency(n)	percentage (%)
**Individual and community level variables**		
**Maternal age**		
15-19	27,890.4	20.7
20-35	69,441.4	51.64
36-49	37,138.6	27.64
**Residency**		
Urban	61,558.03	45.78
Rural	72,912.41	54.22

Formatted: css-1tmeul0, Font: +Body (Calibri), 11 pt
Formatted: css-1g9q2al, Font: +Body (Calibri), 11 pt
Formatted: css-lq4jk2, Font: +Body (Calibri), 11 pt
Formatted: css-1tmeul0, Font: +Body (Calibri), 11 pt
Formatted: css-0, Font: +Body (Calibri), 11 pt
Formatted: css-1tmeul0, Font: +Body (Calibri), 11 pt
Formatted: css-0, Font: +Body (Calibri), 11 pt
Formatted: css-0, Font: +Body (Calibri), 11 pt
Formatted: css-0, Font: +Body (Calibri), 11 pt
Formatted: No Spacing
Formatted: Font color: Black
Formatted: Indent: Left: 0.5"
Formatted: Indent: Left: 0.5"

**Religious status**			
Muslim	48834.95	41.96	**Formatted:** Indent: Left: 0.5"
Christian	46856.90	39.30	
Other	23,524.59	19.73	
**Educational Status**			
No formal education	35,051.70	26.06	**Formatted:** Indent: Left: 0.5"
Primary education	39,651.17	29.48	
Secondary and high	59,767.57	44.44	
**Husband educational**			
No formal education	29,414.44	36.72	**Formatted:** Indent: Left: 0.5"
Primary education	21,245.78	26.52	
secondary and above	29,428.78	36.74	
**Marital Occupational**			
Not working	50,732.90	37.81	**Formatted:** Indent: Left: 0.5"
working	83,417	62.18	
**Husband Occupation**			
Not working	10,390.43	13.0%	**Formatted:** Indent: Left: 0.5"
Working	69,698.11	87.%	
**Marital status**			
single	42,213.40	31.4	**Formatted:** Indent: Left: 0.5"
Married	58,262.00	43.3	
Other	33,995.4	25.3	
**Contraceptive Utilization**			
No	93,855.00	69.8	**Formatted:** Indent: Left: 0.5"
Yes	40,615.5	30.2	
**place of delivery**			
Home	8,279.46	15.5	**Formatted:** Indent: Left: 0.5"
Institution	44,979.28	84.5	
ANC visit			
No visiting	2,012.84	3.77	**Formatted:** Indent: Left: 0.5"
1-4 visiting	24,459.34	45.92	
>4 visiting	26,786.55	50.29	
**Media exposure**			
No	34,628.45	25.75	**Formatted:** Indent: Left: 0.5"
Yes	99,831.57	74.24	
**Intermate utilization**			
No	93,020.34	69.17	**Formatted:** Indent: Left: 0.5"
Yes	41,450.10	30.82	
**Visiting health facility 12 month**			
No	57,137.73	42.49	**Formatted:** Indent: Left: 0.5"

Yes	77,332.71	57.50
**Health insurance**		
No	85,044.69	72.22
Yes	32,709.39	27.77
**Wealth index**		
poor	47,227.18	35.12
middle	25,771.45	19.16
High	61,471.81	45.74
**Region in sub-Saharan Africa**		
East Africa	59,786.29	44.46
West Africa	74,684.15	55.53
**Community poverty level**		
Low	70,776.71	52.63
High	63,693.73	47.47
**Community media exposure**		
Low	61,642.30	45.84
High	72,828.14	54.15
**Community distance health facility**		
Low	65,962	49
High	68,508	51
**Community literacy level**		
Low	57,615.01	42.84
High	76,855.43	57.15
**~~community~~Community ANC visit**		
Low	77,912.32	57.96
High	56,491.92	42.03

In this study, the magnitude of barriers to accessing health care among reproductive age women was found to be 55.84 (95% CI: (55.57, 56.10) owing to one or more of the four factors, the most commonly cited being the difficulty in getting money and the distance from medical facilities **(Figure 1)**

In this study, getting money for healthcare and distance to healthcare facility were the major contributing factor in accessing health care **(Figure 2)**

Random effect (Measures of variation) and model fitness

~~A null model was used to ascertain whether~~To determine if the data supported the ~~decision~~choice to ~~assess~~evaluate randomness ~~of~~ at the community level~~. Findings from the null model revealed~~

Formatted: Indent: Left: 0.5"
Formatted: Indent: Left: 0.5"
Formatted: Indent: Left: 0.5"
Formatted: Indent: Left: 0.5"
Formatted: Indent: Left: 0.5"
Formatted: Font: Not Bold
Formatted: Indent: Left: 0.5"
Formatted: Indent: Left: 0.5"
Formatted: Indent: Left: 0.5"
Formatted: Font: 14 pt, Font color: Auto
Formatted: Heading 3, Left
Formatted: Font: 14 pt
Formatted: Normal

that there were significant differences in barrier to accessing health care between communities, with , a null model was employed. With a variance of -.3784967 and a p value of 0.000 and the variance between cluster was 10.31%. In the null model, the odds of , the null model's results showed that there were notable variations in the barriers to accessing health care differed between higher and lower riskhealthcare among clusters by a factor of 1.79 times. The intraclass correlation value for Model I indicated that 5.41% of the variation in barriers to accessing health care accounts for the disparities between communities. Then with the null model, we used . A significant amount of the variance in the outcome between clusters was jointly explained by both individual- and community-level variables to generate Model II. Cluster variations were the basis for 7.98% of the differences in barriers to accessing health care, according to the ICC value from Model II. In the final, as evidenced by the intra-cluster correlation (ICC), which dropped from 10.31% in the null model to 3.96% in the fully adjusted model (model III), which attributed approximately). According to the proportionate change in variance (PCV), Model III explains the greatest percentage (64.09% of the variation in the likelihood of barriers to accessing health care to both individual and community%) of the group-level variables,variation in comparison to the null model, group comparison showed that, Model I (50.19%) and Model II (24.56%). This suggests that the model fits better as more predictors are included. Akaike information criteria **(AIC)** Among the models evaluated, Model III demonstrated the likelihood of barriers to health care accessing varied by best fit with the lowest AIC (239,608.6), indicating a substantial improvement over the null model (AIC = 1,128,437) and Model II (AIC = 1.41 times across low and high barriers to accessing health care in clusters ,127,704). Model I also showed good fit (AIC = 239,764.8), though slightly less optimal than Model III **(Table 3).**

Table 3: Model comparison and Fitness random effect analysis for barriers of accessing health care of among reproductive age women in sub-Saharan Africa**.**

**Parameter**	**Null model**	**model I**	**model II**	**model III**
Variance	.3784967	.1885269	0.28553	— 0.1358817
ICC	10.31%	5.41%	7.98%	3.96%
MOR	1.79	1.51	1.66	1.41
PCV	Reference	50.19%	24.56%	64.09%
**Model fines**				

Formatted: Line spacing: Multiple 1.15 li
Formatted: Line spacing: Multiple 1.15 li
Formatted Table
Formatted: Line spacing: Multiple 1.15 li
Formatted: Line spacing: Multiple 1.15 li
Formatted: Line spacing: Multiple 1.15 li
Formatted Table

LLR	-90175.365	-21798.27	-86841.704	-21433.284
Deviance	1803596.73	43596.54	173683.408	42866.568
AIC	1128437	239764.8	1127704	239608.6

ICC: intra-cluster correlation**, LLR:** loglikelihood ratio**, MOR:** median odds ratio**, PCV:** proportional change in variance. **AIC:** Akaike information criteria.

Formatted: Line spacing: Multiple 1.15 li

Formatted Table

Determinants of individual and community level factors with barrios of accessing health care among reproductive age women in sub-Saharan Africa.

In the final fitted model of multivariable multilevel logistic regression, Husband educational status, media exposure, internet utilization, place of residency, ANC visit status, place of delivery, health insurance utilization status, wealth index status, visiting health facility within 12 months and, and country category (western, eastern Africa) were significantly associated with barriers of accessing health care among reproductive age women in sub-Saharan Africa.

The odds of barrier of accessing health care were 1.30 times higher among reproductive women living in rural residency compared to women living in urban area **(AOR=** 1.30, 95 CI: (1.22, 1.39). and the odds of barrier of accessing health care were 1.23 and 1.21 times more likely to among reproductive age women whose husband had no education and primary education compared to women whose husband had secondary and above educational level respectively **AOR**=1.23, 95 CI: (1.3015, 1.15)30,) and (**AOR**=1.21, 95% CI: (1.31, 1.41). In addition, odds of barrier of accessing health care were 1.33 times more likely to occur women delivered at home compared to women who had institutional delivery. **(AOR**=1.33, 95% CI: (1.24, 1.43) and the odds of barrier of accessing health care were 1.31- and 1.13-times higher women who had no history of antenatal visit and women who had < 4 ANC visit compared to women who had more than four ANC visit Respectively. (**AOR**= 1.31, 95% CI: (1.10,1.55) and (**AOR=** 1.13, 95% CI: (1.07,1.19) and the odds of the barrier of accessing health care among reproductive age woman who had no media exposure and intranet access were 1.17 and 1.68 times more likely to compared with a woman who had media exposure and internet access respectively **(AOR**=1.17, 95% CI: (1.11, 1.24) and (**AOR**= 1.68, 95% CI: (1.57, 1.79). and the barrier of accessing health care among reproductive age woman who were not visiting health facility within 12 months 9 % times less likely compared to women who had visiting health care facility within 12 months of study period **(AOR=**0.91, 95% (0.87, 0.97). The odds of perceived barrier of accessing health care among reproductive age

woman who had no health insurance utilization were 1.18 times more likely to compared with a woman who had used health insurance (**AOR=**1.18 95% CI: (1.11, 1.26) and The odds of perceived barrier of accessing health care service among reproductive age woman who were classified under poor and middle wealth status were 2.29 and 1.57 times more likely accessing health care challenge compared to reproductive women who were classified under rich wealth status category**(AOR**=2.29 95% CI: (2.14, 2.45) and (**AOR**=1.57.95% CI: (1.46, 1.68).

The odds of perceived barrier of accessing health care service among reproductive age woman who in west Africa region were 2.38 times more likely barriers accessing health care compared to east Africa region category (**AOR**=2.38, 95% CI: (2.21, 2.55) **(Table 4)**

Table 4: Multivariable multilevel logistic regression analysis of individual-level and community level variable associated with accessing health care among reproductive women in sub Saharan Africa.

Individual and community level factors	Model I AOR (95% CI)	Model II AOR (95% CI)	Model III AOR (95% CI)
**Maternal age**			
15-19	0.96(0.86, 1.07)		0.96 (0.86,1.08)
20-35	1.06(1.01,1.13)		1.04 (0.99, 1.11)
36-45	1		1
**Residency**			
Urban		1	1
Rural		2.29 (2.24, 2.35)	**1.30 (1.22, 1.39)**
**Maternal education**			
No education	1.10 (1.02, 1.18)		1.04 (0.97, 1.11)
Primary education	0.93 (0.87, 0.99)		1.06 (0.99, 1.14)
Secondary and above	1		1
**Husband education**			
No education	1.26 (1.19,1.35)		**1.23 (1.~~30~~15, 1.~~15~~30)**
Primary education	1.04(0.97,1.12)		1.23 (0.67, 1.22)
secondary education	1		1
**Marital occupation**	1		1
working	0.99 (0.94~~—~~, 1.04)		**1.~~01~~02 (1.~~02~~01, 1.11)**
not working			
**Husband occupation**			
working	1		1
not working	0.90 (0.83, 0.97)		1.00 (0.92, 1.08)

Formatted: Normal

Formatted: Font color: Auto

Formatted: Indent: Left: 0.5"

Formatted: Indent: Left: 0.5"

Formatted: Indent: Left: 0.5"

Formatted: Indent: Left: 0.5"

Formatted: Indent: Left: 0.5"

Formatted: Indent: Left: 0.5"

**Religion**			
Muslim	1		1
Christian	1.62 (1.53, 1.71)		0.46 (0.57, 1.94)
Other	1.22 (1.13, 1.31)		1.36 (0.75, 1.46)
**Contraceptive utilization**			
No	1.11 (1.05,1.17		1.03 (0.98, 1.08)
Yes	1		1
**Place of delivery**			
home	1.30 (1.21, 1.40)		**1.33 (1.24, 1.43)**
Institution	1		**1**
**ANC visit**			
No	1.01 (0.85, 1.19)		**1.31 (1.10, 1.55)**
1-4 visit	0.94 (0.89, 0.99)		1.13 (0.37, 1.19)
> 4 visits	1		1
**Media exposure**			
No	1.14 (1.08, 1.21)		**1.17 (1.11, 1.24)**
Yes	1		1
**Internet utilization**			
No	1.69 (1.58, 1.80)		1.68 (0.67, 1.79)
Yes	1		1
**Visiting health facility 12 month**			
No	0.92 (0.87, 0.98)		**0.91 (0.87, 0.97)**
Yes	1		1
**Health insurance**			
No	1.26 (1.19, 1.34)		**1.18 (1.11, 1.26)**
Yes	1		1
**Wealth index**			
poor	2.80 (2.63, 2.97)		**2.29 (2.14, 2.45)**
middle	1.79 (1.68, 1.92)		**1.57 (1.46, 1.68)**
Rich	1		1
**Region SSA**			
East Africa		0.50 (0.49, 0.52)	**0.42 (0.39, 0.45)**
West Africa		1	1
**community ANC visit**			
Low		1.00 (0.93, 1.09)	0.93 (0.86, 1.00)
High		1	1
**community wealth index**			
Low		0.75 (0.70, 0.81)	**1.12 (1.02, 1.22)**
High		1	1
**Community media exposure**			
Low		1.01 (0.92, 1.10)	0.94 (0.86, 1.03)
High		1	1
**Community literacy level**			
Low		1.10(1.00, 1.20)	**1.13 (1.03, 1.24)**
High		1	**1**

Table 5: Key Individual and Community-Level Determinants of Barriers to Healthcare Access Among Women of Reproductive Age in Sub-Saharan Africa.

**Individual and community level factors**	**Model I AOR (95% CI)**	**Model II AOR (95% CI)**	**Model III AOR (95% CI)**
**Residency**			
Urban		1	1
Rural		2.29 (2.24, 2.35)	**1.30 (1.22, 1.39)**
**Husband education**			
No education	1.26 (1.19,1.35)		**1.23 (1.15, 1.30)**
Primary education	1.04(0.97,1.12)		1.23 (0.67, 1.22)
secondary education	1		1
**Marital occupation**			
working	1		1
not working	0.99 (0.94 1.04)		**1.02 (1.01, 1.11)**
**Place of delivery**			
home	1.30 (1.21, 1.40)		**1.33 (1.24,1.43)**
Institution	1		**1**
**ANC visit**			
No	1.01 (0.85, 1.19)		**1.31(1.10,1.55)**
1-4 visit	0.94 (0.89, 0.99)		1.13(0.37,1.19)
> 4 visits	1		1
**Media exposure**			
No	1.14 (1.08, 1.21)		**1.17 (1.11, 1.24)**
Yes	1		1
**Visiting health facility 12 month**			
No	0.92 (0.87, 0.98)		**0.91(0.87, 0.97)**
Yes	1		1
**Health insurance**			
No	1.26 (1.19, 1.34)		**1.18 (1.11,1.26)**
Yes	1		1
**Wealth index**			
poor	2.80 (2.63, 2.97)		**2.29 (2.14, 2.45)**
middle	1.79 (1.68, 1.92)		**1.57 (1.46, 1.68)**
Rich	1		1
**Region SSA**			
East Africa		0.50 (0.49, 0.52)	**0.42 (0.39, 0.45)**
West Africa		1	1

Formatted: Line spacing: Multiple 1.15 li
Formatted: Line spacing: Multiple 1.15 li
Formatted: Indent: Left: 0.5", Line spacing: Multiple 1.15 li
Formatted: Line spacing: Multiple 1.15 li
Formatted: Indent: Left: 0.5", Line spacing: Multiple 1.15 li
Formatted: Line spacing: Multiple 1.15 li
Formatted: Line spacing: Multiple 1.15 li
Formatted: Indent: Left: 0.5", Line spacing: Multiple 1.15 li
Formatted: Line spacing: Multiple 1.15 li
Formatted: Line spacing: Multiple 1.15 li
Formatted: Indent: Left: 0.5", Line spacing: Multiple 1.15 li
Formatted: Line spacing: Multiple 1.15 li

~~**community ANC visit**~~		
~~Low~~	~~1.00 (0.93, 1.09)~~	~~0.93(0.86,1.00)~~
~~High~~	~~1~~	~~1~~
**community wealth index**		
Low	0.75 (0.70, 0.81)	**1.12 (1.02,1.22)**
High	1	1
~~**Community media exposure**~~		
~~Low~~	~~1.01 (0.92, 1.10)~~	~~0.94(0.86, 1.03)~~
~~High~~	~~1~~	~~1~~
**Community literacy level**		
Low	1.10(1.00, 1.20)	**1.13(1.03,1.24)**
High	1	**1**

Discussion

Formatted: Line spacing: Multiple 1.15 li

Formatted: Indent: Left: 0.5", Line spacing: Multiple 1.15 li

Formatted: Line spacing: Multiple 1.15 li

Formatted: Line spacing: Multiple 1.15 li

Formatted: Indent: Left: 0.5", Line spacing: Multiple 1.15 li

Formatted: Line spacing: Multiple 1.15 li

Formatted: Font: 18 pt

Formatted: No Spacing

Formatted: Font: 18 pt

~~The barrier of ~~Barriers to accessing health care ~~were significantly contribute in~~have contributed to maternal and child mortality and morbidity in developing countries such as those in Sub-Saharan Africa. ~~The aim of this~~This study ~~was~~aimed to ~~determine~~assess the magnitude and ~~its ~~determinants of barriers ~~of~~to health care access among women of reproductive age ~~women ~~in ~~sub~~Sub-Saharan ~~African~~Africa.

In this study, the magnitude of barriers to accessing health care among reproductive age women was found to be 55.84 (95% CI: (55.57, 56.10) owing to one or more of the four factors, the most commonly cited being the difficulty in getting money and the distance from medical facilities.

This finding is lower than the previous study conducted in South Africa 65%~~(17, 18),~~[18, 19], and Tanzania 65% ~~(15), Ethiopia 71.8%,70.2, 69.9%~~[15], Ethiopia 71.8%,70.2, 69.9%~~(~~[1, ~~19, ~~20~~)~~, 21], (SSA) 60.4%, 61.5% ~~(21).~~[17]. this variation could be due to the fact that socio-economic and variability in health infrastructure and health system policy, women's attitudes towards health care seeking behavior and cultural differences across countries in sub-Saharan Africa, moreover, this discrepancy might be due to study period, this study includes, the most recent DHS data set from 2019-2023. On the other hand, the finding of this study is similar to but slightly higher than in previous study in Gambia 45.5%~~(22),~~[22], Ghana (51%)~~(23).~~[23]. this variation could be due to the fact that socio-economic and variability in health infrastructure and health system policy, women's attitudes towards health care seeking behavior and cultural differences across countries in sub-Saharan Africa.

The odds of barrier of accessing health care were 1.30 times higher among reproductive women living in rural residency compared to women living in urban area. This finding is supported by previous studies conducted in Ethiopia ~~(~~[1, ~~19)~~20], guinea~~(24), Gambia, Tanzania and Ghana ~~[24], Gambia, Tanzania and Ghana ~~(~~[15, 22, 25~~)~~] respectively. Sub-Saharan~~(23),~~[23], East Africa~~(26).~~[26]. This association may be, due to the fact that rural areas have low health infrastructure i.e. inaccessibility of health facilities, less privileged and the influences of socio-cultural practices that need women to seek permission from their husband to visit the facility or women's decisions for seeking healthcare services is under their husband/partners control. Moreover, male involvement and support for women health care access are limited~~(~~[1, 14, 23, 26~~)~~]. This implies that the government should expand infrastructure services in rural area and encourage male involvement and support for improving health care access for reproductive age women~~(14).~~[14].

Formatted: No Spacing

The odds of perceived barrier of accessing health care were 1.23 and 1.21 times more likely to among reproductive age women whose husband had no education and primary education compared to women whose husband had secondary and above educational level respectively. This find was consistent with previous study conducted in east Africa, Ethiopia, Tanzania, Benin, Bangladesh respectively ~~(~~[1, 15, ~~19~~20, 26, 27~~)~~]. The possible explanation could be in better access to the healthcare among women with educated husbands might be due to better participation and involvement of husbands in their families' health~~(23).~~[23]. Women whose partners are educated are also likely to be informed regarding their fundamental human rights and may have higher health literacy. As a result, they are more likely to deal with any form of barrier to healthcare, compared to their counterparts who are less educated and may have lower health literacy ~~(~~[14, 15, ~~19~~20, 28~~)~~].

The odds of barrier to accessing health care were 1.33 times more likely to occur women delivered at home compared to women who had institutional delivery. This evidence was supported by study done in Senegal~~(29).~~[29]. The plausible justification might be women who delivered at home may not get health information/counselling from healthcare providers that empower them to use other maternal healthcare services~~.~~ and women who deliver at home may face varous barriers to accessing health care such as financial constraint, lack of transportation, limited autonomy in healthcare decision and sociocultural norms that discourage institutional delivery. Moreover, importance of enhancing health system outreach, strengthen community-based education, and addressing sociocultural and socioeconomic disparities that affect health seeking behavior. On the other hand, the odds of ~~perceived~~ barrier of accessing health care among ~~reproductive age~~ woman who had no media exposure ~~and intranet access~~ were 1.17 ~~and~~ times more likely to compared with a woman who had ~~intermate access and~~ media exposure. This evidence was supported by study done in Ethiopia~~(30),~~[30], Nepal~~(31).~~[31]. This could be the power of mass media in disseminating information concerning maternal health that may enhance women's knowledge and attitude towards health ~~service~~care access and utilization~~(31).~~[31]. Barrier of accessing health care among ~~reproductive age~~ woman who were not visiting health facility within 12 months were 9 % times less likely compared to ~~–~~women who had visiting health care facility within 12 months of study period. The odds of ~~perceived~~ barrier ~~of~~to accessing health care among ~~reproductive age~~ woman who had no health insurance utilization were 1.18 times more likely to compared with a woman who had used health insurance. This finding was supported with study done in Ethiopia~~(1),~~[1],

sub-Saharan Africa~~(23),~~[23], Tanzania~~(15),~~[15], Ghana~~(25).~~[25]. The possible reason ~~was~~might be due to the fact that, individuals who were not had health insurance faced for unexpected catastrophic expenditure and difficulties in obtaining money for consultation of the doctor~~(1).~~ [1]. The odds of ~~perceived~~ barrier ~~of~~to accessing ~~health care service~~healthcare among ~~reproductive age~~, woman who were classified under poor and middle wealth status were 2.29 and 1.57 times more likely accessing health care difficulty compared to ~~reproductive~~ women who were classified under rich wealth index category**.** This evidenced was supported with study done in Ethiopia, ~~(~~[1, 30, 32, 33~~)~~] ~~Tanzania(15) Uganda(34), sub Saharan Africa(14). This finding may be explained by the fact that being in the poor and middle class requires individuals to spend their income on basic needs,~~

~~therefore be less likely to be affordable to health care access(35, 36)~~

~~The odds of perceived barrier of accessing health care among reproductive age woman who live west Africa region were 2.38 times more likely barriers accessing health care compared to east Africa region category. Women from all regions of in sub-saran Africa face higher problems of accessing healthcare as compared with northern and southern. In this study being in western Africa region, reproductive age women face difficulties of accessing health care compared to east Africa region. The possible reason might be differences in healthcare accessibility, infrastructure and socioeconomic level in the regions(22)~~

Tanzania[15] Uganda[34], sub Saharan Africa[14]. This finding might be explained by the fact that high out-of-pocket expenses and a lack of accessibility to healthcare facilities pose significant obstacles for middle-class and impoverished women in sub-Saharan Africa. Many people in the region cannot afford healthcare services because over 40% of health costs are paid for directly by individuals[35-38]

The odds of barrier of accessing health care among woman who live west Africa region were 2.38 times more likely barriers to accessing health care compared to east Africa region. According a recent joint report from the World Health Organization and the United Nations states that high out-of-pocket costs, a lack of adequate healthcare infrastructure, and other sociocultural barriers make it more difficult for women of reproductive age in West African nations such as Nigeria, Mali, and Sierra Leone to access maternal healthcare. On the other hand, East African countries like Rwanda, Kenya, and Ethiopia have strengthened their community health initiatives and gender-inclusive policies, which have improved women's autonomy in accessing healthcare services[39].

Formatted: Font color: Black

Furthermore, our current findings are supported by recent Demographic and Health Survey (DHS) program data (2022–2023), which indicate that women in West African countries are 2-3 times more likely to report delays in accessing healthcare due to cost, shortages of healthcare personnel, and long distances to health facilities. In contrast, East African countries, particularly Uganda and Tanzania, have strengthened their health insurance systems and rural outreach initiatives. Additionally, the World Bank's 2023 Health Service Coverage Index reveals that women in West Africa have significantly lower access to family planning, skilled birth attendance, and other essential reproductive health services[22, 40, 41]

Strength and limitation

Formatted: Font: 18 pt

This strength of study nationally representative data to evaluate contextual and individual characteristics related to barriers to healthcare access among reproductive age women in the SSA. Thus, findings can therefore, generalized to all women in their reproductive age in SSA, in order to account for the clusters within the sample, the study also employed advanced statistical models. This study has its own limitation, that is, the findings do not establish a cause and effect relationship due to the cross-sectional nature of the data/surveys and health-worker related factors could not be accounted for in this study.

Conclusion and Recommendation

Formatted: Font: 18 pt

This study found that 55.55% of reproductive age women had barriers to accessing healthcare in SSA. Husband educational status, media exposure, internet utilization, place of residency, ANC visit status, place of delivery, health insurance utilization status, wealth index status, visiting health facility within 12 months, community literacy level and community wealth index and country category (western, eastern Africa) were significantly associated with barriers of accessing health care. Therefore, the Government and ministry of health in Sub-Saharan Africa countries should taking in to consideration those factors while designing policies and strategies targeting to health care access and tackling the preventable barriers to accessing health care in sub-Saharan Africa.

Declaration

Formatted: Font: 18 pt

Ethical approval and consent to participate

Formatted: Font: 16 pt

This study was based on analysis of existing survey datasets in the public domain (SSA) that are freely available online with all the identifier information anonymized, no ethical approval was required

Consent for publication

Not applicable

Data and material availability

Demographic and Health Survey were used, and it is publicly available online at (https://www.dhsprogram.com).

Competing interest

Authors Declared that, there is no competing interest.

Funding

Not applicable

Author's contributions

GAZ: involved in designing, data extraction, data analysis, interpretation, report and manuscript writing.

BT: involved in data analysis, interpretation, and, and manuscript writing.

BT: involved in analysis,

TTT: involved in designing the study, interpretation, report and

BSW: involved in analysis, and result interpretation

AFZ: involved in designing and interpretation study

EGM: analysis and manuscript writing

MW: Result interpretation

AA: manuscript writing: involved in data extraction

MSA: involved manuscript writing

Acknowledgements

We would like to say the DHS programs for allowing us to use the relevant DHS dataset in this study.

Formatted: Justified

Formatted: Font: 14 pt

Formatted: Font: 14 pt

Formatted: Font: 14 pt

Formatted: Font: 14 pt

Formatted: Font: 14 pt

Formatted: Font: 18 pt

Reference

1. Tamirat~~ KS;~~, K.S., Z.T. Tessema~~ ZT;~~, and F.B.J.B.H.S.R. Kebede~~ FBJBHSR.~~, Factors associated with the perceived barriers of health care access among reproductive-age women in Ethiopia: a secondary data analysis of 2016 Ethiopian demographic and health survey. 2020~~;~~. **20**: p. 1-8.
2. Erasmus~~ MO.~~, M.O., The barriers to access for maternal health care amongst pregnant adolescents in the Mitchells plain sub-district. 2017.
3. Washington~~ DL, Bean-Mayberry B, Riopelle~~, D~~, Yano EMJJogim.~~.L., et al., Access to care for women veterans: delayed healthcare and unmet need. 2011~~;~~. **26**: p. 655-~~61~~661.
4. Regan~~ LJBMA.~~, L.J.B.M.A., Addressing unmet needs in global women's health. 2018.
5. Izugbara, C~~,~~., E. Covan, and E~~,~~. Fugate-Whitlock~~ E.~~, Women's health in Africa: Issues, challenges and opportunities. 2017: Routledge~~; 2017~~.
6. Organization~~ WH.~~, W.H., Primary health care on the road to universal health coverage: 2019 global monitoring report: Executive summary. 2019, World Health Organization~~;~~ ~~2019. Report No.: 9240029044~~.
7. Badiuzzaman, M~~,~~., S.M. Murshed~~ SM,~~, and M.J.T.J.o.D.S. Rieger~~ MJTJoDS.~~, Improving maternal health care in a post conflict setting: evidence from Chittagong Hill tracts of Bangladesh. 2020~~;~~. **56**(2): p. 384-400.
8. Peters~~ DH, Garg A, Bloom G, Walker DG, Brieger WR, Hafizur Rahman MJAotnYAoS.~~, D.H., et al., Poverty and access to health care in developing countries. 2008~~;~~. **1136**(1): p. 161-~~71~~171.
9. Alkire~~ BC, Peters AW, Shrime MG, Meara JGJHA.~~, B.C., et al., The economic consequences of mortality amenable to high-quality health care in low-and middle-income countries. 2018~~;~~. **37**(6): p. 988-~~96~~996.
10. Nyakang'o~~ SB,~~, S.B. and A.J.M. Booth~~ AJM.~~, Women's perceived barriers to giving birth in health facilities in rural Kenya: A qualitative evidence synthesis. 2018~~;~~. **67**: p. 1-11.
11. Colglazier~~ WJS.~~, W.J.S., Sustainable development agenda: 2030. 2015~~;~~. **349**(6252): p. 1048-~~50~~1050.

Formatted: Font: 18 pt

Formatted: Font: 18 pt, Bold

Formatted: Indent: Left: 0", Hanging: 0.5", Line spacing: 1.5 lines

Formatted: Font: Bold

Formatted: Font: Bold

Formatted: Font: Bold

Formatted: Font: Bold

Formatted: Font: Bold

Formatted: Font: Bold

Formatted: Font: Bold

12.~~-~~ Cahill~~ CM, Alhasson B, Blumberg N, Melvin A, Knight P, Gloff ~~, C.M~~;~~., et al~~.~~., Preoperative anemia management program reduces blood transfusion in elective cardiac surgical patients, improving outcomes and decreasing hospital length of stay. 2021~~;~~. **61**(9): p. 2629-~~36~~2636.

13.~~-~~ Peeri~~ NC, Shrestha~~, N~~, Rahman MS, Zaki R, Tan Z, Bibi S~~,.C., et al~~.~~., The SARS, MERS and novel coronavirus (COVID-19) epidemics, the newest and biggest global health threats: what lessons have we learned? 2020~~;~~. **49**(3): p. 717-~~26~~726.

14.~~-~~ Tessema~~ ZT, Worku MG, Tesema GA, Alamneh TS, Teshale AB, Yeshaw Y~~, Z.T., et al~~.~~., Determinants of accessing healthcare in Sub-Saharan Africa: a mixed-effect analysis of recent Demographic and Health Surveys from 36 countries. 2022~~;~~. **12**(1): p. e054397.

15.~~-~~ Bintabara, D~~,~~., K. Nakamura, and K~~,~~.J.B.o. Seino~~ KJBo.~~, Improving access to healthcare for women in Tanzania by addressing socioeconomic determinants and health insurance: a population-based cross-sectional survey. 2018~~;~~. **8**(9): p. e023013.

~~16. Kea AZ, Tulloch O, Datiko DG, Theobald S, Kok MCJBp, childbirth.~~16. Kea, A.Z., et al., Exploring barriers to the use of formal maternal health services and priority areas for action in Sidama zone, southern Ethiopia. 2018~~;~~. **18**: p. 1-12.

17.~~-~~ Zegeye, B., et al., Breaking barriers to healthcare access: a multilevel analysis of individual-and community-level factors affecting women's access to healthcare services in Benin. 2021. **18**(2): p. 750.

18. Silal~~ SP, Penn-Kekana L, Harris B, Birch~~, S~~, McIntyre DJBhsr.~~.P., et al., Exploring inequalities in access to and use of maternal health services in South Africa. 2012~~;~~. **12**: p. 1-12.

~~18.~~ 19. Tsawe, M~~,~~. and A.S.J.B.r.n. Susuman~~ ASJBrn.~~, Determinants of access to and use of maternal health care services in the Eastern Cape, South Africa: a quantitative and qualitative investigation. 2014~~;~~. **7**: p. 1-10.

~~19.~~ 20. Zegeye, B~~, Adjei NK, Ahinkorah BO, Ameyaw EK, Budu E, Seidu A-A~~,., et al~~.~~., Barriers and facilitators to accessing health care services among married women in Ethiopia: a multi-level analysis of the Ethiopia demographic and health survey. 2021.

~~20.~~ 21. Demsash~~ AW~~, A.W., B. Chakilu~~ B~~, and A. Mazengia~~ A.~~, Knowledge Sharing Practice and Its Associated Factors Among Healthcare Providers at University of Gondar Comprehensive Specialized Hospital, North West Ethiopian: Cross-sectional Study. 2021.

Formatted: Font: Bold

Formatted: Font: Bold

Formatted: Font: Bold

Formatted: Font: Bold

Formatted: Font: Bold

Formatted: Indent: Left: 0", Hanging: 0.5", Line spacing: 1.5 lines

Formatted: Font: Bold

Formatted: Font: Bold

~~21. Zegeye B, El-Khatib Z, Ameyaw EK, Seidu A-A, Ahinkorah BO, Keetile M, et al. Breaking barriers to healthcare access: a multilevel analysis of individual- and community-level factors affecting women's access to healthcare services in Benin. 2021;18(2):750.~~

22. Tsegaw, M~~;~~., B. Mulat~~-~~, and K.J.B~~;~~.o. Shitu~~ KJBo.,~~ Problems with accessing healthcare and associated factors among reproductive-aged women in the Gambia using Gambia demographic and Health Survey 2019/2020: a cross-sectional study. 2023~~;~~. **13**(8): p. e073491.

23. Seidu, A~~ AJPO.~~.-A.J.P.O., Mixed effects analysis of factors associated with barriers to accessing healthcare among women in sub-Saharan Africa: Insights from demographic and health surveys. 2020~~;~~. **15**(11): p. e0241409.

24. Seidu, A~~-~~.-A~~, Agbaglo E, Dadzie LK, Ahinkorah BO, Ameyaw EK, Tetteh JKJIH.~~., et al., Individual and contextual factors associated with barriers to accessing healthcare among women in Papua New Guinea: insights from a nationwide demographic and health survey. 2021~~;~~. **13**(6): p. 573-~~85~~585.

25. Seidu, A~~-~~.-A~~, Darteh EKM, Agbaglo E, Dadzie LK, Ahinkorah BO, Ameyaw EK,~~., et al~~.~~., Barriers to accessing healthcare among women in Ghana: a multilevel modelling. 2020~~;~~. **20**: p. 1-12.

26. Minyihun, A~~,~~., Z.T.J.R.M. Tessema~~ ZTJRM,~~, and H. Policy~~ H.,~~ Determinants of access to health care among women in East African countries: a multilevel analysis of recent demographic and health surveys from 2008 to 2017. 2020: p. 1803-~~13~~1813.

27. Sen~~ KK, Nilima S, Zahura F-T, Bari WJBPH.~~, K.K., et al., Do education and living standard matter in breaking barriers to healthcare access among women in Bangladesh? 2023~~;~~. **23**(1): p. 1431.

28. Dimbuene~~ ZT, Amo-Adjei J, Amugsi D, Mumah J, Izugbara CO, Beguy DJJobs.~~, Z.T., et al., Women's education and utilization of maternal health services in Africa: a multi-country and socioeconomic status analysis. 2018~~;~~. **50**(6): p. 725-~~48~~748.

~~29. Zegeye B, Ahinkorah BO, Idriss-Wheelr D, Oladimeji O, Olorunsaiye CZ, Yaya SJAoPH.~~29. Zegeye, B., et al., Predictors of institutional delivery service utilization among women of reproductive age in Senegal: a population-based study. 2021~~;~~. **79**: p. 1-11.

30. Demsash~~ AW,~~, A.W., A.D.J.B.H. Walle~~ ADJBH,~~, and C. Informatics~~ C.,~~ Women's health service access and associated factors in Ethiopia: application of geographical information system and multilevel analysis. 2023~~;~~. **30**(1).

Formatted: Indent: Left: 0", Hanging: 0.5", Line spacing: 1.5 lines

Formatted: Font: Bold

Formatted: Font: Bold

Formatted: Font: Bold

Formatted: Font: Bold

Formatted: Font: Bold

Formatted: Font: Bold

Formatted: Font: Bold

Formatted: Font: Bold

31.- Tamang TM, editor, T.M. Factors associated with completion of continuum of Care for Maternal Health in Nepal. in IUSSP XXVIII International Population Conference, Cape Town, South Africa;. 2017.

32.- Fentie EA, Asmamaw DB, Negash WD, Belachew TB, Amare Baykeda T, Addis B,, E.A., et al.., Spatial distribution and determinants of barriers of health care access among female youths in Ethiopia, a mixed effect and spatial analysis. 2023;. **13**(1): p. 21517.

33.- Alamneh TS, Teshale AB, Yeshaw Y, Alem AZ, Ayalew HG, Liyew AM,, T.S., et al.., Barriers for health care access affects maternal continuum of care utilization in Ethiopia; spatial analysis and generalized estimating equation. 2022;. **17**(4): p. e0266490.

34.- Kalule-Sabiti, I;., A.Y. Amoateng AY,, and M.J.A.P.S. Ngake MJAPS., The effect of socio-demographic factors on the utilization of maternal health care services in Uganda. 2014: p. 515-25525.

35.- Shiferaw, S, Spigt M, Godefrooij M, Melkamu Y, Tekie MJBp, childbirth.., et al., Why do women prefer home births in Ethiopia? 2013;. **13**: p. 1-10.

36.- Yaya, S;., G. Bishwajit G,, and V.J.B.g.h. Shah VJBgh., Wealth, education and urban–rural inequality and maternal healthcare service usage in Malawi. 2016;. **1**(2): p. e000085.

37. Voto, T.P., B.E. Voto, and N.J.E. Ngepah, The Impact of Out-of-Pocket Health Expenditure and Public Health Expenditure on Poverty in Sub-Saharan Africa. 2025. **13**(5): p. 134.

38. Alamneh, T.S., et al., Socioeconomic inequality in barriers for accessing health care among married reproductive aged women in sub-Saharan African countries: a decomposition analysis. 2022. **22**(1): p. 130.

39. AKINWALE, A.A.J.I.J.o.I.R. and A. Studies, Gender equity and social progress: empowering women and girls to drive sustainable development in Sub–Saharan Africa. 2023.

40. Arhin, K., E.F. Oteng-Abayie, and J.J.H.e.r. Novignon, Assessing the efficiency of health systems in achieving the universal health coverage goal: evidence from Sub-Saharan Africa. 2023. **13**(1): p. 25.

41. Muhajarine, N., et al., Inequalities and factors associated with maternal healthcare services utilisation in Mozambique: evidence from the Demographic and Health Survey 2022−2023. 2025. **10**(5).

Formatted: Font: Bold

Formatted: Font: Bold

Formatted: Font: Bold

Formatted: Font: Bold

636

Title: Barriers to accessing health care among women's in Sub-Saharan Africa: Multilevel Analysis of Recent Demographic and Health Survey from 2019-2023.

Gebreeyesus Abera Zeleke[1]*, Enyew Getaneh Mekonen[1], Bewuketu Terefe[2], Mulugeta Wassie[3], Agazhe Aemro[3], Alebachew Ferede Zegeye[3], Mohammed Seid Ali[4], Berhan Tekeba[4], Tadesse Tarik Tamir[4], Belayneh Shetie Workneh[5]

[1] Department of Surgical Nursing, School of Nursing, College of Medicine and Health Sciences, University of Gondar, Gondar, Ethiopia

[2] Department of Community Health Nursing, School of Nursing, College of Medicine and Health Sciences, University of Gondar, Gondar, Ethiopia.

[3] Department of Medical Nursing, School of Nursing, College of Medicine and Health Sciences, University of Gondar, Gondar, Ethiopia;

[4] Department of Pediatrics and Child Health Nursing, School of Nursing, College of Medicine and Health Sciences, University of Gondar, Gondar, Ethiopia.

5 Department of Emergency and Critical Care Nursing, School of Nursing, College of Medicine and Health Sciences, University of Gondar, Gondar, Ethiopia

Correspondence Author*

Gebreeyesus Abera Zeleke

Department of Surgical Nursing, School of Nursing, College of Medicine and Health Science, University of Gondar, Gondar, Ethiopia.

Email address: gebreeyesusabe143@gmail.com

Co-authors:

Enyew Getaneh Mekonen **Email Address:** enyewgetaneh12@gmail.com

Bewuketu Terefe: **Email address**: woldeabwomariam@gmail.com

Mulugeta Wassie: **Email address:** mulugeta2113@gmail.com

Agazhe Aemro: **Email address:** agazhe1049@gmail.com

Alebachew Ferede Zegeye: **Email address:** alexferede24@gmail.com

Mohammed Seid Ali: **Email address:** muheseid2592@gmail.com

Berhan Tekeba: **Email address:** berishboss7@gmail.com

Tadesse Tarik Tamir: **Email address:** tadestar140@gmail.com

Belayneh Shetie Workneh: **Email address:** belay2319@gmail.com

Abstract

Background:

Barriers to accessing healthcare have significantly contributed to the high rates of maternal and child mortality and morbidity in developing regions, particularly in Sub-Saharan Africa. Access to healthcare is shaped by multiple factors, including financial constraints, geographic location, the availability of services, and the quality of care provided. This study aimed to assess the magnitude of these barriers and to identify the factors influencing healthcare access among women of reproductive age in Sub-Saharan Africa.

Methods:

This study utilized secondary data from the most recent Demographic and Health Surveys conducted between 2019 and 2023 across Sub-Saharan Africa. A weighted sample of 134,470 women of reproductive age was included in the analysis. Data were analyzed using Stata version 14, employing a multilevel mixed-effects logistic regression model. Factors associated with barriers to healthcare access were considered statistically significant at P-values less than 0.05. Adjusted odds ratios (AORs) with 95% confidence intervals (CIs) were used to interpret the results.

Result: In this study, the magnitude of barriers to accessing healthcare among reproductive age women were found to be 55.84% (95% CI: 55.57–56.10). Husband's educational status [AOR = 1.23, 95% CI: 1.15–1.30], Media exposure [AOR = 1.17, 95% CI: 1.11–1.24], ~~antenatal~~Antenatal care ~~(ANC)~~ visit ~~status~~ [AOR = 1.31, 95% CI: 1.10–1.55], and place of delivery [AOR = 1.33, 95% CI: 1.24–1.43]. Health insurance utilization [AOR = 1.18, 95% CI: 1.11–1.26], ~~wealth index status,~~Wealth index, 2.29 [2.14, 2.45], 1.57 [1.46, 1.68] and visiting a health facility within the past 12 months [AOR = 0.91, 95% CI: 0.87–0.97] were individual associated variable. In addition, place of residence [AOR = 1.30, 95% CI: 1.22–1.39], community literacy level [AOR = 1.13, 95% CI: 1.03–1.24], community wealth index [AOR = 1.12, 95% CI: 1.02–1.22], and Region were associated with barriers to accessing healthcare.

Formatted: No Spacing

Formatted: Font: Bold

Conclusion and recommendation: This study found that 55.55% of women in Sub-Saharan Africa experienced barriers to accessing healthcare. Educational status, media exposure, place of residence, antenatal care attendance, place of delivery, health insurance coverage, wealth index, recent visits to health facilities, community literacy levels, community wealth status, and regional differences were determinant factors. These findings highlight the need for Sub-Saharan African governments to prioritize these determinants when formulating policies and strategies aimed at reducing preventable barriers to healthcare access in the region.

Key words: Access to Healthcare; Barriers to Healthcare; Sub-Saharan Africa; Reproductive Age Women

Introduction

Access to healthcare is broadly defined as the ability to prevent illness, disability, and premature death, to promote and maintain health, and to attain health equity, particularly for women of reproductive age, by offering inclusive, high-quality care and the best possible availability, affordability, and accessibility of medical services[1-3]. Reproductive-age women's health is a critical global public health concern since it influences population health outcomes and fosters socioeconomic growth[4]. The advancement of socioeconomic development is significantly influenced by the health of women of reproductive age, especially in Africa. [5]. The Sustainable Development Goals (SDGs) place a strong emphasis on women's health, with Targets 3.7 and 3.8 of SDG 3 specifically focusing on achieving universal health coverage by 2030 and ensuring access to sexual and reproductive health services, including education and information on family planning[6, 7]. The Sustainable Development Goals (SDGs) explicitly target a substantial reduction in maternal mortality, with Goal 3.1 aiming to lower the global maternal mortality ratio to fewer than 70 per 100,000 live births by 2030. While these global efforts have contributed to some improvements in women's health outcomes, they have yet to fully address the root causes of maternal mortality. Achieving sustained and comprehensive progress remains a critical challenge. In 2016, Sub-Saharan Africa accounted for a disproportionately high share of the 303,000 maternal deaths reported globally, the majority of which were due to preventable causes. Moreover, non-communicable diseases continue to contribute significantly to female morbidity and mortality across nearly all countries, with a particularly severe impact in low- and middle-income settings, where many women are left with long-term disabilities [4-6]. Access to healthcare involves multiple dimensions, including financial affordability, geographic accessibility, availability of medical services, and the quality of care provided in health facilities. [8]. Access to healthcare is a key determinant of whether individuals utilize health services. Globally, approximately 400 million people lack essential healthcare, and around eight million die each year from preventable diseases—resulting in an estimated economic loss of $6 trillion in low- and middle-income countries. [6, 9, 10]. In 2015, world leaders pledged to achieve universal health coverage as part of the Sustainable Development Goals (SDGs), aiming to ensure access to affordable, high-quality medicines and financial protection for all [11, 12]. In 2017, the World Bank and WHO reported that half of the global population lacks access to essential health services. As a result, many countries in Sub-Saharan Africa have made universal healthcare a key part of their national health

strategies. However, there has been slow progress in turning this commitment into tangible results, particularly in securing national funding for equitable, high-quality healthcare, financial security, and overall health [6, 9, 13, 14]. Reproductive age women and child mortality from low family planning uptake, unsafe abortion practices, unintended pregnancies, and an increase in home births are all implications of women in reproductive age having trouble accessing health care[14]. Previous research has offered some evidence regarding the sociodemographic and economic factors that can be barriers to accessing healthcare, such as marital status, place of residence, maternal age, wealth index, educational attainment, and unemployment status. Nevertheless, women of reproductive age continue to face significant disparities in healthcare utilization and access[10, 14-16]. This study makes three unique contributions to the body of existing literature. In order to provide a current and policy-relevant analysis of the barrier to healthcare access, it first makes use of the most recent (2019–2023) Demographic and Health Survey (DHS) data. Second, our analysis takes a harmonized multi-country approach across ten sub-Saharan African nations, improving cross-national comparability and generalizability in contrast to many previous studies that are limited to single-country analyses or rely on older datasets. In order to gain a better understanding of the structural and contextual factors that influence healthcare access, the study employs a comprehensive multilevel analysis of determinants at the individual and community levels. These contributions put our research in a position to guide focused, empirically supported programs provide fair access to healthcare for reproductive age women in sub-Saharan. Therefore, this study aimed to ascertain the magnitude and potential contributing factors associated with barriers to accessing healthcare among women of reproductive age in sub-Saharan Africa.

Methods and materials

Patient and public involvement statement

Secondary data from the Demographic and Health Surveys (DHS) were used in this investigation.

Study design, study area, and period.

Data from ten Sub-Saharan African countries, collected between 2019 and 2023, were used to perform a multilevel mixed-effects analysis, based on the most recent Demographic and Health Surveys (DHS). The DHS collects community-based, cross-sectional data approximately every five years to generate updated health and health-related indicators.

Data source, study population and sampling technique

A secondary data analysis was conducted using Demographic and Health Survey (DHS) data collected between 2019 and 2023 from selected sub-Saharan African countries. The study specifically included DHS datasets from ten countries Burkina Faso, Gabon, Ghana, Kenya, Liberia, Rwanda, Sierra Leone, Tanzania, The Gambia, and Mozambique each of which had recent and publicly available survey data within the specified timeframe. The datasets were appended to assess the extent of, and factors underlying, barriers to healthcare access among women of reproductive age in sub-Saharan Africa. Each country's DHS comprises multiple datasets. The DHS employs a stratified, two-stage cluster sampling design: in the first stage, enumeration areas are selected, followed by systematic sampling of households within each selected area in the second stage.

The variable barrier to accessing health care were generated by computation of (v467b, v467c, v467d, v467f) from maternal recode (IR) data set was recoded. A binary logistic regression model was used to identify determinants a factor barrier to access the health care. Determinants of barrier to accessing the health care were reported in terms of an AOR with a significance level of (95%). In the univariate analysis, at 95% confidence intervals with a p-value of < 0.25 was considered for the candidate multivariable analysis of data. All variables with p values of <0.05 were considered statistically significant. Since DHS data are nationally representative, stratified multistage cluster sampling is required; therefor, sampling weights must be applied to produce unbiased estimates. To correctly weight DHS data, we use the sampling weights provided in the dataset, specifically the individual weight **(**v005**)** for women's individual recode files. This weight must be divided by 1,000,000 (v005/1000000). using "gen wgt=v005/1000000 Stata commands. A total weighted sample of 134,470 reproductive age women were included in the study **(Table 1).**

Table 1: The weighted Sample size for magnitude and determinants of barrier to accessing health care among reproductive age women in sub-Saharan Africa**.**

Country	Year of survey	weighted sample (n)	weighted sample (%)
Brukinefaso	2021	~~17,659~~17659	13.13
Gabon	2019-2021	~~6,507~~6507.15	4.83
Ghana	2022	~~15,014~~15014	11.16
Kenya	2022	~~16,716~~16716.36	12.43
Liberia	2019-2020	~~8,065~~8065	5.99
Rwanda	2019-2020	~~14,634~~14634	10.88
Sera lion	2019	~~15,574~~15574	11.58
Tanzania	2022	~~15,254~~15254	11.34
Gambia	2019-2020	~~11,865~~11865	8.08
Mozambique	2023	~~13,181~~13181.93	9.80
TOTAL		~~134,470~~134470	100%

Study variable

Outcome variables: The outcome of this study was barrier to access the health care. The Data in barrier of accessing the health care were collected among reproductive age women who used one of the health cares. Measurement of the outcome variables was determined by using computation of four variables; such as Distance to health facility (v467b), difficulty in obtaining money (v467c), getting permission go to the doctor (v467d) and not wanting to go alone (v467f). If a woman of reproductive age reported experiencing at least one of the following challenges, such as difficulty in obtaining money, distance to a health facility, lack of permission to seek treatment, or reluctance to go alone were considered to face significant barriers to healthcare access. These cases were coded as '1' and labeled as 'big problem.' Conversely, women who did not report any of these four barriers were considered to have no significant barriers to healthcare access. These were coded as '0' and labeled as 'no big problem'. This approach is consistent with prior literature using DHS data[17]

Independent Variable: Hence DHS data are hierarchical, independent factors come from two sources that was at individual and community levels factors were considered for this analysis. The individual-level independent variables were women's age (15-19, 20-35, 36-49), ~~women~~Women educational status (No education, Primary education , Secondary ~~and~~& Higher education), women occupation (working, not working), Religion (Muslim, Christian , Others), Wealth index (Poor, Middle, Rich) media exposure (yes, No), internet utilization (yes, No), Residency (urban, rural), Husband educational status (No education, Primary education, secondary ~~and~~& higher education), Husband occupational status (No working, Working), Currently Contraceptive (No, Yes) Visiting health facility in 12 month (No, Yes), ~~wanted pregnancy(yes, no) .~~place of delivery (Home , Institutional delivery), ANC visit (No antenatal visit, 1-4 visit, > 4 visit)

The community-level variables: ~~Community poverty~~Poverty level (Low, High), ~~Community literacy~~Literacy level (Low, High), ~~Community media~~Media exposure, (Low, High), ~~Place of residency~~Residency (Urban, rural), Region (East, west~~) in sub Saharan Africa.~~), Community ANC visit

Data processing and statistical analysis

Stata version 14 statistical software was used to clean, record, and analyze secondary DHS data sets. The variables in the DHS data are grouped into clusters, and the variables within a cluster

are more similar to each other than those in other clusters. The presumptions of equal variance across clusters and independent data were violated in order to run a typical logistic regression model. This suggests that between-cluster factors must be considered using a complex model.

Given this, multilevel mixed-effects logistic regression was used to determine the factors that associated with barrier to accessing the health care. Multilevel mixed effect logistic regression follows four models~~: the~~. The null model (only outcome variable), mode I (individual level ~~variables),~~variable such as age, Religious status, educational status, Husband educational, marital occupation, Husband occupation, marital status, Contraceptive Utilization, place of delivery, ANC visit, media exposure, Intermate utilization, visiting health facility within 12 months, health insurance , wealth index) model II ~~(~~community level variables, Region in sub-Saharan Africa, community poverty level, community media exposure, Community distance health facility, Community literacy level, Community ANC visit), and model III (both individual and community level variables~~).~~ such as age, Religious status, educational status, Husband educational, marital occupation, Husband Occupation, marital status, Contraceptive Utilization, place pf delivery, ANC visit, media exposure, Intermate utilization, visiting health facility within 12 months, health insurance , wealth index), model II (community level variables, Region in sub-Saharan Africa, community poverty level, community media exposure, Community distance health facility, Community literacy level, Community ANC visit). The null model without independent variables was used to check the variability of outcome variable across the cluster. The relationship of individual-level variables with outcome variable (Model I) and the association of community-level variables with outcome variable (Model II) were determined. In the final model, the association of both individual and community-level variables was fitted with the outcome variable.

Random effects

The intra-class correlation coefficient (ICC), proportionate change in variance (PCV), and median odds ratio (MOR) were used to quantify random effects or measures of variation of the outcome variables.

To assess the variation between clusters, the proportionate change in variance (PCV) and intra-cluster correlation coefficient (ICC) were calculated. By treating the cluster as a random variable, the ICC shows that the variation in the health care access barrier between clusters may be computed

as ~~follows; ~~follow $\sim\!\!\sim ICC = \frac{VC}{VC+3.29}\sim\!\!\sim$ $\times = \frac{\sigma^2 uo}{\sigma^2 uo + \pi^2/3} \times$ **100%.** The MOR, which is determined by using clusters as a random variable and calculating the median value of the odds ratio between the highest-risk and lowest-risk areas for health care access barriers, is calculated~~;~~ ~~MOR= $e^{0.95\sqrt{VC}}$~~. $\boldsymbol{MOR = \exp(0.6745\ \sqrt{(2 * \sigma^2 uo)})}$ Furthermore, the PCV calculates a variable that explains the variation in the degree of barriers to accessing healthcare ~~; $PCV = \frac{Vnull-VC}{Vnull} \times 100\%$;~~; $\boldsymbol{PCV = \frac{\sigma^2 uo - \sigma^2}{\sigma^2 uo} \times 100\%}$;. where VC is the cluster level variance and Vnull is the variance of the null model.

The ~~fixed effects were employed to calculate the correlation~~associations between ~~the probability of barriers to obtaining health care access and~~ independent factors at the individual and community levels~~.~~ and barriers to accessing health care were estimated using the fixed effects. It was assessed and presented using adjusted odds ratio (AOR) and 95% confidence intervals with a p-value of < 0.05. Because of the nested nature of the model, deviation = -2(log likelihood ratio) was used to compare models, and the model with the lowest deviance was selected as the best-fitted model. By evaluating the variance inflation factors, the model assumption for multi-collinearity was confirmed~~, and the results fell within an acceptable range of 1.03-1.98.~~. In addition~~.~~, Model comparison was performed using Akaike's Information Criterion (AIC). The model ~~including~~includes both random intercepts and random slopes (Model III) ~~had the lowest AIC= (~~was evaluated alongside alternative models, with AIC~~=239608.6), indicating the best balance between~~ values calculated to assess relative model fit and parsimony.

~~Patient and public involvement~~

~~Since this study data was obtain from demographic and health survey (DHS) secondary source, no patient and public were involved.~~

Ethical approval and consent to participate

This study was based on analysis of existing survey datasets in the public domain (SSA) that are freely available online with all the identifier information anonymized, no ethical approval was required

Result

In this study a total of 134,470 reproductive age women were included in Sub-Saharan African. The Mean age of participants were 28.99 (Std. Dev. 9.68) years~~, and the Variance of 93.86557.~~.

Formatted: Font: Bold

Formatted: Font color: Auto

Formatted: Font color: Custom Color(RGB(34,34,34)), Pattern: Clear (White)

Formatted: Font color: Custom Color(RGB(34,34,34)), Pattern: Clear (White)

Formatted: Font color: Custom Color(RGB(34,34,34)), Pattern: Clear (White)

Formatted: Font color: Accent 1

Formatted: Font color: Accent 1

Formatted: No Spacing, Justified, Line spacing: 1.5 lines

Formatted: Font: 12 pt, Underline color: White

More than two thirds (69.8~~)~~%) of women did not use contraception, and over 72.22% reproductive women did not use health insurance, and over half of (54.22%) women were living rural areas in sub-Saharan Africa~~.~~ **(Table 2)**

Table 2: Sociodemographic and economic characteristics of reproductive women who had barriers to accessing health care in sub-Saharan Africa.

**Variable**	**frequency(n)**	**percentage (%)**
**Individual and community level variables**		
**Maternal age**		
15-19	~~27,890~~27890.4	20.7
20-35	~~69,441~~69441.4	51.64
36-49	~~37,138~~37138.6	27.64
**Residency**		
Urban	~~61,558~~61558.03	45.78
Rural	~~72,912~~72912.41	54.22
**Religious status**		
Muslim	48834.95	41.96
Christian	46856.90	39.30
Other	~~23,524~~23524.59	19.73
**Educational Status**		
No formal education	~~35,051~~35051.70	26.06
Primary education	~~39,651~~39651.17	29.48
Secondary and ~~high~~higher education	~~59,767~~59767.57	44.44
**Husband educational**		
No formal education	~~29,414~~29414.44	36.72
Primary education	~~21,245~~21245.78	26.52
secondary and ~~above~~higher education	~~29,428~~29428.78	36.74
**~~Marital~~Maternal Occupational**		
Not working	~~50,732~~50732.90	37.81
working	~~83,417~~83417.01	62.~~18~~19
**Husband Occupation**		
Not working	~~10,390~~10390.43	13.~~0%~~00
Working	~~69,698~~69698.11	87~~.%~~.00
**~~Marital~~Maternal status**		
single	~~42,213~~42213.40	31.4
Married	~~58,262~~58262.00	43.3
Other	~~33,995~~33995.4	25.3
**Contraceptive Utilization**		
No	~~93,855~~93855.00	69.8
Yes	~~40,615~~40615.5	30.2

Formatted: Font: Times New Roman, 12 pt

Formatted: Font: Times New Roman, 12 pt

Formatted: Font: Times New Roman, 12 pt

**place of delivery**		
Home	8,2798279.46	15.5
Institution	44,97944979.28	84.5
ANC visit		
No visiting	2,0122012.84	3.77
1-4 visiting	24,45924459.34	45.92
>4 visiting	26,78626786.55	50.29
**Media exposure**		
No	34,62834628.45	25.75
Yes	99,83199831.57	74.24
**Intermate utilization**		
No	93,02093020.34	69.17
Yes	41,45041450.10	30.82
**Visiting health facility 12 month**		
No	57,13757137.73	42.49
Yes	77,33277332.71	57.50
**Health insurance**		
No	85,04485044.69	72.22
Yes	32,70932709.39	27.77
**Wealth index**		
poor	47,22747227.18	35.12
middle	25,77125771.45	19.16
HighRich	61,47161471.81	45.74
**Region in sub-Saharan Africa**		
East Africa	59,78659786.29	44.46
West Africa	74,684.1574684.16	55.5354
**Community poverty level**		
Low	70,77670776.71	52.63
High	63,69363693.73	47.47
**Community media exposure**		
Low	61,64261642.30	45.84
High	72,82872828.14	54.15
**Community distance health facility**		
Low	65,962	49.00
High	68,50865962 68508	51.00
**Community literacy level**		
Low	57,61557615.01	42.84
High	76,85576855.43	57.15
**Community ANC visit**		

Formatted Table

Formatted: Font: Times New Roman, 12 pt

Formatted: Font: Times New Roman, 12 pt

Formatted: Font: Times New Roman, 12 pt

Formatted: Font: Times New Roman, 12 pt

Low	~~77,912~~77912.32	57.96
High	~~56,491~~56491.92	42.03

In this study, the magnitude of barriers to accessing health care among reproductive age women was found to be 55.84 (95% CI: (55.57, 56.10) owing to one or more of the four factors, the most commonly cited being the difficulty in getting money and the distance from medical facilities **(Figure 1)**

Random effect (Measures of variation) and model fitness

To determine if the data supported the choice to evaluate randomness at the community level, a null model was employed. With ~~a~~the variance of 0.3784967 and a p~~-~~-value of < 0.~~000~~001, the null model's results showed that there were notable variations in the barriers to accessing healthcare among clusters. A significant amount of the variance in the outcome between clusters was jointly explained by both individual- and community-level variables, as evidenced by the intra-cluster correlation (ICC), which dropped from 10.31% in the null model to 3.96% in the fully adjusted model (model III). According to the proportionate change in variance (PCV), Model III explains the greatest percentage (64.09%) of the group-level variation in comparison to the null model, group comparison showed that, Model I (50.19%) and Model II (24.56%). This suggests that the model fits better as more predictors are included. ~~Akaike information criteria~~Model comparison using Akaike's Information Criterion (AIC) ~~Among the models evaluated,~~indicated that Model III ~~demonstrated~~achieved the best fit~~ with~~, as evidenced by the lowest AIC (239,608.6), ~~indicating a substantial improvement over~~markedly lower than both the null model (AIC = 1,128,437) and Model II (AIC = 1,127,704). Model I also showed good fit (AIC = 239,764.8), though slightly less optimal than Model III~~ **(Table 3)**.~~. Multicollinearity using variance inflation factors (VIF) indicated that all VIF values ranged from 1.03-1.98, which is within accepted range **(Table 3)**

Formatted: Font: Not Bold

Formatted: Font: +Body (Calibri), 11 pt

Table 3: Model comparison and Fitness random effect analysis for barriers of accessing health care of among reproductive age women in sub-Saharan Africa**.**

**Parameter**	**Null model**	**model I**	**model II**	**model III**
Variance	0.3784967	0.1885269	0.28553	0.1358817
ICC	10.31%	5.41%	7.98%	3.96%
MOR	1.79	1.51	1.66	1.41

PCV	Reference	50.19%	24.56%	64.09%
**Model fines**				
LLR	-90175.365	-21798.27	-86841.704	-21433.284
Deviance	1803596.73	43596.54	173683.408	42866.568
AIC	1128437	239764.8	1127704	239608.6

ICC: intra-cluster correlation**, LLR:** loglikelihood ratio**, MOR:** median odds ratio**, PCV:** proportional change in variance. **AIC:** Akaike information criteria.

Determinants of ~~individual and community level factors with barrios of~~barriers to accessing health care among reproductive age women in ~~sub~~Sub-Saharan Africa

Formatted: Font: 14 pt, Font color: Text 1

Formatted: Font: 14 pt, Font color: Text 1

Bivariable logistic regression analysis of individual and community level variables associated with barriers to accessing health care among reproductive age women in Sub-Saharan Africa

Bivariable multilevel logistic regression analysis indicated that husband's educational status, media exposure, internet utilization, place of residence, antenatal care attendance, place of delivery, health insurance coverage, community wealth index, recent health facility visits, and country category (Western vs. Eastern Africa) were associated with barriers to accessing health care among reproductive-age women at a p-value < 0.25 (**Table 4**)

Table 4: Bivariable logistic regression of individual and community level factors associated with barriers to accessing health care among reproductive age women in Sub-Saharan Africa.

Variables	Crude Odds Ratio [COR]	P. value	[95% CI]
**Maternal age**			
15-19	0.86	<0.001	[0.83, 0.89]
20-35	0.86	<0.001	[0.84, 0. 88]
36-49	1		1
**Residency**			
Urban	1		1
Rural	2.09	<0.001	[2.04, 2.14]
**Maternal education**			
No education	2.40	<0.001	[2.33, 2.46]
Primary education	1.52	<0.001	[1.48, 1.56]
Secondary & Higher	1		1
**Husband education**	2.43	<0.001	[2.35, 2.51]
No education	1.55	<0.001	[1.49, 1.60]
Primary education	1		

secondary & higher			1
**Maternal occupation**			
working	1		1
not working	0.94	<0.001	[0.92, 0.96]
**Husband occupation**			
working	1		
not working	1.07	<0.001	[1.02, 1.12]
**Religion**			
Muslim	1		1
Christian	1.32	<0.001	[1.29, 1.35]
Other	1.08	<0.001	[1.04, 1.11]
**Contraceptive utilization**			
No	1.18	<0.001	[0.82, 0.86]
Yes	1		1
**Place of delivery**			
Home	1.84	<0.001	[1.75, 1.93]
Institution	1		1
**ANC visit**			
No visit	1.16	<0.002	[1.05, 1.27]
1-4 visit	1.05	<0.003	[1.01, 1.09]
> 4 visits	1		1
**Marital status**			
single	1		
Married	1.23	<0.001	[1.20, 1.26]
Other	1.32	<0.001	[1.29, 1.36]
**Media exposure**			
No	1.90	<0.001	[1.85, 1.95]
Yes	1		1
**Internet utilization**			
No	2.321822	<0.001	[2.26, 2.37]
Yes	1		1
**Visiting health facility 12 month**			
No	1.02	<0.046	[1.00, 1.04]
Yes	1		1
**Health insurance**			
No	1.21	<0.001	[1.18, 1.25]
Yes	1		1
**Wealth index**			
poor	3.58	<0.001	[3.49, 3.67]
middle	2.08	<0.001	[2.02, 2.14]
Rich	1		1

**Region SSA**			
East Africa	1.68	<0.001	[1.65, 1.72]
West Africa	1		1
**community ANC visit**			
Low	0.86	<0.001	[0.85, 0.88]
High	1		1
**community wealth index**			
Low	0.69	<0.001	[0.67, 0.70]
High	1		
**Community media exposure**			
Low	0.73	<0.001	[0.72, 0.75]
High	1		1
**Community literacy level**			
Low	0.77	<0.001	[0.75, 0.79]
High	1		1

Multivariable multilevel logistic regression analysis of individual and community level factors associated with Barriers to health care access among reproductive Age Women in Sub~~-~~Saharan Africa.

In the final fitted model of multivariable multilevel logistic regression, Husband educational status, media exposure, ~~internet utilization,~~ place of residency, ~~ANC~~Antenatal care visit~~ status~~, place of delivery, health insurance utilization~~ status~~, wealth index status, visiting health facility within 12 months, and country category (western, eastern Africa) were significantly associated with barriers of accessing health care among reproductive age women in sub-Saharan Africa. The odds of barrier of accessing health care were 1.30 times higher among reproductive women living in rural residency compared to women living in urban area ~~(~~[**AOR=**~~-~~1.30, 95 CI: (1.22, 1.39~~).~~)]. and the odds of barrier of accessing health care were 1.23 ~~and 1.21~~ times more likely to among reproductive age women whose husband had no education ~~and primary education~~ compared to women whose husband had secondary and ~~above educational level respectively~~ higher education [**AOR**=1.23, 95 CI: (1.15, 1.30~~,) and (**AOR**=1.21, 95% CI: (1.31, 1.41).~~,)]. In addition, odds of barrier of accessing health care were 1.33 times more likely to occur women delivered at home compared to women who had institutional delivery. **(AOR**=1.33, 95% CI: (1.24, 1.43) and the odds of barrier of accessing health care were 1.31~~ and 1.13~~ times higher among women who had no history of antenatal visit ~~and women who had < 4 ANC visit~~ compared to women who had more than four ANC visit~~ Respectively. (~~. [**AOR**= 1.31, 95% CI: (1.10, 1.55~~) and (**AOR**= 1.13, 95% CI: (1.07,1.19) and the~~)]. The odds of the barrier of accessing health care among reproductive age woman who had no media exposure ~~and intranet access~~ were 1.17 ~~and 1.68~~

Formatted: Line spacing: Multiple 1.15 li

Formatted: Font color: Text 1

Formatted: Font color: Auto

Formatted: Font color: Black

Formatted: Font color: Auto

times more likely to compared with -a woman who had media exposure and internet access respectively ([AOR=1.17, 95% -CI: (1.11, 1.24) and (AOR= 1.68, 95% CI: (1.57, 1.79).)] and the barrier of accessing health care among reproductive age woman who were not visiting health facility within 12 months 9-% times less likely compared to women who had visiting health care facility within 12 months of study period ([AOR=0.91, 95% CI: (0.87, 0.97).)]. The odds of perceived barrier of accessing health care among reproductive age woman who had no health insurance utilization were 1.18 times more likely to compared with a woman who had used health insurance ([AOR=1.18 95% CI: (1.11, 1.26))] and The odds of perceived barrier of accessing health care service among reproductive age woman who were classified under poor and middle wealth status were 2.29 and 1.57 times more likely barries accessing health care challenge compared to reproductive women who were classified under rich wealth status category([AOR=2.29 95% CI: -(2.14, 2.45))] and ([AOR=1.57., 95% CI: -(1.46, 1.68).)]. The odds of perceived barrier of accessing health care service among reproductive age woman who reside in west Africa region were 2.38 times more likely barriers accessing health care compared to east Africa region category (. [AOR=2.38, 95% CI: (2.21, 2.55))] **(Table 4)5).**

Table 4: 5: Multivariable multilevel logistic regression analysis of individual level and community level variablefactors associated with barriers to accessing health care among reproductive age women in sub-Sub-Saharan Africa.

**Individual and community level factors**	**Model I AOR ([95% CI)]**	**Model II AOR ([95% CI)]**	**Model III AOR ([95% CI)]**
**Maternal age**			
15-19	0.96([0.86, 1.07)]		0.96 ([0.86,1.08)]
20-35	1.06([1.01,1.13)]		1.04 ([0.99, 1.11)]
36-45	1		1
**Residency**			
Urban		1	1
Rural		2.29 ([2.24, 2.35)]	**1.30 ([1.22, 1.39)]**
**Maternal education**			
No education	1.10 ([1.02, 1.18)]		1.04 ([0.97, 1.11)]
Primary education	0.93 ([0.87, 0.99)]		1.06 ([0.99, 1.14)]
Secondary and above	1		1
**Husband education**			
No education	1.26 ([1.19,1.35)]		**1.23 ([1.15, 1.30)]**
Primary education	1.04([0.97,1.12)]		1.23 ([0.67, 1.22)]
secondary & higher education	1		1

Formatted: Font color: Black

Formatted: Font: 14 pt, Bold, Font color: Text 1

Formatted: Justified

Formatted: Normal

Formatted Table

Formatted: Line spacing: Multiple 1.15 li

Formatted: Font: +Body (Calibri), 11 pt

Formatted: Indent: Left: 0", Line spacing: Multiple 1.15 li

**~~Marital~~Maternal occupation**	1		
working	0.99 ~~(~~ [0.94,		1
not working	1.04~~)~~]		**1.02 ~~(~~[1.01, 1.11~~)~~]**
**Husband occupation**			
working	1		1
not working	0.90 ~~(~~[0.83, 0.97~~)~~]		1.00 ~~(~~[0.92, 1.08~~)~~]
**Religion**			
Muslim	1		1
Christian	1.62 ~~(~~[1.53, 1.71~~)~~]		0.46 ~~(~~[0.57, 1.94~~)~~]
Other	1.22 ~~(~~[1.13, 1.31~~)~~]		1.36 ~~(~~[0.75, 1.46~~)~~]
**Contraceptive utilization**			
No	1.11 ~~(~~[1.05,1.17]		1.03 ~~(~~[0.98, 1.08~~)~~]
Yes	1		1
**Place of delivery**			
home	1.30 ~~(~~[1.21, 1.40~~)~~]		**1.33 ~~(~~[1.24, 1.43~~)~~]**
Institution	1		**1**
**~~ANC visit~~**			
**Antenatal Care Visit**			
No	1.01 ~~(~~[0.85, 1.19~~)~~]		**1.31 ~~(~~[1.10, 1.55~~)~~]**
1-4 visit	0.94 ~~(~~[0.89, 0.99~~)~~]		1.13 ~~(~~[0.37, 1.19~~)~~]
> 4 visits	1		1
**Media exposure**			
No	1.14 ~~(~~[1.08, 1.21~~)~~]		**1.17 ~~(~~[1.11, 1.24~~)~~]**
Yes	1		1
**Internet utilization**			
No	1.69 ~~(~~[1.58, 1.80~~)~~]		1.68 ~~(~~[0.67, 1.79~~)~~]
Yes	1		1
**Visiting health facility 12 month**			
No	0.92 ~~(~~[0.87, 0.98~~)~~]		**0.91 ~~(~~[0.87, 0.97~~)~~]**
Yes	1		1
**Health insurance**			
No	1.26 ~~(~~[1.19, 1.34~~)~~]		**1.18 ~~(~~[1.11, 1.26~~)~~]**
Yes	1		1
**Wealth index**			
poor	2.80 ~~(~~[2.63, 2.97~~)~~]		**2.29 ~~(~~[2.14, 2.45~~)~~]**
middle	1.79 ~~(~~[1.68, 1.92~~)~~]		**1.57 ~~(~~[1.46, 1.68~~)~~]**
Rich	1		1
**Region SSA**		~~0.50 (0.49, 0.52)~~	**~~0.42 (0.39, 0.45)~~**
East Africa		~~1~~1	**1**
West Africa		2.0 [1.92-2.04]	**2.38 [2.21, 2.55]**
**community ANC visit**			
Low		1.00 ~~(~~[0.93, 1.09~~)~~]	0.93 ~~(~~[0.86, 1.00~~)~~]
High		1	1

Formatted: Font: Bold

Formatted: Font: Bold

**community wealth index**			
Low		0.75 ([0.70, 0.81)]	**1.12 ([1.02, 1.22)]**
High		1	1
**Community media exposure**			
Low		1.01 ([0.92, 1.10)]	0.94 ([0.86, 1.03)]
High		1	1
**Community literacy level**			
Low		1.10([1.00, 1.20)]	**1.13 ([1.03, 1.24)]**
High		1	**1**

Table 5: Key Individual and Community-Level Determinants of Barriers to Healthcare Access Among Women of Reproductive Age in Sub-Saharan Africa.

**Individual and community level factors**	**Model I AOR (95% CI)**	**Model II AOR (95% CI)**	**Model III AOR (95% CI)**
**Residency**			
Urban		1	1
Rural		2.29 (2.24, 2.35)	**1.30 (1.22, 1.39)**
**Husband education**			
No education	1.26 (1.19,1.35)		**1.23 (1.15, 1.30)**
Primary education	1.04(0.97,1.12)		1.23 (0.67, 1.22)
secondary education	1		1
**Marital occupation**			
working	1		1
not working	0.99 (0.94 1.04)		**1.02 (1.01, 1.11)**
**Place of delivery**			
home	1.30 (1.21, 1.40)		**1.33 (1.24,1.43)**
Institution	1		**1**
**ANC visit**			
No	1.01 (0.85, 1.19)		**1.31(1.10,1.55)**
1-4 visit	0.94 (0.89, 0.99)		1.13(0.37,1.19)
> 4 visits	1		1
**Media exposure**			
No	1.14 (1.08, 1.21)		**1.17 (1.11, 1.24)**
Yes	1		1
**Visiting health facility 12 month**			
No	0.92 (0.87, 0.98)		**0.91(0.87, 0.97)**
Yes	1		1
**Health insurance**			
No	1.26 (1.19, 1.34)		**1.18 (1.11,1.26)**
Yes	1		1

Formatted: Font: Bold

Formatted: Left, Line spacing: single

**Wealth index**			
poor	2.80 (2.63, 2.97)		**2.29 (2.14, 2.45)**
middle	1.79 (1.68, 1.92)		**1.57 (1.46, 1.68)**
Rich	1		1
**Region SSA**			
East Africa		0.50 (0.49, 0.52)	**0.42 (0.39, 0.45)**
West Africa		1	1
**community wealth index**			
Low		0.75 (0.70, 0.81)	**1.12 (1.02,1.22)**
High		1	1
**Community literacy level**			
Low		1.10(1.00, 1.20)	**1.13(1.03,1.24)**
High		1	**1**

Model I: *individual level variable,* **Model II:** *Community level variable,* **Model III**: *Individual and community level variable*

Discussion

Barriers to accessing health care have contributed to maternal and child mortality and morbidity in developing countries such as those in Sub-Saharan Africa. This study aimed to assess the magnitude and determinants of barriers to health care access among women of reproductive age in Sub-Saharan Africa.

In this study, the magnitude of barriers to accessing health care among reproductive age women was found to be 55.84 (95% CI: (55.57, 56.10) owing to one or more of the four factors, the most commonly cited being the difficulty in getting money and the distance from medical facilities. This finding is lower than the previous study conducted in South Africa 65%% [18, 19], and Tanzania 65% [15], Ethiopia 71.8%,70.2, 69.9%[1, 20, 21], (SSA) 60.4%, 61.5% [17]. this variation could be due to the fact that socio-economic and variability in health infrastructure and health system policy, women's attitudes towards health care seeking behavior and cultural differences across countries in sub-Saharan Africa, moreover, this discrepancy might be due to study period, this study includes, the most recent DHS data set from 2019-2023. On the other hand, the finding of this study is similar to but slightly higher than in previous study in Gambia 45.5%[22], Ghana (51%)[23]. this variation could be due to the fact that socio-economic and variability in health infrastructure and health system policy, women's attitudes towards health care seeking behavior and cultural differences across countries in sub-Saharan Africa.

The odds of barrier of accessing health care were 1.30 times higher among reproductive women living in rural residency compared to women living in urban area. This finding is supported by previous studies conducted in Ethiopia [1, 20], guinea[24], Gambia, Tanzania and Ghana [15, 22, 25] respectively. Sub-Saharan[23], East Africa[26]. This association may be, due to the fact that rural areas have low health infrastructure i.e. inaccessibility of health facilities, less privileged and the influences of socio-cultural practices that need women to seek permission from their husband to visit the facility or women's decisions for seeking healthcare services is under their husband/partners control. Moreover, male involvement and support for women health care access are limited[1, 14, 23, 26]. This implies that the government should expand infrastructure services in rural area and encourage male involvement and support for improving health care access for reproductive age women[14].

The odds of perceived barrier of accessing health care were 1.23 and 1.21 times more likely to among reproductive age women whose husband had no education and primary education compared to women whose husband had secondary and above educational level respectively. This find was consistent with previous study conducted in east Africa, Ethiopia, Tanzania, Benin, Bangladesh respectively [1, 15, 20, 26, 27]. The possible explanation could be in better access to the healthcare among women with educated husbands might be due to better participation and

involvement of husbands in their families' health[23]. Women whose partners are educated are also likely to be informed regarding their fundamental human rights and may have higher health literacy. As a result, they are more likely to deal with any form of barrier to healthcare, compared to their counterparts who are less educated and may have lower health literacy [14, 15, 20, 28].

The odds of barrier to accessing health care were 1.33 times more likely to occur women delivered at home compared to women who had institutional delivery. This evidence was supported by study done in Senegal[29]. The plausible justification might be women who delivered at home may not get health information/counselling from healthcare providers that empower them to use other maternal healthcare services and women who deliver at home may face varous barriers to accessing health care such as financial constraint, lack of transportation, limited autonomy in healthcare decision and sociocultural norms that discourage institutional delivery. Moreover, importance of enhancing health system outreach, strengthen community-based education, and addressing sociocultural and socioeconomic disparities that affect health seeking behavior. On the other hand, the odds of barrier of accessing health care among woman who had no media exposure were 1.17 times more likely to compared with a woman who had media exposure. This evidence was supported by study done in Ethiopia[30], Nepal[31]. This could be the power of mass media in disseminating information concerning maternal health that may enhance women's knowledge and attitude towards health care access and utilization[31]. Barrier of accessing health care among woman who were not visiting health facility within 12 months were 9 % times less likely compared to women who had visiting health care facility within 12 months of study period. The odds of barrier to accessing health care among woman who had no health insurance utilization were 1.18 times more likely to compared with a woman who had used health insurance. This finding was supported with study done in Ethiopia[1], sub-Saharan Africa[23], Tanzania[15], Ghana[25]. The possible reason might be due to the fact that, individuals who were not had health insurance faced for unexpected catastrophic expenditure and difficulties in obtaining money for consultation of the doctor[1]. The odds of barrier to accessing healthcare among woman who were classified under poor and middle wealth status were 2.29 and 1.57 times more likely accessing health care difficulty compared to women who were classified under rich wealth index category**.** This evidenced was supported with study done in Ethiopia, [1, 30, 32, 33] Tanzania[15] Uganda[34], sub Saharan Africa[14]. This finding might be explained by the fact that high out-of-pocket expenses and a lack of accessibility to healthcare facilities pose significant obstacles for middle-class and impoverished

women in sub-Saharan Africa. Many people in the region cannot afford healthcare services because over 40% of health costs are paid for directly by individuals[35-38]

The odds of barrier of accessing health care among woman who live west Africa region were 2.38 times more likely barriers to accessing health care compared to east Africa region. According a recent joint report from the World Health Organization and the United Nations states that high out-of-pocket costs, a lack of adequate healthcare infrastructure, and other sociocultural barriers make it more difficult for women of reproductive age in West African nations such as Nigeria, Mali, and Sierra Leone to access maternal healthcare. On the other hand, East African countries like Rwanda, Kenya, and Ethiopia have strengthened their community health initiatives and gender-inclusive policies, which have improved women's autonomy in accessing healthcare services[39]. Furthermore, our current findings are supported by recent Demographic and Health Survey (DHS) program data (2022–2023), which indicate that women in West African countries are 2-3 times more likely to report delays in accessing healthcare due to cost, shortages of healthcare personnel, and long distances to health facilities. In contrast, East African countries, particularly Uganda and Tanzania, have strengthened their health insurance systems and rural outreach initiatives. Additionally, the World Bank's 2023 Health Service Coverage Index reveals that women in West Africa have significantly lower access to family planning, skilled birth attendance, and other essential reproductive health services[22, 40, 41]

Strength and limitation

This strength of study nationally representative data to evaluate contextual and individual characteristics related to barriers to healthcare access among reproductive age women in the SSA. Thus, findings can therefore, generalized to all women in their reproductive age in SSA, in order to account for the clusters within the sample, the study also employed advanced statistical models. ~~This study has its own limitation, that is, the findings do not establish a cause and effect relationship due to the cross-sectional nature of the data/surveys and health-worker related factors could not be accounted for in this study.~~This study has several limitations. The cross-sectional design restricts the ability to draw causal inferences, and health worker–related factors were not incorporated into the analysis. Furthermore, reliance on self-reported data may introduce recall or social desirability bias; future research should consider integrating objective measures, such as wearable devices, as demonstrated by Zhao et al. (2025)[42]

Formatted: Font: +Body (Calibri), 11 pt

Conclusion and Recommendation

This study found that 55.55% of reproductive age women had barriers to accessing healthcare in SSA. Husband educational status, media exposure, internet utilization, place of residency, ANC visit status, place of delivery, health insurance utilization status, wealth index status, visiting health facility within 12 months, community literacy level and community wealth index and country category (western, eastern Africa) were significantly associated with barriers of accessing health care. Therefore, the Government and ministry of health in Sub-Saharan Africa countries should taking in to consideration those factors while designing policies and strategies targeting to health care access and tackling the preventable barriers to accessing health care in sub-Saharan Africa.

Declaration

Ethical approval and consent to participate

This study was based on analysis of existing survey datasets in the public domain (SSA) that are freely available online with all the identifier information anonymized, no ethical approval was required

Consent for publication

Not applicable

Data and material availability

Demographic and Health Survey were used, and it is publicly available online at (https://www.dhsprogram.com).

Competing interest

Authors Declared that, there is no competing interest.

Funding

Not applicable

Author's contributions

GAZ: involved in designing, data extraction, data analysis, interpretation, report and manuscript writing.

BT: involved in data analysis, interpretation, and, and manuscript writing.

BT: involved in analysis,

TTT: involved in designing the study, interpretation, report and

BSW: involved in analysis, and result interpretation

AFZ: involved in designing and interpretation study

EGM: analysis and manuscript writing

MW: Result interpretation

AA: manuscript writing: involved in data extraction

MSA: involved manuscript writing

Acknowledgements

We would like to say the DHS programs for allowing us to use the relevant DHS dataset in this study.

Reference

1. Tamirat, K.S., Z.T. Tessema, and F.B.J.B.H.S.R. Kebede, Factors associated with the perceived barriers of health care access among reproductive-age women in Ethiopia: a secondary data analysis of 2016 Ethiopian demographic and health survey. 2020. **20**: p. 1-8.
2. Erasmus, M.O., The barriers to access for maternal health care amongst pregnant adolescents in the Mitchells plain sub-district. 2017.
3. Washington, D.L., et al., Access to care for women veterans: delayed healthcare and unmet need. 2011. **26**: p. 655-661.
4. Regan, L.J.B.M.A., Addressing unmet needs in global women's health. 2018.
5. Izugbara, C., E. Covan, and E. Fugate-Whitlock, Women's health in Africa: Issues, challenges and opportunities. 2017: Routledge.
6. Organization, W.H., Primary health care on the road to universal health coverage: 2019 global monitoring report: Executive summary. 2019, World Health Organization.
7. Badiuzzaman, M., S.M. Murshed, and M.J.T.J.o.D.S. Rieger, Improving maternal health care in a post conflict setting: evidence from Chittagong Hill tracts of Bangladesh. 2020. **56**(2): p. 384-400.
8. Peters, D.H., et al., Poverty and access to health care in developing countries. 2008. **1136**(1): p. 161-171.
9. Alkire, B.C., et al., The economic consequences of mortality amenable to high-quality health care in low-and middle-income countries. 2018. **37**(6): p. 988-996.
10. Nyakang'o, S.B. and A.J.M. Booth, Women's perceived barriers to giving birth in health facilities in rural Kenya: A qualitative evidence synthesis. 2018. **67**: p. 1-11.
11. Colglazier, W.J.S., Sustainable development agenda: 2030. 2015. **349**(6252): p. 1048-1050.

Formatted: Font: 12 pt, Not Bold

Formatted: Line spacing: single

12. Cahill, C.M., et al., Preoperative anemia management program reduces blood transfusion in elective cardiac surgical patients, improving outcomes and decreasing hospital length of stay. 2021. **61**(9): p. 2629-2636.
13. Peeri, N.C., et al., The SARS, MERS and novel coronavirus (COVID-19) epidemics, the newest and biggest global health threats: what lessons have we learned? 2020. **49**(3): p. 717-726.
14. Tessema, Z.T., et al., Determinants of accessing healthcare in Sub-Saharan Africa: a mixed-effect analysis of recent Demographic and Health Surveys from 36 countries. 2022. **12**(1): p. e054397.
15. Bintabara, D., K. Nakamura, and K.J.B.o. Seino, Improving access to healthcare for women in Tanzania by addressing socioeconomic determinants and health insurance: a population-based cross-sectional survey. 2018. **8**(9): p. e023013.
16. Kea, A.Z., et al., Exploring barriers to the use of formal maternal health services and priority areas for action in Sidama zone, southern Ethiopia. 2018. **18**: p. 1-12.
17. Zegeye, B., et al., Breaking barriers to healthcare access: a multilevel analysis of individual-and community-level factors affecting women's access to healthcare services in Benin. 2021. **18**(2): p. 750.
18. Silal, S.P., et al., Exploring inequalities in access to and use of maternal health services in South Africa. 2012. **12**: p. 1-12.
19. Tsawe, M. and A.S.J.B.r.n. Susuman, Determinants of access to and use of maternal health care services in the Eastern Cape, South Africa: a quantitative and qualitative investigation. 2014. **7**: p. 1-10.
20. Zegeye, B., et al., Barriers and facilitators to accessing health care services among married women in Ethiopia: a multi-level analysis of the Ethiopia demographic and health survey. 2021.
21. Demsash, A.W., B. Chakilu, and A. Mazengia, Knowledge Sharing Practice and Its Associated Factors Among Healthcare Providers at University of Gondar Comprehensive Specialized Hospital, North West Ethiopian: Cross-sectional Study. 2021.
22. Tsegaw, M., B. Mulat, and K.J.B.o. Shitu, Problems with accessing healthcare and associated factors among reproductive-aged women in the Gambia using Gambia demographic and Health Survey 2019/2020: a cross-sectional study. 2023. **13**(8): p. e073491.
23. Seidu, A.-A.J.P.O., Mixed effects analysis of factors associated with barriers to accessing healthcare among women in sub-Saharan Africa: Insights from demographic and health surveys. 2020. **15**(11): p. e0241409.
24. Seidu, A.-A., et al., Individual and contextual factors associated with barriers to accessing healthcare among women in Papua New Guinea: insights from a nationwide demographic and health survey. 2021. **13**(6): p. 573-585.
25. Seidu, A.-A., et al., Barriers to accessing healthcare among women in Ghana: a multilevel modelling. 2020. **20**: p. 1-12.
26. Minyihun, A., Z.T.J.R.M. Tessema, and H. Policy, Determinants of access to health care among women in East African countries: a multilevel analysis of recent demographic and health surveys from 2008 to 2017. 2020: p. 1803-1813.
27. Sen, K.K., et al., Do education and living standard matter in breaking barriers to healthcare access among women in Bangladesh? 2023. **23**(1): p. 1431.

28. Dimbuene, Z.T., et al., Women's education and utilization of maternal health services in Africa: a multi-country and socioeconomic status analysis. 2018. **50**(6): p. 725-748.
29. Zegeye, B., et al., Predictors of institutional delivery service utilization among women of reproductive age in Senegal: a population-based study. 2021. **79**: p. 1-11.
30. Demsash, A.W., A.D.J.B.H. Walle, and C. Informatics, Women's health service access and associated factors in Ethiopia: application of geographical information system and multilevel analysis. 2023. **30**(1).
31. Tamang, T.M. Factors associated with completion of continuum of Care for Maternal Health in Nepal. in IUSSP XXVIII International Population Conference, Cape Town, South Africa. 2017.
32. Fentie, E.A., et al., Spatial distribution and determinants of barriers of health care access among female youths in Ethiopia, a mixed effect and spatial analysis. 2023. **13**(1): p. 21517.
33. Alamneh, T.S., et al., Barriers for health care access affects maternal continuum of care utilization in Ethiopia; spatial analysis and generalized estimating equation. 2022. **17**(4): p. e0266490.
34. Kalule-Sabiti, I., A.Y. Amoateng, and M.J.A.P.S. Ngake, The effect of socio-demographic factors on the utilization of maternal health care services in Uganda. 2014: p. 515-525.
35. Shiferaw, S., et al., Why do women prefer home births in Ethiopia? 2013. **13**: p. 1-10.
36. Yaya, S., G. Bishwajit, and V.J.B.g.h. Shah, Wealth, education and urban–rural inequality and maternal healthcare service usage in Malawi. 2016. **1**(2): p. e000085.
37. Voto, T.P., B.E. Voto, and N.J.E. Ngepah, The Impact of Out-of-Pocket Health Expenditure and Public Health Expenditure on Poverty in Sub-Saharan Africa. 2025. **13**(5): p. 134.
38. Alamneh, T.S., et al., Socioeconomic inequality in barriers for accessing health care among married reproductive aged women in sub-Saharan African countries: a decomposition analysis. 2022. **22**(1): p. 130.
39. AKINWALE, A.A.J.I.J.o.I.R. and A. Studies, Gender equity and social progress: empowering women and girls to drive sustainable development in Sub–Saharan Africa. 2023.
40. Arhin, K., E.F. Oteng-Abayie, and J.J.H.e.r. Novignon, Assessing the efficiency of health systems in achieving the universal health coverage goal: evidence from Sub-Saharan Africa. 2023. **13**(1): p. 25.
41. Muhajarine, N., et al., Inequalities and factors associated with maternal healthcare services utilisation in Mozambique: evidence from the Demographic and Health Survey 2022–2023. 2025. **10**(5).
42. Zhao, Y., et al., Highly sensitive, wearable piezoresistive methylcellulose/chitosan@MXene aerogel sensor array for real-time monitoring of physiological signals of pilots. 2025. **68**(2): p. 542-551.

Figure legend

Fig 1: Barriers to accessing health care and determinant factor among reproductive age women in sub-Saharan Africa

Formatted: Space After: 0 pt, Line spacing: single

Formatted: Normal, Justified

Formatted: Font: 12 pt

PLOS ONE Decision: Revision required [PONE-D-24-28999] - [EMID:170305376cfa5b87]

PLOS ONE

Tue, May 27, 6:19 AM (4 days ago)

Response to Editor and reviewers' comments

ID: [PONE-D-24-28999] - [EMID:170305376cfa5b87]

Title: Barriers to accessing health care among women in sub-Saharan Africa: Multilevel analysis of recent demographic and health surveys from 2019-2023.

Journal: PLOS ONE

Name of Authors: Gebreeyesus Abera Zeleke[1]*, Enyew Getaneh Mekonen[1], Bewuketu Terefe[2], Mulugeta Wassie[3], Agazhe Aemro[3], Alebachew Ferede Zegeye[3], Mohammed Seid Ali[4], Berhan Tekeba[4], Tadesse Tarik Tamir[4], Belayneh Shetie Workneh[5].

Subject: Submission of revised manuscript

Yours sincerely

Gebreeyesus Abera Zeleke (on behalf of all authors) university of Gondar, Gondar Ethiopia.

Response to Editors and reviewer comments

Thank you for submitting your manuscript to PLOS ONE. After careful consideration, we feel that it has merit but does not fully meet PLOS ONE's publication criteria as it currently stands. Therefore, we invite you to submit a revised version of the manuscript that addresses the points raised during the review process.

Response: Dear editor, thank you for considering my work in your esteemed journal, we truly appreciate your time and support throughout the editing and review process.

Journal Requirements:

When submitting your revision, we need you to address these additional requirements.

1. Please ensure that your manuscript meets PLOS ONE's style requirements, including those for file naming. The PLOS ONE style templates can be found at

Response: Dear editor, thank you for your guides. The authors ensured, the manuscript meets PLOS ONE's style requirements

2. Please provide a complete Data Availability Statement in the submission form, ensuring you include all necessary access information or a reason for why you are unable to make your data freely accessible. If your research concerns only data provided within your submission, please write "All data are in the manuscript and/or supporting information files" as your Data Availability Statement.

Response: Dear editor, thank you for your recommendation. The authors ensured that, all data are in the manuscript and/or supporting information files in submission form on which the data freely accessible

3. Please amend either the abstract on the online submission form (via Edit Submission) or the abstract in the manuscript so that they are identical.

Response: Dear editor, thank you for your comment. The authors ensured that, abstracts on the submission form and abstract in the manuscript was corrected to be identical. Dear editor Kindly see amendment on abstract section and online submission form.

Additional Editor Comments:
The present manuscript tackles a prominent and present-day public health concern barrier to accessing healthcare services among women of reproductive age within sub-Saharan Africa employing nationally representative DHS datasets and multilevel modeling. The study's objective is praiseworthy and has the potential to contribute to the development of policy. However, the manuscript necessitates substantial revision before publication.

Response: Dear editor, thank you for your comment. The authors ensured that the comment that raised by editor and reviewer properly fix.

Reviewers' comments:

Reviewer's Responses to Questions

1. Is the manuscript technically sound, and do the data support the conclusions?

The manuscript must describe a technically sound piece of scientific research with data that supports the conclusions. Experiments must have been conducted rigorously, with appropriate controls, replication, and sample sizes. The conclusions must be drawn appropriately based on the data presented.

Reviewer #1: Yes

Reviewer #2: Yes

Response: Dear reviewers', thank you for your constructive response

---

2. Has the statistical analysis been performed appropriately and rigorously?

Reviewer #1: No

Reviewer #2: No

Response: Dear reviewers', thank you for your comment. Statistical analysis was conducted appropriately and rigorously. Multilevel logistic regression was employed to account for the hierarchical nature of the data. Sampling weights, stratification, and clustering were incorporated to ensure nationally representative estimates. The analysis was performed using Stata version 14, and the results are presented with adjusted odds ratios and 95% confidence intervals. Model assumptions were also checked. Dear reviewer, kindly see on page 5-line number 119-123 and on page 6-line number 140-145 and on page number 8 and line number 182-205.

---

3. Have the authors made all data underlying the findings in their manuscript fully available?

The PLOS Data policy requires authors to make all data underlying the findings described in their manuscript fully available without restriction, with rare exception (please refer to the Data Availability Statement in the manuscript PDF file). The data should be provided as part of the manuscript or its supporting information, or deposited to a public repository. For example, in addition to summary statistics, the data points behind means, medians and variance measures should be available. If there are restrictions on publicly sharing data—e.g. participant privacy or use of data from a third party—those must be specified.

Reviewer #1: No

Reviewer #2: Yes

Response: Dear reviewers', thank you for your comment. The data were fully provided in the body manuscript and supporting information file.

---

4. Is the manuscript presented in an intelligible fashion and written in standard English?

PLOS ONE does not copyedit accepted manuscripts, so the language in submitted articles must be clear, correct, and unambiguous. Any typographical or grammatical errors should be corrected at revision, so please note any specific errors here.

Reviewer #1: No

Reviewer #2: No

Response: Dear Reviewers, thank you for your comments. We, the authors, have revised the manuscript by addressing the language and grammatical issues through careful paraphrasing and editing.

Review Comments to the Author

Please use the space provided to explain your answers to the questions above. You may also include additional comments for the author, including concerns about dual publication, research

ethics, or publication ethics. (Please upload your review as an attachment if it exceeds 20,000 characters)

Reviewer #1: This manuscript tackles an important public-health issue—identifying barriers to healthcare access among reproductive-age women in sub-Saharan Africa (SSA) using pooled DHS data from 2019–2023. The large sample size (N≈134,000), the inclusion of both individual- and community-level variables, and the application of multilevel mixed-effects logistic regression all strengthen its potential impact. However, before this work can be considered for publication, several critical concerns must be addressed:

Response: Dear Reviewer, thank you for your thoughtful and encouraging feedback. We acknowledge that several critical concerns must be addressed, and we are committed to revising the manuscript accordingly. Detailed responses are provided for each point to enhance the clarity, rigor, and overall quality of the manuscript.

Comment 1. The manuscript does not explain how DHS sampling weights, clustering, and stratification were incorporated (e.g., in Stata's svy: framework or via robust standard e the issue.rrors). Please specify your approach in the Methods.

Response: Dear Reviewer, thank you for the valuable comment. Since DHS data are nationally representative**,** stratified multistage cluster sampling is required; therefor, sampling weights must be applied to produce unbiased estimates. To correctly weight DHS data, we use the sampling weights provided in the dataset, specifically the individual weight **(**v005**)** for women's individual recode files. This weight must be divided by 1,000,000 (v005/1000000). using "gen wgt=v005/1000000 Stata commands. Clustering and stratification are key components of the complex sampling design used in DHS surveys to ensure national representativeness and statistical efficiency. In DHS, clustering refers to the selection of enumeration areas **(**EAs) while Stratification involves dividing the population into distinct subgroups **(**strata) before sampling. In DHS, stratification is usually based on geographic regions (like provinces or states) and urban/rural residence. Dear reviewer, please see amendment on page 6-line number 141-146.

Comment 2. Multicollinearity is mentioned but actual VIF values are absent. Provide VIFs for all predictors. Include goodness-of-fit metrics (AIC, ΔDeviance) and a brief discussion of residual or influence diagnostics.

Response: Dear Reviewer, thank you for your insightful comment, we have added VIF values for all predictors (all <5), indicating no multicollinearity and used and AIC to compare multilevel models with varying fixed and random effects structures. The model including random intercepts and slopes (Model III) had the lowest AIC (AIC =239608.6), indicating the best balance between model fit and parsimony. kindly see amendment on age 9-line number 210-211 and on page 12-line number 245-246. And for farther information see table under supporting information file.

Comment 3. Several reported CIs are inverted (e.g., AOR=1.23, 95% CI: 1.30–1.15). Please correct all interval bounds and verify consistency across text, tables, and figures.

Response: Dear reviewer thank you for pointing this. We have reviewed all reported confidence intervals across the text, tables, and figures and corrected any inverted bounds to ensure proper formatting (e.g., lower bound listed first, followed by the upper bound). All intervals have been verified for consistency and accuracy throughout the manuscript. Thank you again.

Comment 4. I recommend citing Zhao et al. (2025) "Applied statistical methods for identifying features of heart rate that are associated with nicotine vaping". Although the outcome differs, that paper presents a robust five-step workflow. Suggest add "The five-step workflow for extracting event-related features from physiological time-series data, originally developed by Zhao et al, inspired our adaptation of mixed-effects modeling to detect 'before/after' patterns around reported access barriers."

Response: Dear reviewer thank you for the valuable suggestion. We appreciate the recommendation to cite Zhao et al. (2025). Although our outcome of interest differs, we agree that their five-step workflow offers a robust and generalizable framework for extracting features from physiological time-series data.

Comment 5. The manuscript contains numerous grammatical errors and non-standard expressions (e.g., "not beg problem," "were significantly contribute"). Engage a native English speaker or professional editor to ensure clarity.

Response: Dear Reviewer**,** thank you for comment about grammatical issues and non-standard expressions in the manuscript. We apologize for these errors and appreciate your careful and detail review of our manuscript. We have thoroughly revised the manuscript to correct these issues. Specifically, we have rephrased problematic expressions such as "not beg problem" and "were significantly contribute" to standard and grammatically correct forms. Additionally, we have carefully proofread the entire manuscript. Kindly see amendment on page number 8-line number 157-163 and page number 16-line number line number 296-299.

Comment 6. Tables are overly dense (Table 4 in particular). Consider splitting key results into separate tables or adding a forest-plot figure for the main AORs. Once these issues are adequately addressed, the manuscript will be much stronger and ready for reevaluation.

Response: Dear Reviewer, thank you for your helpful suggestion regarding the presentation of results in Table 4. We the author, agree that the original table was overly dense and may have hindered readability. There for we splitting key results into separate tables. Dear reviewer, please kindly see amendment on the page number 15-16-line number 285-289.

Reviewer #2:

The present manuscript addresses a salient and contemporary public health concern barrier to accessing healthcare services among women of reproductive age in sub-Saharan Africa utilizing nationally representative DHS datasets and multilevel modeling. The study's objective is praiseworthy and has the potential to contribute to the development of policy. However, in its current state, the manuscript necessitates substantial revision (major) before it can be considered for publication. The following section delineates the issues that should be addressed. **Comment 1.** The document under review contains a multitude of grammatical inaccuracies, cumbersome phrasing, and a lack of consistent terminology. These issues often hinder the reader's ability to comprehend the original message. It is imperative that all documents undergo a thorough revision.

Response: Dear reviewer, Thank you for your valuable feedback. We acknowledge the concern raised about grammatical, inaccuracies and inconsistent terminology. To address this issue, we have performed entire manuscript revision. clarity, coherence, and consistency have been improved. The revised version has been thoroughly edited for grammar and style, and we have ensured uniform use of terminology throughout. We believe these changes have significantly enhanced the readability and overall quality of the document.

Comment 2. It is imperative that all statistical estimates undergo a thorough review to ensure internal consistency and coherence.

Response: Dear reviewer, Thank you for your valuable comment. We have carefully reviewed all statistical estimates presented in the manuscript to ensure internal consistency and coherence. Cross-checks were performed between the results reported in the tables, figures, and text to confirm accuracy and alignment and manuscript has been updated accordingly.

comment 3. The selection of these specific countries is not accompanied by a clear justification, nor is there an explanation for why other countries were excluded (DHS data from 10 sub-Saharan African countries). A rationale must be provided for the inclusion criteria, and the findings must be explicated in terms of their generalizability to the entire SSA region.

Response: Dear reviewer, thanks very much and appreciates your valuable comment. The selection of countries was based primarily on the availability of recent, comprehensive, and comparable Demographic and Health Survey (DHS) data relevant to the study variables. While all sub-Saharan African countries with DHS data were initially considered, the focus was narrowed to 10 countries that met specific criteria namely, data completeness and survey recency. Only countries with fully available DHS data collected between 2019 and 2023 were included. The study draws on data from 134,470 women of reproductive age across diverse regions, enhancing the generalizability and contextual relevance of the findings within sub-Saharan Africa.

Comment 4. The definition of the binary outcome "barrier to accessing health care" is not sufficiently explained. The coding scheme and threshold for categorization are not clearly delineated. It is imperative that further elucidation be provided.

Response: Dear reviewer, thank you for your valuable comment, we acknowledge that the definition and coding of the binary outcome variable “barrier to accessing health care” were not sufficiently detailed in the original manuscript. We have now revised the Methods section to provide a clearer explanation. Specifically, the binary outcome was constructed based on responses to the following DHS questions: "Getting permission to go to the doctor?" "Getting money needed for treatment? "Distance to health facility?" "Not wanting to go alone?" Each of these questions had response options such as a big problem or not a problem. In our analysis, women who reported at least one big problem were coded as having a barrier to healthcare access (1), and those who reported none of the four as were coded as having no barrier (**0).** This approach is consistent with prior literature using DHS data. The coding scheme and threshold for categorization have now been explicitly stated in the manuscript to improve clarity and reproducibility. Dear reviewer, leas kindly see amendment on method section line number 149-161.

Comment 5. The multilevel mixed-effects logistic regression model is a methodologically sound approach; however, the following issues must be addressed: The presence of inconsistent or implausible AORs and confidence intervals is a concern. The section comparing models is not sufficiently clear. An analysis of the data reveals an overlap and redundancy between text, tables, and figures. It is imperative that all model outputs are thoroughly reviewed and that any transcription errors are rectified. The explanation of random effects statistics must be improved. It is recommended that Figures 1 and 2 be removed or merged into a more informative graphical representation.

Response: Dear reviewer, thank you for your constructive comments. We acknowledge the issue and have conducted a thorough revision all AORs and their corresponding confidence intervals. Additionally, we re-ran the regression to confirm the accuracy and consistency of all estimates reported in the manuscript. We have revised the section comparing the multilevel models to enhance clarity. Specifically, we now clearly describe the sequence of model building (null model, random intercept model, full model), the criteria used for model selection (AIC, log-likelihood, and deviance), and the rationale for choosing the final model. A table summarizing model fit statistics has been added to support this comparison. The explanation of random effects has been expanded. We now clearly describe the intraclass correlation coefficient (ICC), variance components, and their interpretation in the context of the multilevel model. This includes a brief

explanation of the importance of accounting for cluster-level variability and the added value of the multilevel approach. We believe these revisions address your concerns and improve the overall clarity, accuracy, and presentation quality of the manuscript. All relevant changes have been tracked in the revised document for ease of review.

---

comment 6. The discourse is predominantly descriptive, exhibiting a paucity of depth and analytical rigor. It is imperative that the discussion be expanded to encompass the mechanisms underpinning the observed associations, as well as their policy ramifications. It is imperative to differentiate distinctly between individual, household, and system-level barriers.

Response: Dear reviewer, thank you for this important concern. As per your recommendation, we improve analytical depth of the discussion and tries to give conceptual clarity. The author revised the Discussion section to move beyond a descriptive summary of findings. Detailed exploration of the possible mechanisms of associations. In addition, we highlight the need for integrated interventions that address both demand- and supply-side barriers and we recommend targeted strategies, such as strengthening community-based health education, improving transportation infrastructure, and expanding health insurance coverage to reduce inequities in access. Dear reviewer please kindly see the amendment in discussion section on page 17-18-line number 303-374.

Comment: 7. The added value of this study is not articulated with sufficient clarity.

Response: Dear reviewer, thank you for the insightful comment. We have revised the manuscript to more clearly articulate the added value of this study. Kindly see amendment on introduction section on Page 5-line number 118-129.

Comment 8. It is imperative that the keywords, table, and reference formatting be corrected.

Response: Dear reviewer, thank you for your impactful comment. We have thoroughly reviewed and corrected the formatting of the keywords, tables, and references to ensure consistency with the journal's guidelines.

Response to reviewers' comments

PONE-D-24-28999R1

Title: Barriers to accessing health care among women's in Sub-Saharan Africa: Multilevel Analysis of Recent Demographic and Health Survey from 2019-2023.

Journal: PLOS ONE

Authors: Gebreeyesus Abera Zeleke[1]*, Enyew Getaneh Mekonen[1], Bewuketu Terefe[2], Mulugeta Wassie[3], Agazhe Aemro[3], Alebachew Ferede Zegeye[3], Mohammed Seid Ali[4], Berhan Tekeba[4], Tadesse Tarik Tamir[4], Belayneh Shetie Workneh[5]

Subject: Submission of revised manuscript

Yours sincerely,

Gebreeyesus Abera Zeleke (on behalf of all authors)

University of Gondar, Gondar, Ethiopia

Comments from PLOS Editorial

Comment: We note that one or more reviewers has recommended that you cite specific previously published works. As always, we recommend that you please review and evaluate the requested works to determine whether they are relevant and should be cited. It is not a requirement to cite these works. We appreciate your attention to this request.

Response: Dear editor, we sincerely thank you for forwarding the reviewers' suggestions regarding additional references. We have carefully revised the recommended works and evaluated their relevance to our study. Accordingly, we have incorporated those that are directly pertinent and contribute meaningfully to the context of our manuscript.

Reviewers' comments:

Reviewer's Responses to Questions

Response to Editors and Reviewer's Comment

We are sincerely grateful to the editor and reviewers for their thoughtful and constructive feedback on our manuscript. The comments are both encouraging and insightful, and we greatly appreciate the recognition of the clinical importance of our study and its findings. Please find below our detailed responses to the reviewers' comments, along with the revised manuscript (clean version)

and a version with tracked changes, submitted separately. We sincerely appreciate the reviewers' time and effort, and we trust that the revisions have addressed the concerns raised and have further strengthened the quality of the manuscript.

Reviewer #1

Comment 1: The manuscript is significantly improved, and the majority of my initial concerns have been comprehensively addressed. However, one minor point remains. In the previous review, I suggested a citation that the authors acknowledged as valuable, but it has not yet been incorporated into the manuscript's body or reference list.

To potentially strengthen the discussion on limitations and future directions, the authors might consider the following point. The current analysis is comprehensive but relies entirely on self-reported data, which can be subject to recall bias. A forward-looking addition to the discussion could acknowledge this and suggest how future studies might overcome this limitation. For example, research could incorporate objective measures from technologies like wearable devices to assess certain behaviors or states. The study by Zhao et al. (2025) could be cited as an excellent example here, as it demonstrates a complete workflow for processing such complex physiological signals. Ultimately, this is just a suggestion for the authors' consideration, and the decision to incorporate this perspective rests with them

Response: Dear reviewer, we sincerely thank you for constructive follow-up comment and valuable suggestion. We regret the oversight in not incorporating the recommended citation in our previous revision. We have now addressed the possible drawbacks of depending only on self-reported data in the updated manuscript, emphasizing the possibility of recall bias. We have also recognized that objective measurements, including data from wearable technology, could be incorporated into future studies to supplement self-reported evaluations. The recommended citation to Zhao et al. (2025), which offers applying physiological signal processing in this situation, has also been included to support this section. Please kindly see the amendment on limitation and recommendation of manuscript on page 22 and 23.

Reviewer #3: Major revisions are necessary, particularly with respect to the clarity of the statistical analyses and the presentation of the data.

Comment 1: Line 197: In the health survey data, if a household-level component exists in addition to the individual and community levels, it should usually be considered in the analysis. Are most households contribute to single observations as well as the household variance is non-negligible, and random effects are weak/unidentified? The reason(s) should be explicitly stated.

Response: Dear reviewer, we appreciate your thoughtful comment on our analysis's inclusion of the household-level component. We concur that in order to get reliable estimates and prevent bias from unmodeled clustering effects, it is essential to consider the hierarchical structure of the data. The household level was not included in the final model since most household contributed one individual and the variance at the household level was insignificant in the preliminary models.

Comment 2: Line 196 -198: The variables included in Model I, Model II, and Model III are to be stated. Alternatively, the table footnotes should indicate the variables that correspond to each model.

Response: Dear Reviewer, we appreciate your careful reviewing to the clarity of our model presentation, particularly regarding the variables included in Models I, II, and III. we have revised the manuscript to variables included in each model in the text. Additionally, we have updated the footnotes of **table 4** to clearly indicate the variables corresponding to each model for ease of reference. These changes enhance transparency and improve the interpretability of our findings. Please kindly see amendment on page number 9-line number 196-212 and kindly refer **table 4** footnote.

Comment 3: Line 211- 215: The notations 'VC', 'V', 'Variance' should be replaced with the appropriate statistical symbols. Variance null, and the variance model is to be used

Response: Dear Reviewer, we sincerely thank you for this constructive comment. We agree that using standard statistical notation improves clarity. Accordingly, we have replaced all instances of 'VC' 'V' 'Variance' in to formulas that reflect the standard notation. We appreciate the reviewer's suggestion, which has strengthened the Methods section. Please kindly see amendment on page 9- and 10-line number 221-225.

Comment 4: Line 214: The standard formula for MOR and not the shortcut version is to be presented.

Response: Dear Reviewer, thank for pointing this out. We agree that presenting the standard formula for the Median Odds Ratio (MOR) improves clarity and transparency. Accordingly, we have replaced the previous shortcut version with the standard formula. Please see amendment on page 9 and Line number 221- 223.

Comment 5: Line 217- 218: The sentence 'The fixed effects were employed to calculate the correlation between the probability of barriers to obtaining health care access and independent factors at the individual and community levels' is incorrect and requires revision.

Response: Dear reviewer, we appreciate bringing this constructive comment. Since fixed effects in multilevel models estimate the relationship between independent variables and the outcome rather than computing correlations, we agree that the original sentence was incorrect. The sentence has been changed to the associations between independent factors at the individual and community levels and barriers to obtaining health care access were estimated using the fixed effects. Please kindly see the amendment on page 10-line number 227-228.

Comment 6: Line 223 – 225: The sentence is to be written as a method, not mentioning the results in the method section.

Response: Dear reviewer, thank you for pointing this out. We have revised the sentence to describe the methodology without including the results, in accordance with standard reporting practices. Please kindly see amendment on page 10-line number 231-235.

Comment 7: Line 231: The variance figures can be omitted.

Response: Dear reviewer, we appreciate the for your valuable comment. In line with the suggestion, we have omitted the variance figures from the results section. Kindly see the amendment on result section on page 10-line number 241.

Comment 8: Line 232: The symbol percentage for 69.8 is missing

Response: Dear reviewer, thank you for noting this. We have corrected the value to 69.8% at line 232. Kindly see amendment on result section on Page 10-line number 241.

Comment 9: Table 2: The figures presented should be standardized, e.g., consistent use of commas or decimal points. The same applies to Table 3.

Response: Dear reviewer, thank you for this observation. We have standardized the numerical formatting in Tables 2 and 3 to ensure consistency, including the uniform use of decimal and commas. Kindly see the amendment on table 2 on page 10-12-line number 246-248 and table 3 on page 13-line number 270-271.

Comment 10**:** Line 244: Typo 'of.3784967'. The p-value cannot be zero (to use symbol p <)

Response: Dear reviewer, thank you for pointing this out. We have corrected the typo and revised the reporting of the p-value to '$p < 0.001$' to ensure accuracy and consistency with statistical reporting standards. Please kindly see amendment on page 13-line number 254-255.

Comment 11: Line 263-294: It was not clear whether the data written based on Table 4, Table 5, or both. A separate write-up should be provided for each table (Table 4 referring to the association, while Table 5 refers to the determinants of barriers). It is also suggested that the manuscript be proofread thoroughly. Also, please include the crude analyses table prior to adjustment.

Response: Dear reviewer, we sincerely appreciate your constructive comment. We acknowledge that the presentation of the results between Table 4 and Table 5 was not sufficiently clear. To address this, we revised the results section with a distinct write-up for Table 4 and removed Table 5, as it introduced confusion and redundancy. In line with your recommendation, we also added a new table presenting the crude odds ratios (unadjusted analyses) prior to adjustment. The revised version can be found in the updated Tables 4 and 5 (pages 14 and 17–18), with corresponding introductory paragraphs provided in the results section (lines 277–283 and 285–319).

Comment 12: Table 4 & Table 5: The title not clear. Need to mention clearly or denote how the variables in Table 4 & 5 were selected.

Response: Dear Reviewer, we sincerely thank you for this insightful comment. We acknowledge the concern regarding the clarity of the titles for Tables 4 and 5. Upon further review, we recognized that

Table 5 was redundant with Table 4 and, to avoid confusion, we have removed it from the manuscript. We have also revised the title of Table 4 to clearly indicate how the variables were selected and presented.

Comment 13: Line 252-253: The sentence requires revision.

Response: Dear reviewer, thank you for this valuable suggestion. We have revised the sentence for clarity. The corrected version now reads: 'Model comparison using Akaike's Information Criterion (AIC) indicated that Model III achieved the best fit, with the lowest AIC value (239,608.6), substantially lower than the null model (AIC = 1,128,437) and Model II (AIC = 1,127,704). Please kindly see amendment on page 13-line number 262-265.

Comment 14: Line 272: In Table 4, there was no data/stem indicating higher than secondary education.

Response: Dear Reviewer, we sincerely thank for this observation. The apparent absence of higher than secondary education" in Table 4 is due to the fact that this category was used as the reference group in the regression analysis. Consequently, it was not displayed in the table with an odds ratio, as is standard practice. We have now clarified this in the table and in the results section to avoid any confusion. Please Kindly see amendment on table 4 on page 15 and Sociodemographic result table on page 11.

Comment 15: Line 273: No information on Table 5 for AOR=1.21, 95% CI: (1.31, 1.41).

Response: Dear reviewer; We appreciate you detail revision of the manuscript. Upon your direction, we remove the above data that is AOR=1.21, 95% CI: (1.31, 1.41) from Table 5 as well table 4. Because this number was introduced by mistake. This number was not available neither table 5 Nor table 4. Please kindly see the correct version the manuscript on page 14-line number 280-283.

Comment 16: Line 278: Typo 1.07 (based on Table 5).

Response: Dear reviewer, we sincerely thank you for carefully and detail revision. Upon re-examining the manuscript, we found that the value "1.07" was introduced incorrectly and does not appear in either Table 4 or Table 5. We have removed this erroneous value to avoid confusion and have revised the text accordingly. Please kindly see the corrected version on page 14-line number 285-287.

comment 17: Line 281: Typo 1.57 (based on Table 4). In Table 5, there was no data on internet utilization.

Response: Dear reviewer, we thank you for careful and detail reviewing of the manuscript. We re-checked the results and confirmed that the value "1.57" was mistakenly introduced. Internet utilization was not a significant variable and does not appear in Table 5. The incorrect value has now been removed, and the text has been revised to align with the data presented in Table 4.

Comment 18: Line 293: Table 4 & Table 5: East Africa is to be used as reference.

Response: Dear reviewer, we appreciate detail and insightful reviewing. We agree that using East Africa consistently as the reference category improves clarity. In the revised manuscript, we have specified East Africa as the reference group in Table 4. To avoid redundancy and potential confusion, we have removed it from Table 5, since the reference category is already defined in Table 4 and does not need to be repeated. We believe this adjustment enhances the accuracy and readability of the results. Please kindly see corrected version of manuscript on page number 16.

comment 19: The references do not conform to the journal's required format.

Response: Dear reviewer, we sincerely thank for pointing out the references did not conform to the journal's required format. We have carefully revised all references and reformatted them according to the *PLOS ONE* reference style guidelines. We double-checked both the in-text citations and the reference list to ensure full consistency with the journal's requirements. Please kindly see amendment in reference section on page 23-25-line number 459-564.
