# Supplementary material for: Barriers to healthcare access among women in sub-Saharan Africa: A pooled analysis of multi-country DHS data (2019–2023)
Source: PLoS One. 2026 Feb 19;21(2):e0331328. doi: 10.1371/journal.pone.0331328 (PMC12919819; doi:10.1371/journal.pone.0331328)
Supplement: Attachment — Submitted filename: PONE-D-24-28999_R3-AF.pdf [file pone.0331328.s008.pdf]

PLOS One

Barriers to healthcare access among women in sub-Saharan Africa: A pooled analysis of multi-country DHS data (2019-2023).

--Manuscript Draft--

**Manuscript Number:**	PONE-D-24-28999R3
**Article Type:**	Research Article
**Full Title:**	Barriers to healthcare access among women in sub-Saharan Africa: A pooled analysis of multi-country DHS data (2019-2023).
**Short Title:**	Barriers to healthcare access among women in sub-Saharan Africa
**Corresponding Author:**	Gebreeyesus Abera Zeleke, MSc<br>University of Gondar College of Medicine and Health Sciences<br>Gondar, ETHIOPIA
**Keywords:**	Access to Healthcare; Barriers to Healthcare; sub-Saharan Africa; Reproductive Age Women
**Abstract:**	Background: Barriers to accessing healthcare have significantly contributed to the high rates of maternal and child mortality and morbidity in developing regions, particularly in Sub-Saharan Africa. Access to healthcare is influenced by multiple factors, including financial constraints, geographic location, the availability of services, and the quality of care provided. This study aimed to assess the magnitude of these barriers and to identify the factors influencing healthcare access among women of reproductive age in Sub-Saharan Africa.<br>Methods: This study utilized secondary data from the most recent Demographic and Health Surveys conducted between 2019 and 2023 across Sub-Saharan Africa. A weighted sample of 134,470 women of reproductive age was included in the analysis. Data were analyzed using Stata version 14, employing a multilevel mixed-effects logistic regression model. Factors associated with barriers to healthcare access were considered statistically significant at P-values less than 0.05. Adjusted odds ratios (AORs) with 95% confidence intervals (CIs) were used to interpret the results.<br>Results: In this study, the prevalence of barriers to healthcare access among women was found to be 55.84% (95% CI: 55.57, 56.10). Husband educational status (AOR=1.23, 95% CI: 1.15, 1.30), media exposure (AOR=1.17, 95% CI: 1.11, 1.24), antenatal care visit (AOR=1.31, 95% CI: 1.10, 1.55), delivery place (AOR=1.33, 95% CI: 1.24, 1.43), health insurance (AOR=1.18, 95% CI: 1.11, 1.26), wealth index (AOR=2.29, 95% CI: 2.14, 2.45), (AOR=1.57, 95% 95CI: 1.46, 1.68), and visiting a health facility within the past 12 months (AOR=0.91, 95% CI: 0.87, 0.97) were individual-level factors associated with the outcome variable. In addition, place of residence (AOR=1.30, 95% CI:1.22, 1.39), community literacy level (AOR=1.13, 95% CI:1.03, 1.24), community poverty level (AOR=1.12, 95% CI:1.02, 1.22), and region were community-level factors associated with barriers to healthcare access.<br>Conclusion: In this study, over half of women in Sub-Saharan Africa face significant barriers to healthcare access. Educational status, media exposure, place of residence, antenatal care attendance, place of delivery, health insurance coverage, wealth index, recent visits to health facilities, community literacy levels, community wealth status, and regional differences were determinant factors. These findings highlight the need for Sub-Saharan African governments to prioritize these determinants when formulating policies and strategies aimed at reducing preventable barriers to healthcare access in the region.
**Order of Authors:**	gebreeyesus abera zeleke, MSc
	Enyew Getaneh Mekonen
	Bewuketu Terefe
	Mulugata Wessie
	Agazhe Aemro
	Alebachew Ferede Zegeye

Powered by Editorial Manager® and ProduXion Manager® from Aries Systems Corporation

	Mohammed Seid Ali
	Berhan Tekeba
	Tadesse Tarik Tamir
	Belayneh Shetie Workneh, MSc
**Opposed Reviewers:**	
**Response to Reviewers:**	very good reviewers
**Additional Information:**	
**Question**	**Response**
**Financial Disclosure**<br><br>Enter a financial disclosure statement that describes the sources of funding for the work included in this submission. Review the submission guidelines for detailed requirements. View published research articles from *PLOS ONE* for specific examples.<br><br>This statement is required for submission and **will appear in the published article** if the submission is accepted. Please make sure it is accurate.<br><br>**Funded studies**<br>Enter a statement with the following details:<br>• Initials of the authors who received each award<br>• Grant numbers awarded to each author<br>• The full name of each funder<br>• URL of each funder website<br>• Did the sponsors or funders play any role in the study design, data collection and analysis, decision to publish, or preparation of the manuscript?<br><br>Did you receive funding for this work?	The author(s) received no specific funding for this work.
**Competing Interests**<br><br>Use the instructions below to enter a competing interest statement for this submission. On behalf of all authors, disclose any competing interests that could be perceived to bias this work—acknowledging all financial support and any other relevant financial or non-financial competing interests.	The authors have declared that no competing interests exist.

This statement is required for submission and **will appear in the published article** if the submission is accepted. Please make sure it is accurate and that any funding sources listed in your Funding Information later in the submission form are also declared in your Financial Disclosure statement.<br><br>View published research articles from *PLOS ONE* for specific examples.<br><br>**NO authors have competing interests**<br><br>Enter: *The authors have declared that no competing interests exist.*<br><br>**Authors with competing interests**<br><br>Enter competing interest details beginning with this statement:<br><br>*I have read the journal's policy and the authors of this manuscript have the following competing interests: [insert competing interests here]*<br><br>* typeset	
**Ethics Statement**<br><br>Enter an ethics statement for this submission. This statement is required if the study involved:<br><br>• Human participants<br>• Human specimens or tissue<br>• Vertebrate animals or cephalopods<br>• Vertebrate embryos or tissues<br>• Field research<br><br>Write "N/A" if the submission does not require an ethics statement.<br><br>General guidance is provided below. Consult the submission guidelines for detailed instructions. **Make sure that all**	N/A

Powered by Editorial Manager® and ProduXion Manager® from Aries Systems Corporation

information entered here is included in the Methods section of the manuscript.<br><br>**Format for specific study types**<br><br>**Human Subject Research (involving human participants and/or tissue)**<br>• Give the name of the institutional review board or ethics committee that approved the study<br>• Include the approval number and/or a statement indicating approval of this research<br>• Indicate the form of consent obtained (written/oral) or the reason that consent was not obtained (e.g. the data were analyzed anonymously)<br><br>**Animal Research (involving vertebrate animals, embryos or tissues)**<br>• Provide the name of the Institutional Animal Care and Use Committee (IACUC) or other relevant ethics board that reviewed the study protocol, and indicate whether they approved this research or granted a formal waiver of ethical approval<br>• Include an approval number if one was obtained<br>• If the study involved *non-human primates*, add *additional details* about animal welfare and steps taken to ameliorate suffering<br>• If anesthesia, euthanasia, or any kind of animal sacrifice is part of the study, include briefly which substances and/or methods were applied<br><br>**Field Research**<br><br>Include the following details if this study involves the collection of plant, animal, or other materials from a natural setting:<br>• Field permit number<br>• Name of the institution or relevant body that granted permission	
**Data Availability**<br><br>Authors are required to make all data underlying the findings described fully available, without restriction, and from the time of publication. PLOS allows rare exceptions to address legal and ethical	Yes - all data are fully available without restriction

concerns. See the PLOS Data Policy and FAQ for detailed information.<br><br>A Data Availability Statement describing where the data can be found is required at submission. Your answers to this question constitute the Data Availability Statement and **will be published in the article**, if accepted.<br><br>**Important:** Stating 'data available on request from the author' is not sufficient. If your data are only available upon request, select 'No' for the first question and explain your exceptional situation in the text box.<br><br>Do the authors confirm that all data underlying the findings described in their manuscript are fully available without restriction?	
**Describe where the data may be found in full sentences. If you are copying our sample text, replace any instances of XXX with the appropriate details.**<br><br>• If the data are **held or will be held in a public repository**, include URLs, accession numbers or DOIs. If this information will only be available after acceptance, indicate this by ticking the box below. For example: *All XXX files are available from the XXX database (accession number(s) XXX, XXX.*).<br>• If the data are all contained **within the manuscript and/or Supporting Information files**, enter the following: *All relevant data are within the manuscript and its Supporting Information files.*<br>• If neither of these applies but you are able to provide **details of access elsewhere**, with or without limitations, please do so. For example:<br><br>*Data cannot be shared publicly because of [XXX]. Data are available from the XXX Institutional Data Access / Ethics Committee (contact via XXX) for researchers who meet the criteria for access to confidential data.*	All relevant data are within the manuscript and its supporting information files.

The data underlying the results presented in the study are available from (include the name of the third party and contact information or URL).<br>• This text is appropriate if the data are owned by a third party and authors do not have permission to share the data.<br><br>* typeset	
Additional data availability information:	Tick here if your circumstances are not covered by the questions above and you need the journal’s help to make your data available.

Powered by Editorial Manager® and ProduXion Manager® from Aries Systems Corporation

Cover letter

July 5 /7/2024

Gondar, Ethiopia

Subject: Manuscript Submission for publication

Dear Sir/Madam

Our manuscript entitled ‘Barriers to accessing health care among women’s in Sub-Saharan Africa: Multilevel Analysis of Recent Demographic and Health Survey from 2019-2023’ was aimed to determine Barriers to accessing health care and determinant factors among women’s. The participants were reproductive age women in sub Saharan Africa. The results of this study will assist African health care providers and policy makers in better understanding of barriers to accessing health care in reproductive age so that they can tailor interventions to the women’s needs. Additionally, the results of this study will be used as a baseline for program and policy planners to implement the idea of evaluating barrier to accessing health care and risk factors in African health system. It can also help researchers conduct similar studies with wider scopes. In this view, we found your esteemed journal to be a better platform to disseminate our finding to scientific community. With this, we kindly request you to consider our contribution for publication in your journal. Given this, the word count of this manuscript was more than the word limit of the journal, because the objective of this study was not answered by such limed word.

Please address all correspondence concerning this manuscript to the corresponding author Gebreeyesus abera Zeleke at gebreeyesusabe143@gmail.com

With kind regards,
Gebreeyesus abera Zeleke

Manuscript

Click here to access/download;Manuscript;Revision 2 Barrier Manuscript.docx

Barriers to healthcare access among women in sub-Saharan Africa: A pooled analysis of multi-country DHS data (2019-2023).

Gebreeyesus Abera Zeleke[1]*, Enyew Getaneh Mekonen[1], Bewuketu Terefe[2], Mulugeta Wassie[3], Agazhe Aemro[3], Alebachew Ferede Zegeye[3], Mohammed Seid Ali[4], Berhan Tekeba[4], Tadesse Tarik Tamir[4], Belayneh Shetie Workneh[5]

[1] Department of Surgical Nursing, School of Nursing, College of Medicine and Health Sciences, University of Gondar, Gondar, Ethiopia

[2] Department of Community Health Nursing, School of Nursing, College of Medicine and Health Sciences, University of Gondar, Gondar, Ethiopia.

[3] Department of Medical Nursing, School of Nursing, College of Medicine and Health Sciences, University of Gondar, Gondar, Ethiopia;

[4] Department of Pediatrics and Child Health Nursing, School of Nursing, College of Medicine and Health Sciences, University of Gondar, Gondar, Ethiopia.

5 Department of Emergency and Critical Care Nursing, School of Nursing, College of Medicine and Health Sciences, University of Gondar, Gondar, Ethiopia

Correspondence Author*

Gebreeyesus Abera Zeleke

Department of Surgical Nursing, School of Nursing, College of Medicine and Health Science, University of Gondar, Gondar, Ethiopia.

Email address: gebreeyesusabe143@gmail.com

Co-authors:

Enyew Getaneh Mekonen **Email Address:** enyewgetaneh12@gmail.com

Bewuketu Terefe: **Email address**: woldeabwomariam@gmail.com

Mulugeta Wassie: **Email address:** mulugeta2113@gmail.com

Agazhe Aemro: **Email address:** agazhe1049@gmail.com

Alebachew Ferede Zegeye: **Email address:** alexferede24@gmail.com

Mohammed Seid Ali: **Email address:** muheseid2592@gmail.com

Berhan Tekeba: **Email address:** berishboss7@gmail.com

Tadesse Tarik Tamir: **Email address:** tadestar140@gmail.com

Belayneh Shetie Workneh: **Email address:** belay2319@gmail.com

Abstracts

Background: Barriers to accessing healthcare have significantly contributed to the high rates of maternal and child mortality and morbidity in developing regions, particularly in Sub-Saharan Africa. Access to healthcare is influenced by multiple factors, including financial constraints, geographic location, the availability of services, and the quality of care provided. This study aimed to assess the magnitude of these barriers and to identify the factors influencing healthcare access among women of reproductive age in Sub-Saharan Africa.

Methods: This study utilized secondary data from the most recent Demographic and Health Surveys conducted between 2019 and 2023 across Sub-Saharan Africa. A weighted sample of 134,470 women of reproductive age was included in the analysis. Data were analyzed using Stata version 14, employing a multilevel mixed-effects logistic regression model. Factors associated with barriers to healthcare access were considered statistically significant at P-values less than 0.05. Adjusted odds ratios (AORs) with 95% confidence intervals (CIs) were used to interpret the results.

Results: In this study, the prevalence of barriers to healthcare access among women found to be 55.84% (95% CI:55.57, 56.10). Husband educational status (AOR=1.23, 95% CI: 1.15, 1.30), media exposure (AOR=1.17, 95% CI: 1.11, 1.24), antenatal care visit (AOR=1.31,95% CI: 1.10, 1.55), delivery place (AOR=1.33, 95% CI:1.24, 1.43), health insurance (AOR=1.18, 95% CI: 1.11, 1.26), wealth index(AOR=2.29, 95% CI 2.14, 2.45), (AOR=1.57, 95CI: 1.46, 1.68) and visiting health facility within the past 12 months (AOR=0.91,95% CI:0.87, 0.97) were individual level factors associated with outcome variable. In addition, place of residence (AOR=1.30, 95% CI:1.22, 1.39), community literacy level (AOR=1.13, 95% CI:1.03, 1.24), community poverty level (AOR=1.12, 95% CI:1.02, 1.22), and Region were community level factors associated with barriers to healthcare access.

Conclusion: In this study, over half of women in Sub-Saharan Africa face significant barriers to healthcare access. Educational status, media exposure, place of residence, antenatal care attendance, place of delivery, health insurance coverage, wealth index, recent visits to health facilities, community literacy levels, community wealth status, and regional differences were determinant factors. These findings highlight the need for Sub-Saharan African governments to prioritize these determinants when formulating policies and strategies aimed at reducing preventable barriers to healthcare access in the region.

Key words: Access to Healthcare; Barriers to Healthcare; Sub-Saharan Africa; Reproductive Age Women

Introduction

Access to healthcare is broadly defined as the ability to prevent illness, disability, and premature death, to promote and maintain health, and to attain health equity, particularly for women of reproductive age, by offering inclusive, high-quality care and the best possible availability, affordability, and accessibility of medical services[1-3]. Reproductive-age women's health is a critical global public health concern since it influences population health outcomes and fosters socioeconomic growth[4]. The advancement of socioeconomic development is significantly influenced by the health of women of reproductive age, especially in Africa. [5]. The Sustainable Development Goals (SDGs) place a strong emphasis on women's health, with Targets 3.7 and 3.8 of SDG 3 specifically focusing on achieving universal health coverage by 2030 and ensuring access to sexual and reproductive health services, including education and information on family planning[6, 7]. The Sustainable Development Goals (SDGs) explicitly target a substantial reduction in maternal mortality, with Goal 3.1 aiming to lower the global maternal mortality ratio to fewer than 70 per 100,000 live births by 2030. While these global efforts have contributed to some improvements in women's health outcomes, they have yet to fully address the root causes of maternal mortality. Achieving sustained and comprehensive progress remains a critical challenge. In 2016, Sub-Saharan Africa accounted for a disproportionately high share of the 303,000 maternal deaths reported globally, the majority of which were due to preventable causes. Moreover, non-communicable diseases continue to contribute significantly to female morbidity and mortality across nearly all countries, with a particularly severe impact in low- and middle-income settings, where many women are left with long-term disabilities [4-6]. Access to healthcare involves multiple dimensions, including financial affordability, geographic accessibility, availability of medical services, and the quality of care provided in health facilities. [8]. Access to healthcare is a key determinant of whether individuals utilize health services. Globally, approximately 400 million people lack essential healthcare, and around eight million die each year from preventable diseases—resulting in an estimated economic loss of $6 trillion in low- and middle-income countries. [6, 9, 10]. In 2015, world leaders pledged to achieve universal health coverage as part of the Sustainable Development Goals (SDGs), aiming to ensure access to affordable, high-quality medicines and financial protection for all [11, 12]. In 2017, the World Bank and WHO reported that half of the global population lacks access to essential health services. As a result, many countries in Sub-Saharan Africa have made universal healthcare a key part of their national health

strategies. However, there has been slow progress in turning this commitment into tangible results, particularly in securing national funding for equitable, high-quality healthcare, financial security, and overall health [6, 9, 13, 14]. Reproductive age women and child mortality from low family planning uptake, unsafe abortion practices, unintended pregnancies, and an increase in home births are all implications of women in reproductive age having trouble accessing health care[14]. Previous research has offered some evidence regarding the sociodemographic and economic factors that can be barriers to accessing healthcare, such as marital status, place of residence, maternal age, wealth index, educational attainment, and unemployment status. Nevertheless, women of reproductive age continue to face significant disparities in healthcare access and Post-COVID-19 evidence on healthcare access remains limited, particularly in low-resource settings; this study addresses gap by leveraging recent DHS data to examine regional disparities, intersectional demographics, and implications for health system recovery and healthcare access[10, 14-16]. This study makes three unique contributions to the body of existing literature. In order to provide a current and policy-relevant analysis of the barrier to healthcare access, it first makes use of the most recent 2019–2023Demographic and Health Survey (DHS) data. Second, our analysis takes a harmonized multi-country approach across ten sub-Saharan African nations, improving cross-national comparability and generalizability in contrast to many previous studies that are limited to single-country analyses or rely on older datasets. In order to gain a better understanding of the structural and contextual factors that influence healthcare access, the study employs a comprehensive multilevel analysis of determinants at the individual and community levels. These contributions put our research in a position to guide focused, empirically supported programs provide fair access to healthcare for reproductive age women in sub-Saharan. Therefore, this study aimed to ascertain the magnitude and potential contributing factors associated with barriers to healthcare access among reproductive age women in sub-Saharan Africa.

Methods

Study design, study area, and period.

Data from ten Sub-Saharan African countries, collected between 2019 and 2023, were used to perform a multilevel mixed-effects analysis, based on the most recent Demographic and Health

Surveys (DHS). The DHS collects community-based, cross-sectional data approximately every five years to generate updated health and health-related indicators.

Patient and public involvement statement

This study was used, Secondary data from the Demographic and Health Surveys data.

Data source, study population and sampling technique

A secondary data analysis was conducted using Demographic and Health Survey (DHS) data collected between 2019 and 2023 from selected sub-Saharan African countries. The study specifically included DHS datasets from ten countries: Burkina Faso, Gabon, Ghana, Kenya, Liberia, Rwanda, Sierra Leone, Tanzania, The Gambia, and Mozambique, each of which had recent and publicly available survey data within the specified timeframe. The datasets were appended to assess the extent of, and factors underlying, barriers to healthcare access among women of reproductive age in sub-Saharan Africa. Each country's DHS comprises multiple datasets. The DHS employs a stratified, two-stage cluster sampling design: in the first stage, enumeration areas are selected, followed by systematic sampling of households within each selected area in the second stage.

The variable barrier to healthcare access was generated by computation of (v467b, v467c, v467d, v467f) from maternal recode (IR) data [illegible] was recoded. A binary logistic regression model was used to identify determinants factor associated with barriers healthcare access. Determinants barriers to healthcare access were reported in terms of an AOR with a significance level of (95%) confidence interval. In the univariate analysis, at 95% confidence intervals with a p-value of < 0.25 was considered for the candidate multivariable analysis of data **(S1 Table 5).** All variables with p values of <0.05 were considered statistically significant. Since DHS data are nationally representative, stratified multistage cluster sampling is required; therefore, sampling weights must be applied to produce unbiased estimates. To correctly weigh DHS data, we used the sampling weights provided in the dataset, specifically the individual weight **(**v005**)** for women's individual recode files. This weight must be divided by 1,000,000 (v005/1000000). using "gen wgt=v005/1000000 Stata commands. A total weighted sample of 134,470 reproductive age women were included in the study **(Table 1).**

Table 1: The weighted Sample size for magnitude and determinants of barriers to accessing health care among reproductive age women in sub-Saharan Africa**.**

Country	Year of survey	weighted sample (n)	weighted sample (%)
Burkina Faso	2021	17659	13.13
Gabon	2019-2021	6507.15	4.83
Ghana	2022	15014	11.16
Kenya	2022	16716.36	12.43
Liberia	2019-2020	8065	5.99
Rwanda	2019-2020	14634	10.88
Sierra Leone	2019	15574	11.58
Tanzania	2022	15254	11.34
Gambia	2019-2020	11865	8.08
Mozambique	2023	13181.93	9.80
TOTAL		134470	100%

Study variables

Outcome variables: The outcome variable of this study was barriers to healthcare access. Based on DHS guidelines, outcome variable was constructed as a composite measure based on four indicators: (1) distance to a health facility, (2) difficulty in obtaining money for treatment, (3) difficulty in obtaining permission to visit a health facility, and (4) reluctance to go to a health facility alone. Women who respond experiencing at least one of these four challenges were classified as facing significant barriers to healthcare access and were coded as 1 and leveled as "big problem"**.** Conversely, women who respond no difficulty in any of the four indicators were categorized as having no significant barriers to healthcare access and were coded as 0 and leveled as "no big problem"[17]. Handling missing ~~value~~ on outcome variables were retained in the denominator accordance with DHS analytical guidelines.

Individual level Variables: ~~Hence~~ DHS data are hierarchical, independent factors come from two sources: ~~that was at~~ individual and community levels factors were considered for this analysis. The individual-level independent variables were women's age (15-19, 20-35, 36-49), Women educational status (no education, Primary education , Secondary & Higher education), women occupation (working, not working), Religion (Muslim, Christian , Others), Wealth index (Poor, Middle, Rich) media exposure (~~yes~~, No), internet utilization (yes, No), Residency (urban, rural), Husband educational status (No education, Primary education, Secondary& Higher

education), Husband occupational status (No working, Working), using Contraception (No, Yes) Visiting health facility in past 12 months (No, Yes), place of delivery (Home, Institutional delivery), ANC visit (No antenatal visit, 1-4 visit, > 4 visit).

The community-level Variables: Poverty level (Low, High), Literacy level (Low, High), Media exposure, (Low, High), Residency (Urban, Rural), Region (East, West), Community ANC visit

Data processing and statistical analysis

Stata version 14 statistical software was used to clean, record, and analyze secondary DHS data sets. The variables in the DHS data are grouped into clusters, and the variables within a cluster are more similar to each other than those in other clusters. The presumptions of equal variance across clusters and independent data were violated in order to run a typical logistic regression model. This suggests that between-cluster factors must be considered using a complex model.

Given this, multilevel mixed-effects logistic regression model was used to determine the factors that associated with ~~barrier~~ to accessing the health care. Multilevel mixed effect logistic regression follows four models. The null model (only outcome variable), mode I (individual level variable such as age, Religious status**,** educational status, Husband educational, marital occupation**,** Husband occupation**,** marital status**,** Contraceptive Utilization, place of delivery, ANC visit, media exposure, Intermate utilization**,** visiting health facility within 12 months, health insurance , wealth index) model II community level variables, Region in sub-Saharan Africa**,** community poverty level, community media exposure, Community distance health facility, Community literacy level, Community ANC visit), and model III (both individual and community level variables such as age, Religious status**,** educational status, Husband educational, marital occupation**,** Husband Occupation**,** marital status**,** Contraceptive Utilization, place pf delivery, ANC visit, media exposure, Intermate utilization**,** visiting health facility within 12 months, health insurance , wealth index), model II (community level variables, Region in sub-Saharan Africa**,** community poverty level, community media exposure, Community distance health facility, Community literacy level, Community ANC visit). The null model was used to check the variability of outcome variable across the cluster. The relationship of individual-level variables with outcome variable (Model I) and the association of community-level variables with outcome variable (Model II) were

determined. In the final model, the association of both individual and community-level variables was fitted with the outcome variable.

Random effects

The intra-class correlation coefficient (ICC), proportionate change in variance (PCV), and median odds ratio (MOR) were used to quantify random effects or measures of variation of the outcome variables.

To assess the variation between clusters, the proportionate change in variance (PCV) and intra-cluster correlation coefficient (ICC) were calculated as By treating the cluster as a random variable, the ICC shows that the variation in the health care access barriers between clusters may be computed as follow $ICC = \frac{\sigma^2 uo}{\sigma^2 uo + \pi^2/3} \times 100\%$. The MOR, which is determined by using clusters as a random variable and calculating the median value of the odds ratio between the highest-risk and lowest-risk areas for barriers to healthcare access was calculated as: $MOR = \exp(0.6745\ \sqrt{(2 * \sigma^2 uo})$) Furthermore, the PCV calculated as: variable that explains the variation in the degree of barriers to healthcare access $PCV = \frac{\sigma^2 uo - \sigma^2}{\sigma^2 uo} \times 100\%$;. where VC is the cluster level variance and Vnull is the variance of the null model.

The associations between independent factors at the individual and community levels and barriers to accessing health care were estimated using the fixed effects. It was assessed and presented using adjusted odds ratio (AOR) and 95% confidence intervals with a p-value of < 0.05. Because of the nested nature of the model, deviation = -2(log likelihood ratio) was used to compare models, and the model with the lowest deviance was selected as the best-fitted model. By evaluating the variance inflation factors, the model assumption for multi-collinearity was confirmed. In addition, Model comparison was performed using Akaike's Information Criterion (AIC). The model includes both random intercepts and random slopes (Model III) was evaluated alongside alternative models, with AIC values calculated to assess relative model fit and parsimony.

Ethical approval and consent to participate

This study was based on analysis of existing survey datasets in the public domain (SSA) that are freely available online with all the identifier information anonymized, no ethical approval was required.

Results

In this study a total of 134,470 reproductive age women were included from 10 Sub-Saharan African countries. The Mean age of participants was 28.99 years with SD. 9.68. More than two thirds (69.8%) of women did not use contraception, and over 72.22% reproductive age women did not use health insurance, and over half (54.22%) of women were living in rural areas in sub-Saharan Africa **(Table 2)**

Table 2: Sociodemographic and economic characteristics of reproductive age women who had barriers to healthcare access in sub-Saharan Africa.

Individual and Community level variables	Frequency(n)	Percentage (%)
**Individual level variables**		
**Maternal age**		
15-19	27890.4	20.7
20-35	69441.4	51.64
36-49	37138.6	27.64
**Religious Status**		
Muslim	48834.95	41.96
Christian	46856.90	39.30
Other	23524.59	19.73
**Educational Status**		
No formal education	35051.70	26.06
Primary education	39651.17	29.48
Secondary and higher education	59767.57	44.44
**Husband's Education**		
No formal education	29414.44	36.72
Primary education	21245.78	26.52
Secondary and Higher education	29428.78	36.74
**Maternal Occupational**		
Not working	50732.90	37.81
Working	83417.01	62.19
**Husband Occupation**		
Not working	10390.43	13.00
Working	69698.11	87.00
**Marital Status**		
Single	42213.40	31.4
Married	58262.00	43.3
Other	33995.4	25.3
**Contraceptive Utilization**		

No	93855.00	69.8
Yes	40615.5	30.2
**Place of Delivery**		
Home	8279.46	15.5
Institution	44979.28	84.5
**ANC visit**		
No visiting	2012.84	3.77
1-4 visiting	24459.34	45.92
>4 visiting	26786.55	50.29
**Media exposure**		
No	34628.45	25.75
Yes	99831.57	74.24
**Internet utilization**		
No	93020.34	69.17
Yes	41450.10	30.82
**Visited Health Facility Last 12 Months**		
No	57137.73	42.49
Yes	77332.71	57.50
**Health Insurance**		
No	85044.69	72.22
Yes	32709.39	27.77
**Wealth Index**		
Poor	47227.18	35.12
Middle	25771.45	19.16
Rich	61471.81	45.74
**Community level variables**		
**Region in sub-Saharan Africa**		
East Africa	59786.29	44.46
West Africa	74684.16	55.54
**Residency**		
Urban	61558.03	45.78
Rural	72912.41	54.22
**Community poverty level**		
Low	70776.71	52.63
High	63693.73	47.47
**Community media exposure**		
Low	61642.30	45.84
High	72828.14	54.15
**Community distance health facility**		

Low	65962	49.00
High	68508	51.00
**Community literacy level**		
Low	57615.01	42.84
High	76855.43	57.15
**Community ANC visit**		
Low	77912.32	57.96
High	56491.92	42.03

In this study, the magnitude of barriers to accessing health care among reproductive age women was found to be 55.84 (95% CI: (55.57, 56.10) owing to one or more of the four factors, the most commonly cited being the difficulty in getting money and the distance from medical facilities (**Figure 1)**

Random effect (Measures of variation) and model fitness

To determine if the data supported the choice to evaluate randomness at the community level, a null model was employed. With the variance of 0.3784967 and a p-value of < 0.001, the null model's results showed that there were notable variations in the barriers to accessing healthcare among clusters. A significant amount of the variance in the outcome between clusters was jointly explained by both individual- and community-level variables, as evidenced by the intra-cluster correlation (ICC), which dropped from 10.31% in the null model to 3.96% in the fully adjusted model (model III). According to the proportionate change in variance (PCV), Model III explains the greatest percentage (64.09%) of the group-level variation in comparison to the null model, group comparison showed that, Model I (50.19%) and Model II (24.56%). This suggests that the model fits better as more predictors are included. Model comparison using Akaike's Information Criterion (AIC) indicated that Model III achieved the best fit, as evidenced by the lowest AIC (239,608.6), markedly lower than both the null model (AIC = 1,128,437) and Model II (AIC = 1,127,704). Model I also showed good fit (AIC = 239,764.8), though slightly less optimal than Model III and highest log-likelihood value (−21,433.284) among all competing models. Multicollinearity using variance inflation factors (VIF) indicated that all VIF values ranged from 1.03-1.98, which is within accepted range **(Table 3)**

Table 3: Model comparison and Fitness random effect analysis for barriers of accessing health care of among reproductive age women in sub-Saharan Africa**.**

Parameter	Null model	model I	model II	model III
**Variance**	0.3784967	0.1885269	0.28553	0.1358817
**ICC**	10.31%	5.41%	7.98%	3.96%
**MOR**	1.79	1.51	1.66	1.41
**PCV**	Reference	50.19%	24.56%	64.09%
**Model Fitness**				
**LLR**	-90175.365	-21798.27	-86841.704	-21433.284
**Deviance**	1803596.73	43596.54	173683.408	42866.568
**AIC**	1128437	239764.8	1127704	239608.6

ICC: intra-cluster correlation**, LLR:** loglikelihood ratio**, MOR:** median odds ratio**, PCV:** proportional change in variance. **AIC:** Akaike information criteria.

Determinants of barriers to healthcare access among reproductive age women in Sub-Saharan Africa

Multivariable multilevel logistic regression analysis of individual and community level factors associated with Barriers to health care access among women in Sub-Saharan Africa.

In the final fitted model of multivariable multilevel logistic regression, Husband educational status, media exposure, place of residency, Antenatal care visit, place of delivery, health insurance utilization, wealth index status, visiting health facility within 12 months, and country category (western, eastern Africa) were significantly associated with barriers of accessing health care among reproductive age women in sub-Saharan Africa. The odds of barriers of accessing health care were 1.30 times higher among reproductive age women living in rural residency compared to women living in an urban area **(**AOR=1.30, 95% CI: 1.22, 1.39). and the odds of barriers healthcare access were 1.23 times more likely to among reproductive age women whose husband had no education compared to women whose husband had secondary and higher education (AOR=1.23, 95% CI: 1.15, 1.30). In addition, odds of barriers healthcare access were 1.33 times more likely to occur in women who delivered at home compared to women who had institutional delivery (AOR=1.33, 95% CI: 1.24, 1.43), and the odds of barriers healthcare access were 1.31 times higher

among women who had no history of antenatal visit compared to women who had more than four ANC visit (AOR=1.31, 95% CI: 1.10, 1.55); The odds of barriers to healthcare access among reproductive age women who had no media exposure were 1.17 times more likely to compared with a woman who had media exposure (AOR=1.17, 95% CI: 1.11, 1.24) and the barriers to healthcare access among reproductive age women who were not visiting a health facility within 12 months were 9% times less likely compared to women who had visiting health care facility within 12 months of study period (AOR=0.91, 95% CI: 0.87, 0.97). The odds of perceived barrier of accessing health care among reproductive age women who had no health insurance utilization were 1.18 times more likely to compared with a woman who had used health insurance (AOR=1.18, 95% CI: 1.11, 1.26). ~~additionally~~, the odds of perceived barriers healthcare access among reproductive age women who were classified under poor and middle wealth status were 2.29 and 1.57 times more likely ~~of barriers to healthcare access~~ compared to reproductive age women who were classified under rich wealth status category (AOR=2.29 95% CI:2.14, 2.45) and (AOR=1.57, 95% CI: 1.46, 1.68). The odds of a perceived barrier of accessing health care service among reproductive age women who live in West Africa region were 2.38 times more likely barriers to healthcare access compared to those living in East Africa region (AOR=2.38, 95% CI: 2.21, 2.55) **(Table 4).**

Table 4: Multivariable multilevel logistic regression of individual and community level factors associated with barriers to healthcare access among reproductive age women in Sub-Saharan Africa.

**Individual and community level factors**	**Model I AOR [95% CI]**	**Model II AOR [95% CI]**	**Model III AOR [95% CI]**
**Individual level factors**			
**Maternal age**			
15-19	0.96 [0.86, 1.07]		0.96 [0.86,1.08]
20-35	1.06 [1.01,1.13]		1.04 [0.99, 1.11]
36-45	1		1
**Residency**			
Urban		1	1
Rural		2.29 [2.24, 2.35]	1.30 [1.22, 1.39] *
**Maternal education**	1.10 [1.02, 1.18]		1.04 [0.97, 1.11]
No education	0.93 [0.87, 0.99]		1.06 [0.99, 1.14]
Primary education	1		1

Secondary and above		
**Husband education**		
No education	1.26 [1.19,1.35]	1.23 [1.15, 1.30] *
Primary education	1.04 [0.97,1.12]	1.23 [0.67, 1.22]
Secondary & higher education	1	1
**Maternal occupation**		
Working	1	1
Not working	0.99 [0.94, 1.04]	1.02 [1.01, 1.11] *
**Husband occupation**		
Working	1	1
Not working	0.90 [0.83, 0.97]	1.00 [0.92, 1.08]
**Religion**		
Muslim	1	1
Christian	1.62 [1.53, 1.71]	0.46 [0.57, 1.94]
Other	1.22 [1.13, 1.31]	1.36 [0.75, 1.46]
**Contraceptive utilization**		
No	1.11 [1.05,1.17]	1.03 [0.98, 1.08]
Yes	1	1
**Place of delivery**		
Home	1.30 [1.21, 1.40]	1.33 [1.24, 1.43] *
Institution	1	**1**
**Antenatal Care Visit**		
No	1.01 [0.85, 1.19]	1.31 [1.10, 1.55] *
1-4 visit	0.94 [0.89, 0.99]	1.13 [0.37, 1.19]
> 4 visits	1	1
**Media exposure**		
No	1.14 [1.08, 1.21]	1.17 [1.11, 1.24] *
Yes	1	1
**Internet utilization**		
No	1.69 [1.58, 1.80]	1.68 [0.67, 1.79]
Yes	1	1
**Visiting health facility last 12 months**		
No	0.92 [0.87, 0.98]	0.91 [0.87, 0.97] *
Yes	1	1
**Health insurance**		
No	1.26 [1.19, 1.34]	1.18 [1.11, 1.26] *
Yes	1	1
**Wealth index**		
Poor	2.80 [2.63, 2.97]	2.29 [2.14, 2.45] *
Middle	1.79 [1.68, 1.92]	1.57 [1.46, 1.68] *
Rich	1	1

**Community level variable**		
**Residency**		
Urban	1	1
Rural	2.29 [2.24, 2.35]	1.30 [1.22, 1.39] *
**Region SSA**		
East Africa	1	**1**
West Africa	2.0 [1.92-2.04]	2.38 [2.21, 2.55] *
**Community ANC visit**		
Low	1.00 [0.93, 1.09]	0.93 [0.86, 1.00]
High	1	1
**Community wealth index**		
Low	0.75 [0.70, 0.81]	1.12 [1.02, 1.22] *
High	1	1
**Community media exposure**		
Low	1.01 [0.92, 1.10]	0.94 [0.86, 1.03]
High	1	1
**Community literacy level**		
Low	1.10 [1.00, 1.20]	1.13 [1.03, 1.24] *
High	1	**1**

Model I: *individual level variable,* **Model II:** *Community level variable,* **Model III**: *Individual and community level variable*

Discussion

Barriers to healthcare access have contributed to maternal and child mortality and morbidity in developing countries such as those in Sub-Saharan Africa. This study aimed to assess the prevalence and determinants of barriers to healthcare access among women in Sub-Saharan Africa. In this study, 55.8 of women experienced barriers to healthcare access. This finding is lower than estimates reported in previous studies from South Africa and Tanzania ~~65%~~ [18, 19] and Ethiopia and sub-Saharan Africa ~~60–72%~~ [1, 17, 20, 21]. This variation could be due to changes in socioeconomic, health infrastructure, health system policies, women's attitudes towards health care seeking behavior, and cultural differences across countries in Sub-Saharan Africa. Moreover, these discrepancies might be due to differences in the study period, because this study includes the most recent DHS data sets, from 2019 to 2023. On the other hand, the findings of this study are similar to but slightly higher than in previous studies Ghana and Gambia ~~(51%), (45.5%)~~ [22, 23]. This variation may be attributed to differences in socioeconomic conditions, health infrastructure, and

health system policies, as well as variations in women's health-seeking attitudes and cultural contexts across countries in sub-Saharan African.

The odds of barriers to healthcare access were 1.30 times higher among reproductive age women living in rural residency compared to women living in an urban area. This finding is supported by previous studies conducted in Ethiopia [1, 20], Guinea[24], Gambia, Tanzania and Ghana [15, 23, 25], respectively and Sub-Saharan countries[22], East Africa[26]. This association may be due to the fact that rural areas have low health infrastructures, i.e., inaccessibility to health facilities, less opportunities and the influences to socio-cultural practices that oblige women to seek permission from their husbands to visit the facility because women's decisions for seeking healthcare services are under their husbands/partners control. Moreover, male involvement and support for women health care access are limited[1, 14, 22, 26]. This implies that governments ~~better to~~ expand infrastructure services in a rural areas and encourage male involvement and support for improving healthcare access for reproductive age women[14].

The odds of perceived barriers to healthcare access were 1.23 and 1.21 times more likely to among reproductive age women whose husbands had no education or primary education compared to women whose husbands had secondary and above educational level, respectively. This finding was consistent with previous studies conducted in east Africa, Ethiopia, Tanzania, Benin, and Bangladesh [1, 15, 20, 26, 27]. The possible explanation could be in a better access to the healthcare among women with educated husbands might be due to better participation and involvement of husbands in their families' health[22]. Women whose partners are educated are also likely to be informed regarding their fundamental human rights and may have higher health literacy. As a result, they are more likely to deal with any form of barrier to healthcare, compared to their counterparts who are less educated and may have lower health literacy [14, 15, 20, 28].

The odds of barriers to healthcare access were 1.33 times more likely to occur among women who delivered at home compared to women who had an institutional delivery. This evidence was supported by a study conducted in Senegal[29]. The plausible justification might be that women who delivered at home may not get health information/counselling from healthcare providers that empower them to use other maternal healthcare services and women who deliver at home may face varous barriers to healthcare access such as financial constraint, lack of transportation, limited autonomy in healthcare decision and sociocultural norms that discourage institutional delivery.

Moreover, they can be affected by the importance of enhancing health system outreach, strengthen community-based education, and addressing sociocultural and socioeconomic disparities that affect health seeking behavior. On the other hand, the odds of barriers of accessing health care among women who had no media exposure were 1.17 times more likely to compared with women who had media exposure. This evidence was supported by studies conducted in Ethiopia[30] and Nepal[31]. This could be the power of mass media in disseminating information concerning maternal health that may enhance women's knowledge and attitude towards health care access and utilization[31]. Barrier of accessing health care among woman who were not visiting health facility within 12 months were 9 % times less likely compared to women who had visiting health care facility within 12 months of study period. The odds of barrier to accessing health care among woman who had no health insurance utilization were 1.18 times more likely to compared with a woman who had used health insurance. This finding was supported with study done in Ethiopia[1], sub-Saharan Africa[22], Tanzania[15], Ghana[25]. The possible reason might be due to the fact that, individuals who were not had health insurance faced for unexpected catastrophic expenditure and difficulties in obtaining money for consultation of the doctor[1]. The odds of barrier to accessing healthcare among woman who were classified under poor and middle wealth status were 2.29 and 1.57 times more likely accessing health care difficulty compared to women who were classified under rich wealth index category**.** This evidenced was supported with study done in Ethiopia, [1, 30, 32, 33] Tanzania[15] Uganda[34], sub Saharan Africa[14]. This finding might be explained by the fact that high out-of-pocket expenses and a lack of accessibility to healthcare facilities pose significant obstacles for middle-class and impoverished women in sub-Saharan Africa. Many people in the region cannot afford healthcare services because over 40% of health costs are paid for directly by individuals[35-38]

The odds of ~~barrier of~~ accessing health care among woman who live ~~west~~ Africa region were 2.38 times more likely ~~barriers to accessing health care~~ compared to east Africa region. According a recent joint report from the World Health Organization and the United Natio~~ns states that~~ high out-of-pocket costs, a lack of adequate healthcare infrastructure, and other sociocultural barriers make it more difficult for women of reproductive age in West African nations such as Nigeria, Mali, and Sierra Leone to access maternal healthcare. On the other hand, East African countries like Rwanda, Kenya, and Ethiopia have strengthened their community health initiatives and gender-inclusive policies, which have improved women's autonomy in accessing healthcare services[39].

Furthermore, our current findings are supported by recent Demographic and Health Survey (DHS) program data (2022–2023), which indicate that women in West African countries are 2-3 times more likely to report delays in accessing healthcare due to cost, shortages of healthcare personnel, and long distances to health facilities. In contrast, East African countries, particularly Uganda and Tanzania, have strengthened their health insurance systems and rural outreach initiatives. Additionally, the World Bank's 2023 Health Service Coverage Index reveals that women in West Africa have significantly lower access to family planning, skilled birth attendance, and other essential reproductive health services[23, 40, 41]

Strengths and limitations

The main strength of this study is the use of nationally representative data, which allows for a comprehensive evaluation of contextual and individual factors associated with barriers to healthcare access. Additionally, the analysis accounted for the clustering within the sample by employing advanced statistical models, enhancing the robustness the findings.

The limitation of this study was many Sub-Saharan African countries have not conducted DHS surveys since 2019, which may affect the representativeness of recent data. In addition, health worker–related factors were not included in the analysis, potentially omitting important segments of population of healthcare access. Furthermore, future research better consider integrating objective measures, such as wearable devices, as demonstrated by Zhao et al. (2025)[42].

Conclusions

In this study, over half of women in Sub-Saharan Africa face barriers to healthcare access, emphasizing the need for focused strategies to improve equitable service delivery. Husband educational status, media exposure, internet utilization, place of residency, ANC visit status, place of delivery, health insurance utilization status, wealth index status, visiting health facility within 12 months, community literacy level and community wealth index and country category (Western, Eastern Africa) were significantly associated with barriers of accessing health care. Therefore, the governments and ministries of health in Sub-Saharan Africa countries ~~better taking in to~~ consideration those factors while designing policies and strategies targeting ~~to~~ health care access and ~~talking~~ the preventable barriers to healthcare access in sub-Saharan Africa.

Declaration

Consent for publication

Not applicable

Data and material availability

Demographic and Health Survey data were used, and are publicly available online at (https://www.dhsprogram.com).

Competing interest

Authors Declared that, there is no competing interest.

Funding

Not applicable

Author’s contributions

GAZ: involved in designing, data extraction, data analysis, interpretation, report and manuscript writing.

EGM: involved in data analysis, interpretation, and, and manuscript writing.

BT: involved in analysis, drafting manuscript and extract data

MW: involved in designing the study, interpretation, report

AA: manuscript writing: involved in data extraction

AA: manuscript writing: involved in data extraction

AFZ: involved in designing and interpretation study

MSA: involved manuscript writing

BT: involved, drafting manuscript and data extraction

TTT: involve analysis and manuscript editing

BSW: involved in analysis, and result interpretation

Acknowledgements

We would like to say the DHS programs for allowing us to use the relevant DHS dataset in this study.

Reference

1. Tamirat, K.S., Z.T. Tessema, and F.B.J.B.H.S.R. Kebede, *Factors associated with the perceived barriers of health care access among reproductive-age women in Ethiopia: a secondary data analysis of 2016 Ethiopian demographic and health survey.* 2020. **20**: p. 1-8.
2. Erasmus, M.O., *The barriers to access for maternal health care amongst pregnant adolescents in the Mitchells plain sub-district.* 2017.
3. Washington, D.L., et al., *Access to care for women veterans: delayed healthcare and unmet need.* 2011. **26**: p. 655-661.
4. Regan, L.J.B.M.A., *Addressing unmet needs in global women's health.* 2018.
5. Izugbara, C., E. Covan, and E. Fugate-Whitlock, *Women's health in Africa: Issues, challenges and opportunities*. 2017: Routledge.
6. Organization, W.H., *Primary health care on the road to universal health coverage: 2019 global monitoring report: Executive summary*. 2019, World Health Organization.
7. Badiuzzaman, M., S.M. Murshed, and M.J.T.J.o.D.S. Rieger, *Improving maternal health care in a post conflict setting: evidence from Chittagong Hill tracts of Bangladesh.* 2020. **56**(2): p. 384-400.
8. Peters, D.H., et al., *Poverty and access to health care in developing countries.* 2008. **1136**(1): p. 161-171.
9. Alkire, B.C., et al., *The economic consequences of mortality amenable to high-quality health care in low-and middle-income countries.* 2018. **37**(6): p. 988-996.
10. Nyakang'o, S.B. and A.J.M. Booth, *Women's perceived barriers to giving birth in health facilities in rural Kenya: A qualitative evidence synthesis.* 2018. **67**: p. 1-11.
11. Colglazier, W.J.S., *Sustainable development agenda: 2030.* 2015. **349**(6252): p. 1048-1050.
12. Cahill, C.M., et al., *Preoperative anemia management program reduces blood transfusion in elective cardiac surgical patients, improving outcomes and decreasing hospital length of stay.* 2021. **61**(9): p. 2629-2636.
13. Peeri, N.C., et al., *The SARS, MERS and novel coronavirus (COVID-19) epidemics, the newest and biggest global health threats: what lessons have we learned?* 2020. **49**(3): p. 717-726.
14. Tessema, Z.T., et al., *Determinants of accessing healthcare in Sub-Saharan Africa: a mixed-effect analysis of recent Demographic and Health Surveys from 36 countries.* 2022. **12**(1): p. e054397.

15. Bintabara, D., K. Nakamura, and K.J.B.o. Seino, *Improving access to healthcare for women in Tanzania by addressing socioeconomic determinants and health insurance: a population-based cross-sectional survey.* 2018. **8**(9): p. e023013.
16. Kea, A.Z., et al., *Exploring barriers to the use of formal maternal health services and priority areas for action in Sidama zone, southern Ethiopia.* 2018. **18**: p. 1-12.
17. Zegeye, B., et al., *Breaking barriers to healthcare access: a multilevel analysis of individual-and community-level factors affecting women's access to healthcare services in Benin.* 2021. **18**(2): p. 750.
18. Silal, S.P., et al., *Exploring inequalities in access to and use of maternal health services in South Africa.* 2012. **12**: p. 1-12.
19. Tsawe, M. and A.S.J.B.r.n. Susuman, *Determinants of access to and use of maternal health care services in the Eastern Cape, South Africa: a quantitative and qualitative investigation.* 2014. **7**: p. 1-10.
20. Zegeye, B., et al., *Barriers and facilitators to accessing health care services among married women in Ethiopia: a multi-level analysis of the Ethiopia demographic and health survey.* 2021.
21. Demsash, A.W., B. Chakilu, and A. Mazengia, *Knowledge Sharing Practice and Its Associated Factors Among Healthcare Providers at University of Gondar Comprehensive Specialized Hospital, North West Ethiopian: Cross-sectional Study.* 2021.
22. Seidu, A.-A.J.P.O., *Mixed effects analysis of factors associated with barriers to accessing healthcare among women in sub-Saharan Africa: Insights from demographic and health surveys.* 2020. **15**(11): p. e0241409.
23. Tsegaw, M., B. Mulat, and K.J.B.o. Shitu, *Problems with accessing healthcare and associated factors among reproductive-aged women in the Gambia using Gambia Demographic and Health Survey 2019/2020: a cross-sectional study.* 2023. **13**(8): p. e073491.
24. Seidu, A.-A., et al., *Individual and contextual factors associated with barriers to accessing healthcare among women in Papua New Guinea: insights from a nationwide demographic and health survey.* 2021. **13**(6): p. 573-585.
25. Seidu, A.-A., et al., *Barriers to accessing healthcare among women in Ghana: a multilevel modelling.* 2020. **20**: p. 1-12.
26. Minyihun, A., Z.T.J.R.M. Tessema, and H. Policy, *Determinants of access to health care among women in East African countries: a multilevel analysis of recent demographic and health surveys from 2008 to 2017.* 2020: p. 1803-1813.

27. Sen, K.K., et al., *Do education and living standard matter in breaking barriers to healthcare access among women in Bangladesh?* 2023. **23**(1): p. 1431.
28. Dimbuene, Z.T., et al., *Women's education and utilization of maternal health services in Africa: a multi-country and socioeconomic status analysis.* 2018. **50**(6): p. 725-748.
29. Zegeye, B., et al., *Predictors of institutional delivery service utilization among women of reproductive age in Senegal: a population-based study.* 2021. **79**: p. 1-11.
30. Demsash, A.W., A.D.J.B.H. Walle, and C. Informatics, *Women's health service access and associated factors in Ethiopia: application of geographical information system and multilevel analysis.* 2023. **30**(1).
31. Tamang, T.M. *Factors associated with completion of continuum of Care for Maternal Health in Nepal*. in *IUSSP XXVIII International Population Conference, Cape Town, South Africa*. 2017.
32. Fentie, E.A., et al., *Spatial distribution and determinants of barriers of health care access among female youths in Ethiopia, a mixed effect and spatial analysis.* 2023. **13**(1): p. 21517.
33. Alamneh, T.S., et al., *Barriers for health care access affects maternal continuum of care utilization in Ethiopia; spatial analysis and generalized estimating equation.* 2022. **17**(4): p. e0266490.
34. Kalule-Sabiti, I., A.Y. Amoateng, and M.J.A.P.S. Ngake, *The effect of socio-demographic factors on the utilization of maternal health care services in Uganda.* 2014: p. 515-525.
35. Shiferaw, S., et al., *Why do women prefer home births in Ethiopia?* 2013. **13**: p. 1-10.
36. Yaya, S., G. Bishwajit, and V.J.B.g.h. Shah, *Wealth, education and urban–rural inequality and maternal healthcare service usage in Malawi.* 2016. **1**(2): p. e000085.
37. Voto, T.P., B.E. Voto, and N.J.E. Ngepah, *The Impact of Out-of-Pocket Health Expenditure and Public Health Expenditure on Poverty in Sub-Saharan Africa.* 2025. **13**(5): p. 134.
38. Alamneh, T.S., et al., *Socioeconomic inequality in barriers for accessing health care among married reproductive aged women in sub-Saharan African countries: a decomposition analysis.* 2022. **22**(1): p. 130.
39. AKINWALE, A.A.J.I.J.o.I.R. and A. Studies, *Gender equity and social progress: empowering women and girls to drive sustainable development in Sub–Saharan Africa.* 2023.
40. Arhin, K., E.F. Oteng-Abayie, and J.J.H.e.r. Novignon, *Assessing the efficiency of health systems in achieving the universal health coverage goal: evidence from Sub-Saharan Africa.* 2023. **13**(1): p. 25.

41. Muhajarine, N., et al., *Inequalities and factors associated with maternal healthcare services utilisation in Mozambique: evidence from the Demographic and Health Survey 2022– 2023.* 2025. **10**(5).

42. Zhao, Y., et al., *Highly sensitive, wearable piezoresistive methylcellulose/chitosan@ MXene aerogel sensor array for real-time monitoring of physiological signals of pilots.* 2025. **68**(2): p. 542-551.

Figure legend

Fig 1: Barriers to accessing health care and determinant factor among reproductive age women in sub-Saharan Africa

Supplementary file

S1. Table 5: Bivariable logistic regression of determinate factors associated with barriers to healthcare access among women in Sub-Saharan Africa.

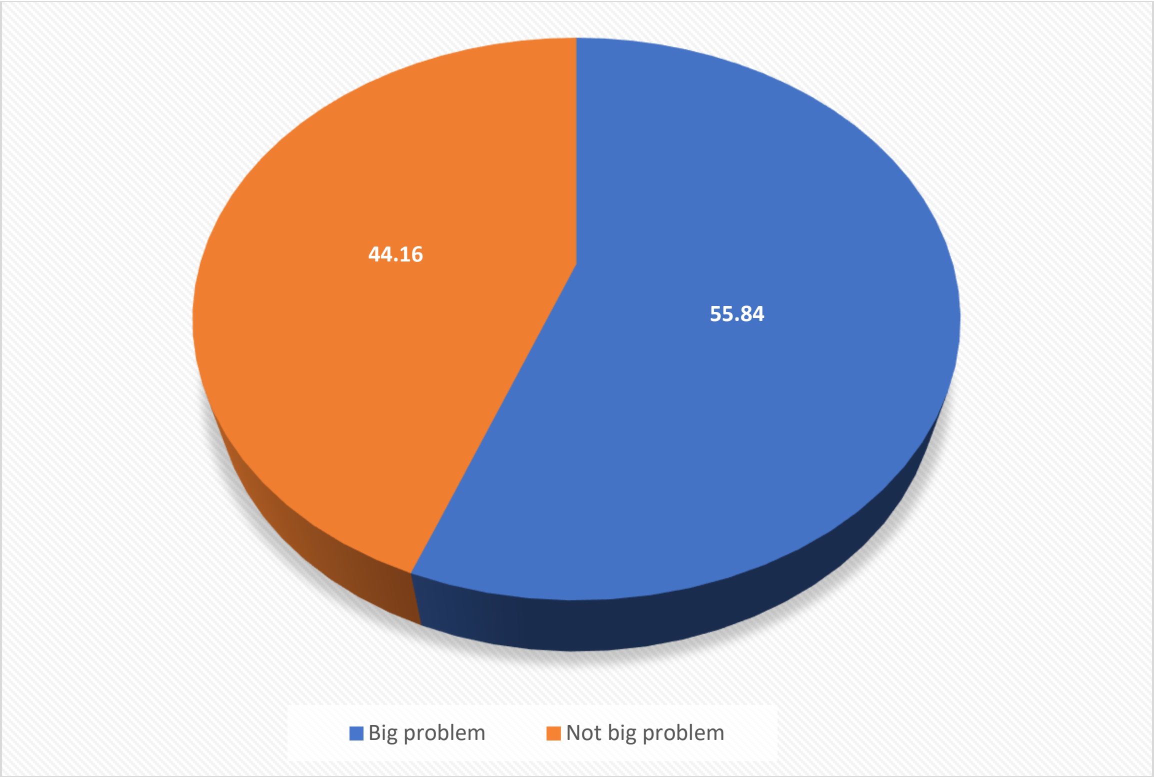

Figure 1: Barriers to accessing health care and determinant factor among reproductive age women in sub-Saharan Africa.

Click here to access/download
Supporting Information
VIF AND AIC AND BIC table.docx

Click here to access/download
Supporting Information
sulimentary table 5.docx

~~Title:~~ Barriers to ~~accessing health care~~healthcare access among ~~women's~~women in ~~Sub~~sub-Saharan Africa: ~~Multilevel Analysis~~A pooled analysis of ~~Recent Demographic and Health Survey from~~ multi-country DHS data (2019-2023~~.~~).

Formatted: Left, Space After: 0 pt

Gebreeyesus Abera Zeleke[1]*, Enyew Getaneh Mekonen[1], Bewuketu Terefe[2], Mulugeta Wassie[3], Agazhe Aemro[3], Alebachew Ferede Zegeye[3], Mohammed Seid Ali[4], Berhan Tekeba[4], Tadesse Tarik Tamir[4], Belayneh Shetie Workneh[5]

[1] Department of Surgical Nursing, School of Nursing, College of Medicine and Health Sciences, University of Gondar, Gondar, Ethiopia

[2] Department of Community Health Nursing, School of Nursing, College of Medicine and Health Sciences, University of Gondar, Gondar, Ethiopia.

[3] Department of Medical Nursing, School of Nursing, College of Medicine and Health Sciences, University of Gondar, Gondar, Ethiopia;

[4] Department of Pediatrics and Child Health Nursing, School of Nursing, College of Medicine and Health Sciences, University of Gondar, Gondar, Ethiopia.

5 Department of Emergency and Critical Care Nursing, School of Nursing, College of Medicine and Health Sciences, University of Gondar, Gondar, Ethiopia

Correspondence Author*
Gebreeyesus Abera Zeleke

Department of Surgical Nursing, School of Nursing, College of Medicine and Health Science, University of Gondar, Gondar, Ethiopia.

Email address: gebreeyesusabe143@gmail.com

Co-authors:

Enyew Getaneh Mekonen **Email Address:** enyewgetaneh12@gmail.com

Bewuketu Terefe: **Email address**: woldeabwomariam@gmail.com

Mulugeta Wassie: **Email address:** mulugeta2113@gmail.com

Agazhe Aemro: **Email address:** agazhe1049@gmail.com

Alebachew Ferede Zegeye: **Email address:** alexferede24@gmail.com

Mohammed Seid Ali: **Email address:** muheseid2592@gmail.com

Berhan Tekeba: **Email address:** berishboss7@gmail.com

Tadesse Tarik Tamir: **Email address:** tadestar140@gmail.com
Belayneh Shetie Workneh: **Email address:** belay2319@gmail.com

~~Abstract~~Abstracts

Background:

Barriers to accessing healthcare have significantly contributed to the high rates of maternal and child mortality and morbidity in developing regions, particularly in Sub-Saharan Africa. Access to healthcare is ~~shaped~~influenced by multiple factors, including financial constraints, geographic location, the availability of services, and the quality of care provided. This study aimed to assess the magnitude of these barriers and to identify the factors influencing healthcare access among women of reproductive age in Sub-Saharan Africa.

Methods:

This study utilized secondary data from the most recent Demographic and Health Surveys conducted between 2019 and 2023 across Sub-Saharan Africa. A weighted sample of 134,470 women of reproductive age was included in the analysis. Data were analyzed using Stata version 14, employing a multilevel mixed-effects logistic regression model. Factors associated with barriers to healthcare access were considered statistically significant at P-values less than 0.05. Adjusted odds ratios (AORs) with 95% confidence intervals (CIs) were used to interpret the results.

~~Result~~Results: In this study, the ~~magnitude~~prevalence of barriers to ~~accessing~~ healthcare access among ~~reproductive age~~ women ~~were~~ found to be 55.84% (95% CI:–55.57~~–~~, 56.10). ~~Husband's~~Husband educational status ~~[~~(AOR ~~=~~ =1.23, 95% CI: 1.15~~–~~, 1.30~~], Media~~), media exposure ~~[~~(AOR~~=~~=1.17, 95% CI: 1.11~~–~~, 1.24~~],~~), antenatal care ~~(ANC)~~ visit ~~status~~ ~~[~~(AOR~~=~~=1.31, 95% CI: 1.10~~–~~, 1.55~~], and place of~~), delivery ~~[~~place (AOR~~=~~=1.33, 95% CI:~~-~~1.24~~–~~, 1.43~~]. Health~~), health insurance ~~utilization~~ ~~[~~(AOR~~=~~=1.18, 95% CI: 1.11~~–~~, 1.26~~],~~), wealth index ~~status,~~(AOR=2.29, 95% CI 2.14, 2.45), (AOR=1.57, 95CI: 1.46, 1.68) and visiting ~~a~~ health facility within the past 12 months ~~[~~(AOR~~=~~=0.91,~~-~~95% CI:~~-~~0.87~~–~~, 0.97~~]~~) were individual level factors associated with outcome variable. In addition, place of residence ~~[~~(AOR~~=~~=1.30, 95% CI:~~-~~1.22~~–~~, 1.39~~],~~), community literacy level ~~[~~(AOR~~=~~=1.13, 95% CI:~~-~~1.03~~–~~, 1.24~~],~~), community ~~wealth index~~

Formatted: Font: Times New Roman, 16 pt, Bold, Font color: Auto
Formatted: Heading 2
Formatted: Font color: Auto
Formatted: Font: 16 pt
Formatted: Font: Times New Roman, 16 pt, Bold, Font color: Auto
Formatted: Font color: Auto
Formatted: Heading 2
Formatted: Font: 16 pt, Bold
Formatted: No Spacing, Space Before: 12 pt

[poverty level (AOR = =1.12, 95% CI: 1.02–, 1.22],), and Region were community level factors associated with barriers to accessing healthcare. access.

Formatted: Font: Bold

Conclusion ~~**and recommendation:**~~ ~~This~~**:** In this study ~~found that 55.55%~~, over half of women in Sub-Saharan Africa ~~experienced~~face significant barriers to ~~accessing~~ healthcare~~.~~ access. Educational status, media exposure, place of residence, antenatal care attendance, place of delivery, health insurance coverage, wealth index, recent visits to health facilities, community literacy levels, community wealth status, and regional differences were determinant factors. These findings highlight the need for Sub-Saharan African governments to prioritize these determinants when formulating policies and strategies aimed at reducing preventable barriers to healthcare access in the region.

Key words: Access to Healthcare; Barriers to Healthcare; Sub-Saharan Africa; Reproductive Age Women

Introduction

Access to healthcare is broadly defined as the ability to prevent illness, disability, and premature death, to promote and maintain health, and to attain health equity, particularly for women of reproductive age, by offering inclusive, high-quality care and the best possible availability, affordability, and accessibility of medical services[1-3]. Reproductive-age women's health is a critical global public health concern since it influences population health outcomes and fosters socioeconomic growth[4]. The advancement of socioeconomic development is significantly influenced by the health of women of reproductive age, especially in Africa. [5]. The Sustainable Development Goals (SDGs) place a strong emphasis on women's health, with Targets 3.7 and 3.8 of SDG 3 specifically focusing on achieving universal health coverage by 2030 and ensuring access to sexual and reproductive health services, including education and information on family planning[6, 7]. The Sustainable Development Goals (SDGs) explicitly target a substantial reduction in maternal mortality, with Goal 3.1 aiming to lower the global maternal mortality ratio to fewer than 70 per 100,000 live births by 2030. While these global efforts have contributed to some improvements in women's health outcomes, they have yet to fully address the root causes of maternal mortality. Achieving sustained and comprehensive progress remains a critical challenge. In 2016, Sub-Saharan Africa accounted for a disproportionately high share of the 303,000 maternal deaths reported globally, the majority of which were due to preventable causes. Moreover, non-communicable diseases continue to contribute significantly to female morbidity and mortality across nearly all countries, with a particularly severe impact in low- and middle-income settings, where many women are left with long-term disabilities [4-6]. Access to healthcare involves multiple dimensions, including financial affordability, geographic accessibility, availability of medical services, and the quality of care provided in health facilities. [8]. Access to healthcare is a key determinant of whether individuals utilize health services. Globally, approximately 400 million people lack essential healthcare, and around eight million die each year from preventable diseases—resulting in an estimated economic loss of $6 trillion in low- and middle-income countries. [6, 9, 10]. In 2015, world leaders pledged to achieve universal health coverage as part of the Sustainable Development Goals (SDGs), aiming to ensure access to affordable, high-quality medicines and financial protection for all [11, 12]. In 2017, the World Bank and WHO reported

Formatted: Font: Times New Roman

Formatted: Font: Times New Roman, 12 pt

Formatted: Font: Times New Roman, 12 pt

that half of the global population lacks access to essential health services. As a result, many countries in Sub-Saharan Africa have made universal healthcare a key part of their national health strategies. However, there has been slow progress in turning this commitment into tangible results, particularly in securing national funding for equitable, high-quality healthcare, financial security, and overall health [6, 9, 13, 14]. ~~-~~Reproductive age women and child mortality from low family planning uptake, unsafe abortion practices, unintended pregnancies, and an increase in home births are all implications of women in reproductive age having trouble accessing health care[14]. Previous research has offered some evidence regarding the sociodemographic and economic factors that can be barriers to accessing healthcare, such as marital status, place of residence, maternal age, wealth index, educational attainment, and unemployment status. Nevertheless, women of reproductive age continue to face significant disparities in healthcare ~~utilization~~access and Post-COVID-19 evidence on healthcare access remains limited, particularly in low-resource settings; this study addresses gap by leveraging recent DHS data to examine regional disparities, intersectional demographics, and implications for health system recovery and healthcare access[10, 14-16]. This study makes three unique contributions to the body of existing literature. In order to provide a current and policy-relevant analysis of the barrier to healthcare access, it first makes use of the most recent ~~(~~2019~~–2023) Demographic~~2023Demographic and Health Survey (DHS) data. Second, our analysis takes a harmonized multi-country approach across ten sub-Saharan African nations, improving cross-national comparability and generalizability in contrast to many previous studies that are limited to single-country analyses or rely on older datasets. In order to gain a better understanding of the structural and contextual factors that influence healthcare access, the study employs a comprehensive multilevel analysis of determinants at the individual and community levels. These contributions put our research in a position to guide focused, empirically supported programs provide fair access to healthcare for reproductive age women in sub-Saharan. Therefore, this study aimed to ascertain the magnitude and potential contributing factors associated with barriers to ~~accessing~~ healthcare access among ~~women of~~ reproductive age women in sub-Saharan Africa.

Methods ~~and materials~~

Formatted: Normal, Justified

~~## Patient and public involvement statement~~

~~Secondary data from the Demographic and Health Surveys (DHS) were used in this investigation.~~

Study design, study area, and period.

Data from ten Sub-Saharan African countries, collected between 2019 and 2023, were used to perform a multilevel mixed-effects analysis, based on the most recent Demographic and Health Surveys (DHS). The DHS collects community-based, cross-sectional data approximately every five years to generate updated health and health-related indicators.

Patient and public involvement statement

This study was used, Secondary data from the Demographic and Health Surveys data.

Data source, study population and sampling technique

A secondary data analysis was conducted using Demographic and Health Survey (DHS) data collected between 2019 and 2023 from selected sub-Saharan African countries. The study specifically included DHS datasets from ten countries: Burkina Faso, Gabon, Ghana, Kenya, Liberia, Rwanda, Sierra Leone, Tanzania, The Gambia, and Mozambique, each of which had recent and publicly available survey data within the specified timeframe. The datasets were appended to assess the extent of, and factors underlying, barriers to healthcare access among women of reproductive age in sub-Saharan Africa. Each country's DHS comprises multiple datasets. The DHS employs a stratified, two-stage cluster sampling design: in the first stage, enumeration areas are selected, followed by systematic sampling of households within each selected area in the second stage.

The variable barrier to ~~accessing health care were~~healthcare access was generated by computation of (v467b, v467c, v467d, v467f) from maternal recode (IR) data set was recoded. A binary logistic regression model was used to identify determinants ~~a~~as factor ~~barrier to~~ barriers healthcare access ~~the health care.~~. Determinants ~~of barrier to accessing the health care~~barriers to healthcare access were reported in terms of an AOR with a significance level of (95~~%).~~%) confidence interval. In the univariate analysis, at 95% confidence intervals with a p-value of < 0.25 was considered for the candidate multivariable analysis of data. ~~-~~All variables with p values of <0.05 were considered statistically significant. Since DHS data are nationally representative, stratified multistage cluster sampling is required; ~~therefor~~therefore, sampling weights must be applied to produce unbiased

Formatted: Normal

Formatted: Font: Times New Roman, 12 pt

Formatted: Font color: Auto

estimates. To correctly ~~weight~~weigh DHS data, we ~~use~~used the sampling weights provided in the dataset, specifically the individual weight **(**v005**)** for women's individual recode files. This weight must be divided by 1,000,000 (v005/1000000). using "gen wgt=v005/1000000 Stata commands. A total weighted sample of 134,470 reproductive age women were included in the study **(Table 1).**

Table 1: The weighted Sample size for magnitude and determinants of ~~barrier~~barriers to accessing health care among reproductive age women in sub-Saharan Africa**.**

**Country**	**Year of survey**	**weighted sample (n)**	**weighted sample (%)**
~~Brukinefaso~~ Burkina Faso	2021	~~17,659~~17659	13.13
Gabon	2019-2021	~~6,507~~6507.15	4.83
Ghana	2022	~~15,014~~15014	11.16
Kenya	2022	~~16,716~~16716.36	12.43
Liberia	2019-2020	~~8,065~~8065	5.99
Rwanda	2019-2020	~~14,634~~14634	10.88
~~Sera lion~~Sierra Leone	2019	~~15,574~~15574	11.58
Tanzania	2022	~~15,254~~15254	11.34
Gambia	2019-2020	~~11,865~~11865	8.08
Mozambique	2023	~~13,181~~13181.93	9.80
TOTAL		~~134,470~~134470	100%

Study ~~variable~~variables

Outcome variables: The outcome variable of this study was ~~barrier to access the health care. The Data in barrier of accessing the health care were collected among reproductive age women who used one of the health cares. Measurement of the~~barriers to healthcare access. Based on DHS guidelines, outcome ~~variables~~variable was ~~determined by using computation of~~constructed as a composite measure based on four ~~variables; such as Distance~~indicators: (1) distance to a health facility ~~(v467b),~~, (2) difficulty in obtaining money ~~(v467c), getting~~ for treatment, (3) difficulty in obtaining permission ~~go~~ to ~~the doctor (v467d)~~ visit a health facility, and ~~not wanting~~(4) reluctance to go to a health facility alone ~~(v467f). If a woman of reproductive age reported~~. Women who respond experiencing at least one of ~~the following~~ these four challenges~~, such~~ were classified as ~~difficulty in obtaining money, distance to a health facility, lack of permission to seek treatment, or reluctance to go alone were considered to face~~facing significant barriers to healthcare access~~. These cases~~ and were coded as ~~'1'~~1 and ~~labeled~~leveled as ~~'~~"big problem~~.'~~". Conversely, women who ~~did not report~~respond no difficulty in any of ~~these~~the four ~~barriers~~indicators were ~~considered to have~~categorized as having no significant barriers to healthcare access~~. These~~ and were coded as ~~'0'~~0 and ~~labeled~~leveled as ~~'~~ "no big ~~problem'. This approach is consistent with prior literature using DHS data~~problem"[17]

~~**Independent Variable:**~~. Handling missing value on outcome variables were retained in the denominator accordance with DHS analytical guidelines.

Individual level Variables: Hence DHS data are hierarchical, independent factors come from two sources; that was at individual and community levels factors were considered for this analysis. The individual-level independent variables were women's age (15-19, 20-35, 36-49), ~~women~~Women educational status (~~No~~no education, Primary education , Secondary ~~and~~& Higher education), women occupation (working, not working), Religion (Muslim, Christian , Others), Wealth index (Poor, Middle, Rich) media exposure (yes, No), internet utilization (yes, No), Residency (urban, rural), Husband educational status (No education, Primary education, ~~secondary and higher~~Secondary& Higher education), Husband occupational status (No working, Working), ~~Currently Contraceptive~~using Contraception (No, Yes) Visiting health facility in past 12

Formatted: Font: Not Bold
Formatted: Default Paragraph Font, Font: +Headings (Calibri Light)
Formatted: Strong, Font: +Headings (Calibri Light)
Formatted: Strong, Font: +Headings (Calibri Light)
Formatted: Strong, Font: +Headings (Calibri Light)
Formatted: Strong, Font: +Headings (Calibri Light)
Formatted: Font: Bold
Formatted: Strong, Font: +Headings (Calibri Light)
Formatted: Strong, Font: +Headings (Calibri Light)
Formatted: Strong, Font: +Headings (Calibri Light)
Formatted: Strong, Font: +Headings (Calibri Light)
Formatted: Strong, Font: +Headings (Calibri Light)
Field Code Changed
Formatted: Normal
Formatted: Font: 12 pt
Formatted: Font: 12 pt
Formatted: Font: 12 pt
Formatted: Font: 12 pt
Formatted: Font: 12 pt
Formatted: Font: 12 pt
Formatted: Font: 12 pt
Formatted: Font: 12 pt
Formatted: Font: 12 pt

monthmonths (No, Yes), wanted pregnancy(yes, no) .place of delivery (Home, Institutional delivery), ANC visit (No antenatal visit, 1-4 visit, > 4 visit).

The community-level ~~variables:~~ Community povertyVariables: Poverty level (Low, High), Community literacyLiteracy level (Low, High), Community mediaMedia exposure, (Low, High), Place of residencyResidency (Urban, ruralRural), Region (East, west) in sub Saharan Africa.West), Community ANC visit,

Data processing and statistical analysis

Stata version 14 statistical software was used to clean, record, and analyze secondary DHS data sets. The variables in the DHS data are grouped into clusters, and the variables within a cluster are more similar to each other than those in other clusters. The presumptions of equal variance across clusters and independent data were violated in order to run a typical logistic regression model. This suggests that between-cluster factors must be considered using a complex model.

Given this, multilevel mixed-effects logistic regression model was used to determine the factors that associated with barrier to accessing the health care. Multilevel mixed effect logistic regression follows four models: the null model (only outcome variable), mode I (individual level variables), model II (community level variables), and model III (both individual and community level variables). The null model without independent variables. The null model (only outcome variable), mode I (individual level variable such as age, Religious status, educational status, Husband educational, marital occupation, Husband occupation, marital status, Contraceptive Utilization, place of delivery, ANC visit, media exposure, Intermate utilization, visiting health facility within 12 months, health insurance , wealth index) model II community level variables, Region in sub-Saharan Africa, community poverty level, community media exposure, Community distance health facility, Community literacy level, Community ANC visit), and model III (both individual and community level variables such as age, Religious status, educational status, Husband educational, marital occupation, Husband Occupation, marital status, Contraceptive Utilization, place pf delivery, ANC visit, media exposure, Intermate utilization, visiting health facility within 12 months, health insurance , wealth index), model II (community level variables, Region in sub-Saharan Africa, community poverty level, community media exposure, Community distance health facility, Community literacy level, Community ANC visit). The null model was used to check the variability of outcome variable across the cluster. The relationship of individual-level

Formatted: Font: 12 pt

Formatted: Font: 12 pt

Formatted: No Spacing, Justified

Formatted: Font: 12 pt

Formatted: Font: 12 pt

Formatted: Font: 12 pt

Formatted: Font: 12 pt

Formatted: Font: 12 pt

Formatted: Font: Times New Roman, Bold

variables with outcome variable (Model I) and the association of community-level variables with outcome variable (Model II) were determined. In the final model, the association of both individual and community-level variables was fitted with the outcome variable.

Random effects

The intra-class correlation coefficient (ICC), proportionate change in variance (PCV), and median odds ratio (MOR) were used to quantify random effects or measures of variation of the outcome variables.

To assess the variation between clusters, the proportionate change in variance (PCV) and intra-cluster correlation coefficient (ICC) were calculated. as By treating the cluster as a random variable, the ICC shows that the variation in the health care access barrierbarriers between clusters may be computed as follows; follow $ICC = \frac{VC}{VC+3.29} \times = \frac{\sigma^2 uo}{\sigma^2 uo+\pi^2/3} \times 100\%$. The MOR, which is determined by using clusters as a random variable and calculating the median value of the odds ratio between the highest-risk and lowest-risk areas for barriers to healthcare access was calculated as: $MOR = \exp(0.6745\ \sqrt{(2 * \sigma^2 uo)})$ Furthermore, the PCV calculated as: variable that explains the variation in the degree of barriers to healthcare access $PCV = \frac{\sigma^2 uo - \sigma^2}{\sigma^2 uo} \times 100\%$;, where VC is the cluster level variance and Vnull is the variance of the null model. health care access barriers, is calculated;

MOR= $e^{0.95\sqrt{VC}}$. Furthermore, the PCV calculates a variable that explains the variation in the degree of barriers to accessing healthcare ; $PCV = \frac{Vnull-VC}{Vnull} \times 100\%$; where VC is the cluster level variance and Vnull is the variance of the null model.

The fixed effects were employed to calculate the correlationassociations between the probability of barriers to obtaining health care access and independent factors at the individual and community levels. and barriers to accessing health care were estimated using the fixed effects. It was assessed and presented using adjusted odds ratio (AOR) and 95% confidence intervals with a p-value of < 0.05. Because of the nested nature of the model, deviation = -2(log likelihood ratio) was used to compare models, and the model with the lowest deviance was selected as the best-fitted model. By evaluating the variance inflation factors, the model assumption for multi-collinearity was confirmed, and the results fell within an acceptable range of 1.03-1.98.. In addition., Model comparison was performed using Akaike's Information Criterion (AIC). The model

Formatted: Font: Bold

Formatted: Font: Bold

Formatted: Font: Bold

Formatted: Font color: Custom Color(RGB(34,34,34)), Pattern: Clear (White)

Formatted: Font color: Custom Color(RGB(34,34,34)), Pattern: Clear (White)

Formatted: Font color: Custom Color(RGB(34,34,34)), Pattern: Clear (White)

Formatted: Font color: Accent 1

includingincludes both random intercepts and random slopes (Model III) had the lowest AIC= (was evaluated alongside alternative models, with AIC=239608.6), indicating the best balance between values calculated to assess relative model fit and parsimony.

Formatted: Font color: Accent 1

Ethical approval and consent to participate

This study was based on analysis of existing survey datasets in the public domain (SSA) that are freely available online with all the identifier information anonymized, no ethical approval was required

Patient and public involvement

Since this study data was obtain from demographic and health survey (DHS) secondary source, no patient and public were involved.

Result

.

Results

In this study a total of 134,470 reproductive age women were included infrom 10 Sub-Saharan African countries. The Mean age of participants werewas 28.99 (Std. Dev.years with SD. 9.68) years, and the Variance of 93.86557.. More than two thirds (69.8)%) of women did not use contraception, and over 72.22% reproductive age women did not use health insurance, and over half of (54.22%) of women were living in rural areas in sub-Saharan Africa. **(Table 2)**

Table 2: Sociodemographic and economic characteristics of reproductive age women who had barriers to accessing health care in sub-Saharan Africa.

Variable	frequency(n)	percentage (%)
**Individual and community level variables**		
**Maternal age**		
15-19	27,890.4	20.7
20-35	69,441.4	51.64
36-49	37,138.6	27.64
**Residency**		
Urban	61,558.03	45.78
Rural	72,912.41	54.22
**Religious status**		
Muslim	48834.95	41.96
Christian	46856.90	39.30
Other	23,524.59	19.73

**Educational Status**		
No formal education	35,051.70	26.06
Primary education	39,651.17	29.48
Secondary and high	59,767.57	44.44
**Husband educational**		
No formal education	29,414.44	36.72
Primary education	21,245.78	26.52
secondary and above	29,428.78	36.74
**Marital Occupational**		
Not working	50,732.90	37.81
working	83,417	62.18
**Husband Occupation**		
Not working	10,390.43	13.0%
Working	69,698.11	87.%
**Marital status**		
single	42,213.40	31.4
Married	58,262.00	43.3
Other	33,995.4	25.3
**Contraceptive Utilization**		
No	93,855.00	69.8
Yes	40,615.5	30.2
**place of delivery**		
Home	8,279.46	15.5
Institution	44,979.28	84.5
ANC visit		
No visiting	2,012.84	3.77
1-4 visiting	24,459.34	45.92
>4 visiting	26,786.55	50.29
**Media exposure**		
No	34,628.45	25.75
Yes	99,831.57	74.24
**Intermate utilization**		
No	93,020.34	69.17
Yes	41,450.10	30.82
**Visiting health facility 12 month**		
No	57,137.73	42.49
Yes	77,332.71	57.50
**Health insurance**		
No	85,044.69	72.22
Yes	32,709.39	27.77

**Wealth index**		
poor	47,227.18	35.12
middle	25,771.45	19.16
High	61,471.81	45.74
**Region in sub-Saharan Africa**		
East Africa	59,786.29	44.46
West Africa	74,684.15	55.53
**Community poverty level**		
Low	70,776.71	52.63
High	63,693.73	47.47
**Community media exposure**		
Low	61,642.30	45.84
High	72,828.14	54.15
**Community distance health facility**		
Low	65,962	49
High	68,508	51
**Community literacy level**		
Low	57,615.01	42.84
High	76,855.43	57.15
**Community ANC visit**		
Low	77,912.32	57.96
High	56,491.92	42.03

**Individual and Community level variables**	**Frequency(n)**	**Percentage (%)**
**Individual level variables**		
**Maternal age**		
15-19	27890.4	20.7
20-35	69441.4	51.64
36-49	37138.6	27.64
**Religious Status**		
Muslim	48834.95	41.96
Christian	46856.90	39.30
Other	23524.59	19.73
**Educational Status**		
No formal education	35051.70	26.06
Primary education	39651.17	29.48
Secondary and higher education	59767.57	44.44
**Husband's Education**		
No formal education	29414.44	36.72

Primary education	21245.78	26.52
Secondary and Higher education	29428.78	36.74
**Maternal Occupational**		
Not working	50732.90	37.81
Working	83417.01	62.19
**Husband Occupation**		
Not working	10390.43	13.00
Working	69698.11	87.00
**Marital Status**		
Single	42213.40	31.4
Married	58262.00	43.3
Other	33995.4	25.3
**Contraceptive Utilization**		
No	93855.00	69.8
Yes	40615.5	30.2
**Place of Delivery**		
Home	8279.46	15.5
Institution	44979.28	84.5
**ANC visit**		
No visiting	2012.84	3.77
1-4 visiting	24459.34	45.92
>4 visiting	26786.55	50.29
**Media exposure**		
No	34628.45	25.75
Yes	99831.57	74.24
**Internet utilization**		
No	93020.34	69.17
Yes	41450.10	30.82
**Visited Health Facility Last 12 Months**		
No	57137.73	42.49
Yes	77332.71	57.50
**Health Insurance**		
No	85044.69	72.22
Yes	32709.39	27.77
**Wealth Index**		
Poor	47227.18	35.12
Middle	25771.45	19.16
Rich	61471.81	45.74
**Community level variables**		

**Region in sub-Saharan Africa**		
East Africa	59786.29	44.46
West Africa	74684.16	55.54
**Residency**		
Urban	61558.03	45.78
Rural	72912.41	54.22
**Community poverty level**		
Low	70776.71	52.63
High	63693.73	47.47
**Community media exposure**		
Low	61642.30	45.84
High	72828.14	54.15
**Community distance health facility**		
Low	65962	49.00
High	68508	51.00
**Community literacy level**		
Low	57615.01	42.84
High	76855.43	57.15
**Community ANC visit**		
Low	77912.32	57.96
High	56491.92	42.03

In this study, the magnitude of barriers to accessing health care among reproductive age women was found to be 55.84 (95% CI: (55.57, 56.10) owing to one or more of the four factors, the most commonly cited being the difficulty in getting money and the distance from medical facilities **(Figure 1)**

Random effect (Measures of variation) and model fitness

To determine if the data supported the choice to evaluate randomness at the community level, a null model was employed. With athe variance of 0.3784967 and a p--value of < 0.000001, the null model's results showed that there were notable variations in the barriers to accessing healthcare among clusters. A significant amount of the variance in the outcome between clusters was jointly explained by both individual- and community-level variables, as evidenced by the intra-cluster correlation (ICC), which dropped from 10.31% in the null model to 3.96% in the fully adjusted model (model III). According to the proportionate change in variance (PCV), Model III explains

the greatest percentage (64.09%) of the group-level variation in comparison to the null model, group comparison showed that, Model I (50.19%) and Model II (24.56%). This suggests that the model fits better as more predictors are included. ~~Akaike information criteria~~Model comparison using Akaike's Information Criterion (AIC) ~~Among the models evaluated,~~indicated that Model III ~~demonstrated~~achieved the best fit~~ with~~, as evidenced by the lowest AIC (239,608.6), ~~indicating a substantial improvement over~~markedly lower than both the null model (AIC = 1,128,437) and Model II (AIC = 1,127,704). Model I also showed good fit (AIC = 239,764.8), though slightly less optimal than Model III ~~**(Table 3).**~~and highest log-likelihood value (−21,433.284) among all competing models. Multicollinearity using variance inflation factors (VIF) indicated that all VIF values ranged from 1.03-1.98, which is within accepted range **(Table 3)**

Table 3: Model comparison and Fitness random effect analysis for barriers of accessing health care of among reproductive age women in sub-Saharan Africa**.**

**Parameter**	**Null model**	**model I**	**model II**	**model III**
**Variance**	0.3784967	0.1885269	0.28553	0.1358817
**ICC**	10.31%	5.41%	7.98%	3.96%
**MOR**	1.79	1.51	1.66	1.41
**PCV**	Reference	50.19%	24.56%	64.09%
**Model ~~fines~~Fitness**				
**LLR**	-90175.365	-21798.27	-86841.704	-21433.284
**Deviance**	1803596.73	43596.54	173683.408	42866.568
**AIC**	1128437	239764.8	1127704	239608.6

ICC: intra-cluster correlation**, LLR:** loglikelihood ratio**, MOR:** median odds ratio**, PCV:** proportional change in variance. **AIC:** Akaike information criteria.

Formatted: Font: Not Bold

Formatted: Font: +Body (Calibri), 11 pt

Formatted: Line spacing: 1.5 lines

Formatted: Font: Bold

Formatted: Line spacing: 1.5 lines

Formatted: Font: Bold

Formatted: Line spacing: 1.5 lines

Formatted: Font: Bold

Formatted: Line spacing: 1.5 lines

Formatted: Font: Bold

Formatted: Line spacing: 1.5 lines

Formatted: Font: Bold

Formatted: Line spacing: 1.5 lines

Formatted: Font: Bold

Formatted: Line spacing: 1.5 lines

Determinants of barriers to healthcare access among reproductive age women in Sub-Saharan Africa

Multivariable multilevel logistic regression analysis of individual and community level factors associated with barrios of accessingBarriers to health care access among reproductive age women in subSub-Saharan Africa.

In the final fitted model of multivariable multilevel logistic regression, Husband educational status, media exposure, internet utilization, place of residency, ANCAntenatal care visit status, place of delivery, health insurance utilization status, wealth index status, visiting health facility within 12 months, and country category (western, eastern Africa) were significantly associated with barriers of accessing health care among reproductive age women in sub-Saharan Africa. The odds of barrierbarriers of accessing health care were 1.30 times higher among reproductive age women living in rural residency compared to women living in an urban area (AOR=-1.30, 95% CI: (1.22, 1.39). and the odds of barrier of accessing health care barriers healthcare access were 1.23 and 1.21 times more likely to among reproductive age women whose husband had no education and primary education compared to women whose husband had secondary and above educational level respectively higher education (AOR=1.23, 95% CI: (1.15, 1.30,) and (AOR=1.21, 95% CI: (1.31, 1.41).). In addition, odds of barrier of accessing health care barriers healthcare access were 1.33 times more likely to occur in women who delivered at home compared to women who had institutional delivery. (AOR=1.33, 95% CI: (1.24, 1.43)), and the odds of barrier of accessing health carebarriers healthcare access were 1.31 and 1.13 times higher among women who had no history of antenatal visit and women who had < 4 ANC visit compared to women who had more than four ANC visit Respectively. (AOR=-1.31, 95% CI: (1.10, 1.55) and (AOR= 1.13, 95% CI: (1.07,1.19) and the odds of the barrier of accessing health care); The odds of barriers to healthcare access among reproductive age womanwomen who had no media exposure and intranet access were 1.17 and 1.68 times more likely to compared with a woman who had media exposure and internet access respectively (AOR=1.17, 95% CI: (1.11, 1.24) and (AOR= 1.68, 95% CI: (1.57, 1.79). and the barrier of accessing health carethe barriers to healthcare access among reproductive age womanwomen who were not visiting a health facility within 12 months were 9 % times less likely compared to –women who had visiting health care facility within 12 months of study period (AOR=0.91, 95% (CI: 0.87, 0.97). The odds of perceived barrier of accessing health care among reproductive age womanwomen who had no health insurance utilization were 1.18 times more

Formatted: Font: 14 pt, Font color: Text 1

Formatted: Heading 3, Left

Formatted: Font color: Text 1

Formatted: Font color: Text 1

Formatted: Font color: Text 1

Formatted: Font color: Text 1

Formatted: Font color: Text 1

Formatted: Font color: Text 1

Formatted: Font: 14 pt, Font color: Text 1

Formatted: Font: Not Bold

Formatted: Font color: Auto

Formatted: Font: Not Bold

Formatted: Font: Not Bold

Formatted: Font color: Black

Formatted: Font: Not Bold

Formatted: Font color: Auto

Formatted: Font: Not Bold

Formatted: Font color: Auto

Formatted: Font: Not Bold

likely to compared with a woman who had used health insurance (AOR=1.18, 95% CI: ~~(~~1.11, 1.26~~)~~ ~~and The~~). additionally, the odds of perceived ~~barrier of accessing health care service~~barriers healthcare access among reproductive age ~~woman~~women who were classified under poor and middle wealth status were 2.29 ~~-~~and 1.57 times more likely ~~accessing health care challenge~~of barriers to healthcare access compared to reproductive age women who were classified under rich wealth status category (AOR=2.29 95% CI: ~~-(~~2.14, 2.45) and (AOR=1.57~~.~~, 95% CI: ~~-(~~1.46, 1.68). The odds of a perceived barrier of accessing health care service among reproductive age ~~woman~~women who live in ~~west~~West Africa region were 2.38 times more likely barriers ~~accessing health care~~to healthcare access compared to ~~east~~those living in East Africa region ~~category~~ (AOR=2.38, 95% CI: ~~(~~2.21, 2.55) **(Table 4~~)~~).**

Table 4: ~~-~~Multivariable multilevel logistic regression ~~analysis~~ of individual ~~-level~~ and community level ~~variable~~factors associated with ~~accessing health care~~barriers to healthcare access among reproductive age women in ~~sub-~~Sub-Saharan Africa.

Individual and community level factors	Model I AOR ~~(~~[95% CI~~)~~]	Model II AOR ~~(~~[95% CI~~)~~]	Model III AOR ~~(~~[95% CI~~)~~]
**Individual level factors**			
**Maternal age**			
15-19	0.96~~(~~ [0.86, 1.07~~)~~]		0.96 ~~(~~[0.86,1.08~~)~~]
20-35	1.06~~(~~ [1.01,1.13~~)~~]		1.04 ~~(~~[0.99, 1.11~~)~~]
36-45	1		1
**Residency**			
Urban		1	1
Rural		2.29 ~~(~~[2.24, 2.35~~)~~]	1.30 ~~(~~[1.22, 1.39~~)~~] *
**Maternal education**			
No education	1.10 ~~(~~[1.02, 1.18~~)~~]		1.04 ~~(~~[0.97, 1.11~~)~~]
Primary education	0.93 ~~(~~[0.87, 0.99~~)~~]		1.06 ~~(~~[0.99, 1.14~~)~~]
Secondary and above	1		1
**Husband education**			
No education	1.26 ~~(~~[1.19,1.35~~)~~]		1.23 ~~(~~[1.15, 1.30~~)~~] *
Primary education	1.04~~(~~ [0.97,1.12~~)~~]		1.23 ~~(~~[0.67, 1.22~~)~~]
~~secondary~~Secondary & higher education	1		1
**~~Marital~~Maternal occupation**	1		
~~working~~	0.99~~——(~~ [0.94, 1.04~~)~~]		1
~~not~~Working			1.02 ~~(~~[1.01, 1.11~~)~~] *

Formatted: Font: Not Bold
Formatted: Font: Not Bold
Formatted: Font: Not Bold
Formatted: Font: Not Bold
Formatted: Font: 14 pt, Bold, Font color: Text 1
Formatted: Justified
Formatted: Line spacing: 1.5 lines
Formatted Table
Formatted Table
Formatted: Font: Not Bold
Formatted: Font: Not Bold
Formatted: Font: Not Bold
Formatted: Normal
Formatted: Line spacing: Multiple 1.15 li
Formatted: Font: Not Bold
Formatted: Font: Not Bold
Formatted: Font: Not Bold
Formatted: Indent: Left: 0", Line spacing: Multiple 1.15 li
Formatted: Font: 11 pt
Formatted: Font: Not Bold
Formatted: Font: Not Bold
Formatted: Font: Not Bold

Not working		
**Husband occupation**		
~~working~~		
~~not~~Working	1	1
Not working	0.90 ~~(~~[0.83, 0.97~~)~~]	1.00 ~~(~~[0.92, 1.08~~)~~]
**Religion**		
Muslim	1	1
Christian	1.62 ~~(~~[1.53, 1.71~~)~~]	0.46 ~~(~~[0.57, 1.94~~)~~]
Other	1.22 ~~(~~[1.13, 1.31~~)~~]	1.36 ~~(~~[0.75, 1.46~~)~~]
**Contraceptive utilization**		
No	1.11 ~~(~~[1.05,1.17]	1.03 ~~(~~[0.98, 1.08~~)~~]
Yes	1	1
**Place of delivery**		
~~home~~		
Home	1.30 ~~(~~[1.21, 1.40~~)~~]	1.33 ~~(~~[1.24, 1.43~~)~~] *
Institution	1	**1**
~~**ANC visit**~~		
**Antenatal Care Visit**		
No	1.01 ~~(~~[0.85, 1.19~~)~~]	1.31 ~~(~~[1.10, 1.55~~)~~] *
1-4 visit	0.94 ~~(~~[0.89, 0.99~~)~~]	1.13 ~~(~~[0.37, 1.19~~)~~]
> 4 visits	1	1
**Media exposure**		
No	1.14 ~~(~~[1.08, 1.21~~)~~]	1.17 ~~(~~[1.11, 1.24~~)~~] *
Yes	1	1
**Internet utilization**		
No	1.69 ~~(~~[1.58, 1.80~~)~~]	1.68 ~~(~~[0.67, 1.79~~)~~]
Yes	1	1
**Visiting health facility last 12 ~~month~~months**		
No	0.92 ~~(~~[0.87, 0.98~~)~~]	0.91 ~~(~~[0.87, 0.97~~)~~] *
Yes	1	1
**Health insurance**		
No	1.26 ~~(~~[1.19, 1.34~~)~~]	1.18 ~~(~~[1.11, 1.26~~)~~] *
Yes	1	1
**Wealth index**		
~~poor~~		
~~middle~~		
Poor	2.80 ~~(~~[2.63, 2.97~~)~~]	2.29 ~~(~~[2.14, 2.45~~)~~] *
Middle	1.79 ~~(~~[1.68, 1.92~~)~~]	1.57 ~~(~~[1.46, 1.68~~)~~] *
Rich	1	1

Community level variable

Formatted: Font: Not Bold
Formatted: Font: Not Bold
Formatted: Font: Not Bold
Formatted: Font: Not Bold
Formatted: Font: Not Bold
Formatted: Font: Not Bold
Formatted: Font: Not Bold
Formatted: Font: Not Bold
Formatted: Font: Not Bold
Formatted: Font: Not Bold
Formatted: Font: Not Bold
Formatted: Font: Not Bold
Formatted: Font: Not Bold
Formatted: Font: Not Bold
Formatted: Font: Not Bold
Formatted: Font: Not Bold
Formatted: Font: Not Bold
Formatted: Font: Not Bold
Formatted: Font: Not Bold
Formatted: Font: Not Bold

**Residency**			
Urban		1	1
Rural		2.29 [2.24, 2.35]	1.30 [1.22, 1.39] *
**Region SSA**		0.50 (0.49, 0.52)	**0.42 (0.39, 0.45)**
East Africa		11	**1**
West Africa		2.0 [1.92-2.04]	2.38 [2.21, 2.55] *
**communityCommunity ANC visit**			
Low		1.00 ([0.93, 1.09)]	0.93 ([0.86, 1.00)]
High		1	1
**communityCommunity wealth index**			
Low		0.75 ([0.70, 0.81)]	1.12 ([1.02, 1.22)] *
High		1	1
**Community media exposure**			
Low		1.01 ([0.92, 1.10)]	0.94 ([0.86, 1.03)]
High		1	1
**Community literacy level**			
Low		1.10([1.00, 1.20)]	1.13 ([1.03, 1.24)] *
High		1	**1**

Table 5: Key Individual and Community-Level Determinants of Barriers to Healthcare Access Among Women of Reproductive Age in Sub-Saharan Africa.

Individual and community level factors	Model I AOR (95% CI)	Model II AOR (95% CI)	Model III AOR (95% CI)
**Residency**			
Urban		1	1
Rural		2.29 (2.24, 2.35)	**1.30 (1.22, 1.39)**
**Husband education**			
No education	1.26 (1.19,1.35)		**1.23 (1.15, 1.30)**
Primary education	1.04(0.97,1.12)		1.23 (0.67, 1.22)
secondary education	1		1
**Marital occupation**			
working	1		1
not working	0.99 (0.94 1.04)		**1.02 (1.01, 1.11)**
**Place of delivery**			
home	1.30 (1.21, 1.40)		**1.33 (1.24,1.43)**
Institution	1		**1**
**ANC visit**			
No	1.01 (0.85, 1.19)		**1.31(1.10,1.55)**
1-4 visit	0.94 (0.89, 0.99)		1.13(0.37,1.19)
> 4 visits	1		1

Formatted Table

Formatted: Font: Bold

Formatted: Font: Not Bold

Formatted: Font: Not Bold

Formatted: Font: Not Bold

Formatted: Font: Not Bold

Formatted: Font: Not Bold

Formatted: Font: Not Bold

Formatted: Font: Bold

Formatted: Left, Line spacing: single

**Media exposure**			
No	1.14 (1.08, 1.21)		**1.17 (1.11, 1.24)**
Yes	1		1
**Visiting health facility 12 month**			
No	0.92 (0.87, 0.98)		**0.91(0.87, 0.97)**
Yes	1		1
**Health insurance**			
No	1.26 (1.19, 1.34)		**1.18 (1.11,1.26)**
Yes	1		1
**Wealth index**			
poor	2.80 (2.63, 2.97)		**2.29 (2.14, 2.45)**
middle	1.79 (1.68, 1.92)		**1.57 (1.46, 1.68)**
Rich	1		1
**Region SSA**			
East Africa		0.50 (0.49, 0.52)	**0.42 (0.39, 0.45)**
West Africa		1	1
**community wealth index**			
Low		0.75 (0.70, 0.81)	**1.12 (1.02,1.22)**
High		1	1
**Community literacy level**			
Low		1.10(1.00, 1.20)	**1.13(1.03,1.24)**
High		1	**1**

***Model I:** individual level variable*, **Model II:** *Community level variable*, **Model III**: *Individual and community level variable*

Discussion

Barriers to ~~accessing health care~~healthcare access have contributed to maternal and child mortality and morbidity in developing countries such as those in Sub-Saharan Africa. This study aimed to assess the ~~magnitude~~prevalence and determinants of barriers to ~~health care~~healthcare access among women~~ of reproductive age~~ in Sub-Saharan Africa.

Formatted: Font: 12 pt, Not Bold

In this study, ~~the magnitude~~55.8 of women experienced barriers to ~~accessing health care among reproductive age women was found to be 55.84 (95% CI: (55.57, 56.10) owing to one or more of the four factors, the most commonly cited being the difficulty in getting money and the distance from medical facilities.~~

healthcare access. This finding is lower than ~~the~~ estimates reported in previous ~~study conducted in~~ studies from South Africa and Tanzania 65~~%~~% [18, 19]~~, and Tanzania 65% [15], Ethiopia 71.8%,70.2, 69.9%[1, 20, 21], (SSA) 60.4%, 61.5% [17]. this variation could be due to the fact that socio-economic and variability in health infrastructure and health system policy, women's attitudes towards health care seeking behavior and cultural differences across countries in sub-Saharan Africa, moreover, this discrepancy might be due to study period, this study includes, the most recent DHS data set from 2019-2023. On the other hand, the finding of this study is similar to but slightly higher than in previous study in Gambia 45.5%[22], Ghana (51%)[23]. this variation could be due to the fact that socio-economic and variability in health infrastructure and health system policy, women's attitudes towards health care seeking behavior and cultural differences across countries in sub-Saharan Africa.~~

and Ethiopia and sub-Saharan Africa 60–72% [1, 17, 20, 21]. This variation could be due to changes in socioeconomic, health infrastructure, health system policies, women's attitudes towards health care seeking behavior, and cultural differences across countries in Sub-Saharan Africa. Moreover, these discrepancies might be due to differences in the study period, because this study includes the most recent DHS data sets, from 2019 to 2023. On the other hand, the findings of this study are similar to but slightly higher than in previous studies Ghana and Gambia (51%), (45.5%) [22, 23]. This variation may be attributed to differences in socioeconomic conditions,

health infrastructure, and health system policies, as well as variations in women's health-seeking attitudes and cultural contexts across countries in sub-Saharan African.

The odds of ~~barrier of accessing health care~~barriers to healthcare access were 1.30 times higher among reproductive age women living in rural residency compared to women living in an urban area. This finding is supported by previous studies conducted in Ethiopia [1, 20], ~~guinea~~Guinea[24], Gambia, Tanzania and Ghana [15, ~~22~~23, 25]~~ respectively. Sub-Saharan[23],~~ respectively and Sub-Saharan countries[22], East Africa[26]. This association may be~~,~~ due to the fact that rural areas have low health ~~infrastructure~~infrastructures, i.e~~.~~., inaccessibility ~~of~~to health facilities, less ~~privileged~~opportunities and the influences ~~of~~to, socio-cultural practices that ~~need~~oblige women to seek permission from their ~~husband~~husbands to visit the facility ~~or~~because women's decisions for seeking healthcare services ~~is~~are under their ~~husband~~husbands/partners control. Moreover, male involvement and support for women health care access are limited[1, 14, ~~23~~22, 26]. This implies that ~~the government should~~governments better to expand infrastructure services in a rural ~~area~~areas and encourage male involvement and support for improving ~~health care~~healthcare access for reproductive age women[14].

Formatted: Font: Times New Roman, Font color: Black

Formatted: Font color: Auto

The odds of perceived ~~barrier of accessing health care~~barriers to healthcare access were 1.23 and 1.21 times more likely to among reproductive age women whose ~~husband~~husbands had no education ~~and~~or primary education compared to women whose ~~husband~~husbands had secondary and above educational level, respectively. ~~-~~This ~~find~~finding was consistent with previous ~~study~~studies conducted in east Africa, Ethiopia, Tanzania, Benin, and Bangladesh~~ respectively~~ [1, 15, 20, 26, 27]. The possible explanation could be in a better access to the healthcare among women with educated husbands might be due to better participation and involvement of husbands in their ~~-~~families' health~~[23].~~[22]. Women whose partners are educated are also likely to be informed regarding their fundamental human rights and may have higher health literacy. As a result, they are more likely to deal with any form of barrier to healthcare, compared to their counterparts who are less educated and may have lower health literacy [14, 15, 20, 28].

The odds of ~~barrier~~barriers to ~~accessing health care~~healthcare access were 1.33 times more likely to occur among women who delivered at home compared to women who had an institutional delivery. This evidence was supported by a study ~~done~~conducted in Senegal[29]. The plausible justification might be that women who delivered at home may not get health

information/counselling from healthcare providers that empower them to use other maternal healthcare services and women who deliver at home may face varous barriers to ~~accessing health care~~healthcare access such as financial constraint, lack of transportation, limited autonomy in healthcare decision and sociocultural norms that discourage institutional delivery. Moreover, they can be affected by the importance of enhancing health system outreach, strengthen community-based education, and addressing sociocultural and socioeconomic disparities that affect health seeking behavior. On the other hand, the odds of ~~barrier~~barriers of accessing health care among ~~woman~~women who had no media exposure were 1.17 times more likely to compared with ~~a woman~~women who had media exposure. This evidence was supported by ~~study done~~studies conducted in Ethiopia[30]~~,~~ and Nepal[31]. This could be the power of mass media in disseminating information concerning maternal health that may enhance women's knowledge and attitude towards health care access and utilization[31]. Barrier of accessing health care among woman who were not visiting health facility within 12 months were 9 % times less likely compared to women who had visiting health care facility within 12 months of study period. The odds of barrier to accessing health care among woman who had no health insurance utilization were 1.18 times more likely to compared with a woman who had used health insurance. This finding was supported with study done in Ethiopia[1], sub-Saharan Africa~~[23]~~[22], Tanzania[15], Ghana[25]. The possible reason might be due to the fact that, individuals who were not had health insurance faced for unexpected catastrophic expenditure and difficulties in obtaining money for consultation of the doctor[1]. The odds of barrier to accessing healthcare among woman who were classified under poor and middle wealth status were 2.29 and 1.57 times more likely accessing health care difficulty compared to women who were classified under rich wealth index category**.** This evidenced was supported with study done in Ethiopia, [1, 30, 32, 33] Tanzania[15] Uganda[34], sub Saharan Africa[14]. This finding might be explained by the fact that high out-of-pocket expenses and a lack of accessibility to healthcare facilities pose significant obstacles for middle-class and impoverished women in sub-Saharan Africa. Many people in the region cannot afford healthcare services because over 40% of health costs are paid for directly by individuals[35-38]

The odds of barrier of accessing health care among woman who live west Africa region were 2.38 times more likely barriers to accessing health care compared to east Africa region. According a recent joint report from the World Health Organization and the United Nations states that high out-of-pocket costs, a lack of adequate healthcare infrastructure, and other sociocultural barriers make

it more difficult for women of reproductive age in West African nations such as Nigeria, Mali, and Sierra Leone to access maternal healthcare. On the other hand, East African countries like Rwanda, Kenya, and Ethiopia have strengthened their community health initiatives and gender-inclusive policies, which have improved women's autonomy in accessing healthcare services[39]. Furthermore, our current findings are supported by recent Demographic and Health Survey (DHS) program data (2022–2023), which indicate that women in West African countries are 2-3 times more likely to report delays in accessing healthcare due to cost, shortages of healthcare personnel, and long distances to health facilities. In contrast, East African countries, particularly Uganda and Tanzania, have strengthened their health insurance systems and rural outreach initiatives. Additionally, the World Bank’s 2023 Health Service Coverage Index reveals that women in West Africa have significantly lower access to family planning, skilled birth attendance, and other essential reproductive health services[~~22~~23, 40, 41]

~~Strength~~Strengths and ~~limitation~~limitations

~~This~~The main strength of this study is the use of nationally representative data~~ to evaluate~~, which allows for a comprehensive evaluation of contextual and individual ~~characteristics related to~~factors associated with barriers to healthcare access~~ among reproductive age women in the SSA. Thus, findings can therefore, generalized to all women in their reproductive age in SSA, in order to account for~~. Additionally, the ~~clusters~~analysis accounted for the clustering within the sample~~, the study also employed~~ by employing advanced statistical models~~. This study has its own limitation, that is,~~, enhancing the robustness the findings~~ do~~.

The limitation of this study was many Sub-Saharan African countries have not ~~establish a cause and effect relationship due to the cross-sectional nature of the data/~~conducted DHS surveys ~~and~~since 2019, which may affect the representativeness of recent data. In addition, health- worker –related factors ~~could~~were not ~~be accounted for in this study.~~included in the analysis, potentially omitting important segments of population of healthcare access. Furthermore, future research better consider integrating objective measures, such as wearable devices, as demonstrated by Zhao et al. (2025)[42].

~~Conclusion and Recommendation~~

Formatted: Heading 1, Left

Formatted: Font color: Auto

Formatted: Normal

Formatted: Font: Bold

~~This~~Conclusions

In this study~~ found that 55.55%~~, over half of ~~reproductive age~~ women ~~had~~in Sub-Saharan Africa face barriers to ~~accessing~~ healthcare ~~in SSA~~access, emphasizing the need for focused strategies to improve equitable service delivery. Husband educational status, media exposure, internet utilization, place of residency, ANC visit status, place of delivery, health insurance utilization status, wealth index status, visiting health facility within 12 months, community literacy level and community wealth index and country category (~~western, eastern~~Western, Eastern Africa) were significantly associated with barriers of accessing health care. Therefore, the ~~Government~~governments and ~~ministry~~ministries of health in Sub-Saharan Africa countries ~~should~~better taking in to consideration those factors while designing policies and strategies targeting to health care access and ~~tackling~~talking the preventable barriers to ~~accessing health care~~healthcare access in sub-Saharan Africa.

Declaration

~~Ethical approval and consent to participate~~

~~This study was based on analysis of existing survey datasets in the public domain (SSA) that are freely available online with all the identifier information anonymized, no ethical approval was required~~

Consent for publication

Not applicable

Data and material availability

Demographic and Health Survey data were used, and ~~it is~~are publicly available online at (https://www.dhsprogram.com).

Competing interest

Authors Declared that, there is no competing interest.

Funding

Not applicable

Formatted: Font color: Auto
Formatted: Heading 1, Left
Formatted: Font: +Headings (Calibri Light), 16 pt
Formatted: Font: 16 pt, Font color: Auto
Formatted: Heading 2
Formatted: Font: 16 pt

Author's contributions

GAZ: involved in designing, data extraction, data analysis, interpretation, report and manuscript writing.

~~BT~~EGM: involved in data analysis, interpretation, and, and manuscript writing.

BT: involved in analysis, drafting manuscript and extract data

~~TTT~~MW: involved in designing the study, interpretation, report ~~and~~

~~BSW: involved in analysis, and result interpretation~~

~~AFZ: involved in designing and interpretation study~~

~~EGM: analysis and manuscript writing~~

~~MW: Result interpretation~~

AA: manuscript writing: involved in data extraction

AA: manuscript writing: involved in data extraction

AFZ: involved in designing and interpretation study

MSA: involved manuscript writing

~~Acknowledgements~~BT: involved, drafting manuscript and data extraction

TTT: involve analysis and manuscript editing

BSW: involved in analysis, and result interpretation

2Acknowledgements

We would like to say the DHS programs for allowing us to use the relevant DHS dataset in this study.

Reference

1. Tamirat, K.S., Z.T. Tessema, and F.B.J.B.H.S.R. Kebede, *Factors associated with the perceived barriers of health care access among reproductive-age women in Ethiopia: a secondary data analysis of 2016 Ethiopian demographic and health survey.* 2020. **20**: p. 1-8.

Formatted: Font: 16 pt, Font color: Auto

Formatted: Font: 16 pt

Formatted: Heading 2

Formatted: Font color: Auto

Formatted: Heading 1

Formatted: Font: Calibri, 11 pt

Formatted: Font: Calibri, 11 pt, Italic

Formatted: Font: Calibri, 11 pt

2. Erasmus, M.O., *The barriers to access for maternal health care amongst pregnant adolescents in the Mitchells plain sub-district.* 2017.
3. Washington, D.L., et al., *Access to care for women veterans: delayed healthcare and unmet need.* 2011. **26**: p. 655-661.
4. Regan, L.J.B.M.A., *Addressing unmet needs in global women's health.* 2018.
5. Izugbara, C., E. Covan, and E. Fugate-Whitlock, *Women's health in Africa: Issues, challenges and opportunities*. 2017: Routledge.
6. Organization, W.H., *Primary health care on the road to universal health coverage: 2019 global monitoring report: Executive summary*. 2019, World Health Organization.
7. Badiuzzaman, M., S.M. Murshed, and M.J.T.J.o.D.S. Rieger, *Improving maternal health care in a post conflict setting: evidence from Chittagong Hill tracts of Bangladesh.* 2020. **56**(2): p. 384-400.
8. Peters, D.H., et al., *Poverty and access to health care in developing countries.* 2008. **1136**(1): p. 161-171.
9. Alkire, B.C., et al., *The economic consequences of mortality amenable to high-quality health care in low-and middle-income countries.* 2018. **37**(6): p. 988-996.
10. Nyakang'o, S.B. and A.J.M. Booth, *Women's perceived barriers to giving birth in health facilities in rural Kenya: A qualitative evidence synthesis.* 2018. **67**: p. 1-11.
11. Colglazier, W.J.S., *Sustainable development agenda: 2030.* 2015. **349**(6252): p. 1048-1050.
12. Cahill, C.M., et al., *Preoperative anemia management program reduces blood transfusion in elective cardiac surgical patients, improving outcomes and decreasing hospital length of stay.* 2021. **61**(9): p. 2629-2636.
13. Peeri, N.C., et al., *The SARS, MERS and novel coronavirus (COVID-19) epidemics, the newest and biggest global health threats: what lessons have we learned?* 2020. **49**(3): p. 717-726.
14. Tessema, Z.T., et al., *Determinants of accessing healthcare in Sub-Saharan Africa: a mixed-effect analysis of recent Demographic and Health Surveys from 36 countries.* 2022. **12**(1): p. e054397.
15. Bintabara, D., K. Nakamura, and K.J.B.o. Seino, *Improving access to healthcare for women in Tanzania by addressing socioeconomic determinants and health insurance: a population-based cross-sectional survey.* 2018. **8**(9): p. e023013.
16. Kea, A.Z., et al., *Exploring barriers to the use of formal maternal health services and priority areas for action in Sidama zone, southern Ethiopia.* 2018. **18**: p. 1-12.

17. Zegeye, B., et al., *Breaking barriers to healthcare access: a multilevel analysis of individual-and community-level factors affecting women's access to healthcare services in Benin.* 2021. **18**(2): p. 750.

18. Silal, S.P., et al., *Exploring inequalities in access to and use of maternal health services in South Africa.* 2012. **12**: p. 1-12.

19. Tsawe, M. and A.S.J.B.r.n. Susuman, *Determinants of access to and use of maternal health care services in the Eastern Cape, South Africa: a quantitative and qualitative investigation.* 2014. **7**: p. 1-10.

20. Zegeye, B., et al., *Barriers and facilitators to accessing health care services among married women in Ethiopia: a multi-level analysis of the Ethiopia demographic and health survey.* 2021.

21. Demsash, A.W., B. Chakilu, and A. Mazengia, *Knowledge Sharing Practice and Its Associated Factors Among Healthcare Providers at University of Gondar Comprehensive Specialized Hospital, North West Ethiopian: Cross-sectional Study.* 2021.

22.22. Tsegaw, M., B. Mulat, and K.J.B.o. Shitu, *Problems with accessing healthcare and associated factors among reproductive-aged women in the Gambia using Gambia* demographic and Health Survey 2019/2020: a cross-sectional study. 2023. **13**(8): p. e073491.

23. Seidu, A.-A.J.P.O., *Mixed effects analysis of factors associated with barriers to accessing healthcare among women in sub-Saharan Africa: Insights from demographic and health surveys.* 2020. **15**(11): p. e0241409.

23. Tsegaw, M., B. Mulat, and K.J.B.o. Shitu, *Problems with accessing healthcare and associated factors among reproductive-aged women in the Gambia using Gambia Demographic and Health Survey 2019/2020: a cross-sectional study.* 2023. **13**(8): p. e073491.

24. Seidu, A.-A., et al., *Individual and contextual factors associated with barriers to accessing healthcare among women in Papua New Guinea: insights from a nationwide demographic and health survey.* 2021. **13**(6): p. 573-585.

25. Seidu, A.-A., et al., *Barriers to accessing healthcare among women in Ghana: a multilevel modelling.* 2020. **20**: p. 1-12.

26. Minyihun, A., Z.T.J.R.M. Tessema, and H. Policy, *Determinants of access to health care among women in East African countries: a multilevel analysis of recent demographic and health surveys from 2008 to 2017.* 2020: p. 1803-1813.

27. Sen, K.K., et al., *Do education and living standard matter in breaking barriers to healthcare access among women in Bangladesh?* 2023. **23**(1): p. 1431.

Formatted: Font: Calibri, 11 pt, Italic
Formatted: Font: Calibri, 11 pt
Formatted: Font: Calibri, 11 pt, Italic
Formatted: Font: Calibri, 11 pt
Formatted: Font: Calibri, 11 pt, Italic
Formatted: Font: Calibri, 11 pt
Formatted: Font: Calibri, 11 pt, Italic
Formatted: Font: Calibri, 11 pt
Formatted: Font: Calibri, 11 pt, Italic
Formatted: Font: Calibri, 11 pt
Formatted: Font: Calibri, 11 pt
Formatted: Font: Calibri, 11 pt, Italic
Formatted: Font: Calibri, 11 pt
Formatted: Font: Calibri, 11 pt, Italic
Formatted: Font: Calibri, 11 pt
Formatted: Font: Calibri, 11 pt
Formatted: Font: Calibri, 11 pt, Italic
Formatted: Font: Calibri, 11 pt
Formatted: Font: Calibri, 11 pt, Italic
Formatted: Font: Calibri, 11 pt
Formatted: Font: Calibri, 11 pt, Italic
Formatted: Font: Calibri, 11 pt
Formatted: Font: Calibri, 11 pt, Italic
Formatted: Font: Calibri, 11 pt
Formatted: Font: Calibri, 11 pt, Italic
Formatted: Font: Calibri, 11 pt

28. Dimbuene, Z.T., et al., *Women's education and utilization of maternal health services in Africa: a multi-country and socioeconomic status analysis.* 2018. **50**(6): p. 725-748.

29. Zegeye, B., et al., *Predictors of institutional delivery service utilization among women of reproductive age in Senegal: a population-based study.* 2021. **79**: p. 1-11.

30. Demsash, A.W., A.D.J.B.H. Walle, and C. Informatics, *Women's health service access and associated factors in Ethiopia: application of geographical information system and multilevel analysis.* 2023. **30**(1).

31. Tamang, T.M. *Factors associated with completion of continuum of Care for Maternal Health in Nepal*. in *IUSSP XXVIII International Population Conference, Cape Town, South Africa*. 2017.

32. Fentie, E.A., et al., *Spatial distribution and determinants of barriers of health care access among female youths in Ethiopia, a mixed effect and spatial analysis.* 2023. **13**(1): p. 21517.

33. Alamneh, T.S., et al., *Barriers for health care access affects maternal continuum of care utilization in Ethiopia; spatial analysis and generalized estimating equation.* 2022. **17**(4): p. e0266490.

34. Kalule-Sabiti, I., A.Y. Amoateng, and M.J.A.P.S. Ngake, *The effect of socio-demographic factors on the utilization of maternal health care services in Uganda.* 2014: p. 515-525.

35. Shiferaw, S., et al., *Why do women prefer home births in Ethiopia?* 2013. **13**: p. 1-10.

36. Yaya, S., G. Bishwajit, and V.J.B.g.h. Shah, *Wealth, education and urban–rural inequality and maternal healthcare service usage in Malawi.* 2016. **1**(2): p. e000085.

37. Voto, T.P., B.E. Voto, and N.J.E. Ngepah, *The Impact of Out-of-Pocket Health Expenditure and Public Health Expenditure on Poverty in Sub-Saharan Africa.* 2025. **13**(5): p. 134.

38. Alamneh, T.S., et al., *Socioeconomic inequality in barriers for accessing health care among married reproductive aged women in sub-Saharan African countries: a decomposition analysis.* 2022. **22**(1): p. 130.

39. AKINWALE, A.A.J.I.J.o.I.R. and A. Studies, *Gender equity and social progress: empowering women and girls to drive sustainable development in Sub–Saharan Africa.* 2023.

40. Arhin, K., E.F. Oteng-Abayie, and J.J.H.e.r. Novignon, *Assessing the efficiency of health systems in achieving the universal health coverage goal: evidence from Sub-Saharan Africa.* 2023. **13**(1): p. 25.

41. Muhajarine, N., et al., *Inequalities and factors associated with maternal healthcare services utilisation in Mozambique: evidence from the Demographic and Health Survey 2022– 2023.* 2025. **10**(5).

42. Zhao, Y., et al., *Highly sensitive, wearable piezoresistive methylcellulose/chitosan@ MXene aerogel sensor array for real-time monitoring of physiological signals of pilots.* 2025. **68**(2): p. 542-551.

Figure legend

Fig 1: Barriers to accessing health care and determinant factor among reproductive age women in sub-Saharan Africa

Supplementary file

S1. Table 5 Bivariable logistic regression of determinate factors associated with barriers to healthcare access among women in Sub-Saharan Africa.

Formatted: Font: Not Bold

Formatted: Space After: 0 pt

Formatted: Font: 12 pt

Formatted: Normal, Justified, Line spacing: 1.5 lines

Formatted: Font: Bold

Point by point response

Response to Editors and reviewers' comment

Manuscript ID: PONE-D-24-28999R2

Title: Barriers to healthcare access among women in sub-Saharan Africa: A pooled analysis of multi-country DHS data (2019-2023).

Journal: PLOS ONE

Authors: Gebreeyesus Abera Zeleke[1]*, Enyew Getaneh Mekonen[1], Bewuketu Terefe[2], Mulugeta Wassie[3], Agazhe Aemro[3], Alebachew Ferede Zegeye[3], Mohammed Seid Ali[4], Berhan Tekeba[4], Tadesse Tarik Tamir[4], Belayneh Shetie Workneh[5]

Subject: Submission of revised manuscript

Yours sincerely,

Gebreeyesus Abera Zeleke **(on behalf of all authors)**

University of Gondar, Gondar, Ethiopia

Response to editor comments

Comment #1 If the reviewer comments include a recommendation to cite specific previously published works, please review and evaluate these publications to determine whether they are relevant and should be cited. There is no requirement to cite these works unless the editor has indicated otherwise.

Response: Dear editor, we carefully revise, the publications suggested by the reviewer and assessed their relevance to the present study. As they were not directly aligned with the scope and objectives of our analysis, we did not include them, in line with the editorial guidance that citation is not mandatory unless explicitly required.

Comment #2 Please review your reference list to ensure that it is complete and correct. If you have cited papers that have been retracted, please include the rationale for doing so in the manuscript text, or remove these references and replace them with relevant current references. Any changes to the reference list should be mentioned in the rebuttal letter that accompanies your

revised manuscript. If you need to cite a retracted article, indicate the article's retracted status in the References list and also include a citation and full reference for the retraction notice

Response: Dear editor, thank you for your comment, the reference list has been carefully reviewed and verified for completeness and accuracy. None of the cited articles have been retracted; therefore, no retracted references are included in the manuscript.

Comment #3 This is an important study using globally accepted data from a number of African countries on an important topic of health care utilization. However, you the authors continue to fail to produce a well-written academic English manuscript, which cannot be understood by any English-speaking reader and does not allow it to be published. I am writing to you asking to PLEASE FIND SOMEBODY WHO WRITES PROPER ACADEMIC ENGLISH before submitting your article again. I have also with extreme detail suggested lots of improvements in the attached file. Also, you cannot use the Discussion to again put each result of each barrier identified and explain it in long detail. You have to find a way to SUMMARIZE the findings so that such section is not too long. I hope you will do that in your next submission, if you want the article published. Thank you.

Response: Dear editor, thank for the detailed and constructive feedback. The manuscript has undergone thorough English language editing to improve clarity, coherence, and readability throughout the manuscript. In addition, the Discussion section has been substantially revised to synthesize and summarize key findings thematically, avoiding repetition and excessive detail, in line with the recommendations provided.

<u>New editors' comment</u>

Comments, We note that there were multiple versions of Manuscript, Track changes, and response to reviewer in your submission's file inventory. We have removed the older file(s), retaining the most recent version(s) for editorial review. Please double check your submission file inventory and let us know if any files appear to be outdated or absent.
2. Please upload a copy of Supporting Information Table 5 which you refer to in your text on page 24. 3. Please ensure that you refer to Supporting Information Table 4 in your text as, if accepted, production will need this reference to link the reader to the Table.

Response; Dear editor, Thank you for your comment. We have reviewed the submission file inventory and confirmed that only the most recent versions of the manuscript and related files are included. Supporting Information Table 5 has now been uploaded as a Supporting Information file and cited in the Methods section, and Table 4 has been corrected to appear in the main manuscript and is now cited appropriately in the text as Table 4.

Reviewers comment to author

Reviewer three

Comment #1 The title is descriptive but could be made more precise, "multi-country DHS pooled analysis"

Response: Dear Reviewer**,** thank you for your feedback regarding the title. Accordingly, we enhance clarity of title. Kindly see correction on the title age line number 1& 2.

Comment #2 Avoid reporting contradictory numbers (e.g., 55.84 vs 55.55). Ensure one consistent prevalence figure with CI.

Response: Dear reviewer, Thank your careful review & valuable feedback. I apologize for the inconsistency in the reported numbers., I confirm that the correct prevalence figure is 55.84. kindly se amendment throughout manuscript.

Comment #3 Avoid reporting contradictory numbers (e.g., 55.84 vs 55.55). Ensure one consistent prevalence figure with CI.

Response: Dear reviewer, thank your careful review & valuable feedback. I apologize for the inconsistency in the reported numbers., I confirm that the correct prevalence figure is 55.84. kindly se amendment throughout manuscript.

Comment #4 The introduction effectively establishes the relevance of studying healthcare access barriers in Sub-Saharan Africa and aligns well with SDG and maternal health priorities, supported by appropriate global health literature. However, it over-relies on general SDG references and should clearly highlight gaps in previous DHS studies, summarize regional differences using 2019–2023 data.

Response: Dear reviewer**,** thank you for your crucial comment. We have revised the Introduction to highlight gaps in prior DHS studies, emphasizing the limited post-COVID-19 evidence on healthcare access, regional disparities, and intersectional demographics. This study uses recent DHS data (2019–2023) to address these gaps and inform health system recovery. Kindly see amendment on introduction section on page5 line number 118-122.

Comment#5 The Methods section is comprehensive, but it needs better organization and clarity. Please restructure for logical flow, clearly explain the construction of the composite “barrier to healthcare” variable, simplify technical details, and state the included countries earlier. Add a brief ethical statement noting secondary DHS data use. To ensure transparency and replicability, include a summary table of all DHS surveys (country, year, sample frame, survey code/version) and clarify whether the analysis covered all women aged 15–49 years or a specific subgroup.

Response: Dear reviewer**,** Thank you for your comment. We have revised the manuscript to clearly and explicitly describe the computation of outcome variable (barriers to healthcare access) in accordance with the DHS guidelines. kindly see amendment on method section on page line number 168-176.

Comment#6 Additionally, specify how missing data were handled.

Response: Dear Reviewer, thank you for this comment. Women with missing information on the outcome variables were retained in the denominator, consistent with DHS analytical guidelines. Kindly see amendment in method section on page 7-line number 176-177.

Comment #7 The Statistical Analysis section requires greater technical clarity. Explicitly report the exact commands used and clearly state whether survey weights were normalized. Provide a clear statistical justification for the final model selection, supported by model comparison results.

Response: Dear Reviewer, thank you for this comment. Stata commands used that DHS survey weights were normalized, and we justify final model selection. Dear reviewer kindly sees amendment techniques of weighting DHS data on page 6-line number 159-164 and justification for final model selection on age 12 -13-line number 257-272.

Comment #8 The Results section should be streamlined to progress from descriptive findings → model comparison → final determinants. present one concise model table reporting ORs, 95% CIs, p-values, and standard errors for all final predictors.

Response: Dear reviewer, Thank you for the constructive suggestions. We have revised the results section to improve clarity and logical flow, progressing from descriptive findings to model comparison and finally determinants in final multilevel regression model. In line with these recommendations, we now present a single concise table reporting adjusted odds ratios (ORs) and 95% confidence intervals for all predictors retained in the final model. Bivariable analyses were used solely for variable screening and are described in the Methods section; to enhance readability and avoid redundancy, the bivariable regression table has been removed from the main manuscript and is provided as Supplementary Material. Kindly see amendment in result section on page 10-18-line number 250-401.

Comment #9 The Discussion should minimize repetition, synthesize results thematically, and focus on explaining mechanisms (e.g., why socioeconomic, contextual, and care-utilization factors influence barriers), including regional differences between East and West Africa and comparisons with 3–5 recent studies. Emphasize interpretation over description and conclude with clear policy implications aligned with UHC, gender equity, and national health priorities**.**

Response: Dear Reviewer, we appreciate detail revision. The Discussion has been revised to reduce repetition, synthesize results thematically, focus on underlying mechanisms and regional differences, compare with recent studies, and emphasize policy implications aligned with UHC, gender equity, and national health priorities. Kindly see amendment in discussion section on page 16-18-line number 320-398.

<u>Reviewer four</u>

Comment #1 This should be Results, this sentence is not clear. Are these the main "barriers"?,

Response: Dear reviewer, Thank you for the comment. We have revised this sentence for clarity. Kindly see amendment on abstract result section on page 2-line number 47-50

Comment #2 "individually associated variables"? If so, what are the ones of the previous sentence? Are these "community associated variables"? [needs to be described better]

Response: Dear Reviewer, Thank you for the comment. We have clarified the terminology by explicitly defining “individual-level variables” and “community-level variables” in the text and clearly specifying which variables belong to each category in the revised manuscript. Kindly see amendment on page 2 linen number 51-57.

Comment #3 There is a need to include some form of theoretical model to explain how the independent variables were selected. You have put a few previous studies so could use those variables that were found in individual countries affecting health care services...

Response: Dear reviewer Thank you for pointing out this concern. The selection of independent variables was guided by the DHS conceptual framework on health service utilization, which classifies determinants into individual-level and community-level factors, and was further informed by evidence from prior DHS-based studies in sub-Saharan Africa. All variables included in the analysis were available in the DHS datasets and selected a priori based on their theoretical and empirical relevance to healthcare access.

Comment #4 " : " [colon, because you are adding the countries after stating it], and " , " [comma, as a pause before continuing]

Response: Dear reviewer, Thank you for the comment. We have revised the punctuation accordingly by using a colon to introduce the list of countries and a comma where a pause was required for clarity. Kindly reamendment on method section line number 143-145.

Comment #5 Not clear how they were selected and recoded. Rephrase again

Response: Dear reviewer, Barriers to healthcare access were constructed from four DHS variables (v467b, v467c, v467d, and v467f) in the maternal recode dataset. Women reporting at least one item as a ‘big problem’ were coded as having barriers. For farther clarification kindly see in method section outcome variable determination kindly on page 7-line number 167-176.

Comment#6 "as factor barriers", "barriers" [plural], "therefore", "weigh", "used"

Response: Dear reviewer, Thank you for the comment. The wording has been corrected to ‘barrier factors’/‘barriers’ (plural), ‘therefore,’ ‘weight,’ and ‘used’ in the revised manuscript. Kindly see correction on age 6-line number 152-159.

Comment #7 Why divided by 1 million? Needs to be explained.

Response: Dear reviewer, we divided the DHS sample weight variable (v005) by 1,000,000 to rescale it to its true value. In the DHS dataset, v005 is stored as an integer multiplied by 1,000,000 to avoid decimals. Dividing by 1,000,000 restores the correct weight for analysis and does not relate to the population size. This procedure follows the DHS guideline for using sampling weights.

Community#8 Why were these variables chosen? Based on previous studies..., etc.? There needs to be some form of theoretical model or using findings from previous studies...etc., that oriented you to use these variables. Otherwise, there is no justification and the reader get confused as to why they in particular were used! Also, these are not "Outcome" variables but more like Independent...can you explain better?

Response: Dear reviewer, thank you for your comment. The independent variables included in our study were selected based on prior research and the DHS conceptual framework for healthcare access. These variables have been consistently identified in previous studies as determinants of barriers to healthcare among women in Sub-Saharan Africa. We have clarified in the manuscript that these are independent variables, while the constructed 'barrier to healthcare access' variable is the outcome.

Comment #9 Please see the manuscript for grammatical consistency, including correct use of singular and plural verbs, spelling, and punctuation (e.g., colons, semicolons, and parentheses).

Response: We have carefully reviewed and revised the entire manuscript to ensure grammatical consistency, including correct use of singular and plural verb forms, spelling, and punctuation (e.g., colons, semicolons, and parentheses). All identified issues have been corrected accordingly. Kindly see amendment in method section line number 140-189.

Comment #10 singular and plural terms (e.g., outcome(s), barrier(s), variable(s)), grammatical errors, punctuation, and inappropriate terminology (e.g., patients vs. respondents) been addressed in the revised manuscript

Response: Dear reviewer, thank detail reviewing, we have carefully revised the manuscript to ensure consistent use of singular and plural terms, corrected grammatical and punctuation errors,

and standardized terminology throughout. Kindly see amendment method section line number 190-241

Comment #11 Patient and public involvement

Response: Dear reviewer, Thank you for the suggestion. The “Patient and Public Involvement” statement has been relocated to the appropriate section of the Methods. Kindly see correction, on age number 6-line number 138 to 139.

Comment #12 from 10 Sub-Saharan African countries", results, grammatical issue,

Response: Dear reviewer, thank detail reviewing, we have carefully revised the manuscript to ensure consistent use of singular and plural terms, corrected grammatical and punctuation errors, and standardized terminology throughout. Kindly see amendment result section line number 241-249.

Commment#13 Individual Level [need to add sub-title to differentiate the two types of variables], You can use one decimal instead of two, so the reading becomes easier... Status, Husband's Education, Secondary, Working, Single, Place of Delivery, Change to in bold, "Internet, Visited Health Facility Last 12 Months, Poor", "Middle" [capitals], Community Leve, No need to put the percentage signs here!

Response: Dear reviewer, thank the reviewer for these valuable and constructive suggestions. Accordingly, we have added appropriate subheadings to clearly differentiate the two types of individual-level variables, simplified the presentation by using one decimal place, corrected capitalization and bold formatting, standardized variable labels, and removed percentage signs where they were not required. These revisions have improved the clarity and readability of the table2. Kindly see correction in result section line number 249 to 252.

Comment #14 Need to explain better, e.g., "Using the Akaike Information Criteria, among... Not clear what this "fines" means...

Response: Dear reviewers, Thank you for this comment. We have revised the manuscript to clearly explain the use of the Akaike Information Criterion (AIC) for model comparison, including its purpose, definition, and the criterion used to select the final model (lowest AIC). Kindly see explanation in result section, line number 256 to 274.

Comment #15 barriers, reproductive age women, an urban area, level, " [a comma before "respectively"], it those living in East., "1.43)," [comma after the parenthesis, given the length of the sentence], visits, respectively" Use a semi-colon (;) because the sentence is already long and there is more to be said... "internet" "access, " [ALWAYS A COMMA BEFORE RESPECTIVELY!], ";" [semicolon], a health facility", Interesting that women who DID NOT visit a health facility had 9% times LESS barriers...? Will need to be analyzed carefully... change this. E.g., "Additionally, the odds...", "a perceived" community-level

Response: Dear reviewer, Thank you for the detailed editorial and interpretative comments. All suggested punctuation, capitalization, and formatting issues (including the consistent use of commas before "respectively", appropriate use of semicolons in long sentences, and wording clarifications) have been carefully corrected throughout the manuscript. Dear reviewer kindly see correction in result section line number 274 to 313.

Comment #16 changes in socioeconomic, health infrastructure, health system policies, women's attitudes towards health care seeking behavior, and cultural differences across countries in Sub-Saharan Africa." "Moreover, these discrepancies might be due to differences in the study period, because this study includes the most recent DHS data sets, from 2019 to 2023." his has already been mentioned before! reproductive age, Guinea, be due [no comma needed], infrastructures, i.e., inaccessibility to..., Sub-Saharan countries and other many punctuation error, grammatical, capitalization etc.

Response: Dear reviewer, Dear reviewer, we thank for these detail comment. Redundant statements regarding the study period and use of the most recent DHS datasets have been removed to improve conciseness. In addition, all identified issues related to grammar, punctuation, capitalization, and wording (including appropriate comma usage, consistency in terminology such as women of reproductive age, country names, and descriptions of health infrastructure) have been carefully corrected throughout the manuscript. These revisions have improved overall clarity and readability. Kindly see all amendment in discussion, strengthen and limitation conclusion section line number 318 to 411.

Comment #17 declared that they have no competing interests., Survey data, are; [semicolon, not comma]

Response: Dear reviewer, Thank you for the comment. The competing interest's statement has been revised to read "The authors declare that they have no competing interests." In addition, grammatical corrections have been made, including changing "Survey data is" to "Survey data are" and replacing commas with semicolons where appropriate**.** kindly see amendment line number 419-432.

Comment 18. THIS SEEMS TO BE THE SAME TABLE...IS IT DIFFERENT?

Responses: Thank you for the comment. We agree with the reviewer that the tables 5 were redundant. Therefore**,** Table 5 has been removed from the revised manuscript, and the relevant information is adequately presented in the remaining table. Kindly see amendment in manuscript.
