# Supplementary material for: Barriers to healthcare access among women in sub-Saharan Africa: A pooled analysis of multi-country DHS data (2019–2023)
Source: PLoS One. 2026 Feb 19;21(2):e0331328. doi: 10.1371/journal.pone.0331328 (PMC12919819; doi:10.1371/journal.pone.0331328)
Supplement: Attachment — Submitted filename: PBP LAST.docx [file pone.0331328.s004.docx]

PLOS ONE Decision: Revision required [PONE-D-24-28999] - [EMID:170305376cfa5b87]

| **PLOS ONE** | Tue, May 27, 6:19 AM (4 days ago) |  |      |
| --- | --- | --- | --- |
| **Response to Editor and reviewers' comments** ID: [PONE-D-24-28999] - [EMID:170305376cfa5b87] **Title:** Barriers to accessing health care among women in sub-Saharan Africa: Multilevel analysis of recent demographic and health surveys from 2019-2023. Journal: PLOS ONE **Name of Authors:** Gebreeyesus Abera Zeleke^1^*, Enyew Getaneh Mekonen^1^, Bewuketu Terefe^2^, Mulugeta Wassie^3^, Agazhe Aemro^3^, Alebachew Ferede Zegeye^3^, Mohammed Seid Ali^4^, Berhan Tekeba^4^, Tadesse Tarik Tamir^4^, Belayneh Shetie Workneh^5^. **Subject:** Submission of revised manuscript Yours sincerely Gebreeyesus Abera Zeleke (on behalf of all authors) university of Gondar, Gondar Ethiopia. **Response to Editors and reviewer comments**  | | |  |

Thank you for submitting your manuscript to PLOS ONE. After careful consideration, we feel that it has merit but does not fully meet PLOS ONE’s publication criteria as it currently stands. Therefore, we invite you to submit a revised version of the manuscript that addresses the points raised during the review process.

**Response**: Dear editor, thank you for considering my work in your esteemed journal, we truly appreciate your time and support throughout the editing and review process.

Journal Requirements:

When submitting your revision, we need you to address these additional requirements.

1. Please ensure that your manuscript meets PLOS ONE's style requirements, including those for file naming. The PLOS ONE style templates can be found at

**Response:** Dear editor, thank you for your guides. The authors ensured, the manuscript meets PLOS ONE's style requirements

2. Please provide a complete Data Availability Statement in the submission form, ensuring you include all necessary access information or a reason for why you are unable to make your data freely accessible. If your research concerns only data provided within your submission, please write "All data are in the manuscript and/or supporting information files" as your Data Availability Statement.

**Response**: Dear editor, thank you for your recommendation. The authors ensured that, all data are in the manuscript and/or supporting information files in submission form on which the data freely accessible

3. Please amend either the abstract on the online submission form (via Edit Submission) or the abstract in the manuscript so that they are identical.

**Response:** Dear editor, thank you for your comment. The authors ensured that, abstracts on the submission form and abstract in the manuscript was corrected to be identical. Dear editor Kindly see amendment on abstract section and online submission form.

**Additional Editor Comments**:
The present manuscript tackles a prominent and present-day public health concern barrier to accessing healthcare services among women of reproductive age within sub-Saharan Africa employing nationally representative DHS datasets and multilevel modeling. The study's objective is praiseworthy and has the potential to contribute to the development of policy. However, the manuscript necessitates substantial revision before publication.

**Response:** Dear editor, thank you for your comment. The authors ensured that the comment that raised by editor and reviewer properly fix.

**Reviewers' comments:
Reviewer's Responses to Questions**

1. Is the manuscript technically sound, and do the data support the conclusions?

The manuscript must describe a technically sound piece of scientific research with data that supports the conclusions. Experiments must have been conducted rigorously, with appropriate controls, replication, and sample sizes. The conclusions must be drawn appropriately based on the data presented.

Reviewer #1: Yes

Reviewer #2: Yes

**Response:** Dear reviewers', thank you for your constructive response

2. Has the statistical analysis been performed appropriately and rigorously?

Reviewer #1: No

Reviewer #2: No

**Response:** Dear reviewers', thank you for your comment. Statistical analysis was conducted appropriately and rigorously. Multilevel logistic regression was employed to account for the hierarchical nature of the data. Sampling weights, stratification, and clustering were incorporated to ensure nationally representative estimates. The analysis was performed using Stata version 14, and the results are presented with adjusted odds ratios and 95% confidence intervals. Model assumptions were also checked. Dear reviewer, kindly see on page 5-line number 119-123 and on page 6-line number 140-145 and on page number 8 and line number 182-205.

3. Have the authors made all data underlying the findings in their manuscript fully available?

The [PLOS Data policy](http://track.editorialmanager.com/CL0/http:%2F%2Fwww.plosone.org%2Fstatic%2Fpolicies.action%23sharing/1/010f019711e60f5e-e8fd2ade-fbb2-4277-a46c-0084adfe97b4-000000/1LON1Z_raTiuqx7L_AjtR-zFDAly6WzcTji-rHZdvlc=212) requires authors to make all data underlying the findings described in their manuscript fully available without restriction, with rare exception (please refer to the Data Availability Statement in the manuscript PDF file). The data should be provided as part of the manuscript or its supporting information, or deposited to a public repository. For example, in addition to summary statistics, the data points behind means, medians and variance measures should be available. If there are restrictions on publicly sharing data—e.g. participant privacy or use of data from a third party—those must be specified.

Reviewer #1: No

Reviewer #2: Yes

**Response:** Dear reviewers', thank you for your comment. The data were fully provided in the body manuscript and supporting information file.

4. Is the manuscript presented in an intelligible fashion and written in standard English?

PLOS ONE does not copyedit accepted manuscripts, so the language in submitted articles must be clear, correct, and unambiguous. Any typographical or grammatical errors should be corrected at revision, so please note any specific errors here.

Reviewer #1: No

Reviewer #2: No

**Response:** Dear Reviewers, thank you for your comments. We, the authors, have revised the manuscript by addressing the language and grammatical issues through careful paraphrasing and editing.

**Review Comments to the Author**

Please use the space provided to explain your answers to the questions above. You may also include additional comments for the author, including concerns about dual publication, research ethics, or publication ethics. (Please upload your review as an attachment if it exceeds 20,000 characters)

**Reviewer #1:** This manuscript tackles an important public-health issue—identifying barriers to healthcare access among reproductive-age women in sub-Saharan Africa (SSA) using pooled DHS data from 2019–2023. The large sample size (N≈134,000), the inclusion of both individual- and community-level variables, and the application of multilevel mixed-effects logistic regression all strengthen its potential impact. However, before this work can be considered for publication, several critical concerns must be addressed:

**Response:** Dear Reviewer, thank you for your thoughtful and encouraging feedback. We acknowledge that several critical concerns must be addressed, and we are committed to revising the manuscript accordingly. Detailed responses are provided for each point to enhance the clarity, rigor, and overall quality of the manuscript.

**Comment 1.** The manuscript does not explain how DHS sampling weights, clustering, and stratification were incorporated (e.g., in Stata’s svy: framework or via robust standard e the issue.rrors). Please specify your approach in the Methods.

**Response**: Dear Reviewer, thank you for the valuable comment. Since DHS data are **nationally representative,** stratified **multistage cluster sampling is required; therefor, sampling weights** must be applied to produce unbiased estimates. To correctly weight DHS data, we use the sampling weights provided in the dataset, specifically the **individual weight (**v005**)** for women’s individual recode files. This weight must be divided by **1,000,000** (v005/1000000). using “gen wgt=v005/1000000 Stata commands. **Clustering and stratification** are key components of the complex sampling design used in DHS surveys to ensure national representativeness and statistical efficiency. In DHS, **clustering** refers to the selection of **enumeration areas (EAs) while Stratification** involves dividing the population into distinct **subgroups (strata)** before sampling. In DHS, stratification is usually based on **geographic regions** (like provinces or states) and **urban/rural** residence. Dear reviewer, please see amendment on page 6-line number 141-146.

**Comment 2.** Multicollinearity is mentioned but actual VIF values are absent. Provide VIFs for all predictors. Include goodness-of-fit metrics (AIC, ∆Deviance) and a brief discussion of residual or influence diagnostics.

**Response**: Dear Reviewer, thank you for your insightful comment, we have added VIF values for all predictors (all <5), indicating no multicollinearity and used and AIC to compare multilevel models with varying fixed and random effects structures. The model including random intercepts and slopes (Model III) had the lowest AIC (AIC =239608.6), indicating the best balance between model fit and parsimony. kindly see amendment on age 9-line number 210-211 and on page 12-line number 245-246. And for farther information see table under supporting information file.

**Comment 3.** Several reported CIs are inverted (e.g., AOR=1.23, 95% CI: 1.30–1.15). Please correct all interval bounds and verify consistency across text, tables, and figures.

**Response:** Dear reviewer thank you for pointing this. We have reviewed all reported confidence intervals across the text, tables, and figures and corrected any inverted bounds to ensure proper formatting (e.g., lower bound listed first, followed by the upper bound). All intervals have been verified for consistency and accuracy throughout the manuscript. Thank you again.

**Comment 4.** I recommend citing Zhao et al. (2025) “Applied statistical methods for identifying features of heart rate that are associated with nicotine vaping”. Although the outcome differs, that paper presents a robust five‐step workflow. Suggest add “The five‐step workflow for extracting event‐related features from physiological time‐series data, originally developed by Zhao et al, inspired our adaptation of mixed‐effects modeling to detect ‘before/after’ patterns around reported access barriers.”

**Response:** Dear reviewer thank you for the valuable suggestion. We appreciate the recommendation to cite Zhao et al. (2025). Although our outcome of interest differs, we agree that their five-step workflow offers a robust and generalizable framework for extracting features from physiological time-series data.

**Comment 5**. The manuscript contains numerous grammatical errors and non-standard expressions (e.g., “not beg problem,” “were significantly contribute”). Engage a native English speaker or professional editor to ensure clarity.

**Response: Dear Reviewer,** thank you for comment about grammatical issues and non-standard expressions in the manuscript. We apologize for these errors and appreciate your careful and detail review of our manuscript. We have thoroughly revised the manuscript to correct these issues. Specifically, we have rephrased problematic expressions such as “not beg problem” and “were significantly contribute” to standard and grammatically correct forms. Additionally, we have carefully proofread the entire manuscript. Kindly see amendment on page number 8-line number 157-163 and page number 16-line number line number 296-299.

**Comment 6**. Tables are overly dense (Table 4 in particular). Consider splitting key results into separate tables or adding a forest‐plot figure for the main AORs. Once these issues are adequately addressed, the manuscript will be much stronger and ready for reevaluation.

**Response:** **Dear Reviewer,** thank you for your helpful suggestion regarding the presentation of results in Table 4. We the author, agree that the original table was overly dense and may have hindered readability. There for we splitting key results into separate tables. Dear reviewer, please kindly see amendment on the page number 15-16-line number 285-289.

**Reviewer #2**:

The present manuscript addresses a salient and contemporary public health concern barrier to accessing healthcare services among women of reproductive age in sub-Saharan Africa utilizing nationally representative DHS datasets and multilevel modeling. The study's objective is praiseworthy and has the potential to contribute to the development of policy. However, in its current state, the manuscript necessitates substantial revision (major) before it can be considered for publication. The following section delineates the issues that should be addressed.
**Comment 1.** The document under review contains a multitude of grammatical inaccuracies, cumbersome phrasing, and a lack of consistent terminology. These issues often hinder the reader's ability to comprehend the original message. It is imperative that all documents undergo a thorough revision.

**Response: Dear reviewer,** Thank you for your valuable feedback. We acknowledge the concern raised about grammatical, inaccuracies and inconsistent terminology. To address this issue, we have performed entire manuscript revision. clarity, coherence, and consistency have been improved. The revised version has been thoroughly edited for grammar and style, and we have ensured uniform use of terminology throughout. We believe these changes have significantly enhanced the readability and overall quality of the document.

**Comment 2.** It is imperative that all statistical estimates undergo a thorough review to ensure internal consistency and coherence.

**Response: Dear reviewer,** Thank you for your valuable comment. We have carefully reviewed all statistical estimates presented in the manuscript to ensure internal consistency and coherence. Cross-checks were performed between the results reported in the tables, figures, and text to confirm accuracy and alignment and manuscript has been updated accordingly.
**comment 3.** The selection of these specific countries is not accompanied by a clear justification, nor is there an explanation for why other countries were excluded (DHS data from 10 sub-Saharan African countries). A rationale must be provided for the inclusion criteria, and the findings must be explicated in terms of their generalizability to the entire SSA region.

**Response**: Dear reviewer, thanks very much and appreciates your valuable comment. The selection of countries was based primarily on the availability of recent, comprehensive, and comparable Demographic and Health Survey (DHS) data relevant to the study variables. While all sub-Saharan African countries with DHS data were initially considered, the focus was narrowed to 10 countries that met specific criteria namely, data completeness and survey recency. Only countries with fully available DHS data collected between 2019 and 2023 were included. The study draws on data from 134,470 women of reproductive age across diverse regions, enhancing the generalizability and contextual relevance of the findings within sub-Saharan Africa.

**Comment 4.** The definition of the binary outcome "barrier to accessing health care" is not sufficiently explained. The coding scheme and threshold for categorization are not clearly delineated. It is imperative that further elucidation be provided.

**Response: Dear reviewer,** thank you for your valuable comment, we acknowledge that the definition and coding of the binary outcome variable “barrier to accessing health care” were not sufficiently detailed in the original manuscript. We have now revised the Methods section to provide a clearer explanation. Specifically, the binary outcome was constructed based on responses to the following DHS questions: **"Getting permission to** **go to the doctor?" "Getting money needed for treatment? "Distance to health facility?" "Not wanting to go alone?"** Each of these questions had response options such as a big problem or not a problem. In our analysis, women who reported at least one **big problem** were coded as **having a barrier** to healthcare access (1), and those who reported **none of the four as** were coded as **having no barrier (0).** This approach is consistent with prior literature using DHS data. The coding scheme and threshold for categorization have now been explicitly stated in the manuscript to improve clarity and reproducibility. Dear reviewer, leas kindly see amendment on method section line number 149-161.

**Comment 5.** The multilevel mixed-effects logistic regression model is a methodologically sound approach; however, the following issues must be addressed: The presence of inconsistent or implausible AORs and confidence intervals is a concern. The section comparing models is not sufficiently clear. An analysis of the data reveals an overlap and redundancy between text, tables, and figures. It is imperative that all model outputs are thoroughly reviewed and that any transcription errors are rectified. The explanation of random effects statistics must be improved. It is recommended that Figures 1 and 2 be removed or merged into a more informative graphical representation.

**Response: Dear reviewer,** thank you for your constructive comments. We acknowledge the issue and have conducted a thorough revision all AORs and their corresponding confidence intervals. Additionally, we re-ran the regression to confirm the accuracy and consistency of all estimates reported in the manuscript. We have revised the section comparing the multilevel models to enhance clarity. Specifically, we now clearly describe the sequence of model building (null model, random intercept model, full model), the criteria used for model selection (AIC, log-likelihood, and deviance), and the rationale for choosing the final model. A table summarizing model fit statistics has been added to support this comparison. The explanation of random effects has been expanded. We now clearly describe the intraclass correlation coefficient (ICC), variance components, and their interpretation in the context of the multilevel model. This includes a brief explanation of the importance of accounting for cluster-level variability and the added value of the multilevel approach. We believe these revisions address your concerns and improve the overall clarity, accuracy, and presentation quality of the manuscript. All relevant changes have been tracked in the revised document for ease of review.

**comment 6.** The discourse is predominantly descriptive, exhibiting a paucity of depth and analytical rigor. It is imperative that the discussion be expanded to encompass the mechanisms underpinning the observed associations, as well as their policy ramifications. It is imperative to differentiate distinctly between individual, household, and system-level barriers.

**Response: Dear reviewer,** thank you for this important concern. As per your recommendation, we improve analytical depth of the discussion and tries to give conceptual clarity. The author revised the Discussion section to move beyond a descriptive summary of findings. Detailed exploration of the possible mechanisms of associations. In addition, we highlight the need for integrated interventions that address both demand- and supply-side barriers and we recommend targeted strategies, such as strengthening community-based health education, improving transportation infrastructure, and expanding health insurance coverage to reduce inequities in access. Dear reviewer please kindly see the amendment in discussion section on page 17-18-line number 303-374.

**Comment: 7**. The added value of this study is not articulated with sufficient clarity.

**Response: Dear reviewer,** thank you for the insightful comment. We have revised the manuscript to more clearly articulate the added value of this study. Kindly see amendment on introduction section on Page 5-line number 118-129.

**Comment 8.** It is imperative that the keywords, table, and reference formatting be corrected.

**Response: Dear reviewer, thank you** for your impactful comment. We have thoroughly reviewed and corrected the formatting of the keywords, tables, and references to ensure consistency with the journal’s guidelines.
