# Supplementary material for: Barriers to healthcare access among women in sub-Saharan Africa: A pooled analysis of multi-country DHS data (2019–2023)
Source: PLoS One. 2026 Feb 19;21(2):e0331328. doi: 10.1371/journal.pone.0331328 (PMC12919819; doi:10.1371/journal.pone.0331328)
Supplement: Attachment — Submitted filename: Point by point Last.docx [file pone.0331328.s007.docx]

**Response to reviewers' comments**

PONE-D-24-28999R1

**Title: Barriers to accessing health care among women’s in Sub-Saharan Africa: Multilevel Analysis of Recent Demographic and Health Survey from 2019-2023.**

**Journal:** PLOS ONE

**Authors:** Gebreeyesus Abera Zeleke^1^*, Enyew Getaneh Mekonen^1^, Bewuketu Terefe^2^, Mulugeta Wassie^3^, Agazhe Aemro^3^, Alebachew Ferede Zegeye^3^, Mohammed Seid Ali^4^, Berhan Tekeba^4^, Tadesse Tarik Tamir^4^, Belayneh Shetie Workneh^5^

**Subject:** Submission of revised manuscript

Yours sincerely,

**Gebreeyesus Abera Zeleke (on behalf of all authors)**

University of Gondar, Gondar, Ethiopia

**Comments from PLOS Editorial**

**Comment**: We note that one or more reviewers has recommended that you cite specific previously published works. As always, we recommend that you please review and evaluate the requested works to determine whether they are relevant and should be cited. It is not a requirement to cite these works. We appreciate your attention to this request.

**Response:** Dear editor, we sincerely thank you for forwarding the reviewers’ suggestions regarding additional references. We have carefully revised the recommended works and evaluated their relevance to our study. Accordingly, we have incorporated those that are directly pertinent and contribute meaningfully to the context of our manuscript.

**Reviewers' comments:
Reviewer's Responses to Questions**

**Response to Editors and Reviewer’s Comment**

We are sincerely grateful to the editor and reviewers for their thoughtful and constructive feedback on our manuscript. The comments are both encouraging and insightful, and we greatly appreciate the recognition of the clinical importance of our study and its findings. Please find below our detailed responses to the reviewers’ comments, along with the revised manuscript (clean version) and a version with tracked changes, submitted separately. We sincerely appreciate the reviewers’ time and effort, and we trust that the revisions have addressed the concerns raised and have further strengthened the quality of the manuscript.

**Reviewer #1**

**Comment 1:** The manuscript is significantly improved, and the majority of my initial concerns have been comprehensively addressed. However, one minor point remains. In the previous review, I suggested a citation that the authors acknowledged as valuable, but it has not yet been incorporated into the manuscript's body or reference list.

To potentially strengthen the discussion on limitations and future directions, the authors might consider the following point. The current analysis is comprehensive but relies entirely on self-reported data, which can be subject to recall bias. A forward-looking addition to the discussion could acknowledge this and suggest how future studies might overcome this limitation. For example, research could incorporate objective measures from technologies like wearable devices to assess certain behaviors or states. The study by Zhao et al. (2025) could be cited as an excellent example here, as it demonstrates a complete workflow for processing such complex physiological signals. Ultimately, this is just a suggestion for the authors' consideration, and the decision to incorporate this perspective rests with them

**Response:** Dear reviewer, we sincerely thank you for constructive follow-up comment and valuable suggestion. We regret the oversight in not incorporating the recommended citation in our previous revision. We have now addressed the possible drawbacks of depending only on self-reported data in the updated manuscript, emphasizing the possibility of recall bias. We have also recognized that objective measurements, including data from wearable technology, could be incorporated into future studies to supplement self-reported evaluations. The recommended citation to Zhao et al. (2025), which offers applying physiological signal processing in this situation, has also been included to support this section. Please kindly see the amendment on limitation and recommendation of manuscript on page 22 and 23.

**Reviewer #3: Major revisions are necessary, particularly with respect to the clarity of the statistical analyses and the presentation of the data.**

**Comment 1:** Line 197: In the health survey data, if a household-level component exists in addition to the individual and community levels, it should usually be considered in the analysis. Are most households contribute to single observations as well as the household variance is non-negligible, and random effects are weak/unidentified? The reason(s) should be explicitly stated.

**Response:** Dear reviewer, we appreciate your thoughtful comment on our analysis's inclusion of the household-level component. We concur that in order to get reliable estimates and prevent bias from unmodeled clustering effects, it is essential to consider the hierarchical structure of the data. The household level was not included in the final model since most household contributed one individual and the variance at the household level was insignificant in the preliminary models.

**Comment 2:** Line 196 -198: The variables included in Model I, Model II, and Model III are to be stated. Alternatively, the table footnotes should indicate the variables that correspond to each model.

**Response:** Dear Reviewer, we appreciate your careful reviewing to the clarity of our model presentation, particularly regarding the variables included in Models I, II, and III. we have revised the manuscript to variables included in each model in the text. Additionally, we have updated the footnotes of **table 4** to clearly indicate the variables corresponding to each model for ease of reference. These changes enhance transparency and improve the interpretability of our findings. Please kindly see amendment on page number 9-line number 196-212 and kindly refer **table 4** footnote.

**Comment 3**: Line 211- 215: The notations ‘VC’, ‘V’, 'Variance' should be replaced with the appropriate statistical symbols. Variance null, and the variance model is to be used

**Response:** Dear Reviewer, we sincerely thank you for this constructive comment. We agree that using standard statistical notation improves clarity. Accordingly, we have replaced all instances of ‘VC’ ‘V’ ‘Variance’ in to formulas that reflect the standard notation. We appreciate the reviewer’s suggestion, which has strengthened the Methods section. Please kindly see amendment on page 9- and 10-line number 221-225.

**Comment 4:** Line 214: The standard formula for MOR and not the shortcut version is to be presented.

**Response:** Dear Reviewer, thank for pointing this out. We agree that presenting the **standard formula** for the Median Odds Ratio (MOR) improves clarity and transparency. Accordingly, we have replaced the previous shortcut version with the standard formula. Please see amendment on page 9 and Line number 221- 223.

**Comment 5:** Line 217- 218: The sentence ‘The fixed effects were employed to calculate the correlation between the probability of barriers to obtaining health care access and independent factors at the individual and community levels’ is incorrect and requires revision.

**Response:** Dear reviewer, we appreciate bringing this constructive comment. Since fixed effects in multilevel models estimate the relationship between independent variables and the outcome rather than computing correlations, we agree that the original sentence was incorrect. The sentence has been changed to the associations between independent factors at the individual and community levels and barriers to obtaining health care access were estimated using the fixed effects. Please kindly see the amendment on page 10-line number 227-228.

**Comment 6:** Line 223 – 225: The sentence is to be written as a method, not mentioning the results in the method section.

**Response:** Dear reviewer, thank you for pointing this out. We have revised the sentence to describe the methodology without including the results, in accordance with standard reporting practices. Please kindly see amendment on page 10-line number 231-235.

**Comment 7:** Line 231: The variance figures can be omitted.

**Response**: Dear reviewer, we appreciate the for your valuable comment. In line with the suggestion, we have omitted the variance figures from the results section. Kindly see the amendment on result section on page 10-line number 241.

**Comment 8:** Line 232: The symbol percentage for 69.8 is missing

**Response:** Dear reviewer, thank you for noting this. We have corrected the value to 69.8% at line 232. Kindly see amendment on result section on Page 10-line number 241.

**Comment 9:** Table 2: The figures presented should be standardized, e.g., consistent use of commas or decimal points. The same applies to Table 3.

**Response:** Dear reviewer, thank you for this observation. We have standardized the numerical formatting in Tables 2 and 3 to ensure consistency, including the uniform use of decimal and commas. Kindly see the amendment on table 2 on page 10-12-line number 246-248 and table 3 on page 13-line number 270-271.
**Comment** 10**:** Line 244: Typo ‘of.3784967’. The p-value cannot be zero (to use symbol p <)

**Response:** Dear reviewer, thank you for pointing this out. We have corrected the typo and revised the reporting of the p-value to ‘p < 0.001’ to ensure accuracy and consistency with statistical reporting standards. Please kindly see amendment on page 13-line number 254-255.

**Comment** 11: Line 263-294: It was not clear whether the data written based on Table 4, Table 5, or both. A separate write-up should be provided for each table (Table 4 referring to the association, while Table 5 refers to the determinants of barriers). It is also suggested that the manuscript be proofread thoroughly. Also, please include the crude analyses table prior to adjustment.

**Response:** Dear reviewer, we sincerely appreciate your constructive comment. We acknowledge that the presentation of the results between Table 4 and Table 5 was not sufficiently clear. To address this, we revised the results section with a distinct write-up for Table 4 and removed Table 5, as it introduced confusion and redundancy. In line with your recommendation, we also added a new table presenting the crude odds ratios (unadjusted analyses) prior to adjustment. The revised version can be found in the updated Tables 4 and 5 (pages 14 and 17–18), with corresponding introductory paragraphs provided in the results section (lines 277–283 and 285–319).

**Comment 12:** Table 4 & Table 5: The title not clear. Need to mention clearly or denote how the variables in Table 4 & 5 were selected.

**Response:** Dear Reviewer, we sincerely thank you for this insightful comment. We acknowledge the concern regarding the clarity of the titles for Tables 4 and 5. Upon further review, we recognized that Table 5 was redundant with Table 4 and, to avoid confusion, we have removed it from the manuscript. We have also revised the title of Table 4 to clearly indicate how the variables were selected and presented.
**Comment 13**: Line 252-253: The sentence requires revision.
**Response:** Dear reviewer, thank you for this valuable suggestion. We have revised the sentence for clarity. The corrected version now reads: ‘Model comparison using Akaike’s Information Criterion (AIC) indicated that Model III achieved the best fit, with the lowest AIC value (239,608.6), substantially lower than the null model (AIC = 1,128,437) and Model II (AIC = 1,127,704). Please kindly see amendment on page 13-line number 262-265.
**Comment 14**: Line 272: In Table 4, there was no data/stem indicating higher than secondary education.

**Response:** Dear Reviewer, we sincerely thank for this observation. The apparent absence of higher than secondary education" in Table 4 is due to the fact that this category was used as the reference group in the regression analysis. Consequently, it was not displayed in the table with an odds ratio, as is standard practice. We have now clarified this in the table and in the results section to avoid any confusion. Please Kindly see amendment on table 4 on page 15 and Sociodemographic result table on page 11.
**Comment 15**: Line 273: No information on Table 5 for AOR=1.21, 95% CI: (1.31, 1.41).

**Response:** Dear reviewer; We appreciate you detail revision of the manuscript. Upon your direction, we remove the above data that is AOR=1.21, 95% CI: (1.31, 1.41) from Table 5 as well table 4. Because this number was introduced by mistake. This number was not available neither table 5 Nor table 4. Please kindly see the correct version the manuscript on page 14-line number 280-283.

**Comment 16:** Line 278: Typo 1.07 (based on Table 5).

**Response:** Dear reviewer, we sincerely thank you for carefully and detail revision. Upon re-examining the manuscript, we found that the value “1.07” was introduced incorrectly and does not appear in either Table 4 or Table 5. We have removed this erroneous value to avoid confusion and have revised the text accordingly. Please kindly see the corrected version on page 14-line number 285-287.
**comment 17:** Line 281: Typo 1.57 (based on Table 4). In Table 5, there was no data on internet utilization.

**Response:** Dear reviewer, we thank you for careful and detail reviewing of the manuscript. We re-checked the results and confirmed that the value “1.57” was mistakenly introduced. Internet utilization was not a significant variable and does not appear in Table 5. The incorrect value has now been removed, and the text has been revised to align with the data presented in Table 4.

**Comment 18:** Line 293: Table 4 & Table 5: East Africa is to be used as reference.
**Response:** Dear reviewer, we appreciate detail and insightful reviewing. We agree that using East Africa consistently as the reference category improves clarity. In the revised manuscript, we have specified East Africa as the reference group in Table 4. To avoid redundancy and potential confusion, we have removed it from Table 5, since the reference category is already defined in Table 4 and does not need to be repeated. We believe this adjustment enhances the accuracy and readability of the results. Please kindly see corrected version of manuscript on page number 16.  **comment 19:** The references do not conform to the journal’s required format.

**Response:** Dear reviewer, we sincerely thank for pointing out the references did not conform to the journal’s required format. We have carefully revised all references and reformatted them according to the PLOS ONE reference style guidelines. We double-checked both the in-text citations and the reference list to ensure full consistency with the journal’s requirements. Please kindly see amendment in reference section on page 23-25-line number 459-564.
