# Supplementary material for: Barriers to healthcare access among women in sub-Saharan Africa: A pooled analysis of multi-country DHS data (2019–2023)
Source: PLoS One. 2026 Feb 19;21(2):e0331328. doi: 10.1371/journal.pone.0331328 (PMC12919819; doi:10.1371/journal.pone.0331328)
Supplement: Attachment — Submitted filename: Revision 2 PBP Barriers.docx [file pone.0331328.s009.docx]

**Point by point response**

**Response to Editors and reviewers' comment**

**Manuscript ID:** PONE-D-24-28999R2

**Title:** Barriers to healthcare access among women in sub-Saharan Africa: A pooled analysis of multi-country DHS data (2019-2023).

**Journal:** PLOS ONE

**Authors:** Gebreeyesus Abera Zeleke^1^*, Enyew Getaneh Mekonen^1^, Bewuketu Terefe^2^, Mulugeta Wassie^3^, Agazhe Aemro^3^, Alebachew Ferede Zegeye^3^, Mohammed Seid Ali^4^, Berhan Tekeba^4^, Tadesse Tarik Tamir^4^, Belayneh Shetie Workneh^5^

**Subject:** Submission of revised manuscript

Yours sincerely,

Gebreeyesus Abera Zeleke **(on behalf of all authors)**

University of Gondar, Gondar, Ethiopia

**Response to editor comments**

**Comment #1** If the reviewer comments include a recommendation to cite specific previously published works, please review and evaluate these publications to determine whether they are relevant and should be cited. There is no requirement to cite these works unless the editor has indicated otherwise.

**Response:** Dear editor, we carefully revise, the publications suggested by the reviewer and assessed their relevance to the present study. As they were not directly aligned with the scope and objectives of our analysis, we did not include them, in line with the editorial guidance that citation is not mandatory unless explicitly required.

**Comment #2** Please review your reference list to ensure that it is complete and correct. If you have cited papers that have been retracted, please include the rationale for doing so in the manuscript text, or remove these references and replace them with relevant current references. Any changes to the reference list should be mentioned in the rebuttal letter that accompanies your revised manuscript. If you need to cite a retracted article, indicate the article’s retracted status in the References list and also include a citation and full reference for the retraction notice

**Response:** Dear editor, thank you for your comment, the reference list has been carefully reviewed and verified for completeness and accuracy. None of the cited articles have been retracted; therefore, no retracted references are included in the manuscript.

**Comment #3 T**his is an important study using globally accepted data from a number of African countries on an important topic of health care utilization. However, you the authors continue to fail to produce a well-written academic English manuscript, which cannot be understood by any English-speaking reader and does not allow it to be published. I am writing to you asking to PLEASE FIND SOMEBODY WHO WRITES PROPER ACADEMIC ENGLISH before submitting your article again. I have also with extreme detail suggested lots of improvements in the attached file. Also, you cannot use the Discussion to again put each result of each barrier identified and explain it in long detail. You have to find a way to SUMMARIZE the findings so that such section is not too long. I hope you will do that in your next submission, if you want the article published. Thank you.

**Response:** Dear editor, thank for the detailed and constructive feedback. The manuscript has undergone thorough English language editing to improve clarity, coherence, and readability throughout the manuscript. In addition, the Discussion section has been substantially revised to synthesize and summarize key findings thematically, avoiding repetition and excessive detail, in line with the recommendations provided.

**New editors’ comment**

**Comments,**  We note that there were multiple versions of Manuscript, Track changes, and response to reviewer in your submission's file inventory. We have removed the older file(s), retaining the most recent version(s) for editorial review. Please double check your submission file inventory and let us know if any files appear to be outdated or absent.
2. Please upload a copy of Supporting Information Table 5 which you refer to in your text on page 24. 3. Please ensure that you refer to Supporting Information Table 4 in your text as, if accepted, production will need this reference to link the reader to the Table.

**Response;** Dear editor, Thank you for your comment. We have reviewed the submission file inventory and confirmed that only the most recent versions of the manuscript and related files are included. Supporting Information Table 5 has now been uploaded as a Supporting Information file and cited in the Methods section, and Table 4 has been corrected to appear in the main manuscript and is now cited appropriately in the text as Table 4.

***Reviewers comment to author***

**Reviewer three**

**Comment #1** The title is descriptive but could be made more precise, “multi-country DHS pooled analysis”

**Res**p**onse: Dear Reviewer,** thank you for your feedback regarding the title. Accordingly, we enhance clarity of title. Kindly see correction on the title age line number 1& 2.

**Comment #2** Avoid reporting contradictory numbers (e.g., 55.84 vs 55.55). Ensure one consistent prevalence figure with CI.

**Response: Dear reviewer,** Thank your careful review & valuable feedback. I apologize for the inconsistency in the reported numbers., I confirm that the correct prevalence figure is 55.84. kindly se amendment throughout manuscript.

**Comment #3** Avoid reporting contradictory numbers (e.g., 55.84 vs 55.55). Ensure one consistent prevalence figure with CI.

**Response: Dear reviewer,** thank your careful review & valuable feedback. I apologize for the inconsistency in the reported numbers., I confirm that the correct prevalence figure is 55.84. kindly se amendment throughout manuscript.

**Comment #4** The introduction effectively establishes the relevance of studying healthcare access barriers in Sub-Saharan Africa and aligns well with SDG and maternal health priorities, supported by appropriate global health literature. However, it over-relies on general SDG references and should clearly highlight gaps in previous DHS studies, summarize regional differences using 2019–2023 data.

**Response: Dear reviewer,** thank you for your crucial comment. We have revised the Introduction to highlight gaps in prior DHS studies, emphasizing the limited post-COVID-19 evidence on healthcare access, regional disparities, and intersectional demographics. This study uses recent DHS data (2019–2023) to address these gaps and inform health system recovery. Kindly see amendment on introduction section on page5 line number 118-122.

**Comment#5** The Methods section is comprehensive, but it needs better organization and clarity. Please restructure for logical flow, clearly explain the construction of the composite “barrier to healthcare” variable, simplify technical details, and state the included countries earlier. Add a brief ethical statement noting secondary DHS data use. To ensure transparency and replicability, include a summary table of all DHS surveys (country, year, sample frame, survey code/version) and clarify whether the analysis covered all women aged 15–49 years or a specific subgroup.

**Response: Dear reviewer,** Thank you for your comment. We have revised the manuscript to clearly and explicitly describe the computation of outcome variable (barriers to healthcare access) in accordance with the DHS guidelines. kindly see amendment on method section on page line number 168-176.

**Comment#6** Additionally, specify how missing data were handled.

**Response: Dear Reviewer,** thank you for this comment. Women with missing information on the outcome variables were retained in the denominator, consistent with DHS analytical guidelines. Kindly see amendment in method section on page 7-line number 176-177.

**Comment #**7 The Statistical Analysis section requires greater technical clarity. Explicitly report the exact commands used and clearly state whether survey weights were normalized. Provide a clear statistical justification for the final model selection, supported by model comparison results.

**Response: Dear Reviewer,** thank you for this comment. Stata commands used that DHS survey weights were normalized, and we justify final model selection. Dear reviewer kindly sees amendment techniques of weighting DHS data on page 6-line number 159-164 and justification for final model selection on age 12 -13-line number 257-272.

**Comment #8** The Results section should be streamlined to progress from descriptive findings → model comparison → final determinants. present one concise model table reporting ORs, 95% CIs, p-values, and standard errors for all final predictors.

**Response:** Dear reviewer, Thank you for the constructive suggestions. We have revised the results section to improve clarity and logical flow, progressing from descriptive findings to model comparison and finally determinants in final multilevel regression model. In line with these recommendations, we now present a single concise table reporting adjusted odds ratios (ORs) and 95% confidence intervals for all predictors retained in the final model. Bivariable analyses were used solely for variable screening and are described in the Methods section; to enhance readability and avoid redundancy, the bivariable regression table has been removed from the main manuscript and is provided as Supplementary Material. Kindly see amendment in result section on page 10-18-line number 250-401.

**Comment #9** The Discussion should minimize repetition, synthesize results thematically, and focus on explaining mechanisms (e.g., why socioeconomic, contextual, and care-utilization factors influence barriers), including regional differences between East and West Africa and comparisons with 3–5 recent studies. Emphasize interpretation over description and conclude with clear policy implications aligned with UHC, gender equity, and national health priorities**.**

**Response: Dear Reviewer,** we appreciate detail revision. The Discussion has been revised to reduce repetition, synthesize results thematically, focus on underlying mechanisms and regional differences, compare with recent studies, and emphasize policy implications aligned with UHC, gender equity, and national health priorities. Kindly see amendment in discussion section on page 16-18-line number 320-398.

**Reviewer four**

**Comment #1** This should be Results, this sentence is not clear. Are these the main "barriers"?,

**Response:** Dear reviewer, Thank you for the comment. We have revised this sentence for clarity. Kindly see amendment on abstract result section on page 2-line number 47-50

**Comment #2** "individually associated variables"? If so, what are the ones of the previous sentence? Are these "community associated variables"? [needs to be described better]

**Response: Dear Reviewer,** Thank you for the comment. We have clarified the terminology by explicitly defining “individual-level variables” and “community-level variables” in the text and clearly specifying which variables belong to each category in the revised manuscript. Kindly see amendment on page 2 linen number 51-57.

**Comment #3** There is a need to include some form of theoretical model to explain how the independent variables were selected. You have put a few previous studies so could use those variables that were found in individual countries affecting health care services...

**Response:** Dear reviewer Thank you for pointing out this concern. The selection of independent variables was guided by the DHS conceptual framework on health service utilization, which classifies determinants into individual-level and community-level factors, and was further informed by evidence from prior DHS-based studies in sub-Saharan Africa. All variables included in the analysis were available in the DHS datasets and selected a priori based on their theoretical and empirical relevance to healthcare access.

**Comment #4** " : " [colon, because you are adding the countries after stating it], and " , " [comma, as a pause before continuing]

**Response:** Dear reviewer, Thank you for the comment. We have revised the punctuation accordingly by using a colon to introduce the list of countries and a comma where a pause was required for clarity. Kindly reamendment on method section line number 143-145.

**Comment #5** Not clear how they were selected and recoded. Rephrase again

**Response**: Dear reviewer, Barriers to healthcare access were constructed from four DHS variables (v467b, v467c, v467d, and v467f) in the maternal recode dataset. Women reporting at least one item as a ‘big problem’ were coded as having barriers. For farther clarification kindly see in method section outcome variable determination kindly on page 7-line number 167-176.

**Comment#6 "as factor barriers", "barriers" [plural], "therefore", "weigh", "used"**

**Response:** Dear reviewer, Thank you for the comment. The wording has been corrected to ‘barrier factors’/‘barriers’ (plural), ‘therefore,’ ‘weight,’ and ‘used’ in the revised manuscript. Kindly see correction on age 6-line number 152-159.

**Comment #7** Why divided by 1 million? Needs to be explained.

**Response:** Dear reviewer, we divided the DHS sample weight variable (v005) by 1,000,000 to rescale it to its true value. In the DHS dataset, v005 is stored as an integer multiplied by 1,000,000 to avoid decimals. Dividing by 1,000,000 restores the correct weight for analysis and does not relate to the population size. This procedure follows the DHS guideline for using sampling weights.

**Community#8** Why were these variables chosen? Based on previous studies..., etc.? There needs to be some form of theoretical model or using findings from previous studies...etc., that oriented you to use these variables. Otherwise, there is no justification and the reader get confused as to why they in particular were used! Also, these are not "Outcome" variables but more like Independent...can you explain better?

***Response:*** Dear reviewer, thank you for your comment. The independent variables included in our study were selected based on prior research and the DHS conceptual framework for healthcare access. These variables have been consistently identified in previous studies as determinants of barriers to healthcare among women in Sub-Saharan Africa. We have clarified in the manuscript that these are independent variables, while the constructed ‘barrier to healthcare access’ variable is the outcome.

**Comment #9** Please see the manuscript for grammatical consistency, including correct use of singular and plural verbs, spelling, and punctuation (e.g., colons, semicolons, and parentheses).

***Response:*** We have carefully reviewed and revised the entire manuscript to ensure grammatical consistency, including correct use of singular and plural verb forms, spelling, and punctuation (e.g., colons, semicolons, and parentheses). All identified issues have been corrected accordingly. Kindly see amendment in method section line number 140-189.

**Comment #10** singular and plural terms (e.g., outcome(s), barrier(s), variable(s)), grammatical errors, punctuation, and inappropriate terminology (e.g., patients vs. respondents) been addressed in the revised manuscript

**Response:** Dear reviewer, thank detail reviewing, we have carefully revised the manuscript to ensure consistent use of singular and plural terms, corrected grammatical and punctuation errors, and standardized terminology throughout. Kindly see amendment method section line number 190-241

**Comment #**11 Patient and public involvement

**Response:** Dear reviewer, Thank you for the suggestion. The “Patient and Public Involvement” statement has been relocated to the appropriate section of the Methods. Kindly see correction, on age number 6-line number 138 to 139.

**Comment #**12 from 10 Sub-Saharan African countries", results, grammatical issue,

**Response:** Dear reviewer, thank detail reviewing, we have carefully revised the manuscript to ensure consistent use of singular and plural terms, corrected grammatical and punctuation errors, and standardized terminology throughout. Kindly see amendment result section line number 241-249.

**Commment#13** Individual Level [need to add sub-title to differentiate the two types of variables], You can use one decimal instead of two, so the reading becomes easier... Status, Husband's Education, Secondary, Working, Single, Place of Delivery, Change to in bold, "Internet, Visited Health Facility Last 12 Months, Poor", "Middle" [capitals], Community Leve, No need to put the percentage signs here!

**Response:** Dear reviewer, thank the reviewer for these valuable and constructive suggestions. Accordingly, we have added appropriate subheadings to clearly differentiate the two types of individual-level variables, simplified the presentation by using one decimal place, corrected capitalization and bold formatting, standardized variable labels, and removed percentage signs where they were not required. These revisions have improved the clarity and readability of the table2. Kindly see correction in result section line number 249 to 252.

**Comment #14** Need to explain better, e.g., "Using the Akaike Information Criteria, among... Not clear what this "fines" means...

**Response:** Dear reviewers, Thank you for this comment. We have revised the manuscript to clearly explain the use of the Akaike Information Criterion (AIC) for model comparison, including its purpose, definition, and the criterion used to select the final model (lowest AIC). Kindly see explanation in result section, line number 256 to 274.

**Comment #15** barriers, reproductive age women, an urban area, level, " [a comma before "respectively"], it those living in East., "1.43)," [comma after the parenthesis, given the length of the sentence], visits, respectively" Use a semi-colon (;) because the sentence is already long and there is more to be said... "internet" "access, " [ALWAYS A COMMA BEFORE RESPECTIVELY!], ";" [semicolon], a health facility", Interesting that women who DID NOT visit a health facility had 9% times LESS barriers...? Will need to be analyzed carefully... change this. E.g., "Additionally, the odds...", "a perceived" community-level

**Response**: Dear reviewer, Thank you for the detailed editorial and interpretative comments. All suggested punctuation, capitalization, and formatting issues (including the consistent use of commas before “respectively”, appropriate use of semicolons in long sentences, and wording clarifications) have been carefully corrected throughout the manuscript. Dear reviewer kindly see correction in result section line number 274 to 313.

**Comment #16** changes in socioeconomic, health infrastructure, health system policies, women's attitudes towards health care seeking behavior, and cultural differences across countries in Sub-Saharan Africa." "Moreover, these discrepancies might be due to differences in the study period, because this study includes the most recent DHS data sets, from 2019 to 2023." his has already been mentioned before! reproductive age, Guinea, be due [no comma needed], infrastructures, i.e., inaccessibility to..., Sub-Saharan countries and other many punctuation error, grammatical, capitalization etc.

**Response:** Dear reviewer, Dear reviewer, we thank for these detail comment. Redundant statements regarding the study period and use of the most recent DHS datasets have been removed to improve conciseness. In addition, all identified issues related to grammar, punctuation, capitalization, and wording (including appropriate comma usage, consistency in terminology such as women of reproductive age, country names, and descriptions of health infrastructure) have been carefully corrected throughout the manuscript. These revisions have improved overall clarity and readability. Kindly see all amendment in discussion, strengthen and limitation conclusion section line number 318 to 411.

**Comment #17** declared that they have no competing interests., Survey data, are; [semicolon, not comma]

**Response:** Dear reviewer, Thank you for the comment. The competing interest’s statement has been revised to read “The authors declare that they have no competing interests.” In addition, grammatical corrections have been made, including changing “Survey data is” to “Survey data are” and replacing commas with semicolons where appropriate**.** kindly see amendment line number 419-432.

**Comment 18. THIS SEEMS TO BE THE SAME TABLE...IS IT DIFFERENT?**

**Responses:** Thank you for the comment. We agree with the reviewer that the tables 5 were redundant. Therefore**, Table 5 has been removed from the revised manuscript**, and the relevant information is adequately presented in the remaining table. Kindly see amendment in manuscript.
